# Supplementary material for: RECORD, a high-throughput, customizable system that unveils behavioral strategies leveraged by rodents during foraging-like decision-making
Source: Commun Biol. 2024 Jul 6;7:822. doi: 10.1038/s42003-024-06489-8 (PMC11227549; doi:10.1038/s42003-024-06489-8)
Supplement: Supplementary file 2 — Supplementary Information [file 42003_2024_6489_MOESM2_ESM.pdf]

Supplemental Figure 1

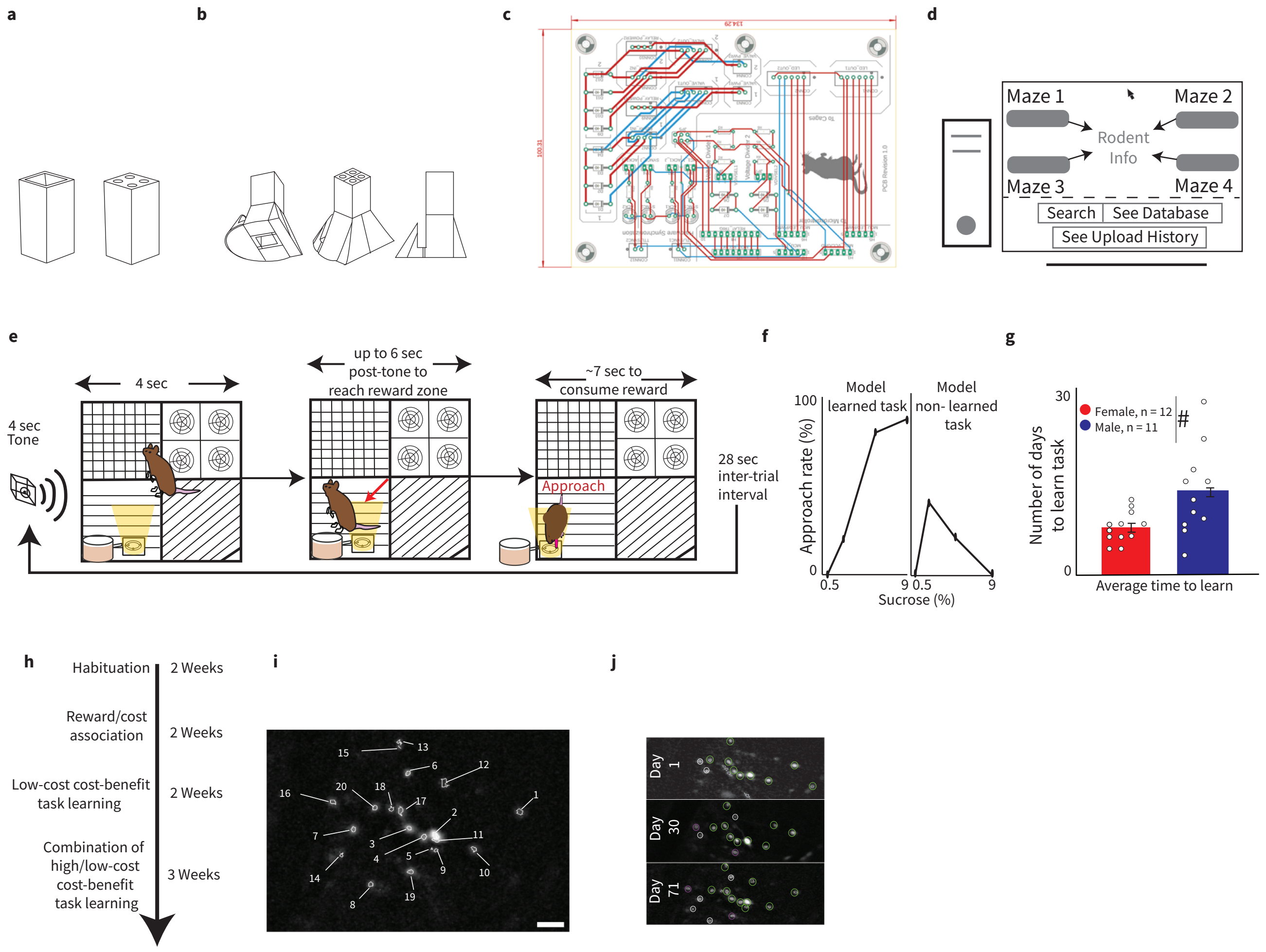

Supplemental Fig. 1: System Components and Task Training

a. 3D-printed floor support pillars.

b. 3D-printed wall support pillars. The walls slide into the slot on the sides of the pillars. The walls are easily removed, thus enabling walls with unique features to be inserted.

c. Custom printed circuit board (PCB) used to interface with the microcontroller. Illustration of the connections on the PCB.

d. The RECORD system generates large datasets. We created a customized parser and database management tools that can handle the varying subjects and parameters required during experimentation. For validation of this project, we have recorded 39381 sessions across 103 animals, across 159185 trials, all of which our system has injected into a standard PostgreSQL database prepared for future analysis.

e. Example trial of decision-making task. Each trial consists of four main phases: Tone presentation, marking the beginning of the trial; offer presentation, where light is presented at a corner of the arena to represent a cost-reward pairing; the approach/avoid phase, where the animal either approaches or ignores the offer; and finally, the delivery phase, where the reward is dispensed if the offer was accepted. Each trial is separated by a 28-second inter-trial interval (ITI). During the ITI, the system is reset, and hardware is re-synchronized (for a more detailed breakdown of a trial please see “A note about RECORD and Noldus Ethovision” in supplemental materials).

f. Example sessions of individual rats performing the reward/association task. One example session where the rat learned to approach the reward (left) contrasted with a single session where the rat has not yet learned (right) is shown. Error bars = mean ± SEM for all plots.

g. Graph demonstrating the average time to learn the task in males and females (paired t-test *p = 0.01, mean ± SEM).

h. Approximate training timeline. Following habituation to the arena and association of light and sucrose to cost and reward, the rats were eased into the low-cost cost-benefit task first. After a 2-week training period, they were introduced to the high-cost cost-benefit task through a combination of low and high-cost trials randomly distributed within the same session.

i. Cell map of extracted cells using PCA-ICA from an example behavioral session. Somas of individual cells are numbered. Scale bar = 100 μm.

j. Longitudinal tracking of cells spanning 71 days. Cell maps from three separate sessions with individual cells color coded according to the number of sessions in which they were detected (green = 3 sessions, purple = 2 sessions, white =1 session).

Supplemental Figure 2

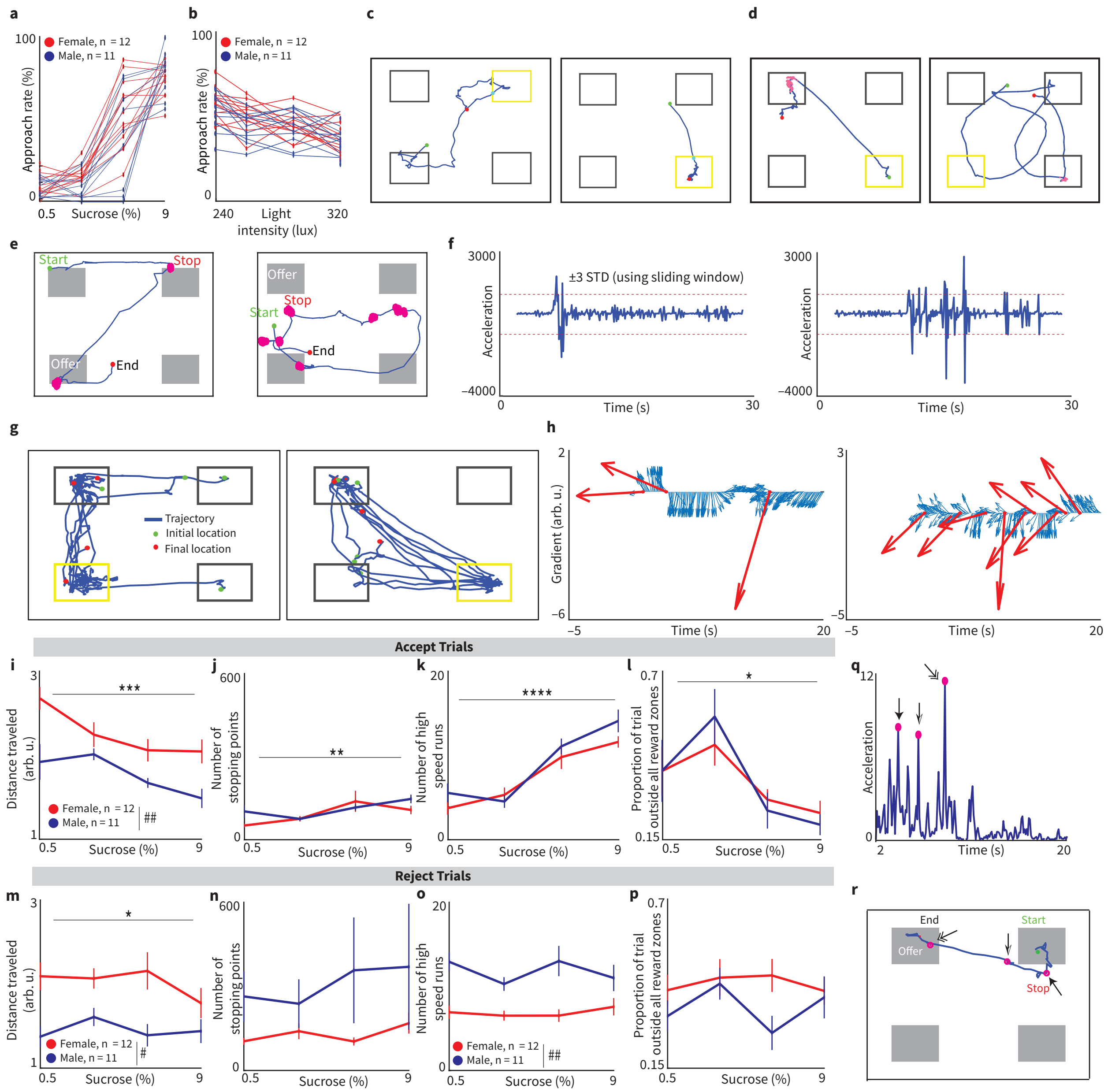

Supplemental Fig. 2. Relevant decision-making features measured using rats' timing of choice and spatial location during the task.

a. Approach rates across reward levels for individual rats. Error bars = mean ± SEM for all plots.

b. Approach rates across cost levels for individual rats.

c. Example trajectory when a low SC is offered (left) and when a high SC is offered (right, green = trial start point, red = trial end point, pink = stopping points).

d. Trajectory examples tracked for two different rats during a trial. Some trajectories are direct (left) while others are circuitous and indirect (right).)

e. We developed an algorithm that identifies when the rat does not move 0.1 units in the x or y direction for 3 seconds (a "stopping point") during a task.

f. We developed an algorithm that identifies high accelerations during individual rat trajectories. High acceleration is identified by detecting speeds that are two standard deviations above the mean (dotted line).

g. Multiple trajectories for a single rat on different trials are shown.

h. Rotation points (red arrows) are identified when the orientation of a rat's body changes at least 180 degrees within 0.3 seconds.

i-l. Complementary to Fig. 2e-h, with analysis limited to trials in which the offers were approached (excluding "reject" trials). Distance traveled during approach trials was impacted by both SC (**i,** $ANOVA_{RM}$ ***p = 0.00017) and sex (##p = 0.0038). Number of stops during approach trials were significantly affected by SC (**j,** **p = 0.0015) but not sex (p = 0.5). Frequency of high-speed runs during approach trials was significantly affected by SC (**k,** ****p < 0.0001) but not sex (p = 0.22). Time outside feeder zones during approach trials was also affected by SC (**l, *****p = 0.0003) but not sex (p = 0.9).

m-p. Limiting the analysis to trials in which the offers were rejected (not approached), number of high-speed runs (##p = 0.0022) and distance traveled (#p = 0.014) were significantly different between sexes. Distance traveled also decreased as concentration increased during reject only trials (*p = 0.047). Number of stopping points (p = 0.22) and proportion of trial outside all reward zones (p = 0.3) had no significant sex or concentration interactions (see **Methods: Statistics and Reproducibility** for all statistics).

q-r. Example of direct relation between acceleration **(r)** and animal location **(s)** where stopping points are temporally localized. This shows that RECORD has sufficient temporal resolution to provide precise timestamps that can be related across "features". Arrows indicate sharp increase in acceleration (left) that correspond to movement initiation (right).

Supplemental Figure 3

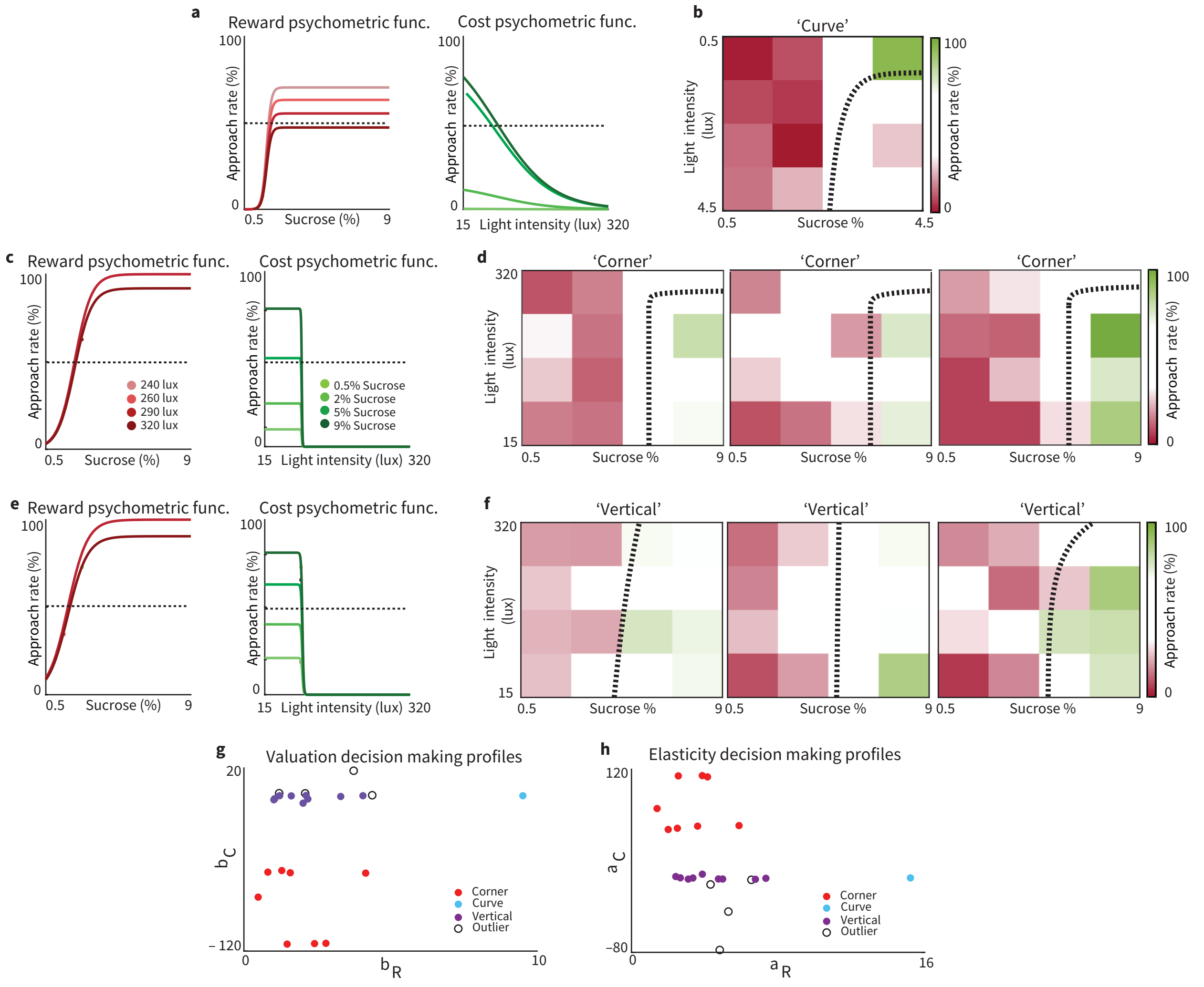

Supplemental Fig. 3: Neuroeconomic modelling with RECORD data.

a. Two-dimensional representations of the 3D plots in Fig. 3c, like the stacked classical 2D psychometric functions that form the middle and right panels of Fig. 3b. Here, the data appear noisy when shown in either cost (left) or reward (right) slices. Yet after fitting through three dimensions (see Fig. 3 eq. 3), a pattern emerges that can be used to describe the animal's decisions.

b-d. Samples of three common decision-making profiles, the first **(b)** which we term a "corner," where the dashed line bends sharply such that the animal only approaches >50% of the time in the bottom right corner of the plot; second **(c)**, which we term a "vertical," where the animal approaches >50% across the entirety of a right region of the plot., and third which we term a "curve" were animal gradually shift from avoid to approach (**d**).

e,f. Across animals, these classifications correspond to clusters in the parameters of fit a_R, b_R,

a_C, b_C, which together form a way to quantify features of decision-making. "Curve": like a "corner" but with a gradual (maximum curvature K_{max}, normalized on a [0, 1] scale, < 0.5) bend in the 50% approach line; "outlier": assigned to animals that are not "corner," "curve," nor "vertical."

Supplemental Figure 4

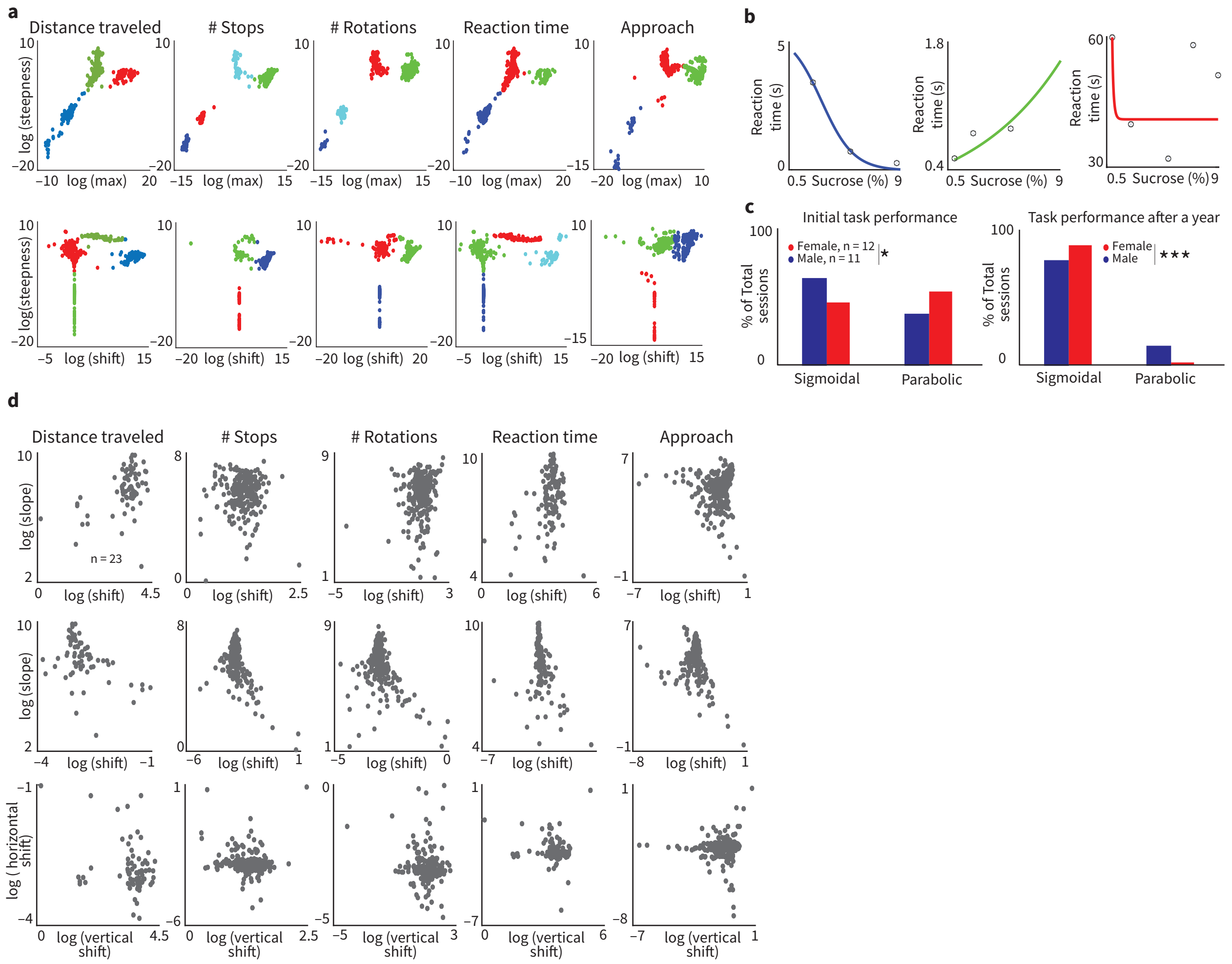

Supplemental Fig. 4: Further examples of RECORD's ability to generate functions used to develop clusters of decision-making schemas and more examples of how clusters can be analyzed.

a. Clusters calculated using steepness and max/shift parameters generated using the same technique described Fig. 4d.

b. The blue and green clusters of reaction time are comprised of distinct psychometric functions, unlike the red cluster**.**

c. During the first three months of task performance, psychometric functions are roughly split between sigmoid and parabolic. After a year, psychometric functions are almost entirely mostly (parabolic vs sigmoidal shape for 1-3 months of task performance *p = 0.016, chi-square test, 4 ± 2 months; parabolic vs sigmoidal shape for 10 ± 2 months of task performance ***p = 0.0009, chi-square test).

d. We were unable to identify distinct clusters using features of parabolic psychometric functions.

Supplemental Figure 5

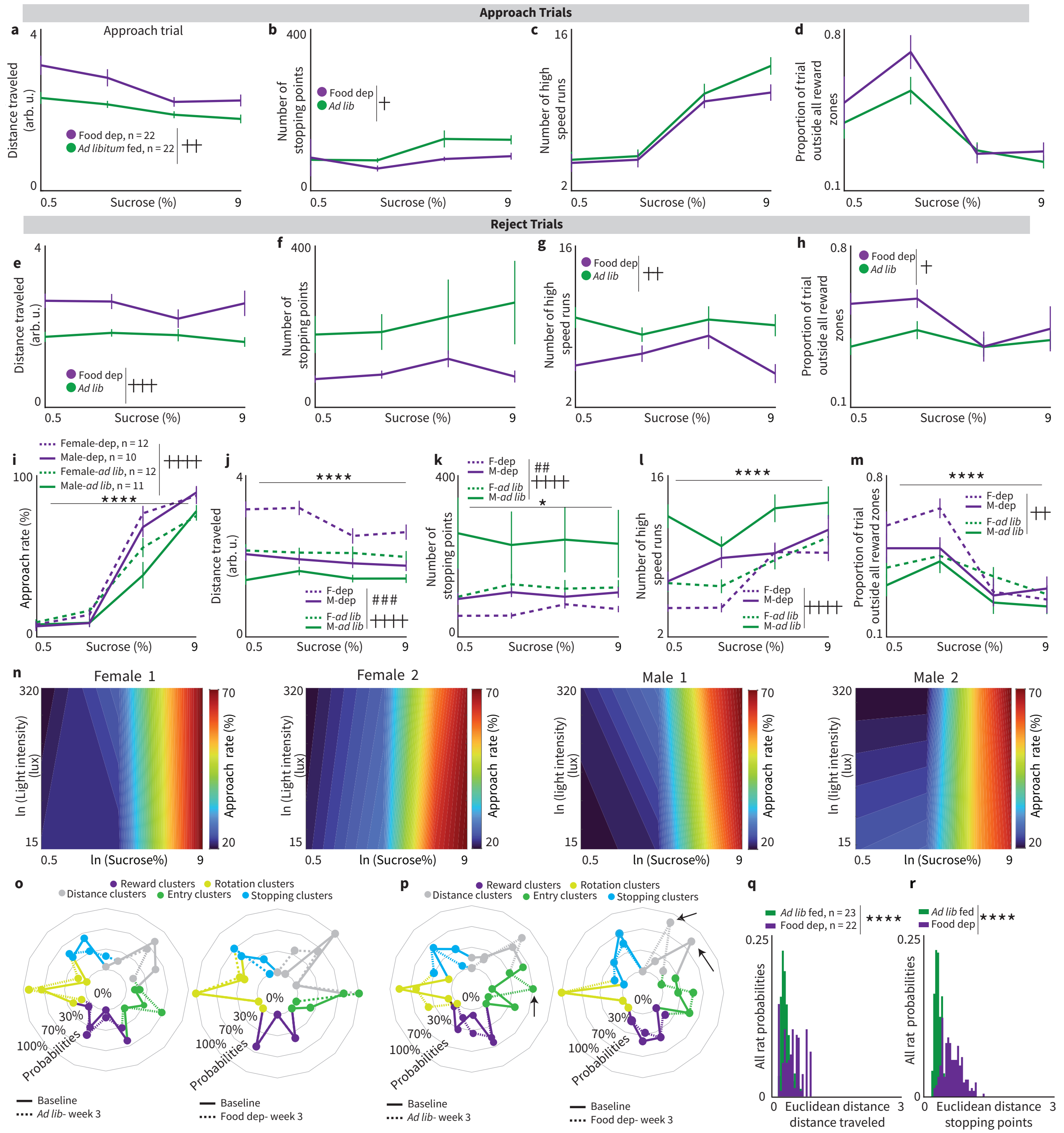

Supplemental Fig. 5: Additional food deprivation data

a-d. During approach-only trials, the distance traveled was significantly greater during food deprived sessions (main effect of deprivation, ++p = 0.0033) while the number of stopping points decreased in sessions conducted under food deprivation (main effect of deprivation, +p = 0.021). In contrast, deprivation did not impact the number of high-speed runs (p = 0.4263) or proportion of trial spent outside all reward zones (p = 0.08). Error bars = mean ± SEM for all plots.

e-h. During reject-only trials, the distance traveled was significantly greater during sessions conducted under food deprivation (main effect of deprivation, +++p = 0.0002), while the number of stopping points was unaffected (p = 0.14) However, the number of high-speed runs is significantly lower under deprivation (++p = 0.0034) and proportion of the time spent outside all reward zones is enhanced by deprivation (+p = 0.012).

i-m. Sex differences in behavioral features are still present during food deprivation, however these sex differences shift. Approach rate, number of acceleration points, and time outside feeder have similar patterns of significance before and during food deprivation. Distance traveled had concentration become significant ($ANOVA_{RM}$ ****p < 0.0001) while sex remained significant (###p = 0.0002). Number of stopping points had significant differences between both concentration ($ANOVA_{RM}$ *p = 0.01) and sex ($ANOVA_{RM}$ ##p = 0.007) compared to both concentration and sex being insignificant before food deprivation. Figure 2 data was replotted for comparison as dashed lines. When comparing across sex, sucrose concentration, and food deprivation vs. *ad libitum* fed every feature was significantly different across food deprivation (main effect of condition, approach rate: ++++p <0.0001, distance traveled: ++++p < 0.0001, number of stopping points: ++++p < 0.0001, number of high-speed runs: ++++p < 0.0001, and proportion of trial outside all reward zones: ++p = 0.0026).

n. Examples of individual cost-benefit maps from four different food-deprived rodents (2 females, left, and 2 males, right).

o-p. Food deprivation impacts the clustering of behavioral features. Radar plot showing the cluster distribution compared between *ad libitum* fed and food-deprived conditions. Two examples with small changes in Euclidean distance (**o**) and changes in Euclidean distance (**p**). Arrows indicate clusters of behavioral features that display the greatest changes between conditions.

q-r. Using the average cluster distribution of *ad libitum* fed rats, we created a distribution. We then calculated the Euclidean distance of each cluster of distance between ad libitum fed and food deprivation conditions for both distance traveled (**q**) and stopping points (**r**)and found that food deprivation shifted the peak completely outside of the baseline normal distribution. These shifts were statistically significant for both groups (****p < 0.0001, determined by two-sample Kolmogorov-Smirnov test).

Supplemental Figure 6

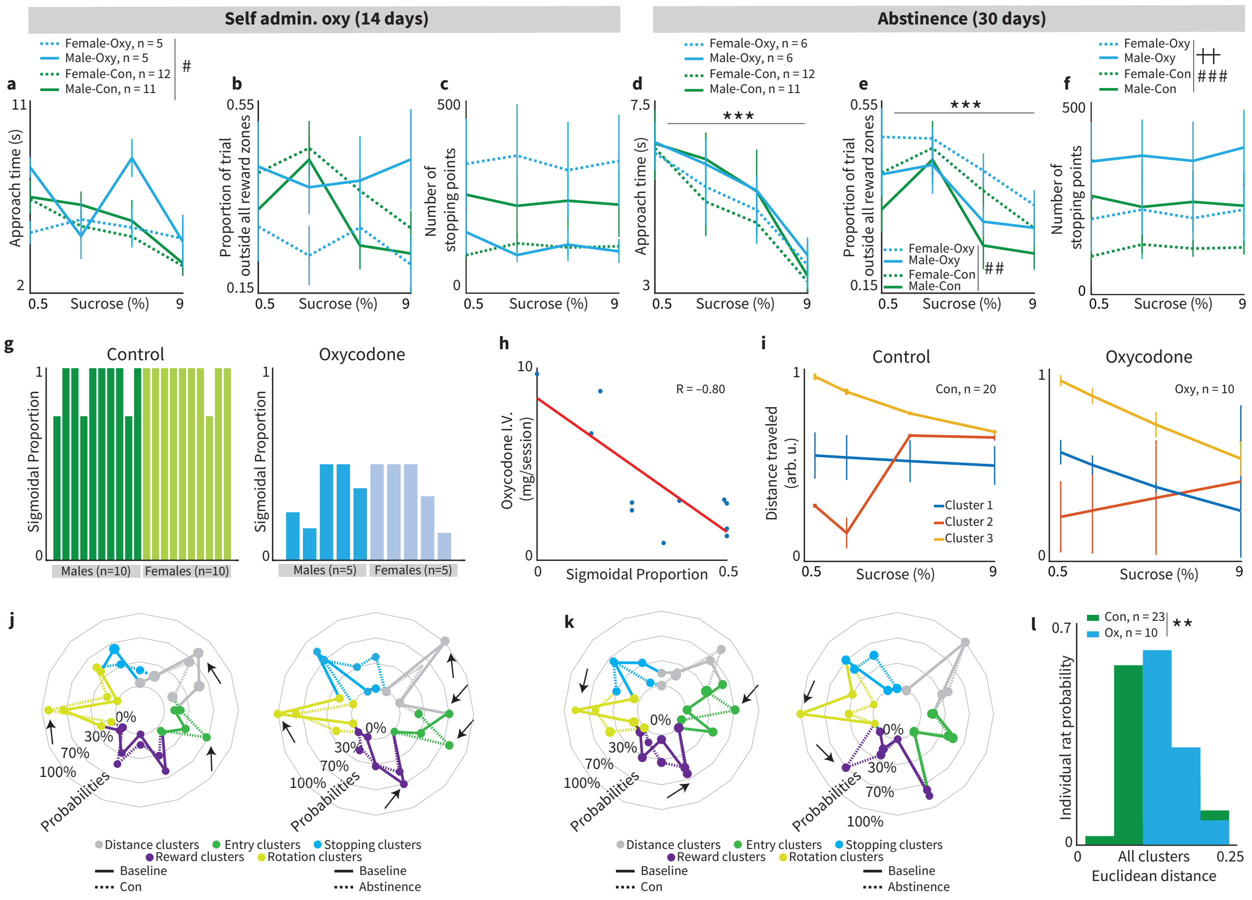

Supplemental Fig. 6: Additional Oxycodone data.

a-c. (**a**) Approach time during self-administration task performance was significantly different between sexes (effect of sex #p = 0.0315), primarily due to the spikes seen in males at 0.5% and 5% sucrose concentrations, whereas concentration had no significant effects. No significant differences were found when analyzing interactions between sex, concentration, and condition (n-way ANOVArm, sex x condition, sex x concentration, and condition x concentration). Error bars = mean ± SEM for all plots. (**b**) Proportion of trials outside feeder zone had no significant interactions between concentration or sex. No significant factors were observed across control and self-administration while the only significant interaction detected was between sex and condition (p = 0.0005, n-way ANOVArm). (**c**) The number of stopping points similarly had no significant effects of concentration or sex. When compared to the control group there were no significant main effects while there was a significant interaction between sex and condition ($p < 0.0001$, n-way ANOVArm).

d-f. After oxycodone, features seem to trend towards pre-oxycodone shapes. (**d**) The effect of sucrose concentration went from not being significant during oxy self-administration to becoming significant again (***p = 0.0003, n-way ANOVArm), with no significant interactions between the control group and abstinence conditions. (**e**) The proportion of the trial spent outside all feeder zones decreased as sucrose concentration increased with no significant interactions detected between conditions, meanwhile the main effect of sex (p = 0.0063) and concentration (***p = 0.0006, n-way ANOVArm) became significant between baseline and abstinence groups. (**f**) When comparing abstinence groups, the number of stopping points remained insignificant across concentration and sex. However, when compared to the control group there were significant main effects of sex (###p = 0.0005) and condition (++p = 0.006, n-way ANOVArm) with no significant interactions between sex, condition, or concentration.

g. Individual sigmoid frequencies across control and oxycodone conditions.

h. Correlation plot between amount of oxycodone self-administered and sigmoid frequency. We were unable to track the opioid self-administration of one rat due to technical difficulties.

i. Average psychometric functions extracted from distance traveled clusters between control (left) and oxycodone (right) conditions.

j,k. Oxycodone impacts the clustering of behavioral features. Radar plot showing the cluster distribution compared between baseline and oxycodone conditions. Some rats have small changes in Euclidean distance (**j**) or large changes (**k**). Arrow indicates a feature that has shifted.

l. Individual Euclidian distances between clusters for baseline and oxycodone self-administration conditions (**p = 0.0011, two-sample Kolmogorov-Smirnov test).

Table 1: Method Comparison

**Features**	RECORD	Manual t-mazes[8,19,21,59]	Automatic t-mazes[5,6]	Virtual reality tasks[7,70]	Operant Chambers (lever pressing and nose pokes)[12,17,18,24,71,72]	Wheel Displacement[10,73]	Rodent Iowa Gambling Task[16,46,47]
Can offer numerous levels of rewards and costs during *a single session*	✓	✗	✗	≈	≈	≈	✗
High sensitivity and efficient neuroeconomic modeling	✓	✗	✗	✗	✗	✗	≈
Does not utilize food or water restriction	✓	≈**	✗	✗	✗	✗	≈
Combines three established principles for substance use disorder research (conditioned place preference, runaway models, and self-administration)	✓	✗	✗	✗	✗	✗	✗
Customizable for a variety of DM tasks	✓	≈	≈	≈	≈	≈	≈
Automatic/high throughput	✓	✗	✓	≈	✓	✓	≈
Economical (< $350 per maze)	✓	✓	✓	≈	✓	✓	✓

Mimics foraging (ethologically valid)	✓	✗	✗	≈	≈	✗	≈
Free navigation in open field environment (ethologically valid)	✓	✗	✗	✗	✗	✗	✗
Short training time	✓	✗	✓	✓	✓	✓	✓
Compatible with multiple neuronal recording systems	✓	✓	✓	✓	✓	✓	✓
Integration with two-photon microscopy	✗*	✗*	✗*	✓	✓	✓	≈

* The development of two-photon microscopy systems that allow for a rodent to move will allow these behavioral systems to implement two-photon microscopy.
** The spontaneous t-maze is an exception and does not require food or water restriction.

Table 2: Resource Table

REAGENT or RESOURCE	SOURCE	IDENTIFIER
Deposited data		
Custom RECORD hardware, software, and scripts		
RECORD Microcontroller Firmware		https://github.com/rjibanezalcala/RECORD/tree/main/microcontroller
RECORD Printed Circuit Board Design Files		https://github.com/rjibanezalcala/RECORD/tree/main/pcb/Revision%201.0
3D printing RECORD CAD files		https://github.com/rjibanezalcala/RECORD/tree/main/3d-prints/cad
3D printing RECORD STL files		https://github.com/rjibanezalcala/RECORD/tree/main/3d-prints/stl
Ethovision XT 16 Experiments		https://github.com/rjibanezalcala/RECORD/tree/main/ethovision_experiments
Bonsai Workflows		https://github.com/rjibanezalcala/RECORD/tree/main/bonsai_workflows
RECORD Matlab library		https://github.com/rjibanezalcala/RECORD/tree/main/matlab_app
RECORD Python library (RECORD-lib)		https://github.com/rjibanezalcala/RECORD/tree/main/python/RECORD-lib
SerendiPYty		https://github.com/rjibanezalcala/RECORD/tree/main/python/SerendiPYty

Feature extraction scripts		https://github.com/atanugiri/Feature-Extraction
Serendipity app with built-in behavioural data parser		https://github.com/lddavila/UTEP-Brain-Computation-Lab-Remote-Databases-and-Serendipity-App/tree/main/App%20Deployment%20Folder
Psychometric function shape-fitting analysis and<br><br>clustering analysis scripts		https://github.com/lddavila/UTEP-Brain-Computation-Lab-Remote-Databases-and-Serendipity-App/tree/main/Updated%20Analysis
Supplemental notes (guides and documentation)		
RECORD system documentation (user guide)		https://github.com/rjibanezalcala/RECORD/blob/main/documentation/RECORD_User_Manual.pdf<br><br>Supplementary note 1
RECORD electronics build guide		https://github.com/rjibanezalcala/RECORD/blob/main/documentation/electronics_build_guide.pdf<br><br>Supplementary note 2
RECORD arena setup guide		https://github.com/rjibanezalcala/RECORD/blob/main/documentation/arena_setup_guide.pdf<br><br>Supplementary note 3
Database and Serendipity user guide		https://github.com/lddavila/UTEP-Brain-Computation-Lab-Remote-Databases-and-Serendipity-App/blob/main/Supplemental%20Note%204%20Database.docx<br><br>Supplementary note 4

Shape fitting and cluster analysis documentation and figure guide		https://github.com/lddavila/UTEP-Brain-Computation-Lab-Remote-Databases-and-Serendipity-App/blob/main/Supplemental%20Note%205%20Decision%20Making%20Schemas.docx<br>Supplementary note 5
Statistics and reproducibility documentation		https://github.com/atanugiri/Data-Analysis/blob/main/statistics and reproducibility.pdf
Figure reproduction and documentation		
Calcium Trace Analysis Code		https://github.com/lrakocev/inscopix
Neuroeconomic Analysis Code		https://github.com/rjibanezalcala/RECORD/tree/main/data_analysis/neuroeconomic_analysis
Data analysis codes		https://github.com/atanugiri/Data-Analysis/tree/main/Data Analysis
Shape fit and clustering analysis code		https://github.com/lddavila/UTEP-Brain-Computation-Lab-Remote-Databases-and-Serendipity-App/tree/main/Updated%20Analysis
		https://github.com/WhiteHatArnav/RECORDFiguresCode/tree/main

Raw data		
Manual Excel calculations		https://doi.org/10.7910/DVN/QADUKS
Raw Ethovision data		https://doi.org/10.7910/DVN/QADUKS
Oxycodone data		https://doi.org/10.7910/DVN/QADUKS
PostgreSQL database backups		https://doi.org/10.7910/DVN/QADUKS
Experimental models: Organisms/strains		
Rat: Long Evans HsdBlu:LE Male	Envigo	14001M
Rat: Long Evans HsdBlu:LE Female	Envigo	14001F
Software and algorithms		
Autodesk AutoCAD 2022 or later	Autodesk	https://www.autodesk.com/products/autocad/overview

Autodesk EAGLE 9.6.2 or later	Autodesk	https://www.autodesk.com/products/eagle/free-download
Code Composer Studio 10.4.0 or later	Texas Instruments	https://www.ti.com/tool/download/CCSTUDIO/10.4.0.00006
MATLAB R2021a	Mathworks	https://www.mathworks.com/products/matlab.html
PostgreSQL 14.2	PostgreSQL	https://www.postgresql.org/download/
PuTTY 0.76 or later	PuTTY	https://www.chiark.greenend.org.uk/~sgtatham/putty/latest.html
Ethovision XT16	Noldus	https://www.noldus.com/ethovision-xt
Bonsai	Open Ephys	https://bonsai-rx.org/
Other		
Through-hole LED (blue)	Digikey	C503B-BAS-CY0C0461-ND
Through-hole LED (red)	Digikey	VLCS5830-ND
Solid hook up wire kit, 22 gauge	Electronix Express	27WK22SLD100
Dupont wire female to female	Digikey	1568-1588-ND
Dupont wire male to female	Digikey	1568-1578-ND

Speaker Wire Spool, 2-conductor	Local Hardware Store	N/A
CAT5 ethernet cable, 10 ft.		
Qibaok 760 pcs JST-XH connector kit, 2, 5, and 6 pin male and female connectors, 2.54 mm pitch	Qibaok	Qibaok535
Qibaok 1550 pcs dupont connector kit, 2.54mm pitch	Qibaok	Qibaok536
Through-hole Resistor Kit	Sparkfun electronics	COM-10969
Through-hole Diode	Digikey	P/N:1N4007E-E3/54
MSP430-FR2355 Development Kit	Texas Instruments	MSP-EXP430FR2355
SunFounder 5V 8 Channel Relay Shield Module	SunFounder	Relay: Songle - SRD-05VDC-SL-C<br>Amazon ASIN: B00DR9SE4A
Formlabs Tough 2000 v.1 resin	Formlabs	RS-F2-TO20-01
Resin tank V2	Formlabs	RT-F3-02
Build platform	Formlabs	
Form3 printer, wash, and cure	Formlabs	SKU: PKG-F3-SVC-COMPLETE
Glue	GorillaGlue	Mfr#5003601

Metal jack screw standoff #4-40	StarTech.com	SCREWNUTM4_40
Screw M3 X 1/4"	StarTech.com	SCREWM3
Screw #4-40 x 3/16"	StarTech.com	SCREW4_40
GigE Camera with IR Pass Filter	Basler	M106580-12; acA1300-60gc
Infrared illuminator	Axton	Smart AT-11S
Ethovision XT USB-IO Box	Noldus	00955-000
Opaque white HDPE sheet	Grainger	226-CXG-44ZT07
Versilon™ 2001 1.6 mm internal diameter	Masterflex	HV-06475-14
60mL Plastic Syringes	Fisherbrand	14955461
ASCO Solenoid Valves 24/60V	Valin	SC8256B45V
Scientific Stands		
Autopole	Noldus	Autopole
Superclamp for Autopole	Noldus	Superclamp for Autopole
Four-port video capture card	Noldus	Four Port Video Capture Card

Supplemental Note 1

R.E.C.O.R.D.

(Reward-Cost in Rodent Decision-making)

User Manual

Revision 1.1

Background information

This guide will talk specifically about the Texas Instruments MSP-EXP430FR2355 launchpad development kit (https://www.ti.com/tool/MSP-EXP430FR2355) and about microcontroller features specific to the RECORD system. Pin numbers, peripherals, and connections will differ between microcontrollers from the same and different manufacturers. Therefore, we cannot guarantee that this guide will be accurate for any other microcontroller. For a detailed user guide on this microcontroller, please see the manufacturer user guide ((https://www.ti.com/product/MSP430FR2355).

How LED brightness is controlled

The RECORD system microcontroller uses Pulse Width Modulation (PWM) to regulate the brightness of the LEDs which encode cost levels. A total of four PWM signals are produced by the microcontroller, one for each feeder. The amount of time within the PWM signal's period that the signal is on (logical high) is defined as the **duty cycle**. A higher duty cycle will result in a brighter LED, while a lower duty cycle will result in a dimmer LED. The **duty cycle** is dependent on two **Capture/Compare Register (CCR) values**; CCR0 and CCR1, which are defined in the microcontroller firmware.

The duty cycle is defined by the formula:

$$DC(\%) \; = \; 1 - \frac{on\ time}{signal\ period},$$

where *signal period* is the length of time it takes for the signal to complete a full cycle, and *on time* is the amount of time the signal is held at a logical high. We can expand this formula to be in terms of our two **CCR values**:

$$DC(\%) \; = \; 1 - \frac{CCR1}{CCR0},$$

Where CCR0 defines how many **ticks** it will take to count up to the signal's period and sets the signal to a logical high, and CCR1 is how many **ticks** it takes to bring the signal back down to logical 0.

To explain **ticks**, we must first talk about the microcontroller's internal clock. The microcontroller chip runs at a certain speed determined by an internal clock, which is driven by a crystal oscillator. In the RECORD system, the oscillator signal is internally divided and fed to the system clock, which produces an 8 MHz clock signal, this is set as the **system clock**. Each cycle in the system clock signal is defined as a **tick**.

With the system clock set to a frequency of 8MHz, we can define the period of the PWM signal as 1 millisecond by setting CCR0 to a value of 8000. We determine this value by using the formula:

$$CCR0 = \frac{Desired\ PWM\ period}{Clock\ period}$$

We want a PWM period of 1 millisecond since this value is relatively easy to work with, so plugging the values in , we get:

$$CCR0 = \frac{0.001}{8{,}000{,}000^{-1}} = 8000.$$

CCR1 is a similar but user-variable value which defines the amount of **ticks** it should take for the signal to be set to a logical low. This in turn defines the width of the signal pulse. As CCR1 approaches CCR0, the width of the pulse will increase, making the LED glow brighter, as CCR1 approaches 0 the shorter the pulse will be and the dimmer the LED will glow. Since CCR1 is the only parameter that can be adjusted, we will refer to it only as the **CCR value**. Thus, the driving factor in regulating the cost intensity is the **CCR value**, a whole number ranging from 0 to a maximum of 8000.

In summary, the brightness of the cost LED can be controlled by varying how long in the signal period the signal is kept at logical high vs logical low. This is defined as the duty cycle of the PWM signal. The rest of this guide will be referencing the **CCR value** for instructions.

Lastly, we must relate LED brightness (Lux) to the **CCR value**. It is also important to relate these two parameters to the duty cycle of the PWM signal, as the brightness of the cost LED will be dependent on the shape of the PWM signal and not voltage. We do this in our cost LED calibration document, linked below:

LED Calibration Data - Google Sheets

What are cost levels?

Cost levels are discrete brightness levels that the microcontroller can set. As mentioned before, cost levels are dictated by the **CCR value**. It is important to note that the **CCR value** and **LED brightness** have an inverse relationship, meaning that as the **CCR value** approaches its maximum of 8000, the **LED brightness** will approach 0, or the LED will be dimmer until it turns off. Cost levels are measured in **Lux**, which is a distance-dependent brightness unit measured using a **lightmeter**. We typically measure Lux at 3 mm or less away from the LEDs. Each cost level is calibrated to a range of Lux values. In rodents, bright light induces an aversive reaction, which is why light was chosen to serve as a cost in our cost/benefit tasks. **Brightness requirements may change from rat to rat, for this reason, it is important to calibrate!**

The following are general guidelines of how many Lux are required for each cost level. Always check with your section supervisor or the PI before determining what is needed at each level.

- Cost level 0 **(L0, 0 Lux)** represents an LED which is off. This presents no cost to the rat in the arena. It is typically not used in an experiment setting outside of simply turning lights off, though some special cases may apply. This level is not configurable.

- Cost level 1 **(L1, 7-15 Lux)** corresponds to the lowest brightness . This presents a low cost for the rat in the arena to obtain its reward. We typically keep this level low enough to not scare a rat, but high enough for them to see the light.

- Cost level 2 **(L2, 40-60 Lux)** corresponds to an intermediate brightness between the lowest and highest. It is associated with a mid-level cost. This level should be high enough to be somewhat uncomfortable, but not so bright that the rat never approaches offers associated with this cost.

- Finally, cost level 3 **(L3, 140-320 Lux)** corresponds to the highest (safe) brightness for our experiments. The highest Lux that an LED can output will depend on the LED itself, but it should never shine so bright that the rat is traumatised by it and becomes conditioned to hate running our experiments.

Cost levels are calibrated using the program PuTTY, which has a command line analogue called Plink. This guide has a section on using PuTTY to explain how calibration and reconfiguration is done, as its graphical user interface is somewhat friendlier for the average user.

What are reward levels?

Reward levels are not so much dictated by the microcontroller, but rather the rewards made available on the RECORD arenas. However, the microcontroller sees reward levels as the relay which activates the solenoid valve that dispenses a reward to a particular feeder, thus it is extremely important that each solenoid valve and feeder is associated with the correct relay port. When it comes to reward, the only thing the microcontroller can do is select a particular feeder to dispense reward to, and hold a relay closed for a set amount of time so that the solenoid valve dispenses more or less solution into a feeder (see "Configuring relay active time").

Communicating with the RECORD microcontrollers

Using PuTTY to communicate with RECORD

PuTTY is an SSH/Telnet client which also has the capability to serve as a serial communications interface between a PC and a serial device connected through a USB COM port, such as a microcontroller running the Universal Asynchronous Receive Transmit (UART) protocol. *In other words, you use PuTTY to communicate to the RECORD system microcontrollers while they are connected via USB cable to a PC.* Multiple PuTTY sessions may be loaded at the same time to communicate with each microcontroller, but two sessions cannot communicate with the same microcontroller. *This section will walk you through how to go through the process of calibrating each feeder to a desired lux level.*

This guide assumes a Windows PC with Code Composer Studio (https://www.ti.com/tool/CCSTUDIO) and PuTTY (https://www.putty.org/) already installed is being used, and that a microcontroller running *firmware version v1.2 or later* has been plugged into your PC.

Setting PuTTY up for the first time

1. On the RECORD microcontroller, make sure that the top RXD and TXD pins on the top board-to-board header are jumped to P1.6 and P1.7, respectively.
2. Connect the RECORD microcontroller to your computer via USB.
3. On Windows, open the device manager by pressing and holding the Window key and pressing the ‘R’ (‘Run’) key to open the run dialog. Type ‘devmgmt.msc’ into the dialog box and press Enter.

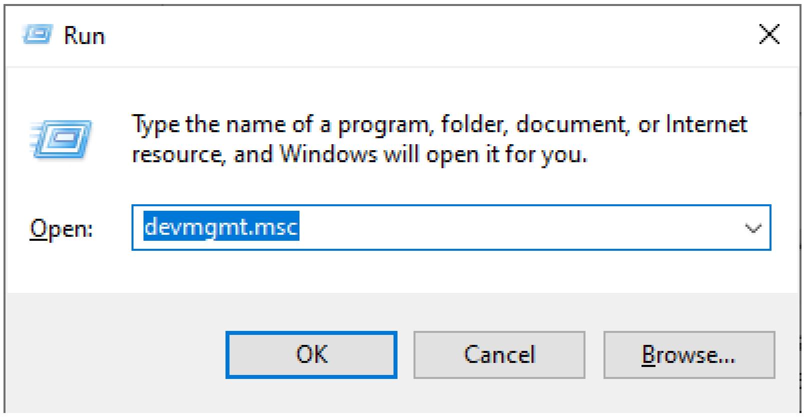

4. Navigate down to ‘Ports (COM & LPT)’ and expand the dropdown menu. Look for ‘MSP Application UART1’ and take note of the COM port number listed here (in parenthesis). If you do not see this entry, make sure that you have Code Composer Studio with the MSP430 library and drivers installed in your system (https://www.ti.com/tool/CCSTUDIO).

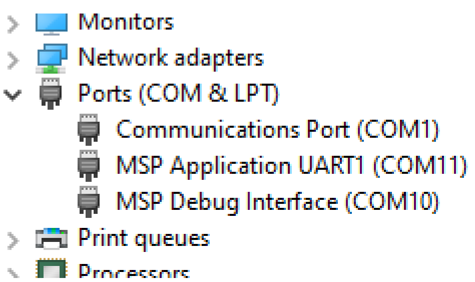

5. Open PuTTY and under ‘Connection type’, select ‘Serial’. This is under the ‘Session’ menu.
6. Change ‘Serial line’ to the COM port that you took note of in step 4.

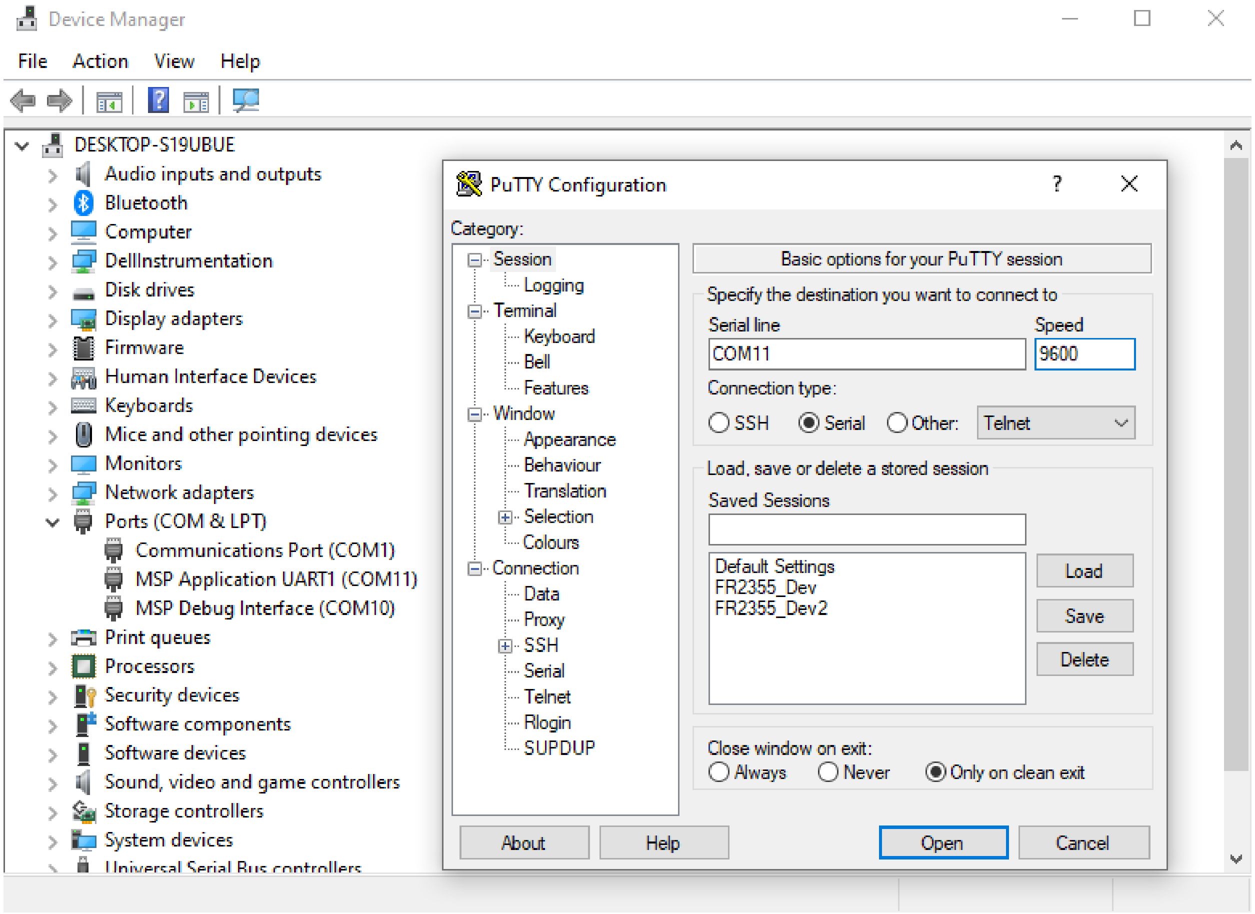

7. Navigate to ‘Connection > Serial’ and change ‘Flow Control’ to ‘None’.

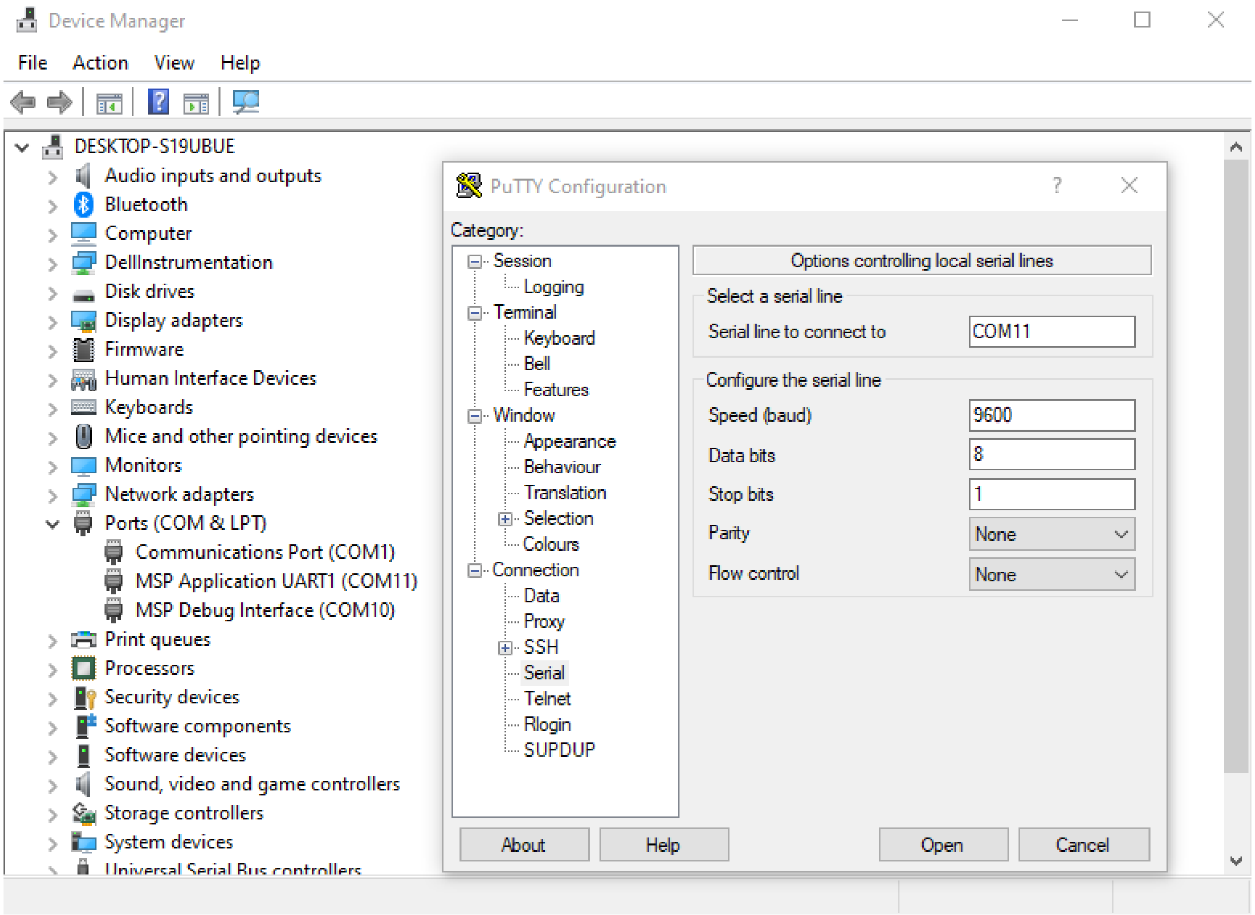

8. Finally, navigate back to the ‘Session’ menu and under ‘Load, save or delete a stored session > Saved Sessions’, give this preset settings profile a name then click ‘Save’. You will be referencing this profile name often.

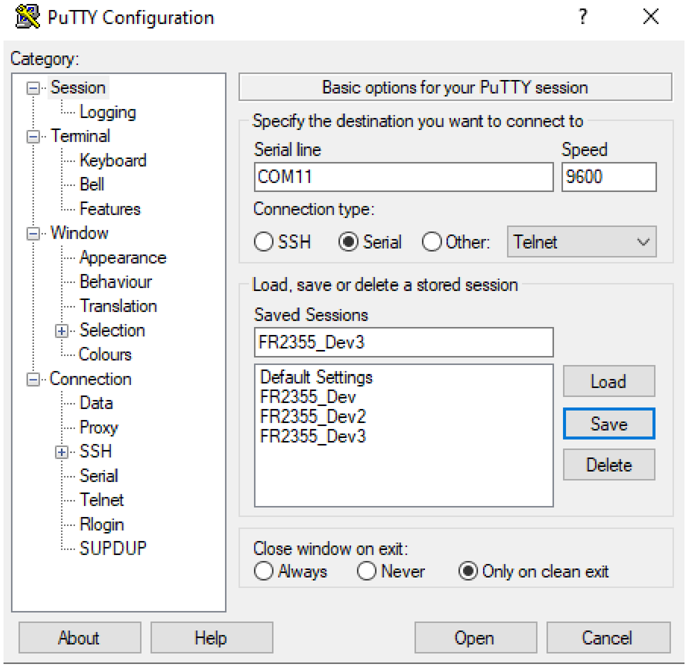

9. Click ‘Open’ and a black command window should appear. Try sending a capital ‘R’ (‘Reset’) to see if the device responds.

Opening PuTTY and connecting to an arena microcontroller

1. Turn on the microcontrollers. Do so by connecting them to the PC via USB. In our case at the Friedman Lab, do so by pressing the rightmost button on the USB hub located to the left of the computer tower in the recording room. The USB hub will light up with blue light indicating that it is on.

2. Open PuTTY. You will likely find this program in a folder named “PuTTY (64-bit)” under the Windows Start menu. Otherwise, you may search for it by typing “putty” in the Windows search bar.

3. Once open, PuTTY will load a default session preset. Near the bottom you will find a white space containing other session presets, and at the very bottom of the windows, you’ll find an “Open” button. DO NOT click the “Open” button yet!

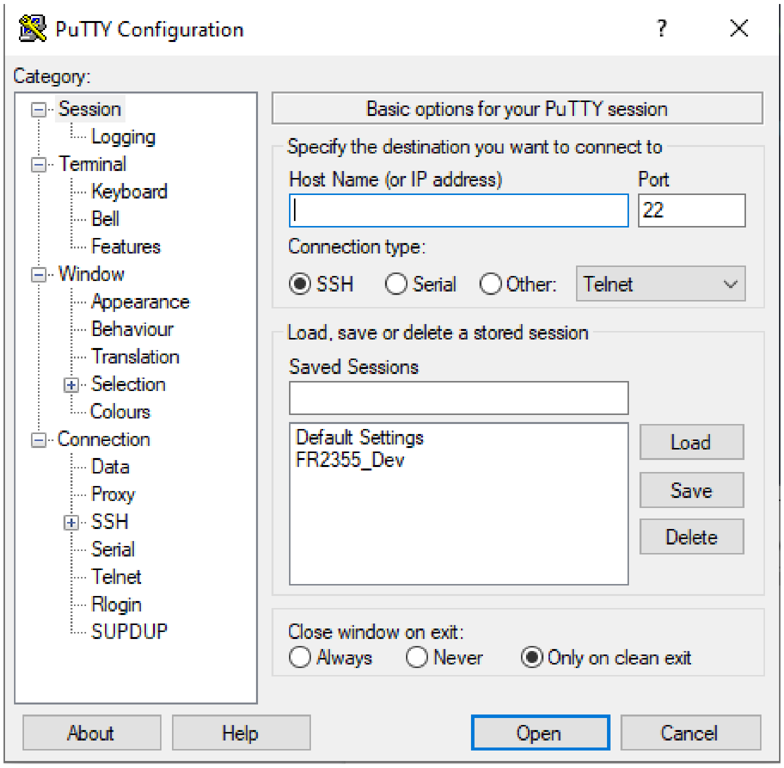

4. Each preset will configure PuTTY to work with its corresponding arena. Double click the preset that corresponds to the arena that you are calibrating, this will open a communication channel to that arena’s microcontroller.

   a. FR2355 - Arena 1
   b. FR2355_2 - Arena 2
   c. FR2355_3 - Arena 3
   d. FR2355_4 - Arena 4

5. A small black window with a green cursor and no text will appear. This is where commands are sent to the microcontroller. With the window in focus, type the letter ‘R’ (capitalised). The microcontroller will respond by repeating what you sent, along with a message, which will change depending on what you send. ‘R’ is a command that resets the microcontroller to its idle state, turning all LEDs off. The black PuTTY window should now display the message “all off”; if no message is displayed, make sure the microcontroller is powered and connected to the PC, then repeat steps 2 through 4 and try again. **Contact a lab supervisor or the PI if there is still no response, DO NOT tamper with the microcontroller on your own.**

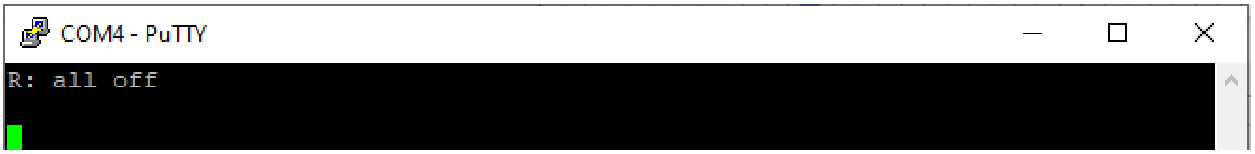

6. You’ve successfully connected and are now communicating with the microcontroller! Continue to the next section to calibrate or configure LED brightness.

Using Plink to communicate with RECORD

RECORD can be controlled via command prompt if Plink, PuTTY’s command line executable, is installed. Once a PuTTY session is created following the steps in the previous two sections, one may opt to interfacing with the system by opening a command line window and typing

`plink -load [session name]`,

After which the command line will behave just the same as any PuTTY command line.

Using RECORD-lib to communicate with RECORD

RECORD-lib is a Python (https://www.python.org/) library custom made for communications with the RECORD system. It uses the ‘pyserial’ library as a basis and includes various functions to do things like set a cost light up, deliver a reward, etc. This along with the ‘Trials’ class was used to create a Python script that can be run through a command line interface or through an IDE like Spyder (https://www.spyder-ide.org/) which runs through our high- and low-cost cost/benefit tasks, as well as our association task through tweaking of a few parameters. You can find RECORD-lib in our Github repository (https://github.com/rjibanezalcala/RECORD/tree/main/python/RECORD-lib) along with our sample trial (https://github.com/rjibanezalcala/RECORD/blob/main/python/RECORD-lib/sample_trial_with_output_v0.3.5.py). Here we’ll describe what each parameter is.

Currently, the following trial parameters must be modified in the code itself, so a graphical IDE is recommended when running trials. After all parameters have been set, click the ‘Run’ button or press ‘F5’ to run the script.

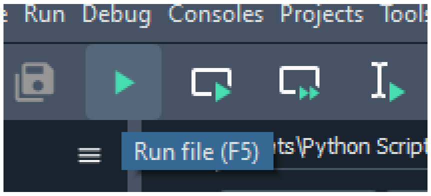

Make sure to set the 'mcu_com_port' parameter to the COM port the UART interface of the microcontroller is in. See steps 1 through 4 under 'Setting PuTTY up for the first time' for more details.

```
mcu_com_port = "COM4"
TTL_ON       = False
```

```
# BE SURE TO NOT REMOVE QUOTATION MARKS WHEREVER YOU MAKE CHANGES!!
trialParams = {
               # Time intervals, in seconds.
               'inter_trial_interval': 1,
               'decision_interval'   : 1,
               'feeding_interval'    : 1,
               # Number of trials
               'trials'              : 5,
               # Do not need to be changed
               'levels'              : [0, 1, 2, 3],
               'feeders'             : ['a', 'b', 'c', 'd'],
               # Probability of a COST level appearing in the trial list. Sum of
               'lvl_probs'           : [0, 1, 0, 0],
               # Probability of a FEEDER appearing in the trial list. Sum of all
               'fdr_probs'           : [0.25, 0.25, 0.25, 0.25],
               # Sugar concentration in reward solution. If compound solution (fo
               'rewards'             : ["9%","5%","2%","0.5%"],
               # Reward volume delivered
               'reward_volume'       : "4ml",
               # Cost intensity, in lux. Include units, no spaces.
               'intensities'         : ["0lux","15lux","140lux","290lux"],
               # Subject's name, as defined in rat roster.
               'subj_name'           : "A4",
               # Specify the type of manipulation done. If regular behaviour tria
               'subj_health'         : "Normal",
               # Specify the subject weight, in grams. Include units, no spaces.
               'subj_weight'         : "Undefined",
               # Specify the type of task done. For example L1, L2, L3, L1L2, L1L
               'task_type'           : "L1",
               # The root folder where all generated data is going to be saved to
               'root_folder'         : "D:/BehaviourData/",
               # Timezone information for timestamping
               'timezone'            : "US/Mountain",
               # Physiological recording made/Technique used. For example: Calciu
               'recording_type'      : "Calcium Imaging"
               ## End
               }
#
##############################################################################
```

Parameter	Description	Example
inter_trial_interval	The time delay in-between trials.	*5*
decision_interval	The time delay between when an offer is presented and when it is decided if a subject approached the offer.	*3*
feeding_interval	The time delay to allow the subject to consume the reward.	*5*
trials	The number of trials in a session	*40*
levels	A list of the available cost levels in the trials, from lowest cost level at element 0, to the highest cost level at element N-1 (typically N= 4).	*[0, 1, 2, 3]*
feeders	A list of the available feeders representing reward levels in the trials, from the first feeder with the highest reward level at element 0, to the last feeder with the lowest reward level at element N-1 (typically N= 4).	*[1, 2, 3, 4]*
lvl_probs	Probability of a COST level appearing in the trial list.<br><br>Cost level 0 is represented in element 0, and the highest cost level is represented in element N-1 (typically N= 4).<br><br>Sum of all elements must equal 1.	*[0, 0.5, 0, 0.5]*<br><br>*(represents an L1 L3 trial)*
fdr_probs	Probability of a FEEDER appearing in the trial list.<br><br>Feeder 1 is represented in element 0, and the last feeder is represented in element N-1 (typically N= 4).	*[0.25, 0.25, 0.25, 0.25]*

	Sum of all elements must equal 1.	
rewards	Sugar concentration in each reward solution. If compound solution (for example sucrose + alcohol), include second solute concentration in parenthesis: X%(Y%). No spaces.<br><br>Serves as a label for the data.	*["9%","5%","2%","0.5%"]*
reward_volume	Reward volume delivered.<br><br>Does not affect the reward volume delivered by RECORD, serves only as a data label.	*"4ml"*
intensities	Cost intensity, in lux. Including units, no spaces.<br><br>Does not affect the LED brightness delivered by RECORD, serves only as a data label.	*["0lux","15lux","140lux","290lux"]*
subj_name	Subject's name or ID number, as defined in rat roster.	*"Princess"*
subj_health	Specifies the type of manipulation done. If regular behaviour trial: "Normal"; if alcohol trial: "Alcohol"; if oxy trial: "Oxycodone"; etc...	*"Normal"*
subj_weight	Specify the subject weight, in grams. Include units, no spaces. If no weight is defined, write "Undefined"	*"200 g"*
task_type	Specify the type of task done. For example L1, L2, L3, L1L2, L1L3, etc.	*"L1L3"*
root_folder	The root folder where all generated data is going to be saved to. Can be an absolute or relative path.	*"D:/BehaviourData/"*

timezone	Timezone information for timestamp data. If not defined, defaults to UTC. We use the ‘pytz’ library to generate timezone data on our ‘datetime’ timestamps.	*“US/Mountain”*
recording_type	Physiological recording made/Technique used. For example: Calcium Imaging, Optogenetics, Electrophysiology, etc.	*“Calcium Imaging”*

RECORD System Operation

The RECORD system consists of the arenas and its individual components, but of equal importance, the microcontroller unit and the various electronic components that surround it. This section will use PuTTY to demonstrate the operation of the system, but the RECORD system can also be operated via batch script or via Python script using the RECORD-lib package (https://github.com/rjibanezalcala/RECORD/tree/main/python/RECORD-lib).

Available Commands

Command	Description
#	Turns on a specific feeder LED ring at a specified level. The microcontroller will quietly wait for an input and only execute it once four characters have been entered. The second and fourth character dictate the feeder that will be activated and the cost level at which the light will be turned on. Generally, it is recommended that inputs are formatted in the following way: FxLy, where X is the feeder, and Y is the cost level.
F, G, H, and J	Toggles relay 1, 2, 3, and 4, respectively, which will open and close their respective valves. The amount of time that the relay will be closed can be configured in configuration mode.
R	Resets everything but the TTL signals. All LEDs are turned off and relays are opened.
$	Starts configuration mode. Instructions will pop up as soon as you input this command. This will allow you to reconfigure how bright feeder LEDs should be, how long valves stay open, how long TTL pulses are, amongst other settings. *
%	Starts calibration mode. This allows the user to increase and decrease the brightness of the cost LEDs on the fly. It is recommended this command is only used to calibrate the LED brightness of each cost level and not during a trial. *
T	Sends a TTL out through port 3.6. *
K	Toggles the 'trial in progress' light.
Q	Starts the internal timer. This timer counts seconds and milliseconds. Starting the timer will increment the timestamps that show in the acknowledgement message for each command.

W	Gets the current time from the internal timer. Time will be formatted as *[seconds].[milliseconds]*.
E	Stops the internal timer. This will reset the timer back to 0.
Y	Allows the system to respond to external TTLs received through P3.5.
?	Shows system information and a list of available commands with a description of each. *

* All commands (with the exception of 'T', ‘$’, ‘%’, and ‘?’) will be acknowledged with the ACK signal through port 3.0 upon execution

The internal timer

An internal timer can be used to keep track of events that happen throughout a trial. Here, each event refers to each time an event is sent to and acknowledged by the microcontroller. In each acknowledgement message, a timestamp will be reported from the internal timer; if the timer has not been started, the timestamp reported will read “0.0”.

Requesting system information

When requesting system information via the ‘?’ command, relevant information will be displayed first, then if a command list is needed, the user can then send an ‘H’ character. Entering any other character (including a return character) will exit the command.

The microcontroller buttons

The onboard microcontroller buttons serve only as a test to determine if the microcontroller is receiving commands. They currently (firmware v2.2.0) serve no other purpose.

Configuration mode

This mode's main purpose is to set the system up with different operating conditions for a customisable setup, when changes need to be made on the fly. However, it is important to note that any changes made in configuration mode will be lost as soon as the microcontroller is power cycled, as it will boot up with the default parameters set up by the "*_cfg.h*" file in the microcontroller firmware (https://github.com/rjibanezalcala/RECORD/blob/main/microcontroller/mcucfg_dev1.h). To make changes to the default configuration of the device, the microcontroller must be re-flashed with the desired changes.

When no other commands are active, configuration mode is accessed via the '$' command. It is recommended that this mode be accessed through a command line interface such as PuTTY or Plink, but configuration can be scripted through any scripting language, or through RECORD-lib, our python library (https://github.com/rjibanezalcala/RECORD/tree/main/python/RECORD-lib).

Configuring LED brightness

Configuring the brightness at which the LEDs turn on at each cost level is done by selecting option 'A' after entering configuration mode. The microcontroller will then prompt the user for some input, namely…

1. What feeder needs to be configured,

2. What cost level is to be configured,

3. And the new **CCR value** (see more on CCR values under the "How LED brightness is controlled" section).

Configuring relay active time

How long the relays are held open when using the F, G, H, or J commands is option 'B' after entering configuration mode. How long this is must be expressed in milliseconds as a whole, positive integer number, without exceeding 9000 milliseconds.

Configuring TTL length

Similar to the relay active time, the TTL length parameter cannot exceed 9000 milliseconds and must be expressed in milliseconds as a whole, positive integer number. This parameter is configured in menu item 'C' after entering configuration mode and affects both the user-prompted outgoing TTL ('T' command) and the ACK TTL, which is sent every time a command is executed.

Configuring outgoing TTL operating modes

The user-prompted outgoing TTL (‘T’ command) can be configured to operate in two different operating modes, and can also be turned off entirely. Selecting menu item ‘D’ after entering configuration mode will give the user the option to select which operating mode to set.

This TTL is ‘low’ (logical 0) by default, and will only be set to ‘high’ (logical 1) when the ‘T’ command is received, with how it is set varying between operation modes. When high, a non-zero voltage will be observed on the output pin; when ‘low’, a negligible voltage will appear at the output pin.

Below is a short description of each operation mode…

- *Toggle* is where a TTL is set to high and held at high until the ‘T’ command is called again.
- *Pulse* is where the TTL is set to high and held only for the amount of time set by the TTL length parameter. When TTL length is elapsed, the TTL will be set back to low.
- *Off* will simply turn outgoing TTL servicing off, meaning that if the ‘T’ command is received, the microcontroller will only display a message saying that a TTL was requested, but no TTL will be sent.

The outgoing TTL will never be accompanied by an ACK signal, regardless of what operation mode it is operating in.

Incoming TTL servicing

Servicing incoming TTLs may be useful when synchronising the RECORD system with another external system. By default, external TTL servicing is turned off, but can be turned on via the ‘Y’ command. Upon receiving an external TTL, the Port_3_ISR(void) interrupt service routine (ISR) is called. In version 2.2.0, this ISR does not contain any useful action and must be configured by the user by modifying the firmware; however, actual configuration of the action to be taken when receiving a TTL is a future feature that is under development.

Calibration and Reconfiguration of Cost Levels for the RECORD System

This guide focuses on the reconfiguration and calibration of LED brightnesses for each feeder at all 3 cost levels. Our RECORD system is able to deliver 3 different levels of cost for our decision-making tasks. This is done through pulse width modulation using crystal oscillator-driven timers on a Texas Instruments MSP430 FR2355 microcontroller.

Fine-tuned calibration of cost levels in calibration mode

This section covers calibration mode. In this mode, you are able to increase and decrease the **CCR value** to match a desired **Lux value** without the need of writing a new value each time. Refer to the LED Calibration document for information on what Lux you should aim for. You will need a **lightmeter** on hand to read Lux. *DO NOT reconfigure cost levels unless directed and supervised by a lab supervisor. Misconfigurations can and will affect our data!!*

1. Follow the steps in the previous section to open a communication channel to the microcontroller.
2. Send a '%' (percent symbol) to enter calibration mode. The microcontroller will respond with instructions on how to proceed.

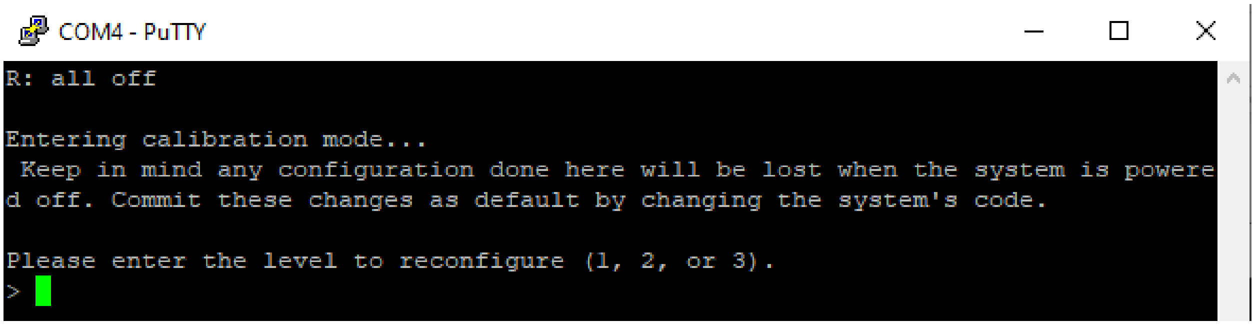

3. At this point, the microcontroller will ask you for some information, interact with it by using your keyboard. Inputs will automatically be sent without the need to press "Enter" in between inputs. Only one feeder can be calibrated at a specific level at a time.
    a. The first prompt will ask you to specify at which **level** you wish to calibrate a feeder (L1, L2, or L3, see the "What are cost levels?" section for more information). **Enter either 1, 2, or 3 only**, do not enter letters, a number greater than 3, or less than 1, doing so will result in an error message at a later step.
    b. The second prompt will ask you what **feeder** you wish to calibrate at the specified level. **Enter a *whole* number between 1 and 4**, corresponding to each feeder on the arena. Similar to before, do not enter letters, numbers over 4, or numbers below 1:
        i. Entering a 1 will reference the feeder at the **diagonal zone**.
        ii. Entering a 2 will reference the feeder at the **grid zone**.
        iii. Entering a 3 will reference the feeder at the **horizontal zone**.
        iv. Entering a 4 will reference the feeder at the **radial zone**.

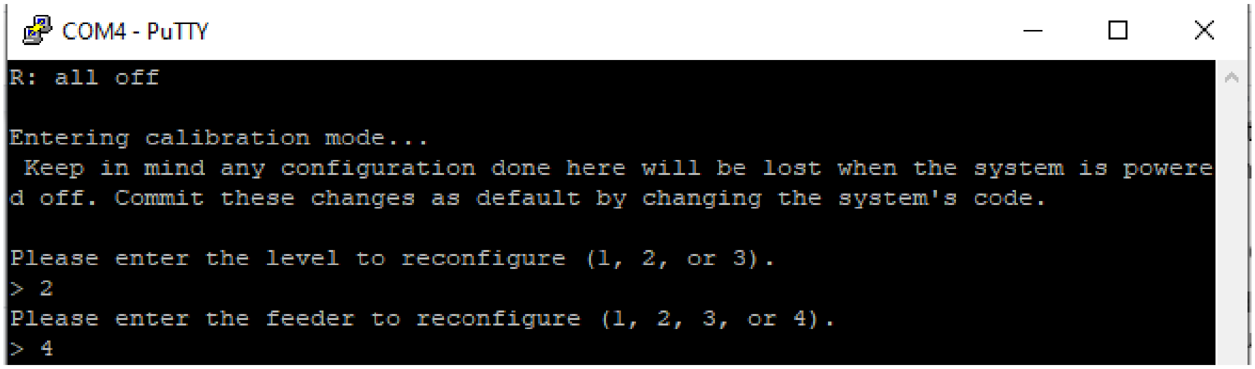

If an invalid input is entered, an error message will appear and calibration will be aborted. The previously saved parameters will be used until successful reconfiguration.

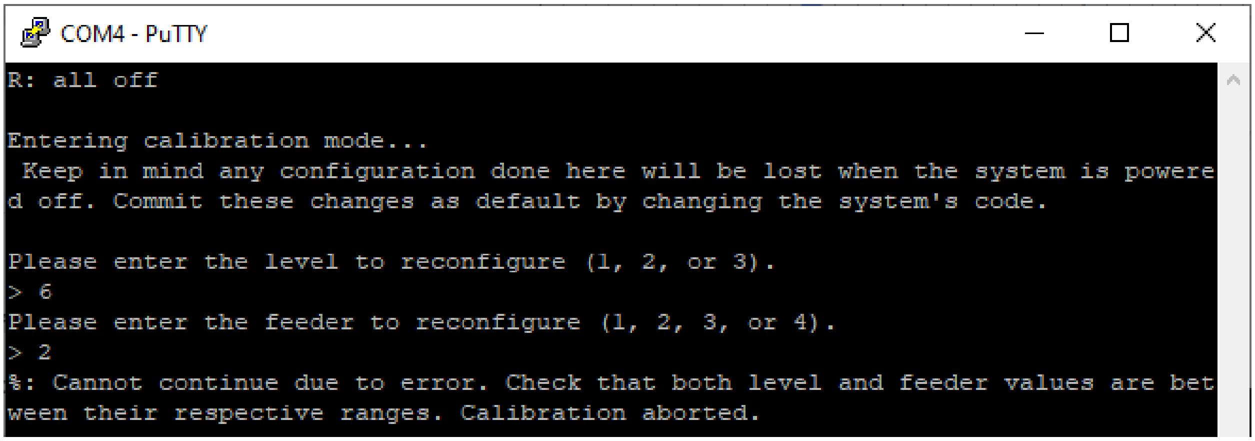

4. At this point, the feeder you selected should have turned on at the level you're calibrating. The microcontroller will tell you what **CCR value** is currently being used for that level, this will be displayed in PuTTY.

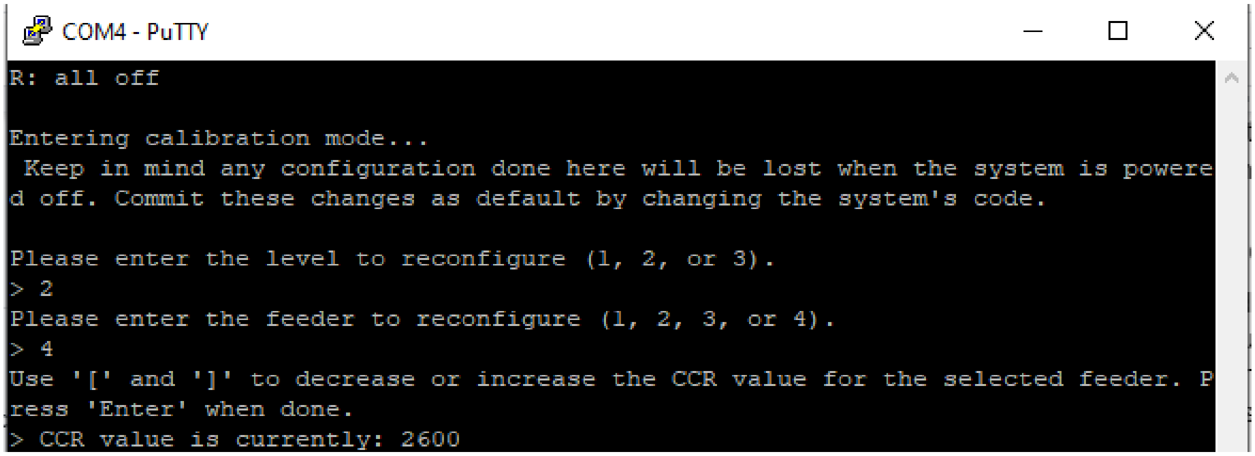

To increase the brightness on that feeder, one must decrease the **CCR value**, to do this, use the closing square bracket (']') To decrease the brightness by lowering the **CCR value** use the opening square bracket ('['). As before, the **CCR value** will update as you send either symbol, and will be displayed on the PuTTY window.

COM4 - PuTTY

```
R: all off

Entering calibration mode...
 Keep in mind any configuration done here will be lost when the system is powere
d off. Commit these changes as default by changing the system's code.

Please enter the level to reconfigure (1, 2, or 3).
> 2
Please enter the feeder to reconfigure (1, 2, 3, or 4).
> 4
Use '[' and ']' to decrease or increase the CCR value for the selected feeder. P
ress 'Enter' when done.
> CCR value is currently: 2600
> CCR value is currently: 2550
> CCR value is currently: 2500
> CCR value is currently: 2550
> CCR value is currently: 2600
> CCR value is currently: 2650
> CCR value is currently: 2700
```

Measure the Lux at that feeder using the **lightmeter** and check that the measured value falls within 10 Lux of the target Lux value. **Make sure to write this value down along with the CCR value that produced it!**

5. Once you are satisfied and have written down both the **CCR value** and the corresponding **Lux value**, hit the “Enter” key. The microcontroller will ask you if you wish to save this new **CCR value**. Enter ‘y’ for **yes**, and ‘n’ for **no**.

   If you enter ‘y’, the **CCR value** will be updated and used until the microcontroller is turned off and a message confirming reconfiguration will be displayed and all feeders will be turned on at the level you just calibrated. Only the feeder you were calibrating will be changed, you may be able to tell a difference in brightness.

```
> CCR value is currently: 2750
> CCR value is currently: 2700
> CCR value is currently: 2650
> CCR value is currently: 2600
Would you like to apply these changes? [y/n]
> y
%: New settings applied! Resuming previous operations...
```

   If you enter ‘n’, all changes from this calibration session will be discarded and the microcontroller will revert back to the last saved **CCR value**. All feeders will turn on.

```
> CCR value is currently: 2850
> CCR value is currently: 2900
> CCR value is currently: 2950
> CCR value is currently: 3000
Would you like to apply these changes? [y/n]
> n
%: Rolled back to previous configuration. Resuming previous operations...
```

6. After the final message is sent, the microcontroller will go back to its idle state and will be able to receive more commands.

Please only calibrate the LEDs under supervision of the PI or a lab supervisor. Small differences in LED brightnesses can affect our data!!

Reconfiguration of cost levels in configuration mode

This section covers configuration mode. In this mode, you are able to change the **CCR value** directly to match a desired **Lux value**. Refer to the LED Calibration document for information on what Lux you should use. You will need a **lightmeter** on hand to read Lux. *DO NOT reconfigure cost levels unless directed and supervised by a lab supervisor. Misconfigurations can and will affect our data!!*

1. Follow the steps in the opening PuTTY section to open a communication channel to the microcontroller.
2. Send a '$' (dollar sign) to enter configuration mode. The microcontroller will respond with instructions on how to proceed.

```
Entering configuration mode...
 Keep in mind any configuration done here will be lost when the system is powere
d off. Commit these changes as default by changing the system's code.

Please enter the level to reconfigure (1, 2, or 3).
>
```

3. At this point, the microcontroller will ask you for some information, interact with it by using your keyboard. Inputs will automatically be sent without the need to press "Enter" in between inputs. Only one feeder can be calibrated at a specific level at a time.
    a. The first prompt will ask you to specify at which **level** you wish to calibrate a feeder (L1, L2, or L3, see the "What are cost levels?" section for more information). **Enter either 1, 2, or 3 only**, do not enter letters, a number greater than 3, or less than 1, doing so will result in an error message at a later step.
    b. The second prompt will ask you what **feeder** you wish to calibrate at the specified level. **Enter a *whole* number between 1 and 4**, corresponding to each feeder on the arena. Similar to before, do not enter letters, numbers over 4, or numbers below 1:
        i. Entering a 1 will reference the feeder at the **diagonal zone**.
        ii. Entering a 2 will reference the feeder at the **grid zone**.
        iii. Entering a 3 will reference the feeder at the **horizontal zone**.
        iv. Entering a 4 will reference the feeder at the **radial zone**.

```
Entering configuration mode...
 Keep in mind any configuration done here will be lost when the system is powere
d off. Commit these changes as default by changing the system's code.

Please enter the level to reconfigure (1, 2, or 3).
> 3
Please enter the feeder to reconfigure (1, 2, 3, or 4).
> 1
Please enter the new integer CCR value for this level and feeder (0 through 8000
, whole numbers only).
Enter a 4-character number or press enter if you're entering less than 4 charact
ers.
>
```

4. The microcontroller will ask for a new **CCR value** for the level and feeder you are reconfiguring. Enter a *whole* number between 0 and 8000 *only*. If your new **CCR value** consists of three digits or less, press the “Enter” key to submit the new value.

   Please refer to the LED Calibration spreadsheet for information about what values result in what Lux. Only use values contained in this spreadsheet unless you are calibrating a feeder!

```
Entering configuration mode...
 Keep in mind any configuration done here will be lost when the system is powere
d off. Commit these changes as default by changing the system's code.

Please enter the level to reconfigure (1, 2, or 3).
> 3
Please enter the feeder to reconfigure (1, 2, 3, or 4).
> 1
Please enter the new integer CCR value for this level and feeder (0 through 8000
, whole numbers only).
Enter a 4-character number or press enter if you're entering less than 4 charact
ers.
> 400
```

5. After entering four digits or pressing the “Enter” key, the microcontroller will ask you if you would like to preview the new brightness. Enter ‘y’ for **yes**, and ‘n’ for **no**.

```
Please enter the new integer CCR value for this level and feeder (0 through 8000
, whole numbers only).
Enter a 4-character number or press enter if you're entering less than 4 charact
ers.
> 400
Would you like to test the new value? [y/n]
>
```

   If you enter ‘y’, all feeders will turn on at the level you have just reconfigured, however only the feeder you specified in step 3 will be affected. Changes will be applied and saved for the rest of the session (until the microcontroller is turned off).

```
Enter a 4-character number or press enter if you're entering less than 4 charact
ers.
> 400
Would you like to test the new value? [y/n]
> y
$: New settings applied! Resuming previous operations...
```

If you enter an ‘n’, the new **CCR value** will still be applied, but the feeder will need to be turned off and then back on again for the changes to become evident.

```
Enter a 4-character number or press enter if you're entering less than 4 charact
ers.
> 400
Would you like to test the new value? [y/n]
> n
$: Configuration applied! Restart feeders to see changes. Resuming previous oper
ations...
```

If an invalid input is entered, an error message after the new CCR value is entered will appear and calibration will be aborted. The previously saved parameters will be used until successful reconfiguration.

```
Entering configuration mode...
 Keep in mind any configuration done here will be lost when the system is powere
d off. Commit these changes as default by changing the system's code.

Please enter the level to reconfigure (1, 2, or 3).
> 5
Please enter the feeder to reconfigure (1, 2, 3, or 4).
> 8
Please enter the new integer CCR value for this level and feeder (0 through 8000
, whole numbers only).
Enter a 4-character number or press enter if you're entering less than 4 charact
ers.
> 777
Error: New CCR value should not exceed 8000 or LEVEL should not exceed 3. Config
uration not set.
$: Configuration aborted due to error, try again. Resuming previous operations..
```

6. Measure the brightness of the feeder using a **lightmeter**, then record the new **CCR value** and its corresponding **Lux value**. After the final message is sent, the microcontroller will go back to its idle state and will be able to receive more commands.

Please only calibrate the LEDs under supervision of the PI or a lab supervisor. Small differences in LED brightnesses can affect our data!!

Suggested procedure to calibrate LEDs

Relevant documentation

Refer to the following documentation when calibrating the RECORD system feeder LEDs:

1. LED Calibration Data:
    a. Arena 1 Calibration (Sheet)
    b. Arena 2 Calibration (Sheet)
    c. Arena 3 Calibration (Sheet)
    d. Arena 4 Calibration (Sheet)
    e. Per-Rat Lux Requirements (Sheet)
2. Reconfiguration of cost levels in configuration mode
3. Fine-tuned calibration of cost levels in calibration mode

Note: DO NOT edit these documents unless otherwise instructed to by the PI or a lab supervisor!

Creating LED lines of best fit to aid in calibration

The “**calibration lines**” or “**trend lines**”, help us estimate a CCR value that may possibly produce the appropriate amount of Lux we are looking for. These are calculated from “**calibration curves**”, which we create by measuring Lux at 6 different CCR values. The **calibration line** is the trend these curves follow, also known as the “line of best fit” or “regression line”, which results from a statistical calculation called the “Least Square Method”. Our LED Calibration Data document does this automatically, all that needs to be done is enter the 6 measurements mentioned before. The following steps will guide you on how to do this, *you will need a **lightmeter** able to measure up to 1000 Lux to proceed*.

1. Open the LED Calibration Data document and open one of the Arena Calibration sheets. You will be met with a small table with numbers, underneath it will be a large graph with four coloured lines, these are the **calibration curves**.
2. Check with a lab supervisor or the PI to know if the data is accurate; the date of last measurement is located to the right of the first table on cell H2, the data may or may not need to be updated, depending on changes made to the arenas. If an update is needed, proceed to step 3.

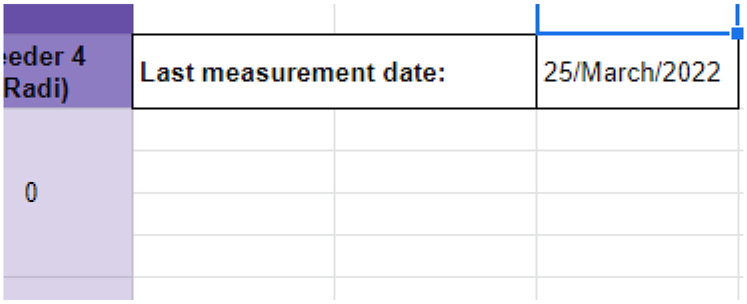

3. Open PuTTY and connect to the microcontroller for the arena you are calibrating, see the "Opening PuTTY and connecting to an arena microcontroller" section on this guide for instructions on how to do so.
4. Using the steps in the "Reconfiguration of cost levels in configuration mode" to enter **configuration mode** on the microcontroller, start by configuring the microcontroller to turn all the feeders on to a CCR value of either 6400, 4800, 3200, 1600, or 1. You may choose any level for this calibration when prompted.
5. Turn on the **lightmeter** and make sure to set it to measure Lux, not Fc or Lumen. Then, set the measurement range to 0 - 2000 Lux by pressing the "R" button until the range indicator on the bottom on the **lightmeter** screen underlines the 2000 Lux range, you may be able to measure using the lowest range if you are calibrating cost level 1, but if the **lightmeter** reads "OL" at any point, you will need to step up a range. Finally, because the measured value will constantly be changing, it helps to take only the maximum value. Point the **light sensor** (it looks like a white half-sphere) and set the **lightmeter** to "Max" mode then at a dark spot on the arena, this will freeze the measured value.
6. Start by measuring feeder 1 on the horizontal corner of the arena. Take the **light sensor** and hold it about 3 millimetres or less above each one of the four LEDs on the feeder, holding it in place for 2 to 3 seconds. The lightmeter will display the maximum value it was able to measure. It is recommended you measure each feeder at least twice, as there will be a wide variability in the measurement.
7. Record the values you measured for each feeder into the appropriate row for the CCR value you're measuring, you'll start to see the calibration curve graph change.
8. Once you are done with that CCR value, move on to the next and repeat steps 4 through 7 until you've measured all feeders at each CCR value.
9. The **trend line equation** will appear on the calibration curve graph in the legend on the right side.

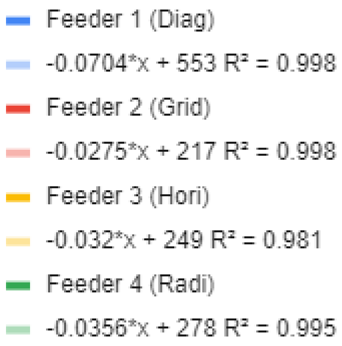

X in this equation is the CCR value, and Y is the Lux. R^2 is the correlation coefficient, the higher this number is, the better the line fits the data, you do not need to worry about this value for now. Let's generalise this equation to $\mathbf{Y = A_{fdr} * X + B_{fdr}}$, where $\mathbf{A_{fdr}}$ and $\mathbf{B_{fdr}}$ are the corresponding slope and Y-intercept values for each feeder's **trend line**. This is the first and last bit of maths you'll need to know for this guide.

10. Solve the equations for X. Below is an example using the first equation marked in muted blue:

$$Y = -0.0704\ X + 553$$

$$Y - 553 = -0.0704 X + 553 - 553$$
$$Y - 553 / (-0.0704) = (-0.0704 X) / -0.0704$$
$$\mathbf{Y - 553 / (-0.0704) = X}$$

Set up this equation in terms of spreadsheet cells for the **CCR value estimator**:

a. For feeder 1, enter “=ROUND((R33-$\mathbf{B_{fdr}}$)/$\mathbf{A_{fdr}}$)” into cell R34
b. For feeder 2, enter “=ROUND((R36-$\mathbf{B_{fdr}}$)/$\mathbf{A_{fdr}}$)” into cell R37
c. For feeder 3, enter “=ROUND((R39-$\mathbf{B_{fdr}}$)/$\mathbf{A_{fdr}}$)” into cell R40
d. For feeder 4, enter “=ROUND((R42-$\mathbf{B_{fdr}}$)/$\mathbf{A_{fdr}}$)” into cell R43

11. Once done setting up the **CCR value estimator**, you are done. Notify a lab member to double check your work!

Calibrating feeder LEDs to rat-specific required Lux values

It is important to know where to start with the calibration process. We have set up a chart with **target lux values**, individualised for each rat in the Per-Rat Lux Requirements sheet on the LED Calibration Data documentation. This document tells you what Lux value to aim for at each cost level when calibrating the RECORD system; but how do you know what CCR value will give you these values?

In the same document, there are four sheets that describe the LED brightness behaviour at each arena, we call these “**calibration lines**” or “**trend lines**”. These graphs are carefully created by our team to predict a ballpark estimate of the CCR value needed for the desired Lux values. The sheets also contain a history of CCR values that were found to produce a Lux value within the desired ranges, in case we need to dial back or crank up the brightness without putting in more effort than needed. See the “Creating LED calibration lines to aid in calibration” section in this guide to learn how these calibration lines are obtained.

Follow the following steps to begin calibrating, *you will need a **lightmeter** able to measure up to 1000 Lux to proceed*:

1. Open the Per-Rat Lux Requirements sheet and refer to columns E, F, and G to find out what Lux value you are to aim for. Choose a level, then choose a Lux value to begin with, this will be your **target Lux value**.

A	B	C	D	E	F	G	H	I
Index	Rat Name	Sex	ID No.	Level 1 Target Lux	Level 2 Target Lux	Level 3 Target Lux		
1	Alexis	F	14290.1	15.0 Lux	40.0 Lux	240.0 Lux	Last Updated:	08/April/2022
2	Sarah	F	14290.2	15.0 Lux	40.0 Lux	240.0 Lux		
3	Kryssia	F	14291.1	15.0 Lux	40.0 Lux	240.0 Lux	Level Probability of Appearance (L1/L2/L3):	(70 / 0 / 30)%
4	Raven	F	14291.2	15.0 Lux	40.0 Lux	240.0 Lux		
5	Harley Quinn	F	14292.1	15.0 Lux	40.0 Lux	240.0 Lux		
6	Shakira	F	14292.2	15.0 Lux	40.0 Lux	240.0 Lux		
7	Raissa	F	14293.1	15.0 Lux	40.0 Lux	240.0 Lux		
8	Renata	F	14293.2	15.0 Lux	40.0 Lux	240.0 Lux		
9	Andrea	F	14294.1	15.0 Lux	40.0 Lux	240.0 Lux		
10	Neftali	F	14294.2	15.0 Lux	40.0 Lux	240.0 Lux		
11	Fiona	F	14295.1	15.0 Lux	40.0 Lux	240.0 Lux		
12	Juana	F	14295.2	15.0 Lux	40.0 Lux	240.0 Lux		
13	Sully	M	14296.1	15.0 Lux	40.0 Lux	180.0 Lux		
14	Mike	M	14296.2	15.0 Lux	40.0 Lux	180.0 Lux		
15	Jafar	M	14297.1	15.0 Lux	40.0 Lux	180.0 Lux		
16	Aladdin	M	14297.2	15.0 Lux	40.0 Lux	180.0 Lux		
17	Kobe	M	14298.1	15.0 Lux	40.0 Lux	180.0 Lux		
18	MJ	M	14298.2	15.0 Lux	40.0 Lux	180.0 Lux		
19	Junior	M	14299.1	15.0 Lux	40.0 Lux	180.0 Lux		
20	Carl	M	14299.2	15.0 Lux	40.0 Lux	180.0 Lux		
21	Scar	M	14300.1	15.0 Lux	40.0 Lux	180.0 Lux		
22	Simba	M	14300.2	15.0 Lux	40.0 Lux	180.0 Lux		
23	Jimi	M	14301.1	15.0 Lux	40.0 Lux	180.0 Lux		
24	Johnny	M	14301.2	15.0 Lux	40.0 Lux	180.0 Lux		

2. Choose an arena to calibrate and check with a lab supervisor that the calibration lines are accurate to the feeders currently present in the arena. If the calibration lines are inaccurate, move on to a different arena.
3. Enter your **target Lux value** in the “Desired Lux” fields for each of the four feeders on the **CCR value predictor** (cells R33, R36, R39, and R42). The predictor will calculate a rough estimate of a CCR value you can start with.

Feeder 1 (Diagonal, Blue)		Level 1	Level 2	Level 3	Level 3	Level 3
Desired Lux =	180	15		142		239
Calculated CCR =	5298	7742		5925		4600
Feeder 2 (Grid, Red)						
Desired Lux =	240	16		142		241
Calculated CCR =	-836	7800		5950		4100
Feeder 3 (Horizontal, Yellow)						
Desired Lux =	240	15		137		240
Calculated CCR =	281	7700		5750		3281
Feeder 4 (Radial, Green)						
Desired Lux =	240	14		141		243
Calculated CCR =	1067	7388		3876		868
	Target Lux:	15	40	140	180	240
	Calibration last updated on:	02/April/2022				

Note: If the calculated CCR value happens to be negative, continue with a value of 0. If it is over 8000, proceed with a value of 8000. Do not use any values lower than 0, or above 8000, as this may result in unexpected microcontroller behaviour.

4. Open PuTTY and connect to the microcontroller for the arena you are calibrating, see the “Opening PuTTY and connecting to an arena microcontroller” section on this guide for instructions on how to do so.
5. With the **estimated CCR values** you got, enter **configuration mode** on the microcontroller by sending a dollar sign (‘$’) to the microcontroller.
6. Follow the steps in the “Reconfiguration of cost levels in configuration mode” to enter the **estimated CCR values** to the microcontroller. You may or may not be able to see the lights change in brightness. Don’t worry if you can’t, we’re making very small changes to the brightness here.
7. Turn on the **lightmeter** and make sure to set it to measure Lux, not Fc or Lumen. Then, set the measurement range to 0 - 2000 Lux by pressing the “R” button until the range indicator on the bottom on the **lightmeter** screen underlines the 2000 Lux range, you may be able to measure using the lowest range if you are calibrating cost level 1, but if the **lightmeter** reads “OL” at any point, you will need to step up a range. Finally, because the measured value will constantly be changing, it helps to take only the maximum value. Point the **light sensor** (it looks like a white half-sphere) and set the **lightmeter** to “Max” mode then at a dark spot on the arena, this will freeze the measured value.
8. Start by measuring feeder 1 on the horizontal corner of the arena. Take the **light sensor** and hold it about 3 millimetres or less above each one of the four LEDs on the feeder, holding it in place for 2 to 3 seconds. The lightmeter will display the maximum value it was able to measure. It is recommended you measure each feeder at least twice, as there will be a wide variability in the measurement.
    a. If the value you measured is within ±10 Lux of the **target Lux**, record this value, the CCR value that produced it, and which feeder on which maze you were calibrating.
    b. If the value is *not* within ±10 Lux of the **target Lux**, use **calibration mode** on the microcontroller by sending a percent sign (‘%’) to finetune the CCR value and repeat steps 7 and 8 until you reach the target. For instructions on how to operate in **calibration mode**, see the Fine-tuned calibration of cost levels in calibration mode section in this guide. Once you have reached your target, save the new CCR value, and record it like you would in (a).
9. Move on to the next feeder. Once you are done, double check your data and hand it to a lab supervisor so that they may database it.

How to upload/update Firmware on a Texas Instrument Microcontroller Dev. Board

This guide assumes the user has an existing workspace and project for their microcontroller development board and is specific to Texas Instrument products. Please ensure you are using a Texas Instruments microcontroller development board.

Before starting, disconnect all microcontrollers from the PC and leave only the one to be updated connected. Make sure the microcontroller is on and functioning correctly by looking for a green light on the top of the microcontroller. If everything looks fine, proceed.

1. Start by opening Code Composer Studio. The software version shown in this guide is Version: 10.2.0.00009.
2. Drop down your project hierarchy on the Project Explorer panel on the left side of the screen.

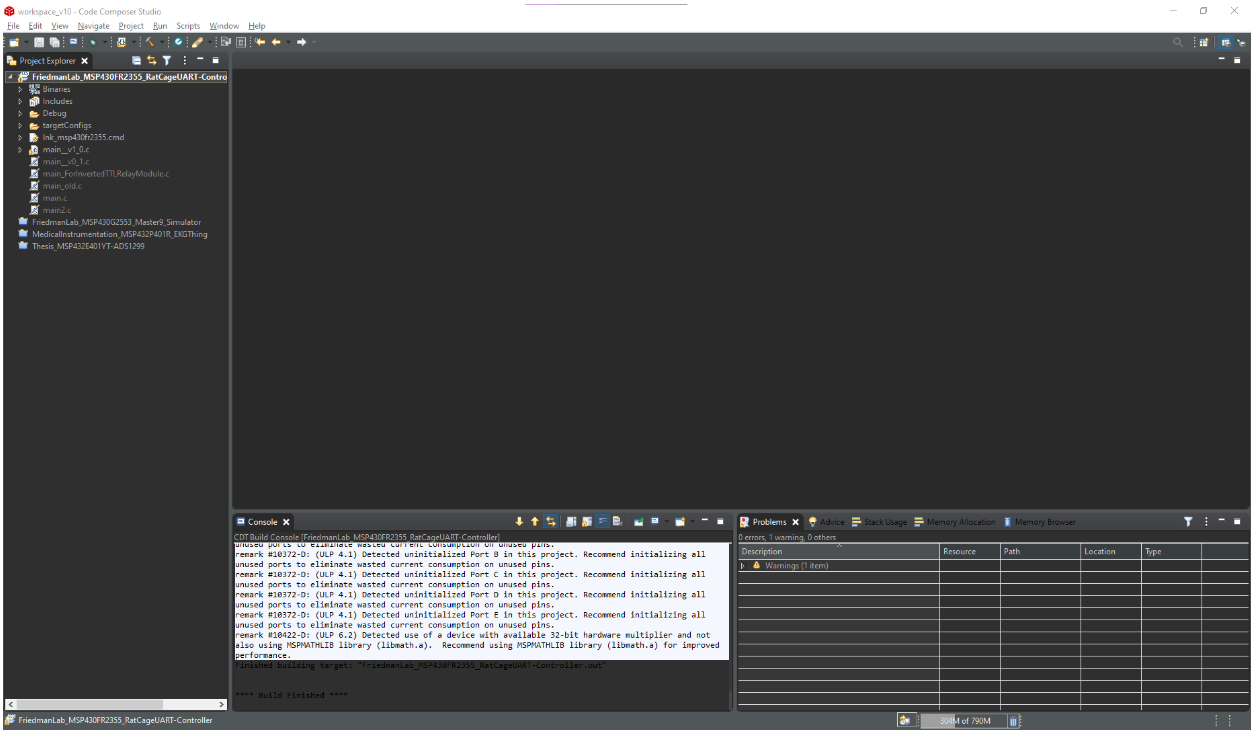

3. Right-click on the top of the hierarchy, then navigate to “Show in Local Terminal”. Click on “System Explorer” to open the folder where your entire workspace is saved.

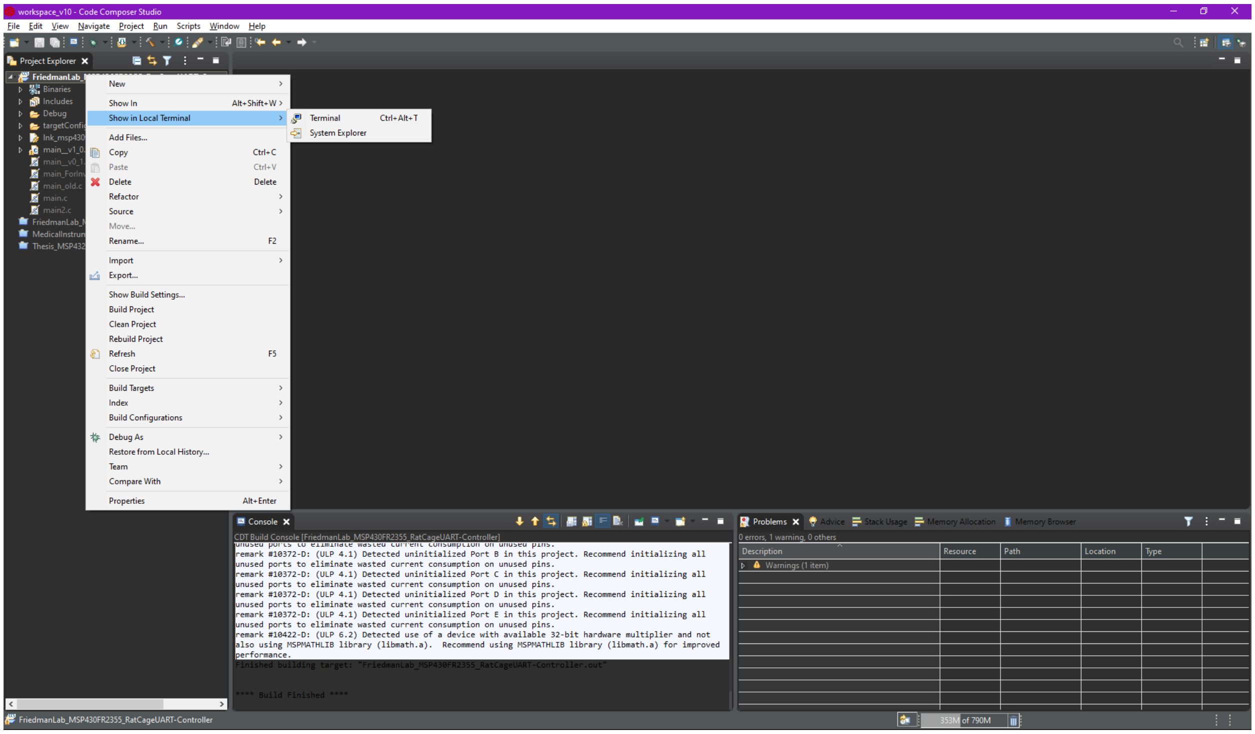

4. Navigate to the project where you wish to upload the new firmware. You will either see several .c files which contain code for the microcontroller, or you will not see any .c files.

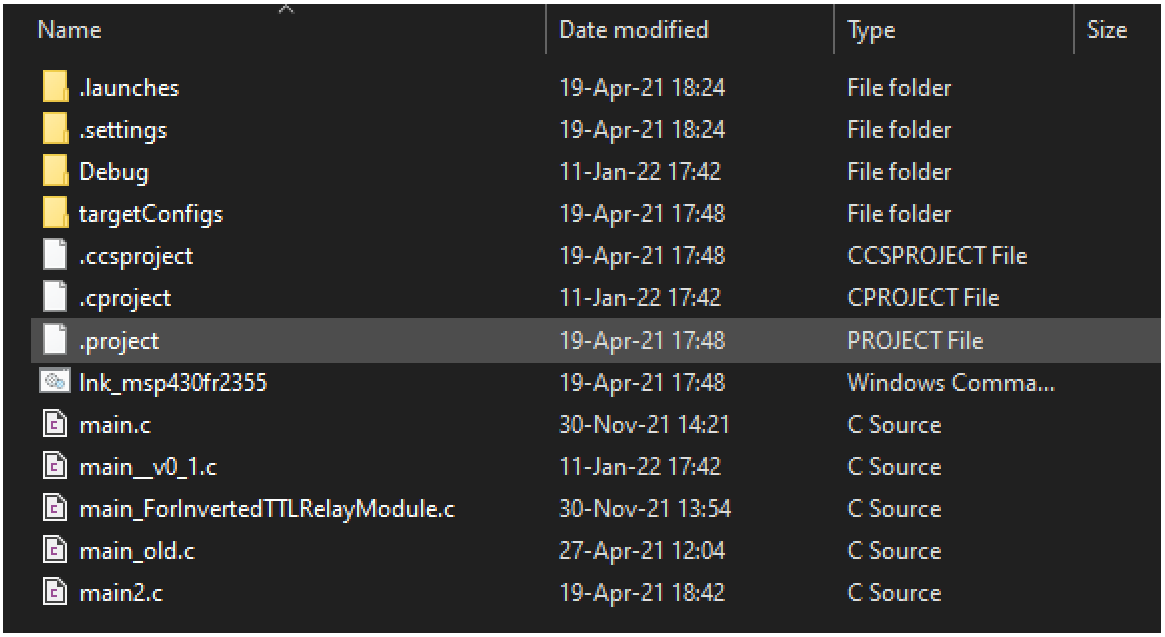

Name	Date modified	Type	Size
.launches	19-Apr-21 18:24	File folder	
.settings	19-Apr-21 18:24	File folder	
Debug	11-Jan-22 17:42	File folder	
targetConfigs	19-Apr-21 17:48	File folder	
.ccsproject	19-Apr-21 17:48	CCSPROJECT File	
.cproject	11-Jan-22 17:42	CPROJECT File	
.project	19-Apr-21 17:48	PROJECT File	
lnk_msp430fr2355	19-Apr-21 17:48	Windows Comma...	
main.c	30-Nov-21 14:21	C Source	
main__v0_1.c	11-Jan-22 17:42	C Source	
main_ForInvertedTTLRelayModule.c	30-Nov-21 13:54	C Source	
main_old.c	27-Apr-21 12:04	C Source	
main2.c	19-Apr-21 18:42	C Source	

5. Paste the new main.c file which contains the new firmware into this folder. In this case we will be updating from main__v0_1.c to main__v1_0.c. Close the file explorer window once you are done.

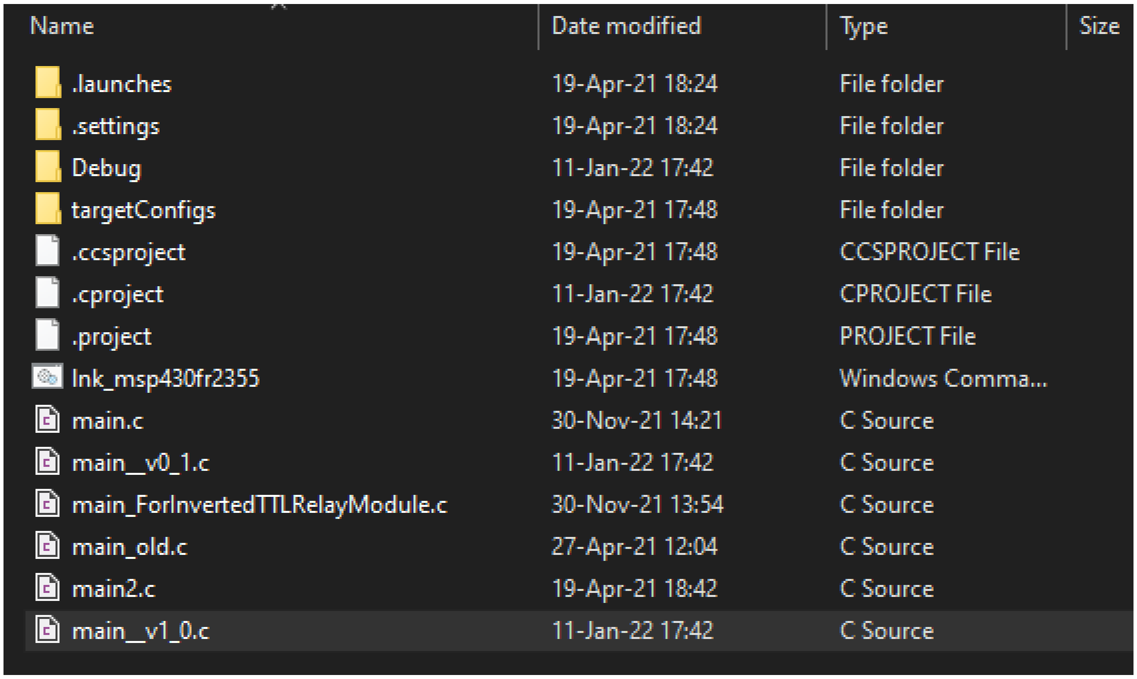

6. Go back into Code Composer. The new file should appear under your project hierarchy. Right click on the old firmware file and select “Exclude from Build”. This will make the program ignore this file when uploading code to the microcontroller.

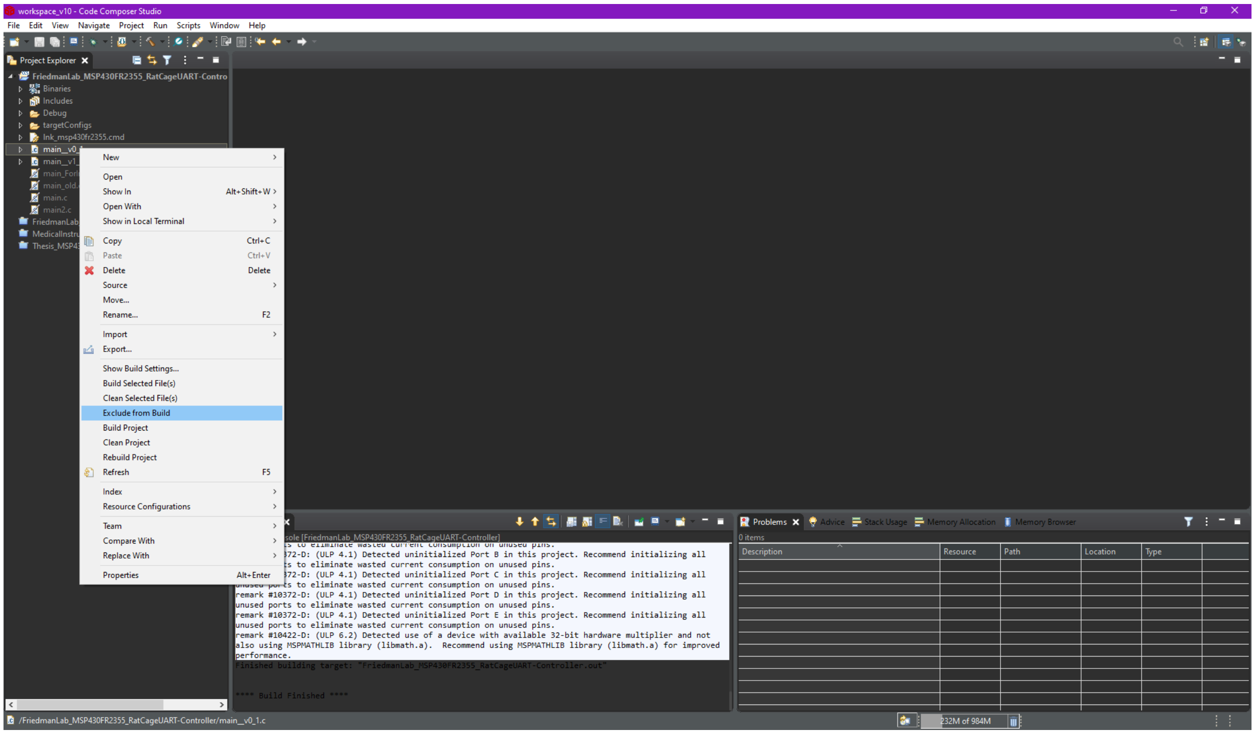

7. Double click on the new file to open it. Code should now appear on your screen. Click on the hammer icon which appears on the toolbar on the top. This will build the project and check the new code for errors. Text will appear on the console located on the bottom of

the screen, simply wait for it to finish. The first build may take longer than subsequent ones.

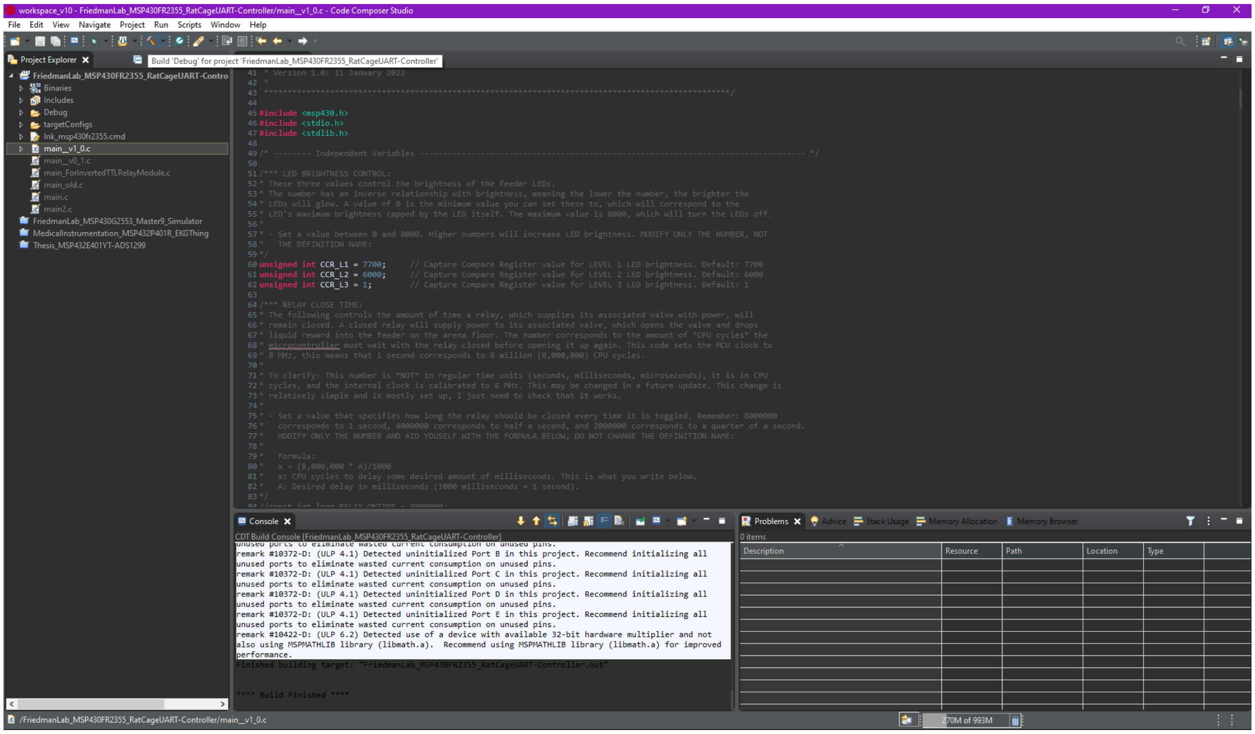

8. Click on the Flash icon located to the left of the hammer icon. This will upload the code to the microcontroller. Click okay if the program asks you to Save and Launch and then click Proceed on the next prompt.

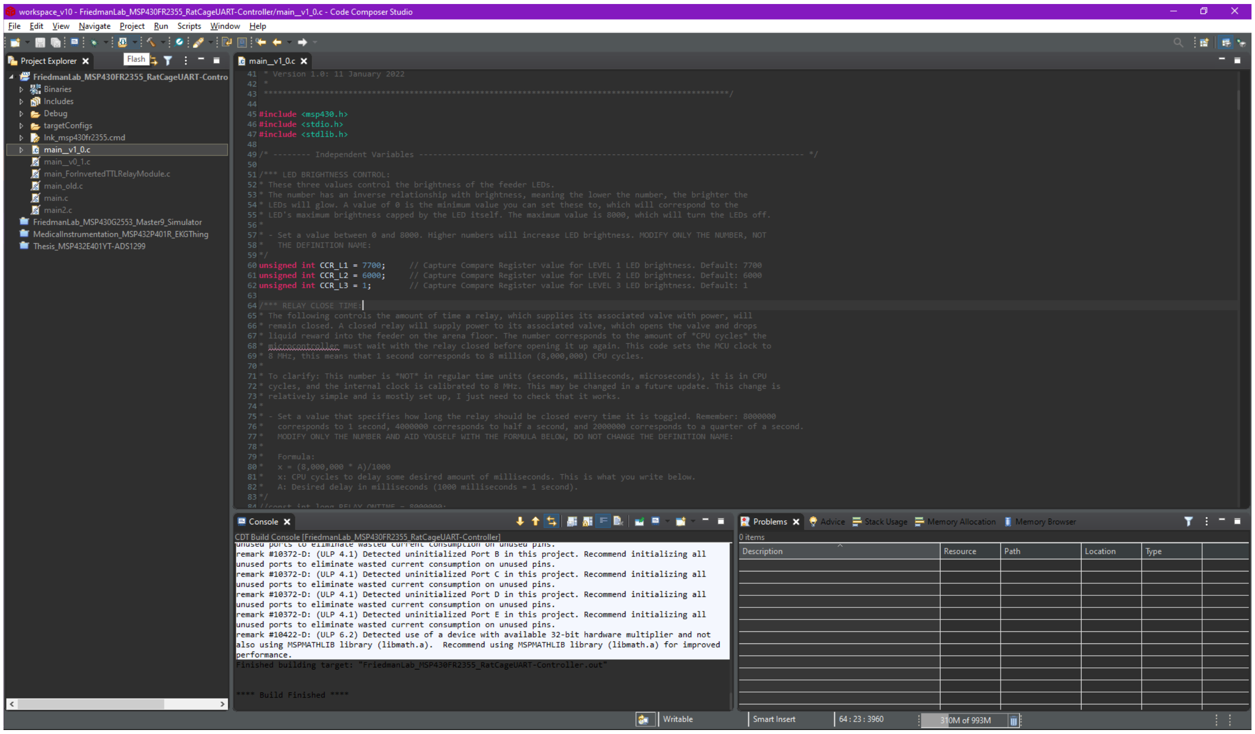

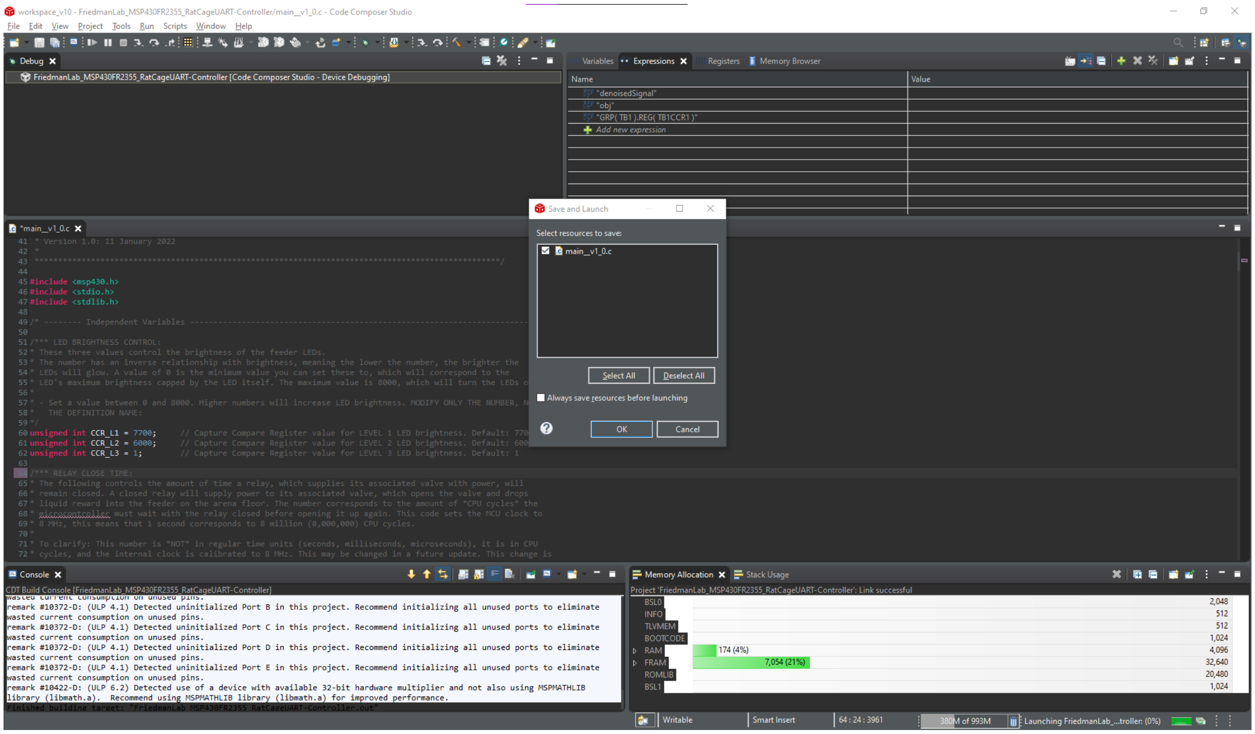

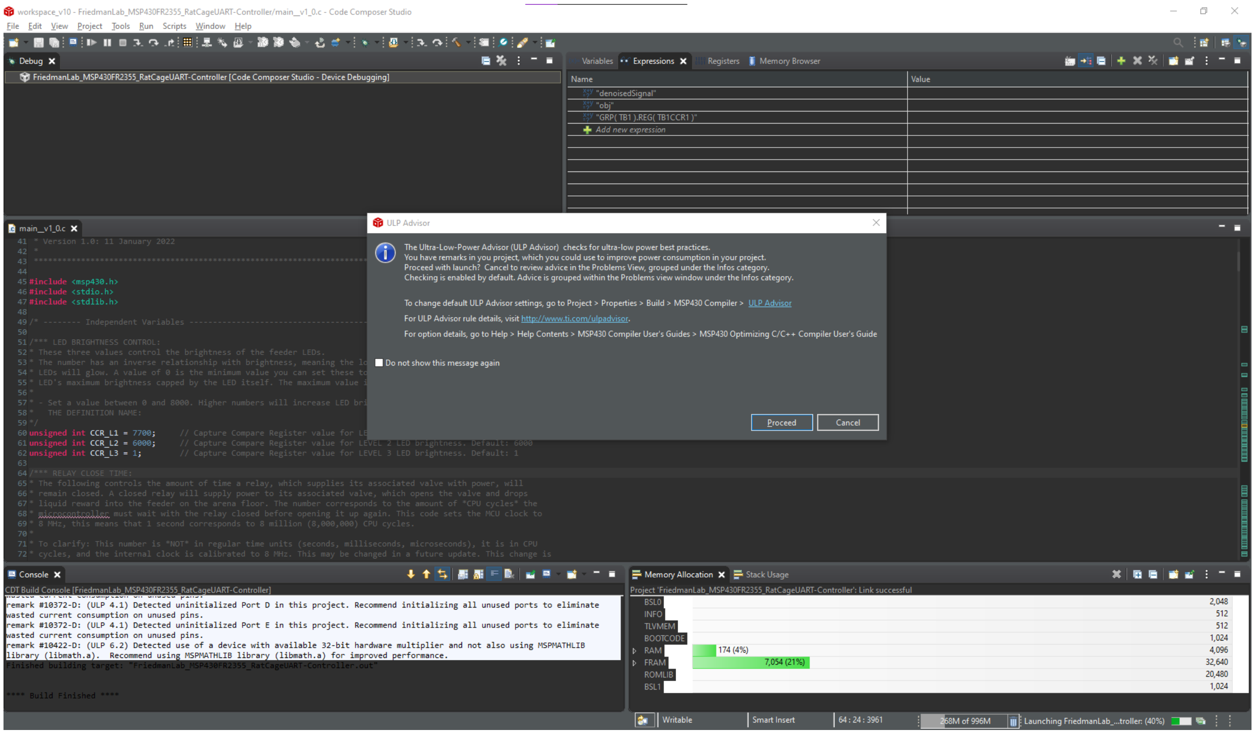

9. The code has now been uploaded to the microcontroller. You may begin using it immediately.

Sometimes, new firmware may be buggy, and one may need to roll back an update. To do this, repeat step 6 to re-include the old firmware code in the build, and instead exclude the new firmware code from the build. Then continue on to the next steps.

A note about RECORD and Noldus Ethovision

RECORD is a behavioural setup that is compatible with various animal tracking solutions, one example of this is Noldus' Ethovision XT. We have set up various experiments using this software which can be found in the RECORD github repository (https://github.com/rjibanezalcala/RECORD/tree/main/ethovision_experiments). In order for RECORD to function with Ethovision, the experiment's trial control must access batch scripts to send the appropriate commands to the arena microcontrollers and execute "trial events". Afterwards, the computer must wait for the MCU to finish executing a command and re-synchronise. Because of this, trial events tend to have some "overhead" and "tail".

We define "overhead" as the time it takes to access a batch script to execute it. The delay is determined by batch file access time and execution time. This time is typically negligible for a single event, but once several events are executed, it can add a small amount of delay to a trial, especially if the file access time on the computer is substantial. Additionally, the batch files introduce a second delay between sending commands to the different arenas. This is because one instance of Plink must be created to send commands to each arena. This delay may be tweaked on the "timeout" function in the batch files, however, it is not recommended to reduce this too much if all MCUs are sharing the same data bus, or are connected to the same USB port.

The "tail" is defined as the time it takes to execute the command, and re-synchronise devices post-execution. This is defined as command execution time on the MCU, plus trial control delays and re-synchronisation. This will likely introduce the largest delays in the trial, extending trial time. Different commands will take different times to execute on the MCU, most of the time, the commands that take the longest will be any commands that open the solenoid valves due to the delay needed to keep the valve open so that enough reward is dispensed as well as the TTL pulse that is sent after command execution. These delays can be tweaked on each MCU setup file and flashed in Code Composer (see How to upload/update Firmware on a Texas Instrument Microcontroller Dev. Board), or can be temporarily changed in configuration mode (see Configuring relay active time and Configuring TTL length). The tail time also includes re-synchronisation trial control delays. Without these delays, the computer and MCU might start running out of sync, and trial events will not be executed. The computer must then wait for an acknowledgement signal from the MCU each time a command is sent using a system delay, some hardware polling (on the I/O box), and finally an additional delay to allow the MCU to return to its baseline state. For more information on how these delays are handled, please see the figure included in this section.

The inter-trial interval (ITI)

While the ITI can be programmed on Ethovision in the "Acquisition" screen, the time a rodent waits in-between trials consists of a trial setup period, a trial cleanup period, and the programmed ITI (see figure below). This amounts to approximately 28 seconds if no tweaking is made to the original RECORD source files.

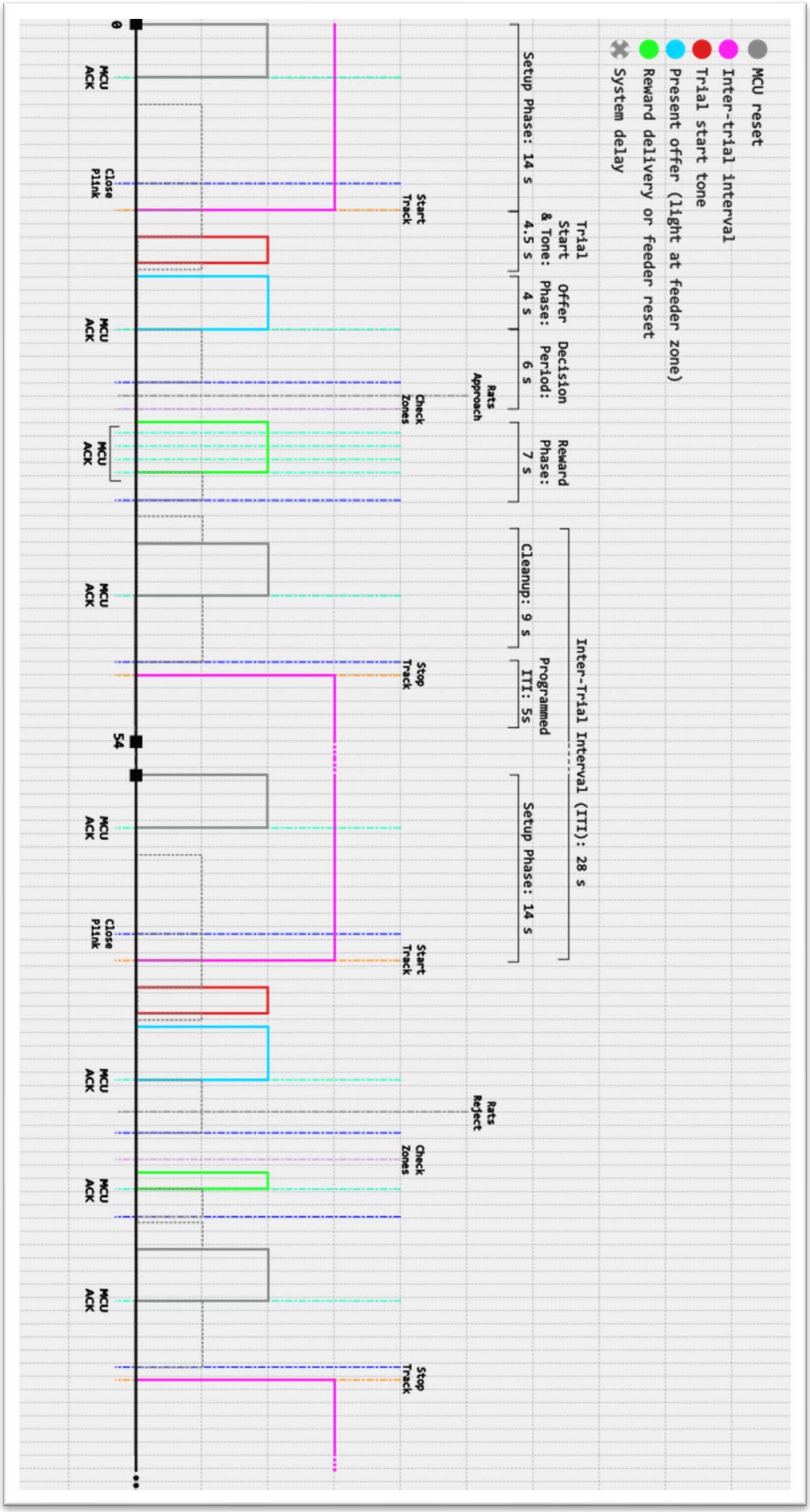
MCU reset
Inter-trial interval
Trial start tone
Present offer (light at feeder zone)
Reward delivery or feeder reset
System delay
Setup Phase: 14 s
Trial Start & Tone: 4.5 s
Offer Phase: 4 s
Decision Period: 6 s
Reward Phase: 7 s
Cleanup: 9 s
Programmed ITI: 5s
Inter-Trial Interval (ITI): 28 s
Setup Phase: 14 s
Rats Approach
Rats Reject
Check Zones

Troubleshooting

By Graham Waller and Raquel Ibáñez Alcalá

Hardware troubleshooting

Anything concerning the physical components of the RECORD electronics and arenas will be contained here. When in doubt, turn everything off and on → unplug and plug. Make sure everything, from the wires and cables, to the tubes that deliver sucrose, is connected.

Valves and Relays

Problem: Relay clicking, but valves not clicking…
Solutions: Before proceeding, check that the valve cable is connected to a valve on one side, and to the corresponding slot on the RECORD box or circuit board on the other side.Use PuTTY to activate each valve individually by sending “F” for valve 1, “G” for 2, “H” for 3, then “J” for 4, and identify which valve clicks and which does not. See ***Protocol for using PuTTY*** on slack in #important-documents.

If you have at least one valve that does click, swap the connectors for a working valve and a non-working valve then use PuTTY to activate both valves.

a. If the non-working valve clicks and the working valve does not, the problem may be at the valve cable. Proceed to step (1) below.
b. If the working valve clicks and the non-working valve does not, the valves may be the problem. Jump to step (3) below.

If no valves click, make sure that the valves are being supplied the correct voltage for them to operate (15 V - 24 V). Check that the power supply cable is connected to the RECORD box and the valve power supply is turned on, then test again. If the problem persists, proceed to step (1) below.

1. Check connections and cables for short circuits. Make sure all exposed copper wire is shielded; wires should not be touching neighbouring wires at the head of connections to the PCB or the valves. *If needed, tape each individual section of exposed wire with electrical tape to shield connections from one another.*
2. Check the connector at the valve side of the valve cable, it is possible that the wire may have come loose from its housing and might not be making contact with the connector on the valve. Alternatively, the contacts on the connectors may be dirty. *Disconnect the valve from the cable and clean the contacts using a damp paper towel, then connect them again.*

3. If the problem persists, it may be necessary to either clean or replace the non-working valve(s). To clean the valve, dismount it from the valve stand, remove the solenoid from it, and submerge the valve plunger in water for at least 1 hour, then test it again.

Problem: Relays not clicking…
Solution: Likely caused by a connection issue between the microcontroller and relays. Carefully open the RECORD box and look for the wires that connect the microcontroller (in red) and the relays (long blue board featuring several blue blocks with white writing). If these wires are disconnected, the relays will not click. Refer to the ***Electronics build guide*** to repair and ask a lab supervisor for help, **do not connect or disconnect anything on the PCB or microcontroller without supervision.**

Problem: Valves and relays are clicking, but no sucrose is being dispensed…
Solution: This is likely caused by air being trapped in the system. Before proceeding, check that all tubing is connected to its corresponding feeders. Check for air bubbles in the tubing between the syringes and the valves, and between the valves and the arena feeders. If there are bubbles present, activate the valves a few times using PuTTY, or run the *system_cleanup* batch script routine. You may also force sucrose solution into the system by using the syringe plunger to push down the solution while the valves are open. To do this, take a syringe plunger and while activating the valve with PuTTY, push the plunger down the syringe to flush liquid through and clear the blockage. When finished, check for leaks in the system and fix them accordingly.

Lights

Problem: Light does not turn on for one or more feeders...
Solutions:

1. Make sure that the feeder lights are active at any (visible) brightness. Use PuTTY to turn the lights on. See ***Protocol for using PuTTY*** on slack in #important-documents.
2. Check that the non-working feeder lights are connected to the LED cable, this should be a two-wire connector that attaches to the cable. If the feeder is connected, make sure the lights are not connected backwards. Some feeders will have a blue and black wire pair, others will have a yellow and green wire pair, and (though uncommon) others may have a red and black wire pair. Make sure that the black wire on the LED cable (representing ground) is connected either to the black or green wire on the feeder LEDs, and the red wire on the LED cable (representing live wire) is connected to the yellow, red, or blue wire on the feeder LEDs. The light should turn on after this.
3. If the light does not turn on, or if one some LEDs turn on on that feeder, lift the feeder up by pushing the “tail” of the feeder up (where the tubing attaches) and pull it up and off the feeder base. Flip the feeder over and look at the underside. *Carefully* separate any exposed metal contacts on the LED ring bundle, making sure there are no short-circuits in the loop. Handle these wires with care, making sure not to break any of the connections. The lights should turn on after separating all the wires.

4. If the lights still do not turn on, check connections and cables for short circuits. Make sure all exposed copper wire is shielded; wires should not be touching neighbouring wires at the head of connections to the PCB or the valves. *If needed, tape each individual section of exposed wire with electrical tape to shield connections from one another.*

Problem: Two lights turning on at once…
Solution: The given cable's copper wires are likely touching at the white connector on the end of the cable which connects to the RECORD box. Use electrical tape to shield each individual exposed copper wire to prevent the wires from touching one another.

Arena Pieces

Pieces will wear down and come apart over time, this process can happen a lot sooner if the rats gnaw on the arena. Simply replace faulty pieces with new parts and/or glue them back together.

Ethovision troubleshooting

Good rule of thumb to avoid issues in EthoVision **is to set the program priority** for EthoVision to *"Real Time"* via TaskManager (ctrl → shift → esc) and having other programs (especially big ones like MatLab and Google Chrome) closed while running trials.

Problem: Trials getting stuck at the beginning stage...
Alternative problem 1: Trial never ends…
Alternative problem 2: Lights stay on after a trial ends…
Solution: Caused by desynchronization between the RECORD system and the animal tracking software. *Stop the trials.* Close all command windows that may have been left open, then turn the microcontrollers off, wait up to 10 seconds, and turn them back on. *Do this only after stopping the trials, doing so during a trial will desynchronize both systems again!*

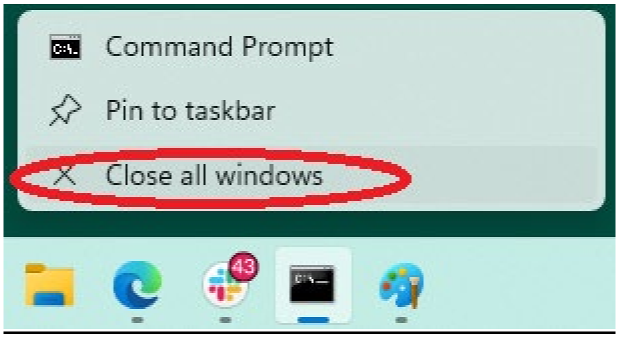

If the problem persists, check that the microcontroller is connected to the I/O interface via one of the data lines on the hardware synchronisation cable. It is possible that the connection might need to be flipped around so that the output from the microcontroller goes to the TTL input line, and the input for the microcontroller goes to the TTL output line.

Changes to the trial control may be needed if the problem persists as the trial may not be allowing enough time for the microcontroller to resynchronize with the trial after running a command, or the serial communication channel may be closing prematurely.

Problem: EthoVision crashed and data was not recovered…
Solution: This is more of a workaround than a solution. Create a backup of the experiment, then create a new experiment and delete any other behavioural task trial control structures from the one which you wish to run. A good practice is to keep different task versions in separate experiments, and always back up your experiments. This is to keep bugs in the program isolated to only one experiment.

Problem: A feeder has a significant delay before delivering sucrose in one or more arenas…
Solution: Check configuration for zones under the trial control settings, make sure that options for each decision-making condition variable (box x accepts, box y rejects, etc.) are set to the appropriate settings. Also increase the time for which accept/reject conditions must be met (for example 0.5s might be too low, and should be increased to 1.0s).

> **Example:** We incurred this error when the condition check in “box 4 rejects” was set to “when centre-point is in all zones simultaneously” instead of “in any of the zones” in the trial control settings. This made the trial hang because that condition can never be met as the rat cannot exist in more than one zone simultaneously, and the program was waiting until the “box 4 accepts” condition was met.

Problem: The Windows “USB disconnect” sound plays when more than one valve/relay clicks, then the current trial never ends…
Solution: This may be due to a power supply overload in the system, which makes the microcontrollers reset and de-synchronizes them from EthoVision.

If this happens to you in the middle of running trials, manually stop the trial, close all command windows, and continue running trials.

To prevent this problem from happening again, modify the trial control in EthoVision so that the delivery stage at each arena is delayed 0.5 seconds after the previous one, this will ensure that valves don’t click at the same time and that the power supply for the relays is not overloaded.

Problem: Ethovision gives an error saying calibration lines are significantly different, when editing arena settings…

Solution: A new calibration line that is significantly smaller or bigger than the others may have been accidentally added. This causes a conflict between the existing calibration lines which measure the arenas at 64.5cm. To solve, delete the new calibration line that was accidentally created.

Problem: RECORD hangs at the reward delivery stage of the trial…
Solution: Sometimes during a trial, the RECORD system will hang at any stage of the trial, especially the reward delivery. This is normal. Reward delivery is heavily dependent on animal detection and this may sometimes cause delays depending on the animal's position. If the system does not respond after 10 - 20 seconds, manually stop the trial and troubleshoot the detection settings or arena settings. *Make sure to close all command windows (small black windows) before starting the next trial.* This will ensure that the RECORD system will be responsive for the next trial.

Firmware troubleshooting

It is rare for the firmware running on the microcontroller to act up. Firmware is debugged before uploading it to the microcontroller. Fixes to the firmware will require C language programming skills, but a good solution that will work most of the time is to turn the microcontroller(s) off, wait 10 seconds, then turn it back on.

Supplemental Note 2

R.E.C.O.R.D.

(Reward-Cost in Rodent Decision-making)

Electronics build guide

Revision 1.1

Overview

This assembly guide will instruct you in your creation of the electronic components of our custom arena behavioural apparatus. These components are made utilising basic wires, lights, and cables, to allow for a tailormade task to be used in the study of rodent decision-making in a cost-effective and open-science friendly way. The customization of our system allows for on the fly changes and adaptations to the experimental environment and process, as well as animal behaviour. Further, it enables the interchangeability and replacement of parts as they wear down over the course of thousands of behavioural trials. In this guide you will find the parts and materials, dimensions, and measurements, along with links and assembly instructions, to our custom electronic system as well as the code and programs that we are using to manipulate it.

LED assemblies and cable

CAUTION: You will be working with very hot equipment in this section. Please make sure you take the proper precautions to not burn yourself, other team members, or any objects inside and outside of your work area!

The LED assemblies on the arena signal a cost (associated with the food rewards) at each corner. Each arena houses four of these assemblies, mounted onto every feeder piece in the arena. Each assembly is made up of four LEDs connected in parallel and are controlled by a single data wire, with a single ground wire providing them ground. By the end of this section, you should have 4 LED modules, each made up of 4 LEDs, with one red wire and one black wire coming out of the assembly.

Materials

- *16 blue LEDs*
- *Black wire*
- *Red wire*
- *Dupont wire, female, red*
- *Dupont wire, female, black*
- *Two-conductor speaker wire*
- *Electrical tape*
- *soldering iron*
- *Solder*
- *Wire strippers*
- *Wire cutters*
- *Multimeter*

1. **LED Anatomy**

Every LED has two “legs”; one receives voltage from a source and the other completes the circuit by allowing the current to flow towards ground. These are called “**anode**” and “**cathode**”, respectively. The anode and cathode can be identified by observing the LED legs, the **anode** is the **longer leg** while the **cathode** is the **shorter leg**, see Figure 2.1 for reference.

Figure 2.1. Identifying the cathode and anode:

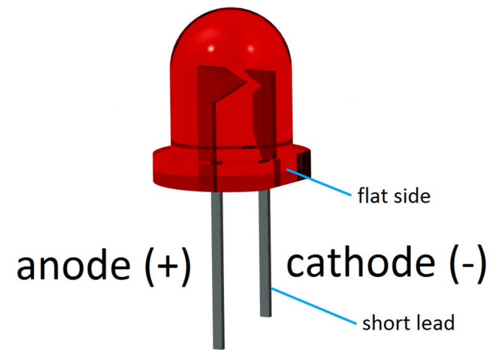

Figure 2.1 A: Illustration showcasing the anode vs cathode-based length leg size.

2. **Preparing the Wires**

   Using the wire cutters, measure and cut three *4 cm* segments of both **red and black wire**. Strip both ends of all wire segments using wire strippers, being careful not to cut the wire (or yourself) by accident.

3. **Soldering Preparation**

   Make sure the soldering iron is resting in its holder and nothing is touching the metal tip at the end. The soldering iron will get *very hot very quickly!* It’s important to handle this device with care. Turn the soldering iron on and wait for it to heat up. *DO NOT HOLD THE SOLDERING IRON BY THE TIP, YOU WILL BURN YOURSELF. Always grab the soldering iron by its heat insulated padding.*

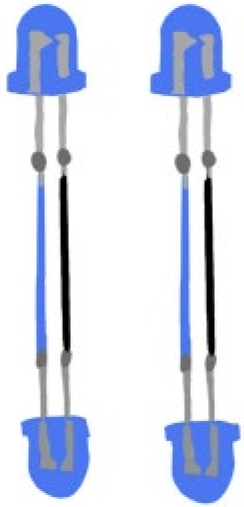

4. **Assembling the LED ring part 1**

   The **cathode** will be colour coded **black** and the **anode** will be colour coded **red** on the LEDs. *It is extremely important that the LED is colour coded and soldered correctly in the next steps or else the LED may not light up!*

   Connect two LEDs together by soldering their cathodes together with a black wire segment and their anodes with a red wire segment. Repeat this step twice to end up with two two-LED circuits.

5. **Assembling The LED Ring part 2**

   Use the remaining black wire segment to bridge the cathodes of the two LED pairs together. Then, using the red wire segment, bridge the anodes of the same two LEDs or of the other two LEDs. This will result in either a U-shaped or O-shaped LED circuit where all four LEDs are connected in parallel.

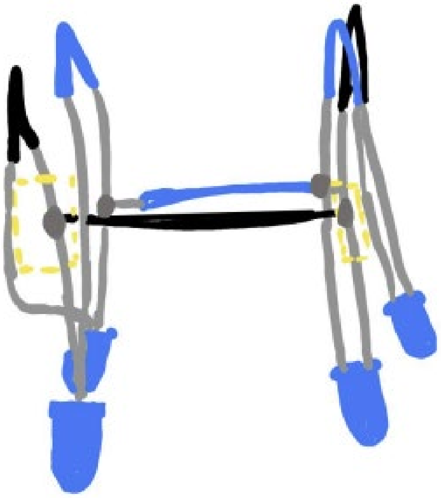

6. **Adding a connector to the LED assembly**

   To make the LED rings detachable (and thus replaceable), a connector is needed. Create a new set of red and black wires with a female Dupont connector on one end, and a stripped end. Solder the stripped end of the red connector wire to the anode of one of the LEDs in the assembly, and the black connector wire to the cathode of another LED in the assembly.

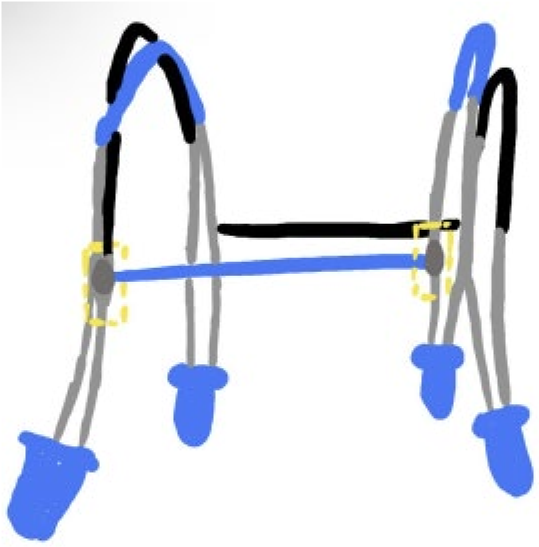

   For a U-shaped arrangement, we recommend soldering the connector wires to the two free-hanging LEDs. For the O-shaped arrangement, the connector wires can be soldered to any two adjacent LEDs.

   Repeat steps 2 through 6 to create three more LED assemblies for one RECORD arena.

7. **Preparing for the LED cable**

   Cut four long segments of two-conductor copper speaker cable and strip about 1 cm of the inside black and red wires at both ends. The segments should be long enough to put some amount of distance between the RECORD electronics and the RECORD arena. It is recommended that this distance is measured beforehand and to add about 1 metre to it for slack.

8. **Creating the JST connector end of the cable**

   On one end of the four cables, twist and solder the four black wires together, then solder an additional wire to the end of this joint.

   Create five wires with a JST connector pin on one end and strip the other end.

Solder the red speaker wires to four of these wires and insert them into pins 1 through 4 of the JST-XH connector housing.
Solder the black speaker wire bundle to the last connector wire and insert it into pin 6 of the JST-XH connector housing.

Put this end of the wire aside for now.

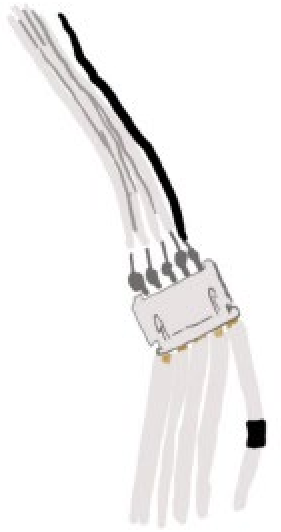

9. **Creating the LED assembly connector**

   On the other end, a male Dupont connection for an LED assembly must be made for each cable. Solder a red male Dupont wire to the red speaker wire and a black male Dupont wire to the black speaker wire. Wrap any exposed solder joints with electrical tape.

10. **Testing connection continuity**

    Connect the LED assemblies to the Dupont connector end of the completed LED cable. Use the multimeter to test continuity between the connected LED terminals and each pin of the JST-XH connector. Keep track of which cable corresponds to which LED assembly and label the wire and LEDs appropriately.

Valves and valve cables

CAUTION: You will be working with very hot equipment in this section. Please make sure you take the proper precautions to not burn yourself, other team members, or any objects inside and outside of your work area!

The solenoid valves are involved in reward (food) delivery to the animal in the arena. These valves are driven by 20 - 25 Volts direct current (DC) which will be delivered by an external power source. By the end of this section, you should have a set of four valves and a valve cable with a 6-position female JST-XH connector.

Materials

- *4 Solenoid Valves*
- *Speaker cable*
- *1 Six-position female JST-XH connector*
- *Dupont wire (female)*

- *Dupont wire (male)*
- *A soldering iron*
- *Solder*
- *Wire strippers*
- *Wire cutters*
- *And a Multimeter*

1. **Preparing the cable**
   Using the wire cutters, measure and cut a section of **speaker cable** for each valve, to end up with 4 segments of speaker cable. The segments should be long enough to put some amount of distance between the RECORD electronics and the RECORD arena. It is recommended that this distance is measured beforehand and to add about 1 metre to it for slack.
   Strip both ends of the speaker wire. One end will need female Dupont connectors and the other will be housed by a 5-pin JST-XH connector.

   Prepare 4 wires with one female Dupont connector each, 8 with a male Dupont connector, and 5 with an XH pin on one end. The other end of each of these wires should be stripped and prepared for soldering.

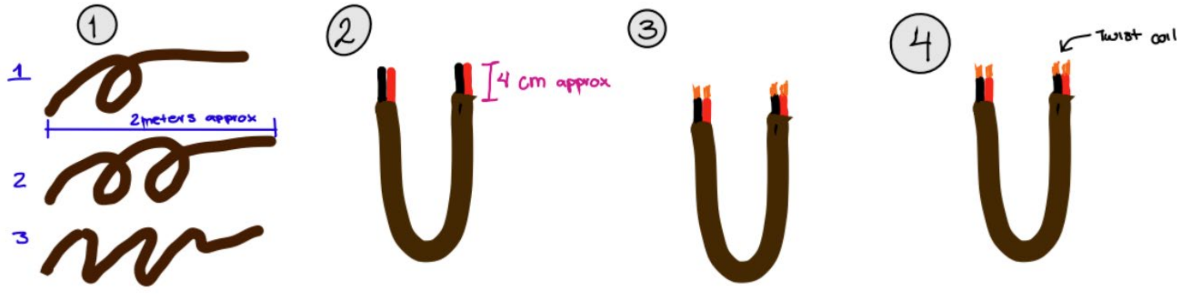

2. **Soldering Preparation**

   Make sure the soldering iron is resting in its holder and nothing is touching the metal tip at the end. The soldering iron will get *very hot very quickly!* It's important to handle this device with care. Turn the soldering iron on and wait for it to heat up. *DO NOT HOLD THE SOLDERING IRON BY THE TIP, YOU WILL BURN YOURSELF. Always grab the soldering iron by its **heat insulated padding.***

3. **Soldering connector wires to the valve**

   Each valve has three terminals, two on the top and one on the bottom. Solder the stripped end of two male Dupont connector wires to one each one of the top terminals. Because the valves are not polarised, colour coding is not required.

4. **Creating the connector on the wire**

As with the LED cables, twist the four internal black wires on one end of the stripped speaker wire and solder them together, then solder a wire with an XH pin to the end of this joint. Then, solder one wire with an XH pin to each of the internal red wires on the same end of the speaker cable. All valves will be sharing one ground through one terminal and will be supplied with power through the other terminal.

On the other end of the cable, solder a female Dupont connector wire to each of the internal speaker wires.

Lastly, introduce the five XH pin wires into the JST-XT housing. Pin 5 will house the 4-wire joint wire and pins 1 through 4 will house the remaining wires.

5. **Testing connection continuity**

   Connect the valves to the Dupont connector end of the completed valve cable. Use the multimeter to test continuity between the connected valve terminals and each pin of the JST-XH connector. Keep track of which cable corresponds to which valve and label the wire and valves appropriately.

Power and synchronisation cables

The power cable in the record system simply supplies the valves with the power they need to operate. This is done by connecting the cable to the RECORD circuit board, which in turn supplies that voltage to the relays that open and close the circuit that carries the external power source voltage. The synchronisation cables on the other hand simply serve as a link between the RECORD system and an external system. How the synchronisation cables are built will vary with respect to the external system that is in use, but the end of the cable that connects to RECORD will be the same.

Herein we will explain how to build synchronisation cables for the Noldus Ethovision system and the Inscopix nVista DAQ box system.

Materials

- Speaker wire (x 1)
- Cat 5 cable (Noldus) (x 1)
- Coaxial cable (Inscopix) (x 1)
- Dupont wire or Dupont connectors crimpable pins and housings (2 female, 2 male)
- 2-position JST-XH connector housing (x 2)
- XH crimpable pins (x 4)
- Soldering iron and solder
- Wire cutters

- Wire strippers (0.80 - 2.6 mm^2, AWG 10-20)
- Crimper (0.1 - 0.5 mm^2, AWG 26-20)

Power cable

1. **Preparing the cable**
   Using the wire cutters, measure and cut a section of **speaker cable.** The segment should be long enough to put some amount of distance between the RECORD electronics and power supply. It is recommended that this distance is measured beforehand and to add about 1 metre to it for slack.
   Strip both ends of the speaker wire. One end will need male Dupont connectors and the other will be housed by a 2-pin JST-XH connector.

   Prepare 2 wires with one male Dupont connector each and 2 with an XH pin on one end. The other end of each of these wires should be stripped and prepared for soldering. Alternatively, the pins and connectors may be crimped onto the speaker wire directly if the wire is small enough to fit in the crimp.

2. **Soldering Preparation**

   Make sure the soldering iron is resting in its holder and nothing is touching the metal tip at the end. The soldering iron will get *very hot very quickly!* It's important to handle this device with care. Turn the soldering iron on and wait for it to heat up. *DO NOT HOLD THE SOLDERING IRON BY THE TIP, YOU WILL BURN YOURSELF. Always grab the soldering iron by its* ***heat insulated padding.***

3. **Creating the connectors on the wire**

   Solder a wire or crimp an XH connector pin to each of the internal wires on one end of the speaker cable. Make one of these wires as "GROUND" and make sure to keep track of it.

   On the other end of the cable, solder or crimp a male Dupont connector to both internal speaker wires.

   Lastly, introduce the two XH pin wires into the JST-XT housing. Pin 5 will house the 4-wire joint wire and pins 1 through 4 will house the remaining wires.

4. **Identify the appropriate wires on the Noldus synchronisation cable**

   Using the following diagram, taken from the "USB-IO box / Mini USB-IO box for EthoVision XT 13.0" reference guide, identify and solder the female Dupont wires to "Add-On Supply V+ 18 Volt" and "ground" and connect them to the cable you just made.

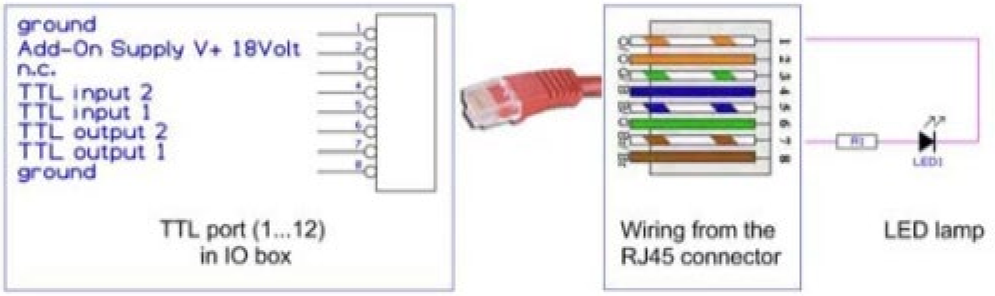

Figure 6 *Wiring scheme for connecting a LED lamp.*

Noldus synchronisation cable

1. **Preparing the cable**

   Follow the same steps followed for the **power cable**, but instead of using male Dupont connectors, use female connectors. This will create a detachable interface between the RECORD system's cable and the Noldus sync cable.

   Because the Noldus I/O box uses ethernet connections, we will be using a CAT5 cable for synchronisation. Expose the ends of each internal wire in the CAT5 cable to prepare them for soldering/crimping.

2. **Identify the CAT5 wires**

   Using the following diagram, taken from the "USB-IO box / Mini USB-IO box for EthoVision XT 13.0" reference guide, identify and solder the male Dupont wires to "TTL input 1" and ground and connect them to the cable prepared in step 1. Solder additional wires if needed but be sure to make more sync cables for any other TTL being used.

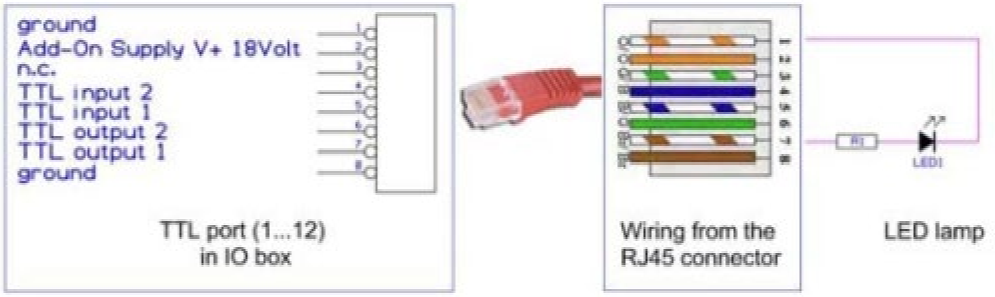

Figure 6 *Wiring scheme for connecting a LED lamp.*

Inscopix synchronisation cable

1. **Preparing the cable**
   Using the wire cutters, measure and cut a section of **coaxial cable.** The segment should be long enough to put some amount of distance between the RECORD electronics and power supply. It is recommended that this distance is measured beforehand and to add about 1 metre to it for slack.
   Expose the internals of this cable. The outer shielding will need to be grounded and the internal conductor will carry the TTL signal.

   You'll need a wire with an XH pin crimped onto it and a wire with a female Dupont connector for this build. The other end of each of these wires should be stripped and prepared for soldering.

5. **Soldering Preparation**

   Make sure the soldering iron is resting in its holder and nothing is touching the metal tip at the end. The soldering iron will get *very hot very quickly!* It's important to handle this device with care. Turn the soldering iron on and wait for it to heat up. *DO NOT HOLD THE SOLDERING IRON BY THE TIP, YOU WILL BURN YOURSELF. Always grab the soldering iron by its* ***heat insulated padding.***

6. **Creating the connectors on the wire**

   Solder the XH wire to the internal conductor of the coax cable, then solder the Dupont wire to the wire that wraps around the coax shielding. Mark the Dupont wire as "GROUND". Wrap all exposed cable internals with electrical tape, making sure that the shielding wire does not touch the internal conductor wire, then insert the XH wire into the JST-XH housing.

7. **Grounding the coaxial shielding**

   Connect the Dupont wire that was soldered to the shielding wire on the coax cable directly to a GROUND pin on the microcontroller. This will ensure that the TTL signal is clean and does not float.

Hardware connections

Microcontroller

The microcontroller unit (MCU) provides control for all electronic components in the RECORD system (excluding any external spatial tracking software). The following table and figure describe the pin that drives each electronic component. All connections from the MCU to its respective component listen on the table below (except for the eUSCI pins) are indirect, as they pass first through the printed circuit board (PCB). We use the Texas Instruments MSP-EXP430FR2355 launchpad development kit (https://www.ti.com/tool/MSP-EXP430FR2355). For additional documentation on this microcontroller, please visit the manufacturers website (https://www.ti.com/product/MSP430FR2355).

**Peripheral Type**	**Associated Electronic Component**	**Pin on MSP430-FR2355 Launchpad**
GPIO	TTL out (ACK signal)	P3.0
GPIO	Relay 1 or 5	P3.1
GPIO	Relay 2 or 6	P3.2
GPIO	Relay 3 or 7	P3.7
GPIO	Relay 4 or 8	P3.4
GPIO	TTL in (SYNC signal)	P3.5
Capture/Compare Output, Timer B1 (PWM)	Cost LED Ring 1	P6.0
Capture/Compare Output, Timer B2 (PWM)	Cost LED Ring 2	P6.1
Capture/Compare Output, Timer B3 (PWM)	Cost LED Ring 3	P6.2
Capture/Compare Output, Timer B4 (PWM)	Cost LED Ring 4	P6.3

Capture/Compare Output, Timer B5 (PWM)	Trial Indicator LED	P6.4
eUSCI, UART RXD	Serial Communication Receive	P1.6
eUSCI, UART TXD	Serial Communication Transmit	P1.7

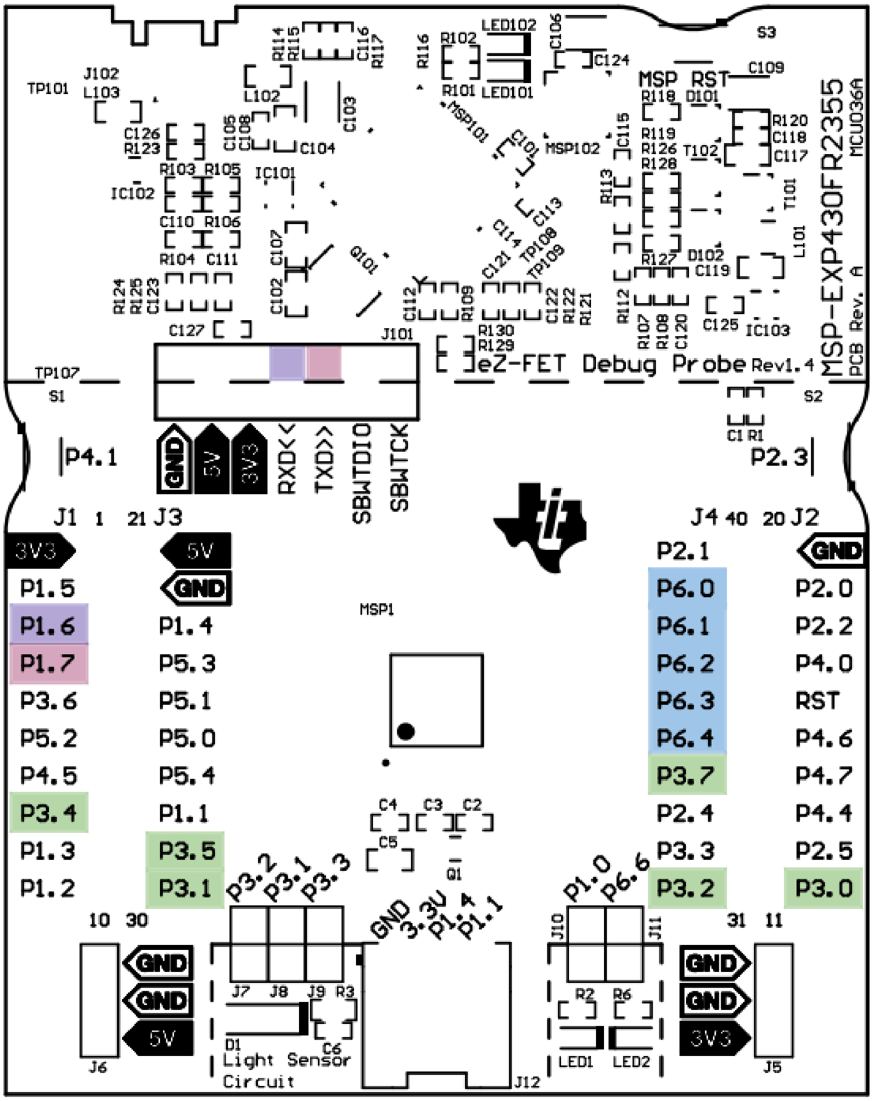

PCB layout image credit to Texas Instruments. https://www.ti.com/tool/MSP-EXP430FR2355#design-files (SLAR152.zip, "MSP-EXP430FR2355_PCBlayers.pdf" pg. 1)

Relay Shield

Position on Relay Shield (For each relay)	Connection on PCB	Name
VCC	H4.9	Positive power supply
GND	H4.10	Ground
NO	CONN7.x / CONN8.x	Normally open terminal
NC	Not Connected	Normally closed terminal
COM	CONN9.x / CONN10.x	Common voltage terminal
INx (1 through 8)	H4.x (1 through 8)	Input trigger signal terminal

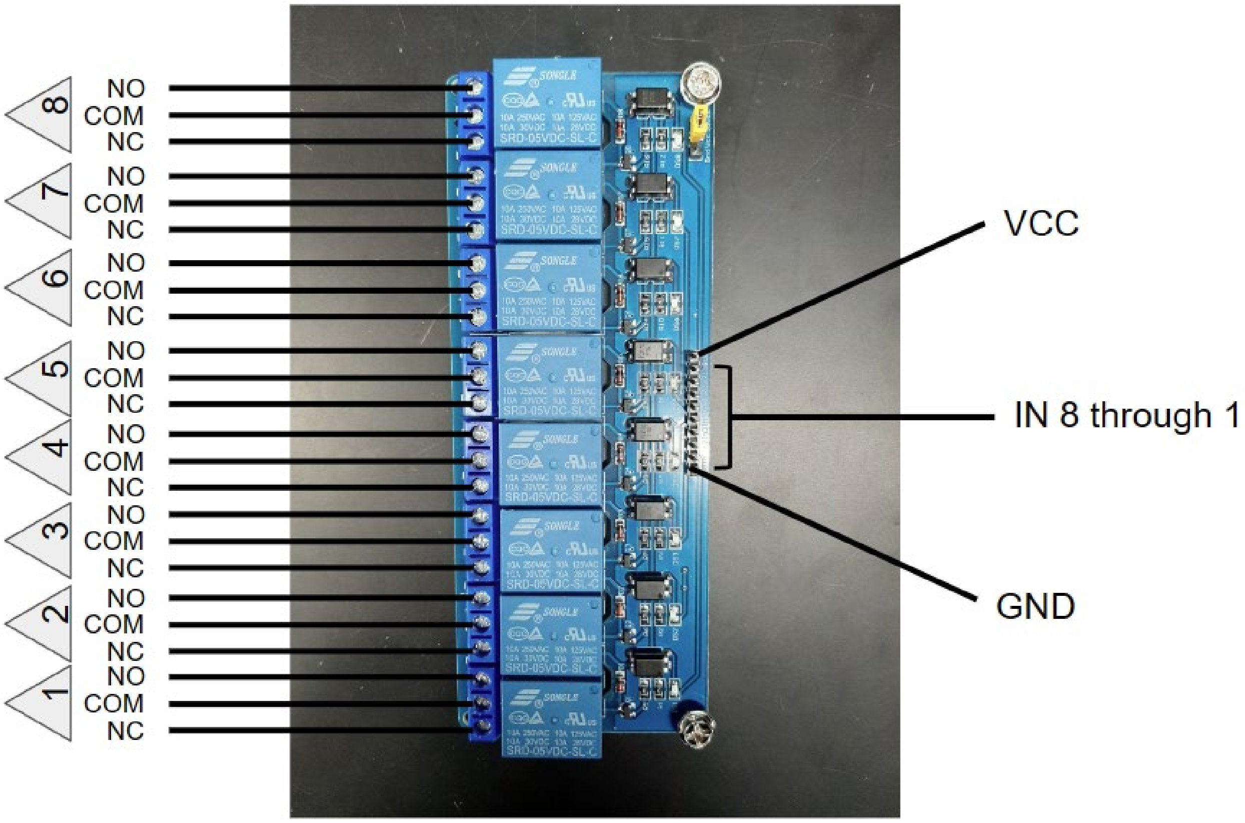

Printed Circuit Board (PCB)

The PCB can relay up to two different RECORD microcontroller inputs and outputs or drive up to eight cost/reward components from a single microcontroller, if the firmware and hardware allow it. No cost/reward components are connected directly to the microcontroller, rather, they are connected to the PCB to be held tightly in place, while the PCB is connected to the microcontroller through jumper wires. See “Microcontroller, Relay, and PCB connections” for a detailed connection table.

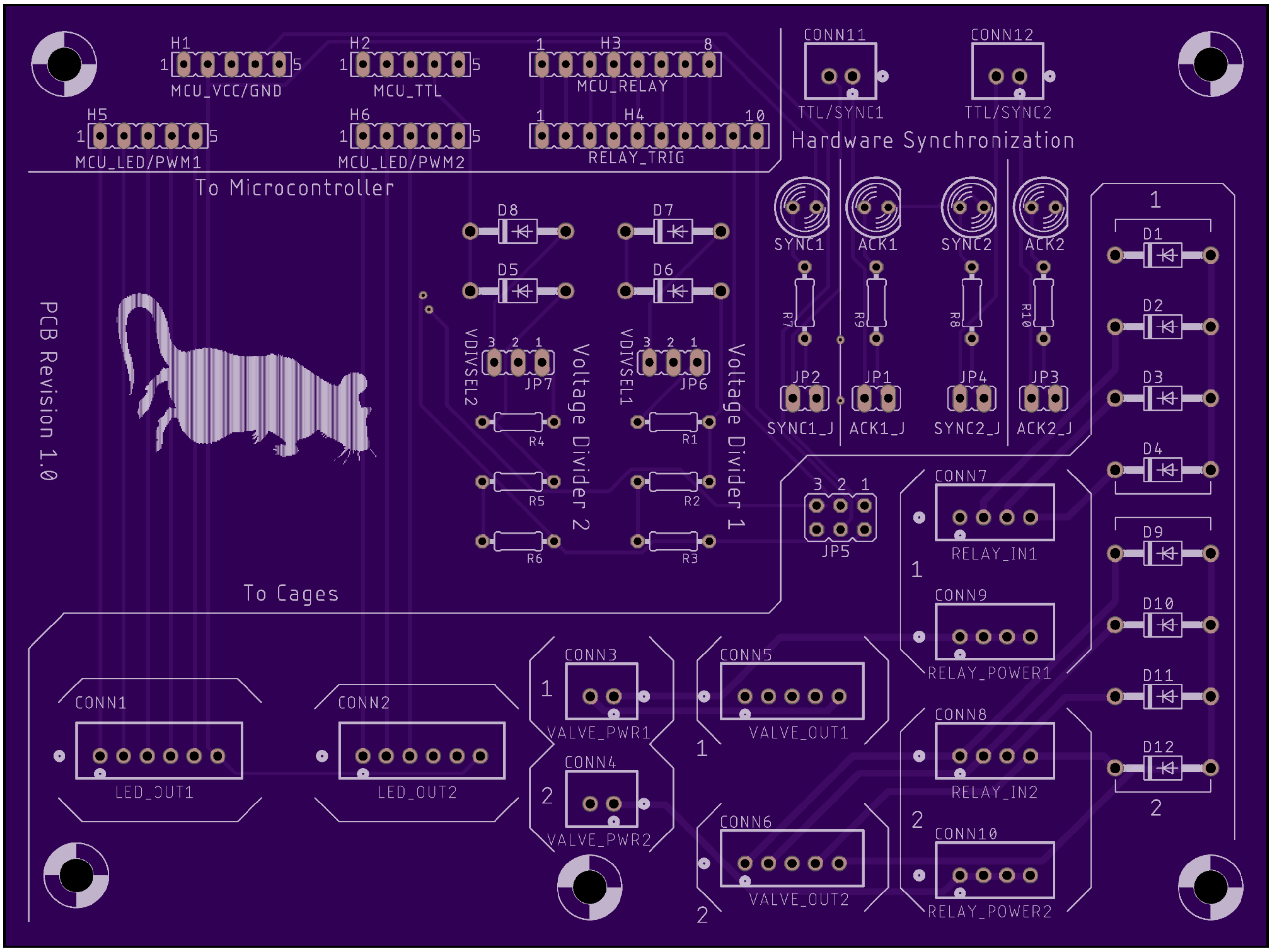

Custom RECORD PCB, Revision 1.0

PCB schematics

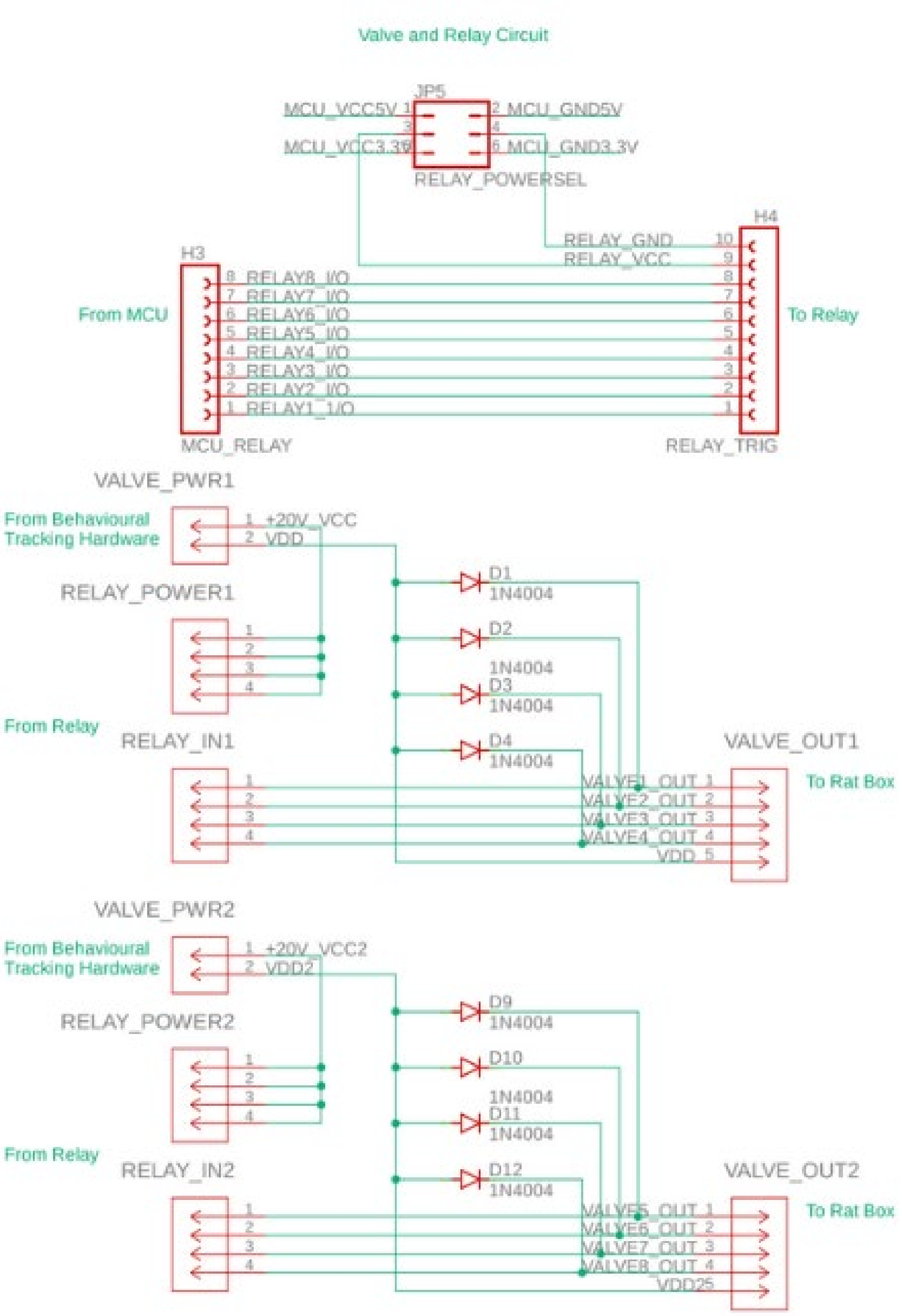

Custom RECORD PCB schematic, generated in Autodesk Eagle, PCB Revision 1.0

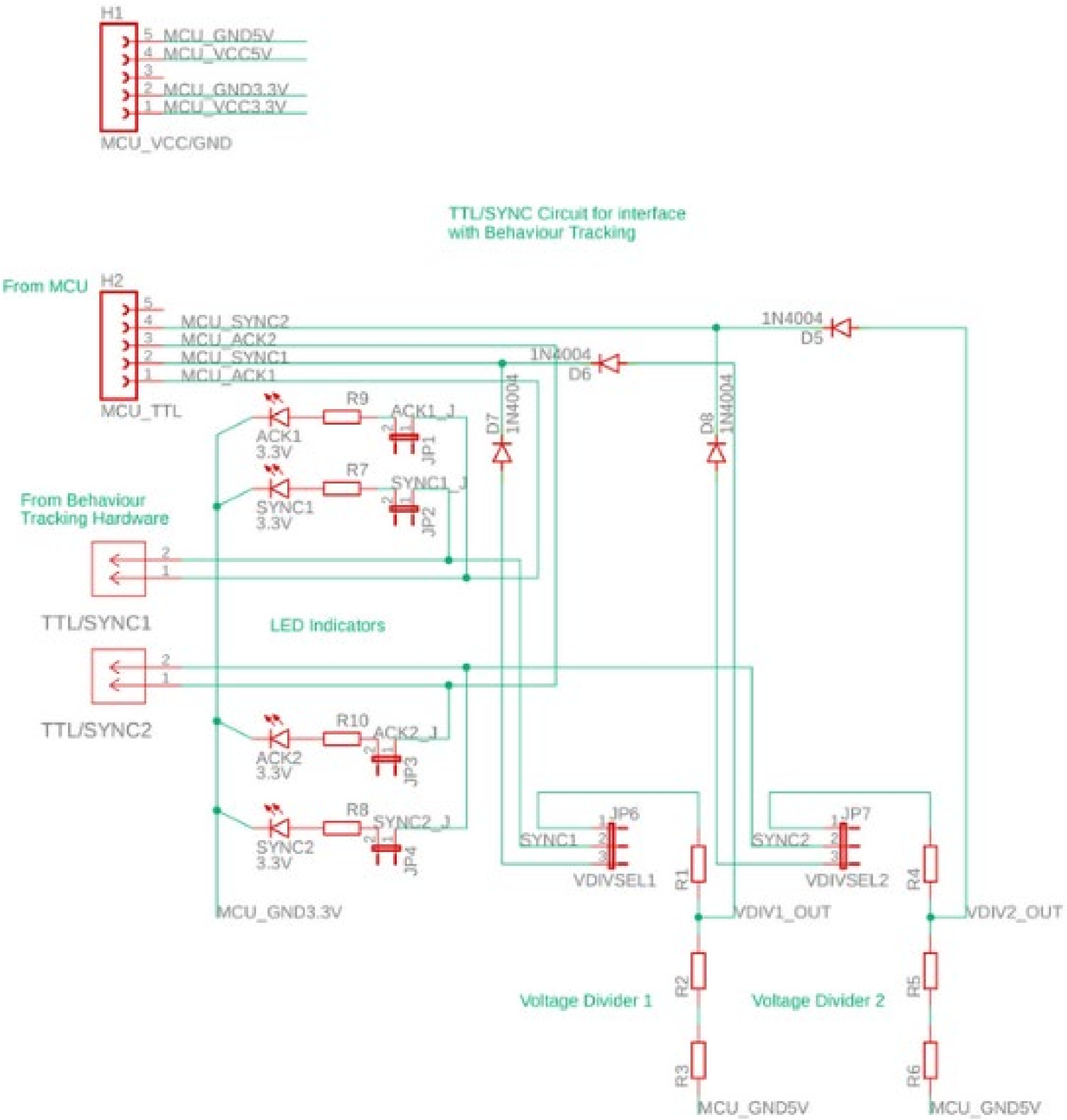

Custom RECORD PCB schematic, generated in Autodesk Eagle, PCB Revision 1.0

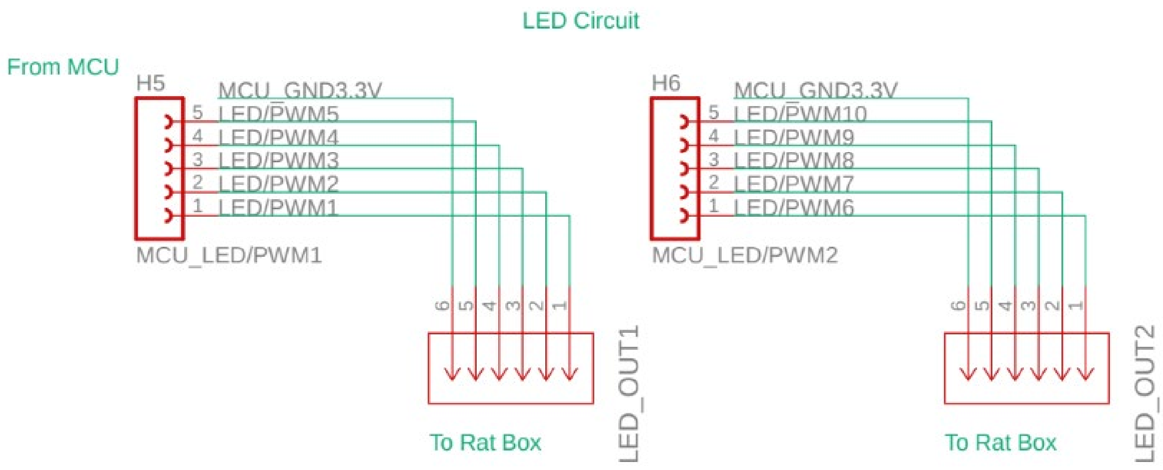

Custom RECORD PCB schematic, generated in Autodesk Eagle, PCB Revision 1.0

List of components

PCB Position	Manufacturer	Part Number	Value	Units	Component type
R1*	Sparkfun	COM-10969	2.2*<br>± 5	kΩ<br>%	Resistor
R2*	Sparkfun	COM-10969	3.3*<br>± 5	kΩ<br>%	Resistor
R3*	Sparkfun	COM-10969	0*	Ω	Resistor
R4*	Sparkfun	COM-10969	2.2*<br>± 5	kΩ<br>%	Resistor
R5*	Sparkfun	COM-10969	3.3*<br>± 5	kΩ<br>%	Resistor
R6*	Sparkfun	COM-10969	0*	Ω	Resistor
R7*	Sparkfun	COM-10969	0*	Ω	Resistor
R8*	Sparkfun	COM-10969	0*	Ω	Resistor
R9*	Sparkfun	COM-10969	0*	Ω	Resistor
R10*	Sparkfun	COM-10969	0*	Ω	Resistor
D1	Vishay General Semiconductors	1N4007E-E3/54	V_f = 1.1<br>V_r = 1000	V	Diode
D2	Vishay General Semiconductors	1N4007E-E3/54	V_f = 1.1<br>V_r = 1000	V	Diode
D3	Vishay General Semiconductors	1N4007E-E3/54	V_f = 1.1<br>V_r = 1000	V	Diode
D4	Vishay General Semiconductors	1N4007E-E3/54	V_f = 1.1<br>V_r = 1000	V	Diode
D5	Vishay General Semiconducto	1N4007E-E3/54	V_f = 1.1<br>V_r = 1000	V	Diode

	rs				
D6	Vishay General Semiconductors	1N4007E-E3/54	V_f = 1.1<br>V_r = 1000	V	Diode
D7	Vishay General Semiconductors	1N4007E-E3/54	V_f = 1.1<br>V_r = 1000	V	Diode
D8	Vishay General Semiconductors	1N4007E-E3/54	V_f = 1.1<br>V_r = 1000	V	Diode
D10	Vishay General Semiconductors	1N4007E-E3/54	V_f = 1.1<br>V_r = 1000	V	Diode
D11	Vishay General Semiconductors	1N4007E-E3/54	V_f = 1.1<br>V_r = 1000	V	Diode
D12	Vishay General Semiconductors	1N4007E-E3/54	V_f = 1.1<br>V_r = 1000	V	Diode
H1	Samtec Inc	TSW-105-07-T-S	Pitch = 2.54<br>Position = 5	mm	Board to Board Header
H2	Samtec Inc	TSW-105-07-T-S	Pitch = 2.54<br>Position = 5	mm	Board to Board Header
H3	Samtec Inc	TSW-108-07-T-S	Pitch = 2.54<br>Position = 8	mm	Board to Board Header
H4	Samtec Inc	TSW-110-07-T-S	Pitch = 2.54<br>Position = 10	mm	Board to Board Header
H5	Samtec Inc	TSW-105-07-T-S	Pitch = 2.54<br>Position = 5	mm	Board to Board Header

H6	Samtec Inc	TSW-105-07-T-S	Pitch = 2.54<br>Position = 5	mm	Board to Board Header
JP1	Samtec Inc	TSW-102-07-T-S	Pitch = 2.54<br>Position = 2	mm	Board to Board Header
JP2	Samtec Inc	TSW-102-07-T-S	Pitch = 2.54<br>Position = 2	mm	Board to Board Header
JP3	Samtec Inc	TSW-102-07-T-S	Pitch = 2.54<br>Position = 2	mm	Board to Board Header
JP4	Samtec Inc	TSW-102-07-T-S	Pitch = 2.54<br>Position = 2	mm	Board to Board Header
JP5	Samtec Inc	TSW-103-07-T-S	Pitch = 2.54<br>Position = 3<br>Rows = 2	mm	Board to Board Header
JP6	Samtec Inc	TSW-103-07-T-S	Pitch = 2.54<br>Position = 3	mm	Board to Board Header
JP7	Samtec Inc	TSW-103-07-T-S	Pitch = 2.54<br>Position = 3	mm	Board to Board Header
CONN1	Qibaok	XH-6A	Pitch = 2.54<br>Female<br>Position = 6	mm	JST-XH Female Header
CONN2	Qibaok	XH-6A	Pitch = 2.54<br>Female<br>Position = 6	mm	JST-XH Female Header
CONN3	Qibaok	XH-2A	Pitch = 2.54<br>Female<br>Position = 2	mm	JST-XH Female Header
CONN4	Qibaok	XH-2A	Pitch = 2.54<br>Female<br>Position = 2	mm	JST-XH Female Header
CONN5	Qibaok	XH-5A	Pitch = 2.54<br>Female<br>Position = 5	mm	JST-XH Female Header

CONN6	Qibaok	XH-5A	Pitch = 2.54<br>Female<br>Position = 5	mm	JST-XH Female Header
CONN7	Qibaok	XH-4A	Pitch = 2.54<br>Female<br>Position = 4	mm	JST-XH Female Header
CONN8	Qibaok	XH-4A	Pitch = 2.54<br>Female<br>Position = 4	mm	JST-XH Female Header
CONN9	Qibaok	XH-4A	Pitch = 2.54<br>Female<br>Position = 4	mm	JST-XH Female Header
CONN10	Qibaok	XH-4A	Pitch = 2.54<br>Female<br>Position = 4	mm	JST-XH Female Header
CONN11	Qibaok	XH-2A	Pitch = 2.54<br>Female<br>Position = 2	mm	JST-XH Female Header
CONN12	Qibaok	XH-2A	Pitch = 2.54<br>Female<br>Position = 2	mm	JST-XH Female Header
SYNC1*	Kingbright Company LLC	WP7113VBC/D	V_f = 3.3**<br>Lens = 5	V<br>mm	Light Emitting Diode (LED)
ACK1*	Kingbright Company LLC	WP7113VBC/D	V_f = 3.3**<br>Lens = 5	V<br>mm	Light Emitting Diode (LED)
SYNC2*	Kingbright Company LLC	WP7113VBC/D	V_f = 3.3**<br>Lens = 5	V<br>mm	Light Emitting Diode (LED)
ACK2*	Kingbright Company LLC	WP7113VBC/D	V_f = 3.3**<br>Lens = 5	V<br>mm	Light Emitting Diode (LED)

* Components and values reflect what was used for our particular setup. These Values should be chosen according to each system's requirements
** Different LED forward voltages may be needed depending on each individual system. The LED should be able to turn on with its corresponding TTL signal voltages delivered by the microcontroller and voltage divider output. Voltage divider output is dependent on R1, R2, and R3 for SYNC1 and ACK1, as well as R4, R5, and R6 for SYNC2 and ACK2. Value shown is the

typical operating voltage for the LED, see component datasheet for maximum and minimum forward voltages.

Internal Connectors:
Pictured in the image below (bottom right), they serve to connect the PCB to the relay shield input/outputs.

CONN7	Qibaok	XH-4Y	Pitch = 2.54 Male Position = 4	mm	JST-XH Male Connector
CONN8	Qibaok	XH-4Y	Pitch = 2.54 Male Position = 4	mm	JST-XH Male Connector
CONN9	Qibaok	XH-4Y	Pitch = 2.54 Male Position = 4	mm	JST-XH Male Connector
CONN10	Qibaok	XH-4Y	Pitch = 2.54 Male Position = 4	mm	JST-XH Male Connector

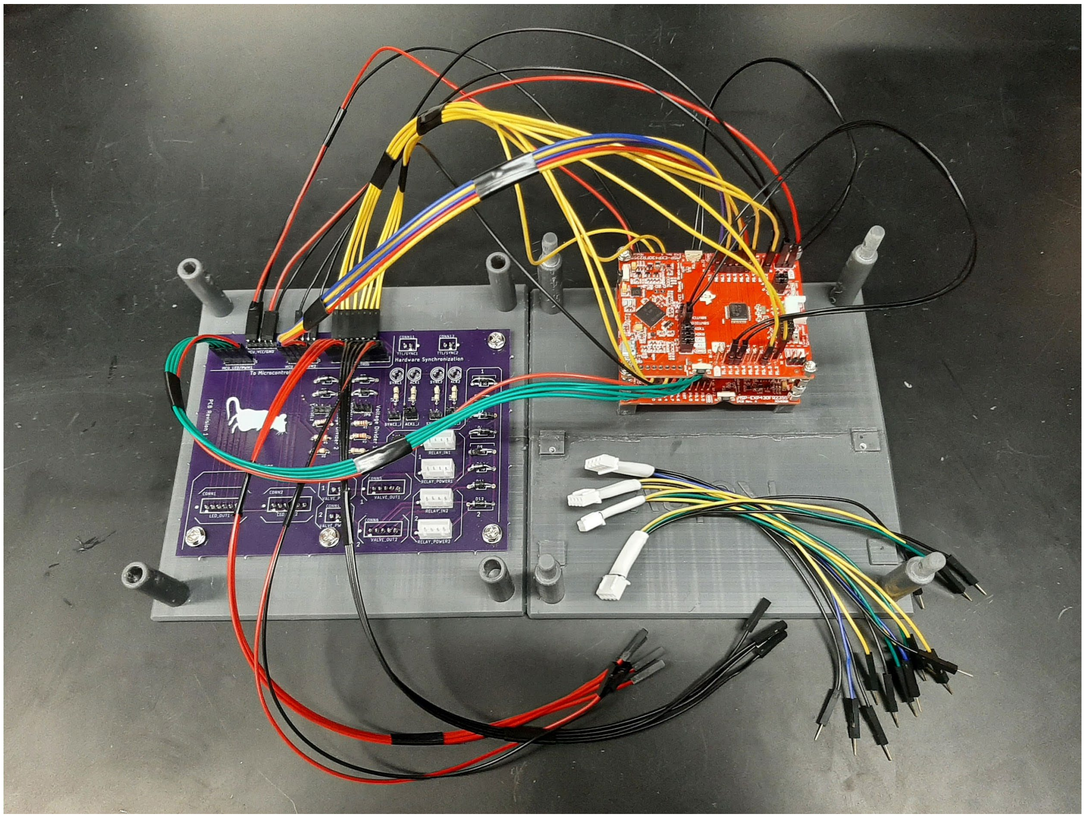

Microcontroller, Relay, and PCB Connections

Connections for MCU 1

**Signal**	**Position on MCU (MSP430-FR2355)**	**Position on PCB**	**Position on Relay Shield**
Cost LED1	P6.0	H5.1	-
Cost LED2	P6.1	H5.2	-
Cost LED3	P6.2	H5.3	-
Cost LED4	P6.3	H5.4	-
Trial Indicator LED	P6.4	H5.5	-
Low-Voltage Supply*	3V3 or 5V*	H1.1	-
Ground	GND	H1.2	-
SYNC	P3.5 or P3.6***	H2.1	-
ACK	P3.0***	H2.2	-
Trigger Signal for Relay 1	P3.1	H3.1	-
Trigger Signal for Relay 2	P3.2	H3.2	-
Trigger Signal for Relay 3	P3.7	H3.3	-
Trigger Signal for Relay 4	P3.4	H3.4	-
MCU to Relay 1	-	H4.1	IN1
MCU to Relay 2	-	H4.2	IN2
MCU to Relay 3	-	H4.3	IN3
MCU to Relay 4	-	H4.4	IN4
Relay VCC	-	H4.9	VCC
Relay GND	-	H4.10	GND
Relay 1 to Valve 1	-	CONN7.1	Relay 1, Normally Open Terminal

Relay 2 to Valve 2	-	CONN7.2	Relay 2, Normally Open Terminal
Relay 3 to Valve 3	-	CONN7.3	Relay 3, Normally Open Terminal
Relay 4 to Valve 4	-	CONN7.4	Relay 4, Normally Open Terminal
Common High-voltage supply for valves 1 - 4	-	CONN9.1-4**	Relay 1-4, COM Terminal

* Supplying low-voltage power to the PCB serves to route the voltage and ground to the relay shield. Make sure you are providing enough power to the relay shield and that the jumpers on JP5 are configured in the correct way to carry that voltage. The relay shield may be able to operate on 3.3V, however 5V is recommended.
** Connector 9 simply supplies voltage to the relay. The COM terminal is shorted with the Normally Open terminal when the relay is activated. The order in which the pins on the connector are connected to each relay's COM terminal has no effect on the function of the system.
*** This connection depends on the particular use-case scenario. P3.0 is the outgoing ACK signal that pulses every time a command is executed, P3.5 is an user-triggerable outgoing TTL signal, and P3.6 is for incoming TTL signals. Incoming TTL signals must be stepped down to 3.3V to not damage the system. Care must be taken when selecting what should be connected where.

Connections for MCU 2

Signal	Position on MCU	Position on PCB	Position on Relay Shield
Cost LED1	P6.0	H6.1	-
Cost LED2	P6.1	H6.2	-
Cost LED3	P6.2	H6.3	-
Cost LED4	P6.3	H6.4	-
Trial Indicator LED	P6.4	H6.5	-
Low-Voltage Supply*	3V3 or 5V*	H1.3	-
Ground	GND	H1.4	-
SYNC	P3.5 or P3.6***	H2.4	-
ACK	P3.0***	H2.5	-
Trigger Signal for Relay 1	P3.1	H3.5	-
Trigger Signal for Relay 2	P3.2	H3.6	-
Trigger Signal for Relay 3	P3.7	H3.7	-
Trigger Signal for Relay 4	P3.4	H3.8	-
MCU to Relay 1	-	H4.5	IN5
MCU to Relay 2	-	H4.6	IN6
MCU to Relay 3	-	H4.7	IN7
MCU to Relay 4	-	H4.8	IN8
Relay VCC	-	H4.9	VCC
Relay GND	-	H4.10	GND
Relay 5 to Valve 1	-	CONN8.1	Relay 5, Normally Open Terminal
Relay 6 to Valve 2	-	CONN8.2	Relay 6, Normally Open Terminal

Relay 7 to Valve 3	-	CONN8.3	Relay 7, Normally Open Terminal
Relay 8 to Valve 4	-	CONN8.4	Relay 8, Normally Open Terminal
Common High-voltage supply for valves 1 - 4	-	CONN10.1-4**	Relay 5-8, COM Terminal

* Supplying low-voltage power to the PCB serves to route the voltage and ground to the relay shield. Make sure you are providing enough power to the relay shield and that the jumpers on JP5 are configured in the correct way to carry that voltage. The relay shield may be able to operate on 3.3V, however 5V is recommended.
** Connector 10 simply supplies voltage to the relay. The COM terminal is shorted with the Normally Open terminal when the relay is activated. The order in which the pins on the connector are connected to each relay's COM terminal has no effect on the function of the system.
*** This connection depends on the particular use-case scenario. P3.0 is the outgoing ACK signal that pulses every time a command is executed, P3.5 is an user-triggerable outgoing TTL signal, and P3.6 is for incoming TTL signals. Incoming TTL signals must be stepped down to 3.3V to not damage the system.

Supplemental Note 3

R.E.C.O.R.D.

(Reward-Cost in Rodent Decision-making)

Arena setup guide

Revision 1.1

Introduction

This assembly guide will serve to help you create the 3D printed rodent decision-making arena. The arena is assembled utilising SLA 3D printing and custom electronics to allow for a custom-made task to be used in the study of decision-making in rodents. Such flexibility allows for on the fly changes and adaptations to the experimental environment and process, as well as animal behaviour. Further, it enables the interchangeability and replacement of maze arenas and parts as they wear down over the course of thousands of behavioural trials. In this guide you will find the parts and materials, dimensions and measurements, along with links and assembly instructions, to our custom maze arenas and electronic system(s).

Directly below is a link to our Github repository with finished versions of the CAD drawings we used to 3D print the behavioural open-field arena. Under the “stl” directory, you will find STL files for each arena component, ready to print after download. The “cad” directory contains .dwg CAD files of each of the arena components, more convenient for editing.

https://github.com/rjibanezalcala/RECORD/tree/main/3d-prints

Why 3D printed arenas?

We use 3D printing rather than store-bought or DIY because of the modularity and customizability it provides. We use 3D printing to create more customised platforms that can act as a simulated habitat for the animal subjects. The different individual components act as modifiable building blocks that can be reorganised to different shapes and sizes to accommodate for different test subjects such as either rats or mice. The arenas can also be built in different sizes to provide further freedom in customization. Our 3D-printed feeder is a custom design to fit the purpose of human-translatable cost and reward delivery, while also taking advantage of natural behaviours like foraging and threat avoidance. The corners of the maze are designed as a baseplate to hold any kind of variation on the cost/reward delivery system, for example, one may design an air puff nozzle to deliver cost instead. One may also choose to add more than 4 feeders to an arena for more resolution of the decision-making “range”.

The RECORD open-field arena

Materials

Below is a suggested list of materials for a square RECORD arena. We include the materials needed for a smaller 4 x 4 mouse arena, but the rest of this guide assumes an 8 x 8 rat arena.

Component	Quantity for rat arena	Quantity for mouse arena	Approx. resin required per piece	Source
**3D print**				
Diagonal floor tile	15	3	40 mL per piece	https://github.com/rjibanezalcala/RECORD/blob/main/3d-prints/stl/Diagonal%20Floor%20Tile.stl
Grid floor tile	15	3	40 mL per piece	https://github.com/rjibanezalcala/RECORD/blob/main/3d-prints/stl/Grid%20Floor%20Tile.stl
Horizontal floor tile	15	3	40 mL per piece	https://github.com/rjibanezalcala/RECORD/blob/main/3d-prints/stl/Horizontal%20Floor%20Tile.stl
Radial floor tile	15	3	40 mL per piece	https://github.com/rjibanezalcala/RECORD/blob/main/3d-prints/stl/Radial%20Floor%20Tile.stl
Wall support pilar	12	8	62 mL per piece	https://github.com/rjibanezalcala/RECORD/blob/main/3d-prints/stl/Wall%20Support%20Pillar%20v2.0.stl
Basic pillar	65	17	50 mL per piece	https://github.com/rjibanezalcala/RECORD/blob/main/3d-

				prints/stl/Basic%20Pillar%20v2.1.stl
Feeder base tile	4	4	20 mL per piece	https://github.com/rjibanezalcala/RECORD/blob/main/3d-prints/stl/Feeder%20Base%20Tile.stl
Feeder	4	4	25.5 mL per piece	https://github.com/rjibanezalcala/RECORD/blob/main/3d-prints/stl/Feeder%20v1.9.9.stl
**Other materials**				
Formlabs Form 3/3+ SLA printer *	-	-	-	https://formlabs.com/3d-printers/catalog/
Formlabs Tough 2000 resin *	-	-	-	https://formlabs.com/materials/tough-durable/
Superglue	-	-	-	Local hardware store
Acrylic PVC sheet (opaque white) 48" x 24" x 0.125"	4	4	-	https://www.grainger.com/product/GRAINGER-APPROVED-Plastic-Sheet-0-125-in-Plastic-44ZT07
Tools for cutting PVC sheet	-	-	-	Local hardware store
Spray paint primer Rust-Oleum 2x ULTRAcover primer **	-	-	-	Local hardware store

Neon green spray paint **	-	-	-	Local hardware store

*If desired, one may print the floor tiles using FDM printing instead, however, it is recommended that SLA printing is used for at least the feeders, as these must be water-tight and require precision. Type of resin is not critical for the floor pieces as they do not bear much weight, but the wall support pillars may also require a tougher material for support of the arena walls.
**Only important if recording in with colour video. If recording under infrared, the colour of the arena floor is not as important.

Wall support pillar

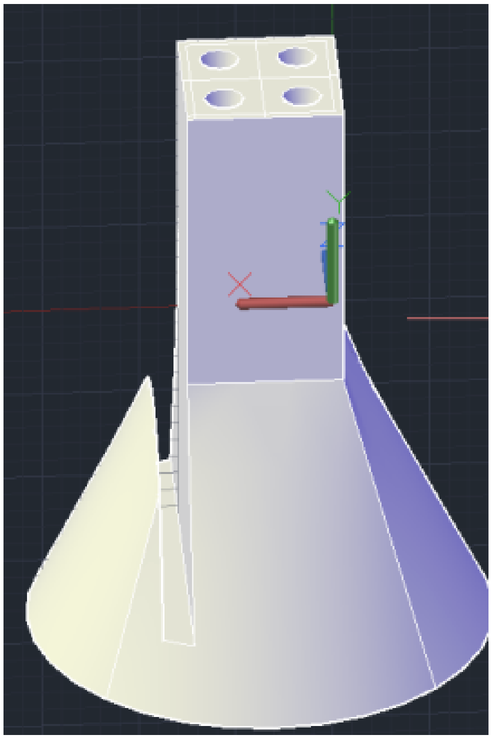

Basic pillar

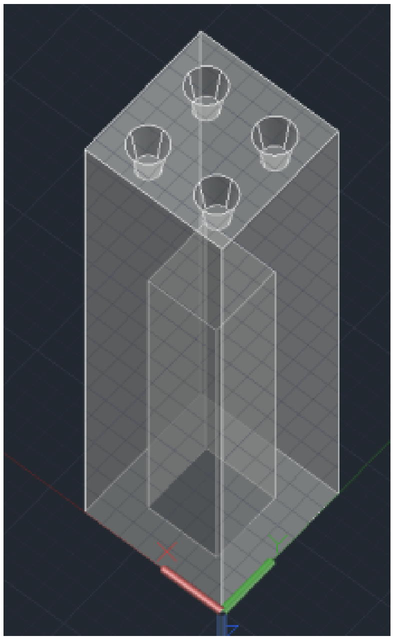

Feeder base tile

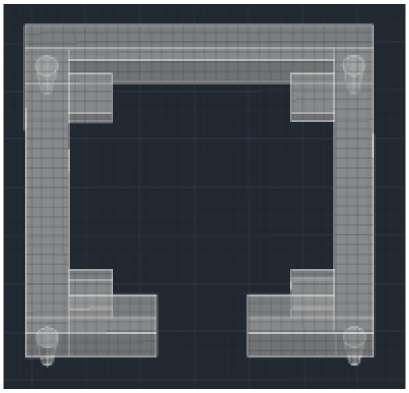

Grid floor tile

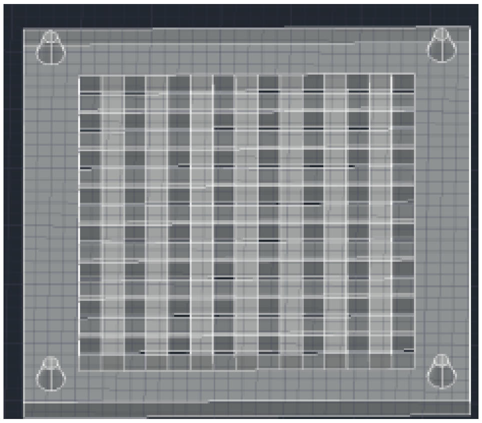

Horizontal floor tile

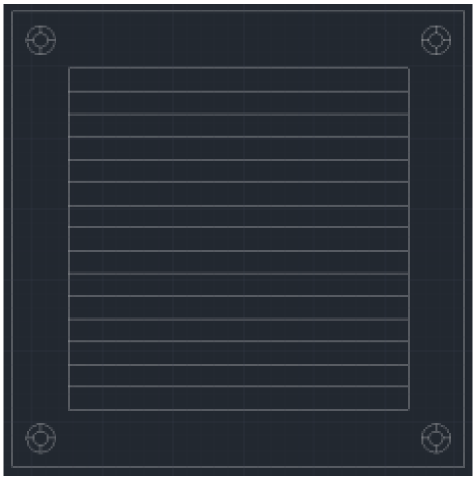

Diagonal floor tile

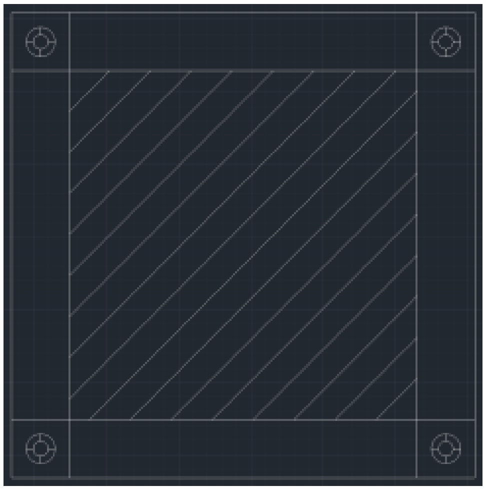

Radial floor tile

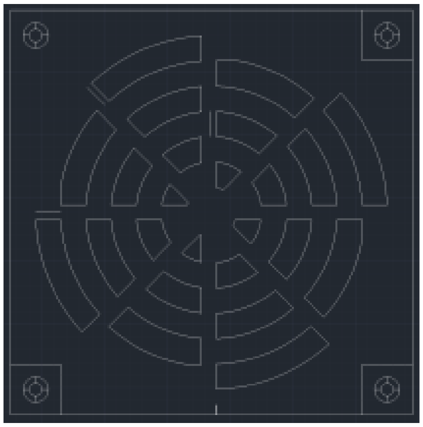

Feeder

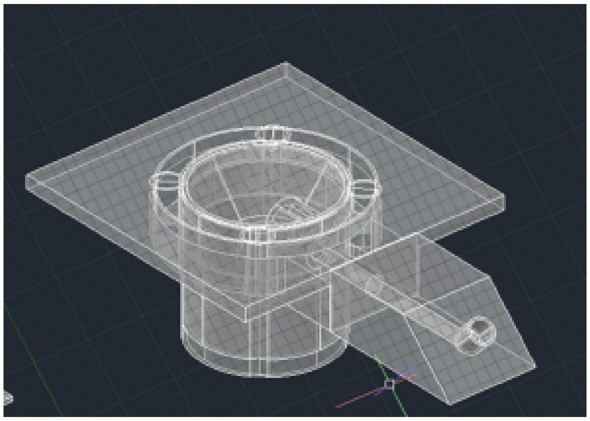

RECORD arena build guide

Step 1:

The arena floor was designed so it can be modified in scale to suit experimental needs.

Lay the floor tiles face-down (nubs should face up) on a large, flat surface. Place the tiles down so that the diagonal tile quadrant diagonally opposes the radial quadrant, and the grid quadrant opposes the horizontal quadrant. Place a feeder base tile on each corner, with the opening on each side facing the same direction. Each quadrant should be 4 tiles by 4 tiles.

Step 2:

Simple pillars are to be located wherever two or four tiles meet and at the corners. They should also be placed flush with the sides of the arena floor.

Each side of the arena floor should also have at least 2 (though we use three) wall support pillars to securely hold a wall on each side. Make sure that, on each side, the notches on the pillar base face out from the centre of the arena.

Doing so one quadrant at a time, super-glue the simple and support pillars to the underside of the arena. Use a flat, heavy object to press the components down while the glue cures and make sure the pillars do not tilt.

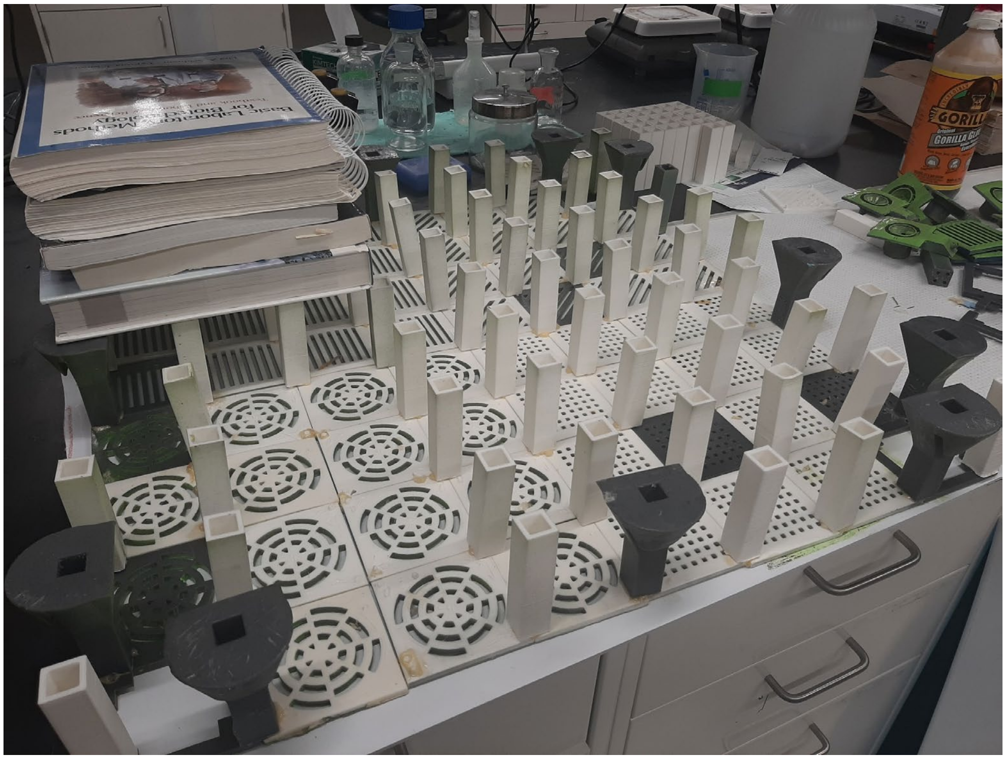

Step 3 (optional):

If behavioural recordings will be done in colour video, or if the printed arena has a glossy finish, you may choose to paint the RECORD arena a colour that contrasts that of the subjects. We chose a neon green colour as a general-purpose colour for any breed of rat.

Using a respirator and in a well-ventilated area, spray one or two coats of primer onto the arena floor and the feeders. When the final primer coat has dried, spray the neon green paint onto the arena until the arena floor is uniformly coated. Let both the primer and paint dry in-between coats. Optionally, you may add an additional clear coat on top of the paint, however it is important that the arena floor has a matte finish to avoid glare.

Step 4:

The arena walls serve to not only prevent the rodents from wandering off and away from the RECORD arena, but also to prevent the animal from getting too distracted by the test environment. Because of this, the PVC sheets should be non-reflective and a neutral, solid colour. However, this does not mean that this design cannot be modified to add a pattern on the wall.

Cut four acrylic PVC sheets to be about the length of 8 floor tiles or one side of the square arena (~64.5cm), and around 50 - 60 cm tall. Make sure that the walls fit snugly around the arena with little to no gap at the corners.

If the size of the sheets is satisfactory, pre-drill screw holes on either side of the sheet nearing the top of the sheet to add a corner bracket. This bracket will hold the sheets together to form a square.

Because the feeders need to jut out from the arena walls, measure and cut two slots on two of the sheets or wherever the open face of the feeder base tile faces. These slots must be as snug to the feeders as possible, while still allowing them to be lifted from the tail. We have provided approximate measurements in the figure below.

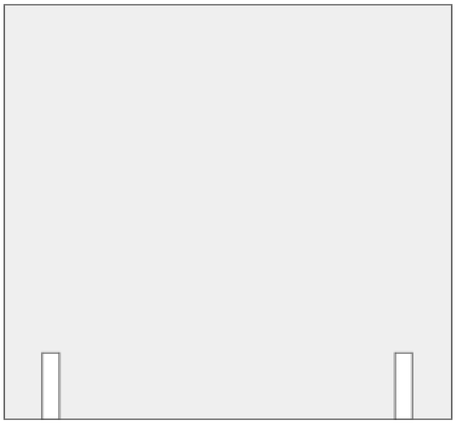

2x Walls with slots for feeders

Height: 75cm

Width: 64.5cm

(4x walls per 8x8 arena)

Slots are (8.25)cm tall and (2.3)cm wide. They are cut (3.3)cm from the corners/edges of the maze and are (54.61)cm apart from one another

Step 5:

Join the four walls with a corner bracket on each corner and secure them with screws. The wall assembly should then be ready to be lowered onto the nooks at the base of each wall support pillar.

Reward delivery assembly

Materials

The reward delivery assembly involves five components:

Component	Quantity per arena	Measurements	Source
Food-grade plastic tubing	~ 458 cm	3 mm I.D.	
Zero-pressure Solenoid valves	4	⅛” Female pipe thread, Normally closed, 0 psi minimum operating pressure differential	https://www.grainger.com/product/ASCO-Solenoid-Valve-1-8-in-Pipe-4ELA1?searchQuery=4ela1&searchBar=true&tier=Not+Applicable
Hose barb adapters	8	⅛” barb to ⅛” male NPT (National Pipe Thread)	https://www.amazon.com/Metalwork-Adapter-Barbed-Connector-Fitting/dp/B07ZBQ146L
Plastic syringe	4	60 cc	
Syringe tips	8	-	
Wide-base stand	~ 1 m tall	-	
Teflon tape	-	-	Local hardware store

The main purpose of the reward delivery assembly is to dispense liquid reward into the RECORD arena feeders. Solenoid valves allow us to electronically control how much liquid reward is dispensed by varying how long the valve remains open (see the RECORD user guide).

This part of the guide focuses on making only one reward delivery assembly. No more than one of these assemblies is needed per arena (unless more than four reward levels are implemented), and it is recommended that each is placed near its respective arena.

Reward delivery assembly build guide

Preparation:

Make sure to decide where in your setup the reward delivery assembly will be placed. Take note of the distance between the stand and each of the feeder corners, as this will be important for determining how much tubing you'll need.

Step 1:

Start by attaching two barb hose adapters to each end of the solenoid valve. If the adapter is not of the exact measurements, a pipe reducer can be used (see picture below). Wrap the thread of the adapters in Teflon tape to ensure that no liquid leaks through the joints.

Step 2:

Decide now which end will be the input (syringe to valve) and which will be the output (valve to feeder). Attach a shorter section of tubing (about 25 cm) to the valve input; this will be the tubing that will run from the liquid reward receptacle (syringe) to the valve. Next, attach a longer section of tubing to the output side of the valve. The output tubing should be long enough to extend from the valve to the feeder already installed onto an arena with some slack, so measure this before cutting the tubing.

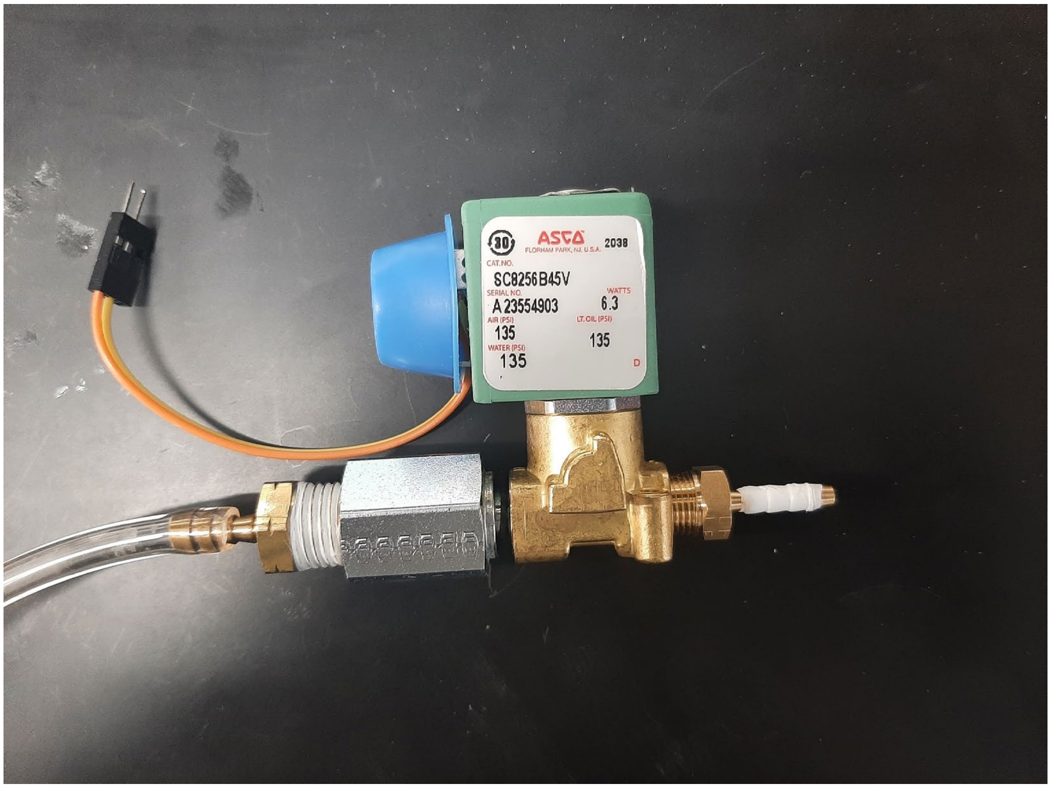

Step 3:

Remove the plunger from the syringe. Attach the other end of the input tubing to a syringe tip and install the syringe tip onto the syringe. You may use the syringe plunger to test if there are any leaks between these components. Keep the plunger as it may help get rid of bubbles or clogs in the system.

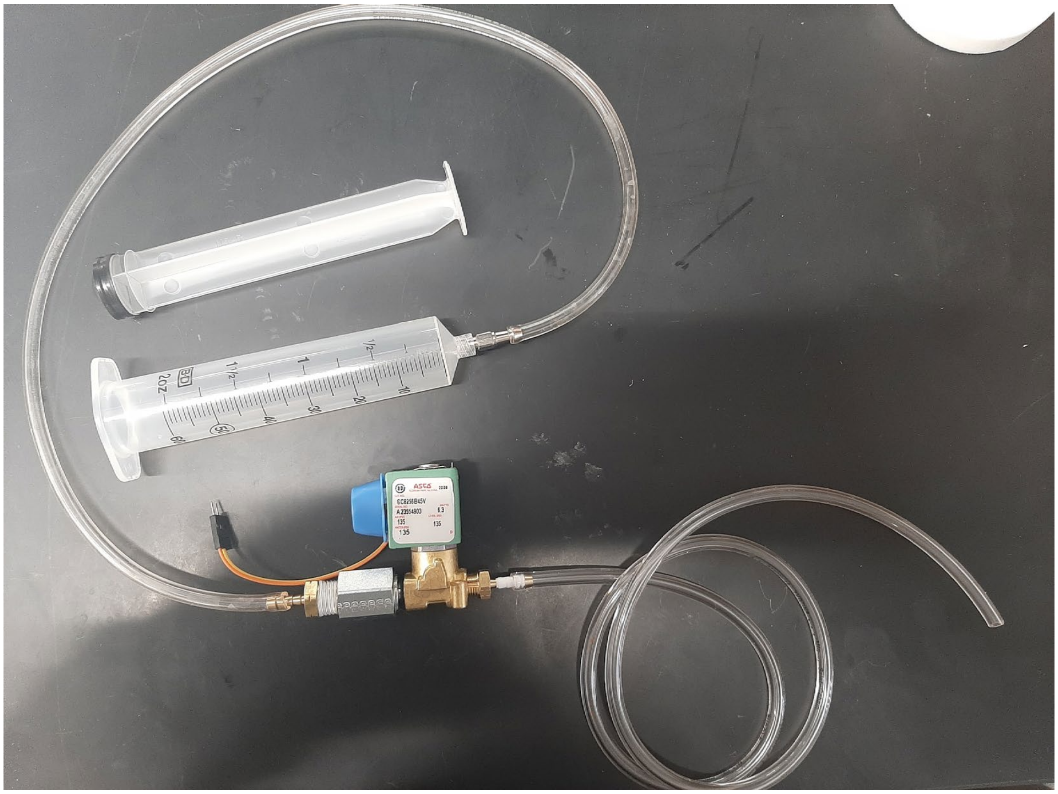

Step 4:

Repeat steps 1 through 3 for all four valves. Mount the four valve assemblies to the stand using braces or zip ties. All four syringes should be at around the same height, and the four valves should also be at around the same height. Keep in mind that the higher the syringes and valves are, the more pressure can build up in the system. Adjust the height of these components appropriately.

Step 5:

If you have not already, install all four feeders onto the arena. Attach the other end of the output tubing to the end of each of the feeders, keeping note and marking which valve and syringe corresponds to each feeder. Connect the RECORD valve cables to each of the valves. To test, fill the syringes with plain water and use the RECORD system with a serial console window (see RECORD user guide) to open the valves until the whole system is full of water and test if water can be delivered to the feeders.

If the valves are working but the water is not running through properly:
Make sure that the input tubing is properly inserted into the hose adapters and syringes. Also make sure that there isn't a large bubble in the system. Check if when opening the valves, the pockets of air move.

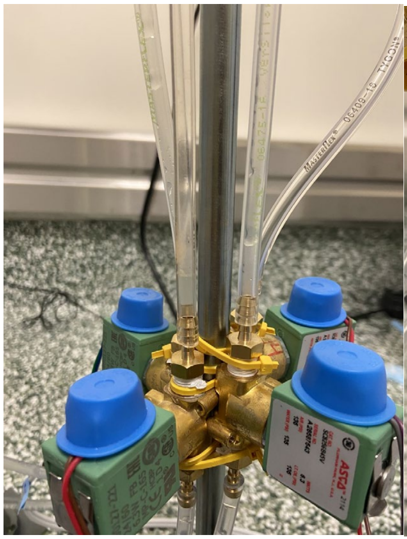
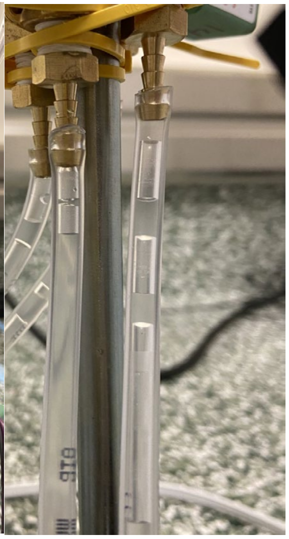

Camera setup

The camera setup enables spatial tracking of the rodents on the record arena. There exist other spatial tracking methods, such as lighthouse tracking, but many may follow the same procedure described below. Here, we provide materials and a general setup guideline to follow for recordings in low- to no-light conditions with gigabit cameras, as we have used in our validated RECORD setups.

Materials

Component	Quantity per arena	Specifications	Source
CMOS sensor with compatible lenses (Gigabit camera)	1	Gigabit, Infrared sensor *, 1280 px x 1024 px	https://www.baslerweb.com/en/products/cameras/area-scan-cameras/ace/aca1300-60gc/
PoE (power over ethernet) power supply for camera	1	-	https://www.amazon.com/Injector-Adapter-1000Mbps-802-3af-Compliant/dp/B07V24C4M8/
PCIe ethernet adapter	1	One or multiple ports, Gigabit ethernet	https://www.amazon.com/Dual-Port-Gigabit-Network-Express-Ethernet/dp/B09D3JL14S/
Extendable mounting autopole	< 1	Maximum length dependent on room size	https://www.manfrotto.com/us-en/autopole-black-extends-from-210cm-to-370cm-032b/
Autopole clamp	3	-	https://www.manfrotto.com/us-en/super-clamp-w-stud-035rl/
Camera software	1	-	https://www.baslerweb.com/en/downloads/software-downloads/software-pylon-7-3-0-windows/
Infrared (IR) illuminators *	2	-	https://axtontech.com/illuminators/ir-

			illuminator-smart-at-11s/

* Needed if recording in low-light or no-light conditions.

Animal spatial tracking guidelines

- The autopole should be placed directly above (but not necessarily centred with) the RECORD arena. This is where cameras and illuminators will be mounted onto.
- Place the autopole high enough so that it does not hinder access to the RECORD arena, but not so high that the camera cannot be adjusted to only have the arena floor in view.
- Mount the camera and IR illuminators onto their respective clamps before mounting onto the pole.
- Connect the cameras to your computer and open the camera preview to align the RECORD arena so that it is centred in the camera's view. The crosshair utility on the Pylon Viewer is especially helpful for this.

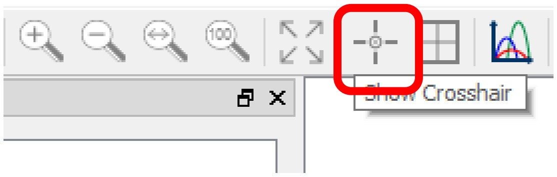

- If using spatial tracking software such as Ethovision XT, keep in mind that the arenas must always be in the same spot in order for the animal detection to work well. Otherwise, arena settings or detection settings will need to be adjusted every time.
- Use the Pylon Viewer software to adjust the resolution, image quality, colour, colour corrections, etc before starting any recordings. If using a Bonsai-driven setup, keep in mind you will need to export the camera features into a file and feed them into your Bonsai workflow.

Supplemental Note 4

R.E.C.O.R.D.

(Reward-Cost in Rodent Decision-making)

Setting up a remote PostgreSQL database

And the Serendipity App

Restoring A Database from backup

Assuming you have downloaded the backup database from Harvard Dataverse from the following link please follow the following steps to restore it. In case you have not yet downloaded the file it can be found in the link below inside the Database Backup folder which is viewable under tree view. Be sure to unzip the file before proceeding. Download the latest version available.
https://doi.org/10.7910/DVN/QADUKS
Run the following commands in the PSQL command line which should have been installed when you set up PostgreSQL on your local machine.

```
Server [localhost]:
Database [postgres]:
Port [5432]:
Username [postgres]:
Password for user postgres:
psql (16.0)
WARNING: Console code page (437) differs from Windows code page (1252)
         8-bit characters might not work correctly. See psql reference
         page "Notes for Windows users" for details.
Type "help" for help.

postgres=# \d
Did not find any relations.
postgres=# CREATE DATABASE live_database;
```

In your machine's Local Command Prompt run the following commands. Before running the commands ensure to add PostgreSQL 16's bin file to the command line path.

```
C:\Program Files\PostgreSQL\16>pg_restore -U postgres -d live_database C:\backupFile1.tar
Password:

C:\Program Files\PostgreSQL\16>
```

When prompted for password please enter the password you used when setting up PostgreSQL on your local machine.

Remote Database Folder (What's included?)

1.) App Deployment Folder (What's included?)

a. AdvanceSearchWindow (.mlapp file)

This app will conduct basic searches in the live_table.

b. HardCodeTable (.xlsx file)

This excel file is a representation app.info which is explained later in detail later in this paper.

c. InfoWeWant (.xlsx file)

This excel file is a list of variables we want the Serendipity app to keep track of.

d. Main (.mlapp file)

This app is the main page of the Serendipity app

e. postgresql-42.3.1 (.jar file)

This file is the jar file that the “Start.mlapp” and database connection both need to execute.

f. Start (.mlapp file)

IMPORTANT!!!!

This app is the start page for Serendipity. It is HIGHLY RECCOMMENDED that you leave this file in the App Deployment folder and simply create a shortcut to this file on your desktop. This can be accomplished by right clicking on “Start.mlapp” and clicking the “Create shortcut” option.

2.) backupProtocal (.bat file)

This program will automatically create backup files in the designated directory. You must specify the directory by editing the file.

3.) createDummyTable (.m file)

4.) createLiveTable (.m file)

5.) createRatTable (.m file)

6.) displayTables (.m file)

Introduction

The purpose of this paper is to give a detailed explanation of how to set up a remote PostgreSQL database that the Serendipity App can read from and write to. It will also provide a high-level explanation of how the Serendipity app works, but for a detailed explanation the

source code should be referenced. Together with the Serendipity App and a supporting remote database the Big-Data analysis can easily be performed on neatly sorted data.

A Windows computer should be used for this process for best results, but it could be recreated on other computers with some research on the user's part.

Things to Download Before Beginning

The following programs should be downloaded before beginning as they will all be used in the process of creating and setting up the remote database.

1. MATLAB
    a. https://www.mathworks.com/help/install/ug/install-products-with-internet-connection.html
2. MATLAB App Designer
    a. https://www.mathworks.com/products/matlab/app-designer.html
3. MATLAB database toolbox
    a. https://www.mathworks.com/products/database.html
4. PostgreSQL
    a. https://www.postgresql.org/download/
5. PostgreSQL JDBC Driver file
    a. https://jdbc.postgresql.org/download.html

Provided that everything was properly downloaded we can now begin configuring the computer to allow for remote access.

Configuring the Firewall

It is important to configure the firewall because without doing this your server will not allow for remote connections, and thus no data will be able to be uploaded making it impossible for the project to continue.

1. Open up the windows control panel, you can find this program by clicking on the windows start logo and searching “Control Panel”

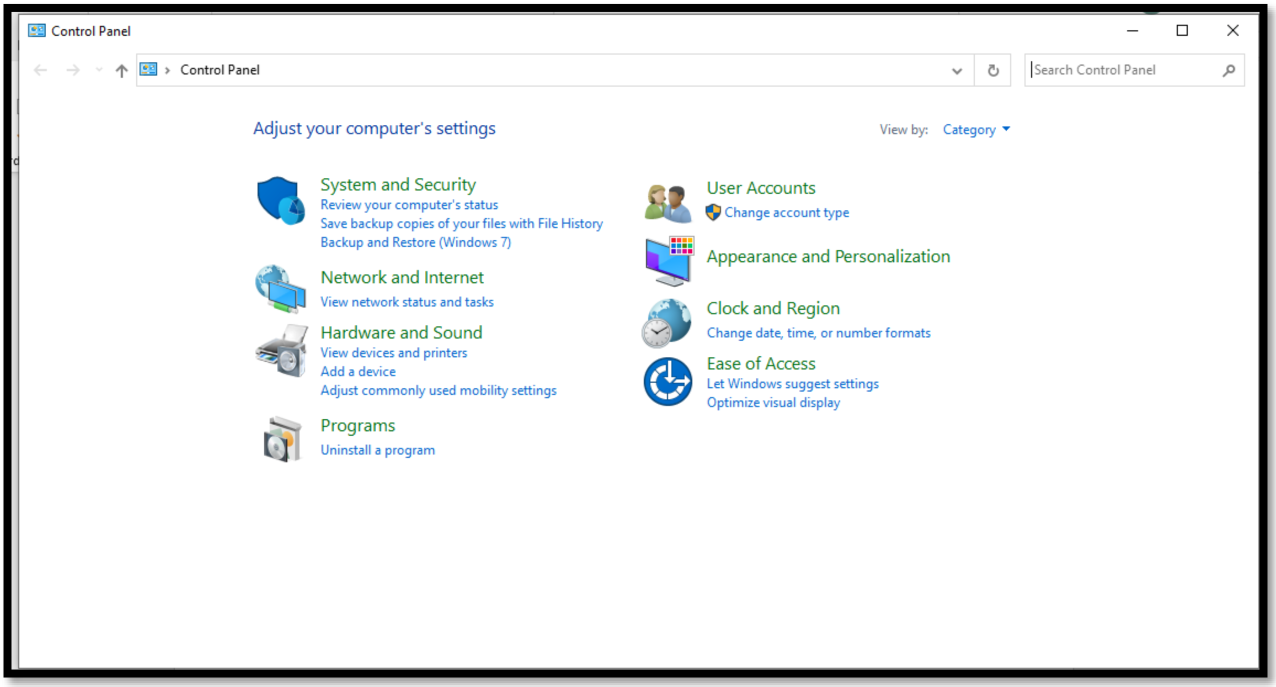

2. Select the “System and Security” option and arrive at the following screen

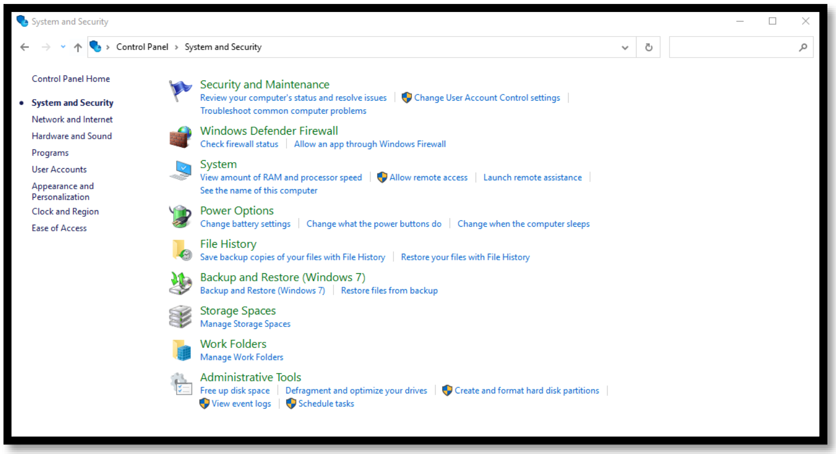

3. Select the “Windows Defender Firewall” Option and arrive at the following screen

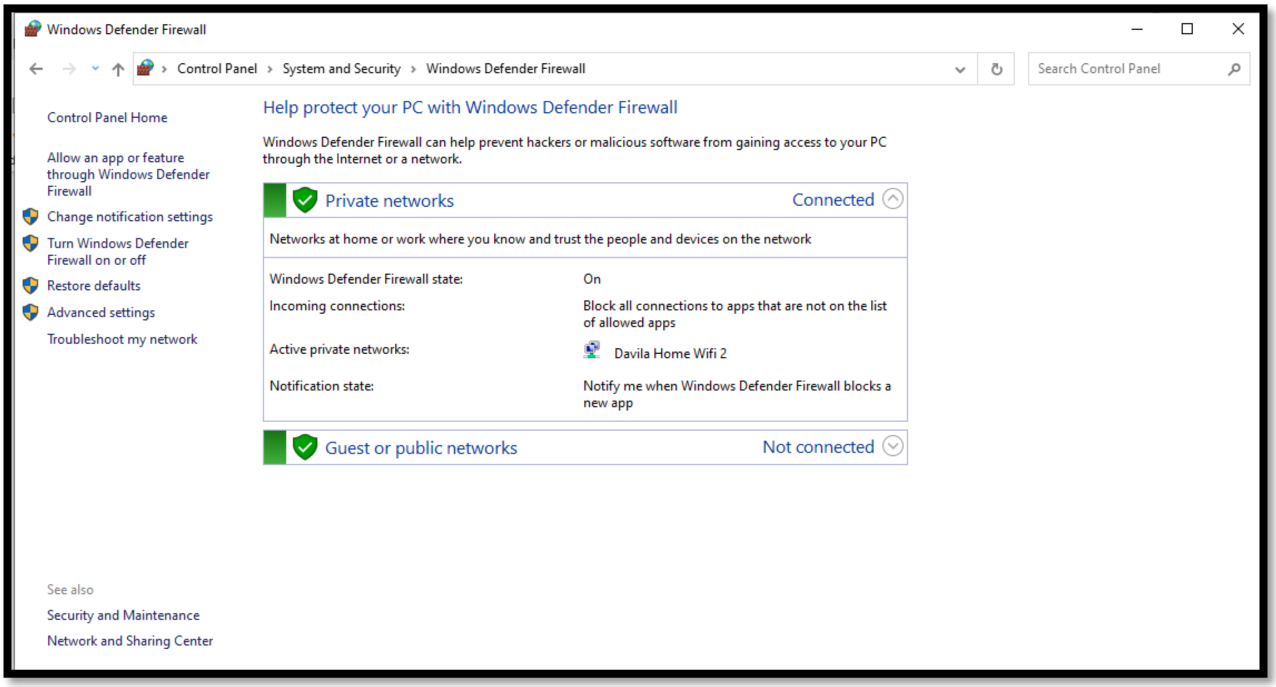

4. Click on Advanced Settings and arrive at the following screen.

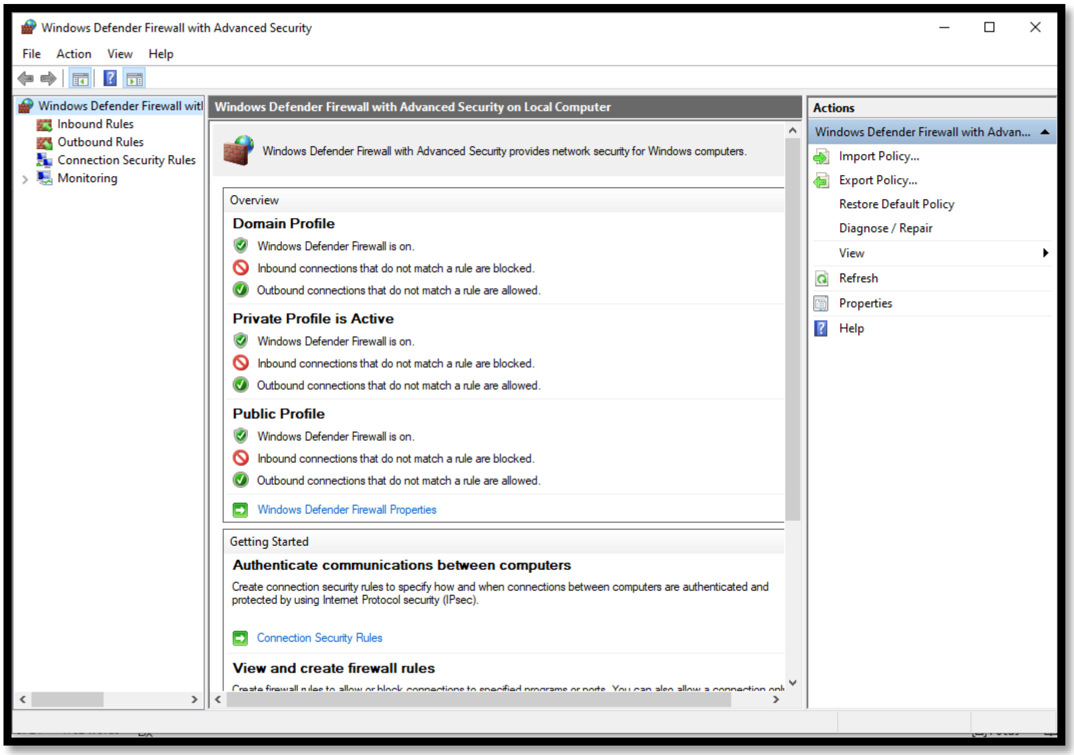

5. Click on “Inbound Rules” and arrive at the following screen. The listed rules might be different in your particular case, but this is no reason for case.

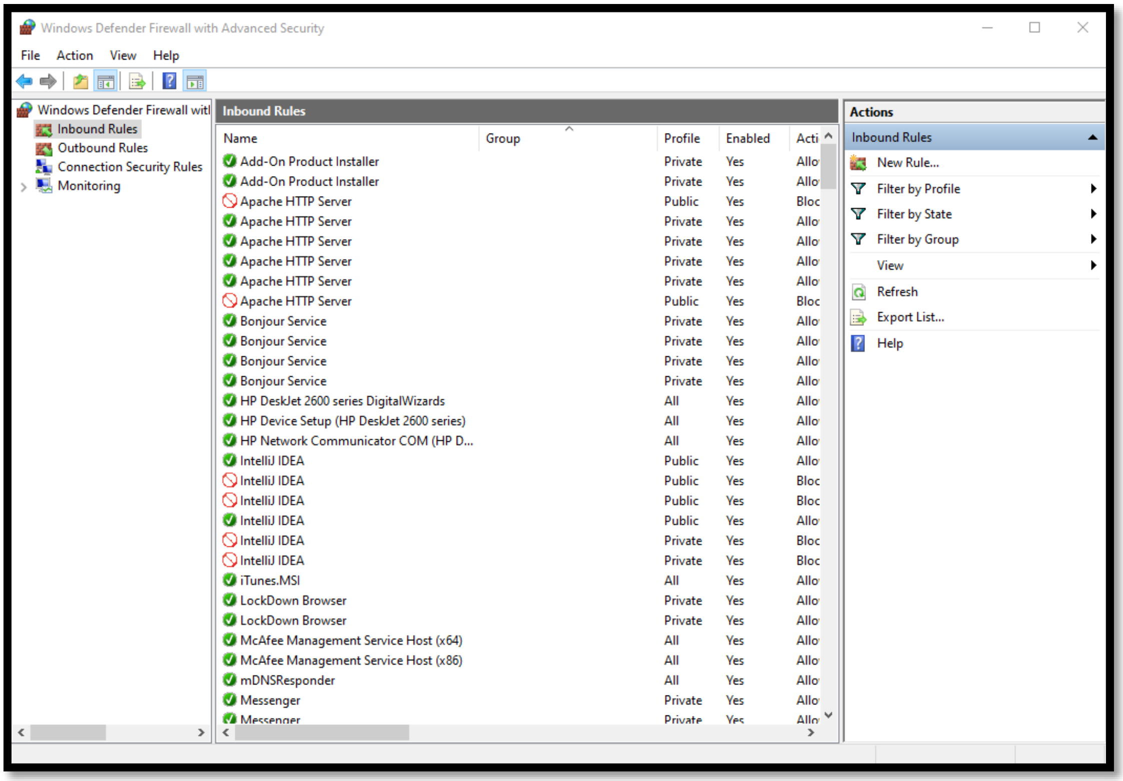
Windows Defender Firewall with Advanced Security
File Action View Help
Windows Defender Firewall witl
Inbound Rules
Outbound Rules
Connection Security Rules
Monitoring
Inbound Rules
Name
Group
Profile
Enabled
Acti
Add-On Product Installer Private Yes Allo
Add-On Product Installer Private Yes Allo
Apache HTTP Server Public Yes Bloc
Apache HTTP Server Private Yes Allo
Apache HTTP Server Private Yes Allo
Apache HTTP Server Private Yes Allo
Apache HTTP Server Private Yes Allo
Apache HTTP Server Public Yes Bloc
Bonjour Service Private Yes Allo
Bonjour Service Private Yes Allo
Bonjour Service Private Yes Allo
Bonjour Service Private Yes Allo
HP DeskJet 2600 series DigitalWizards All Yes Allo
HP Device Setup (HP DeskJet 2600 series) All Yes Allo
HP Network Communicator COM (HP D... All Yes Allo
IntelliJ IDEA Public Yes Allo
IntelliJ IDEA Public Yes Bloc
IntelliJ IDEA Public Yes Bloc
IntelliJ IDEA Public Yes Allo
IntelliJ IDEA Private Yes Bloc
IntelliJ IDEA Private Yes Bloc
iTunes.MSI All Yes Allo
LockDown Browser Private Yes Allo
LockDown Browser Private Yes Allo
McAfee Management Service Host (x64) All Yes Allo
McAfee Management Service Host (x86) All Yes Allo
mDNSResponder All Yes Allo
Messenger Private Yes Allo
Actions
Inbound Rules
New Rule...
Filter by Profile
Filter by State
Filter by Group
View
Refresh
Export List...
Help

6. Click on the “New Rule…” option found under the Actions tab on the right side, and arrive at the following screen

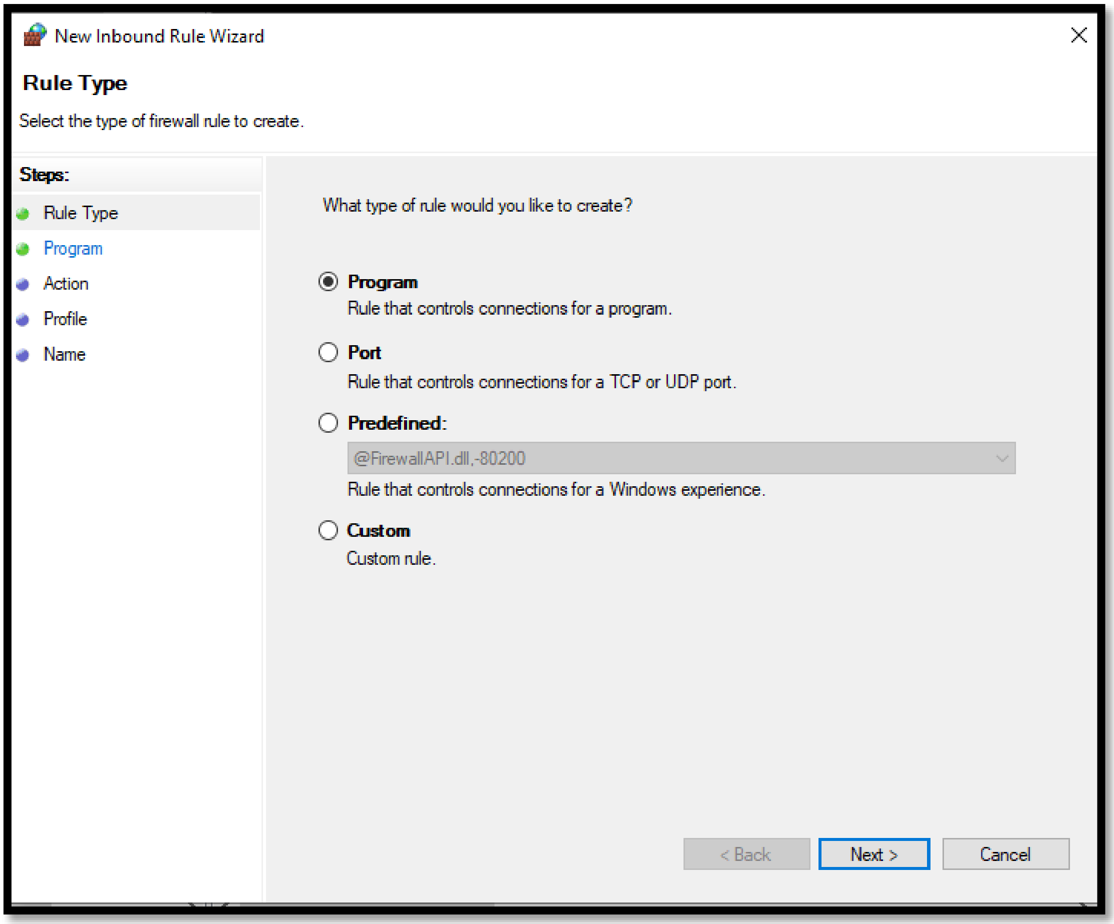

7. Select the “Port” bubble and click “Next”, arriving at the following screen.

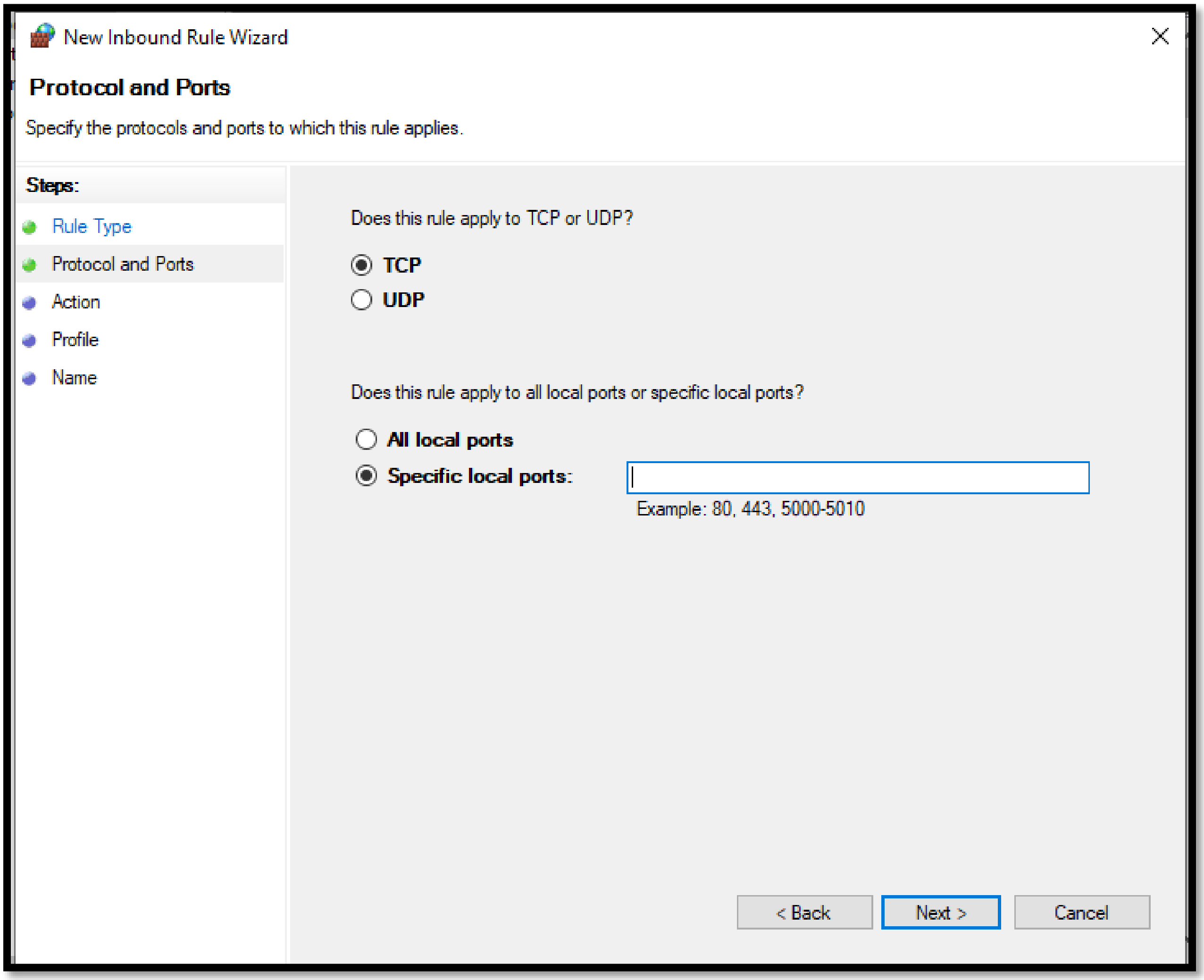

8. Click the “Specific local ports:” bubble and enter “5432” in the text box, which is the default port that PostgreSQL uses. This might change in the future as PostgreSQL releases new updates, PostgreSQL documentation should be referenced for this.

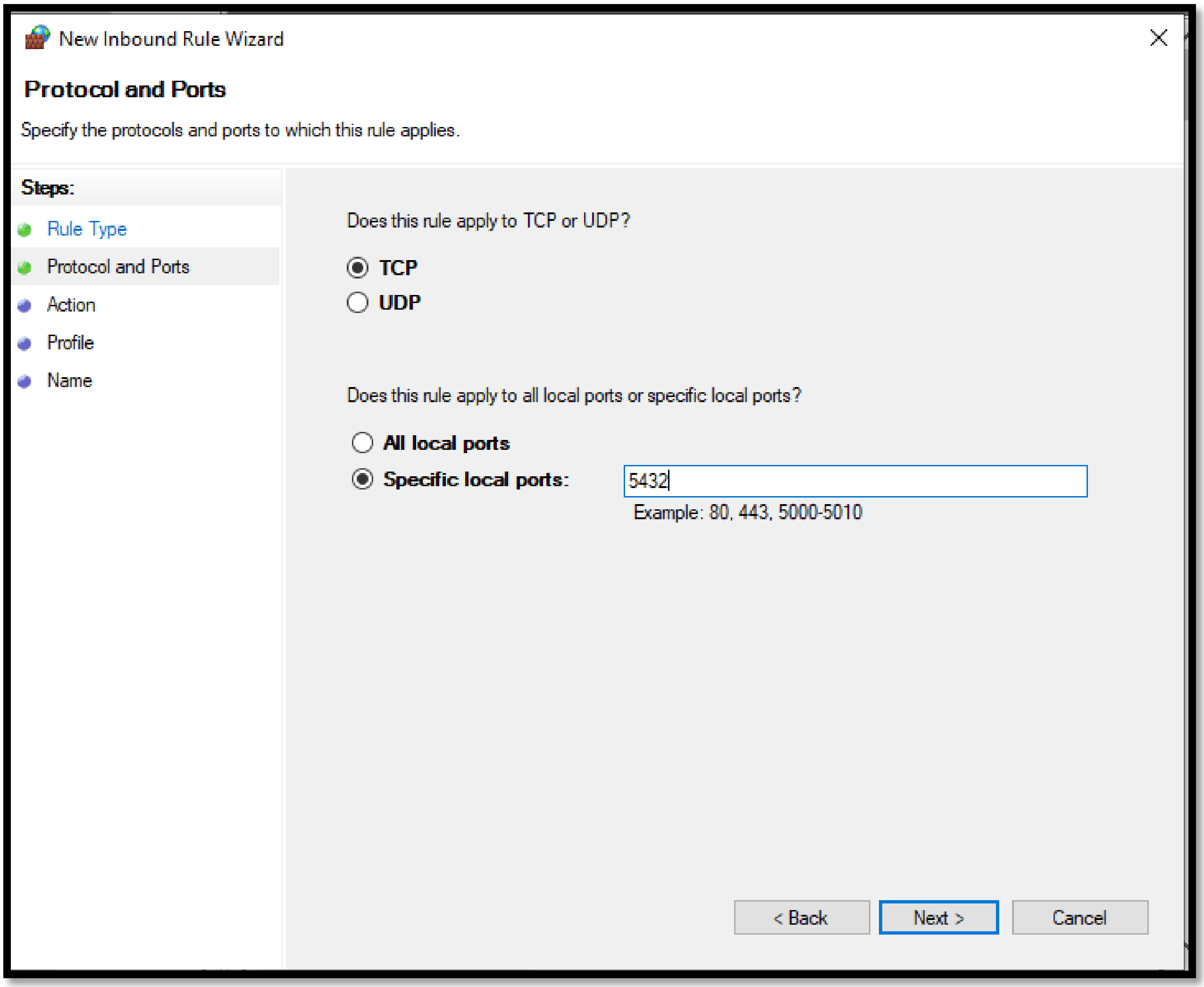

9. Click on next and arrive at the following page

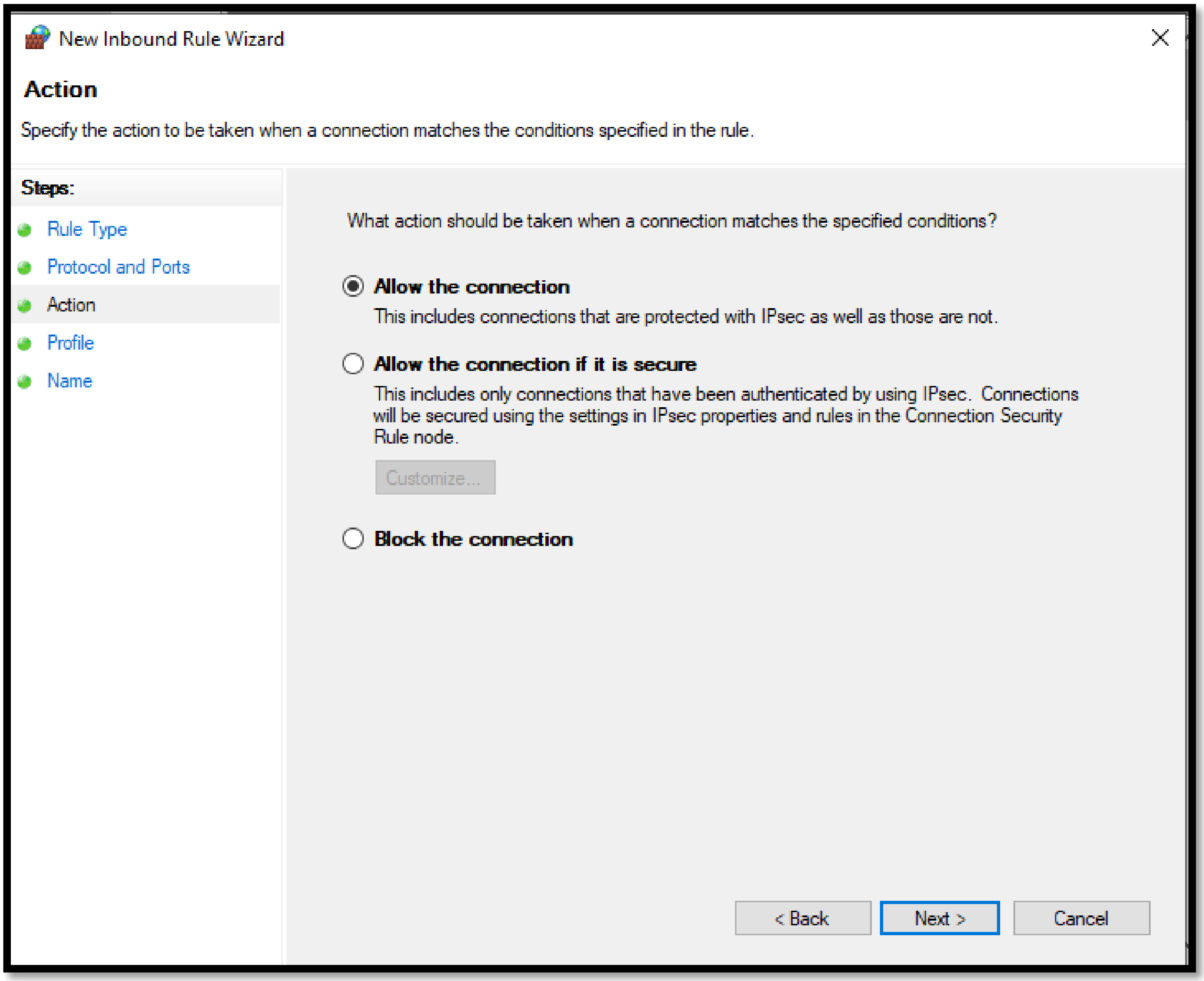

10. Click on the “Allow the connection” bubble and click next, arriving at the following screen.

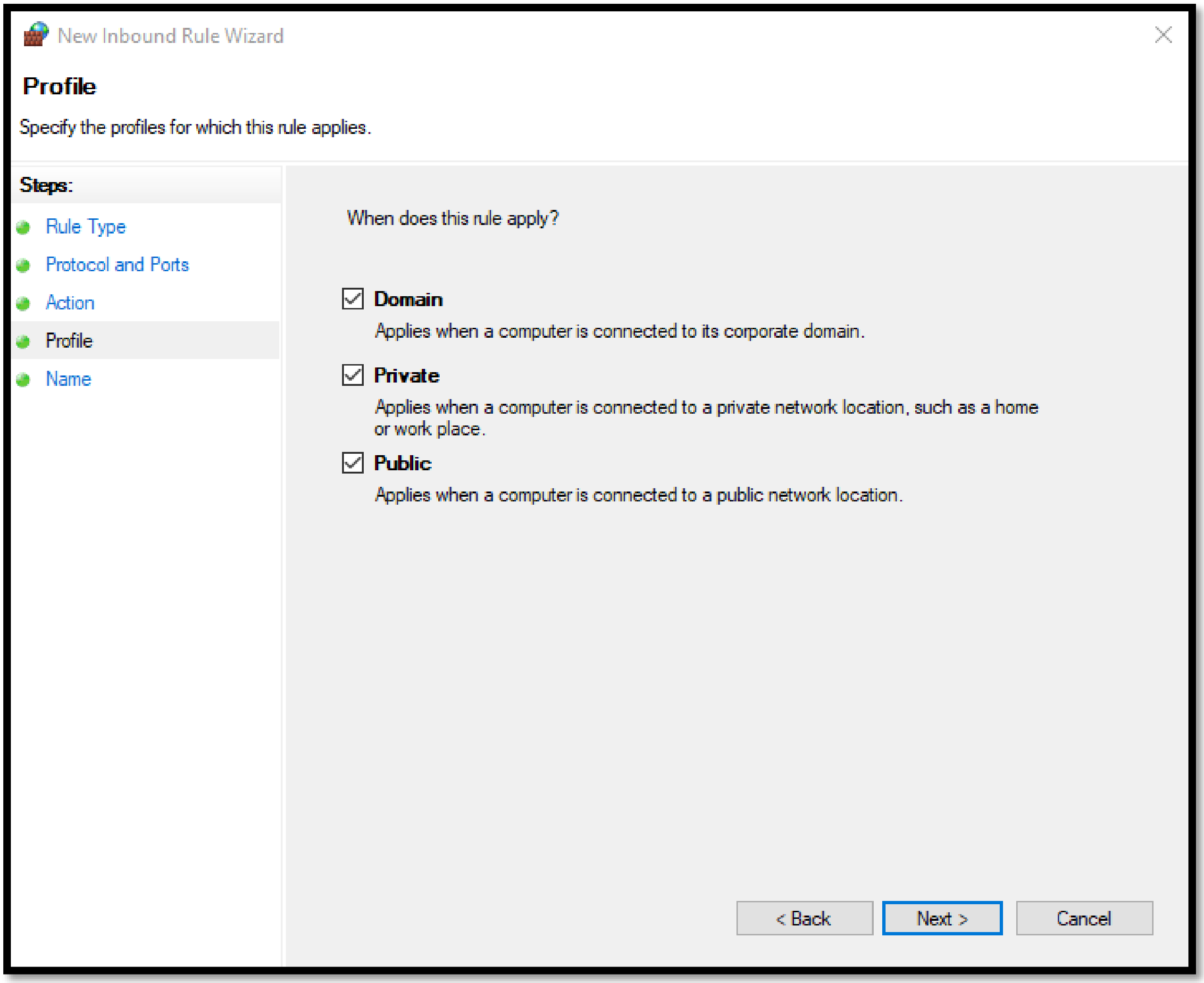

11. Click “Next” without modifying anything, and arrive at the following page

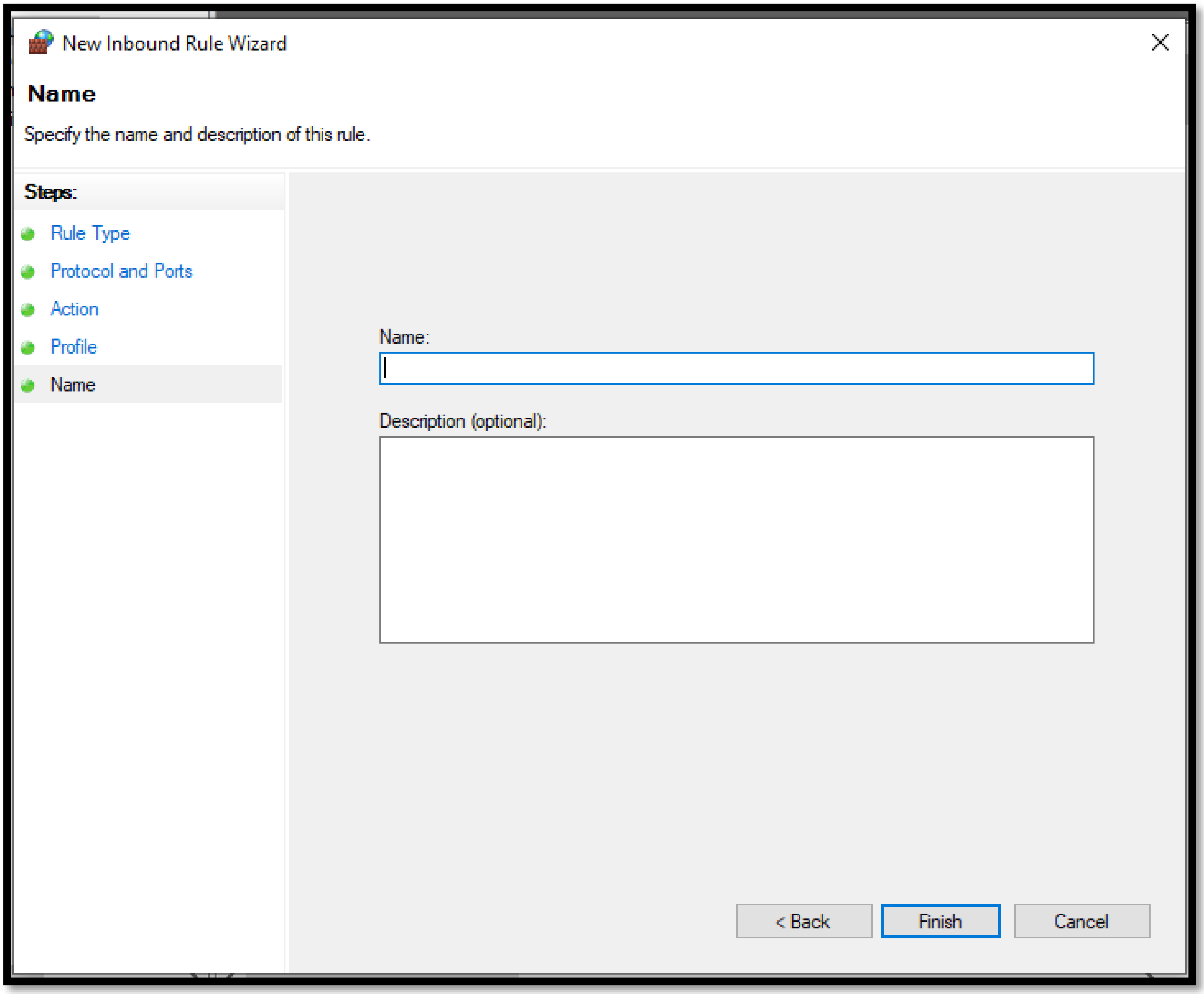

12. Fill “PostgreSQL Database Rule” in the “Name” field and enter “Rule that allows outside connections to the PostgreSQL database” in the “Description (optional):” field.

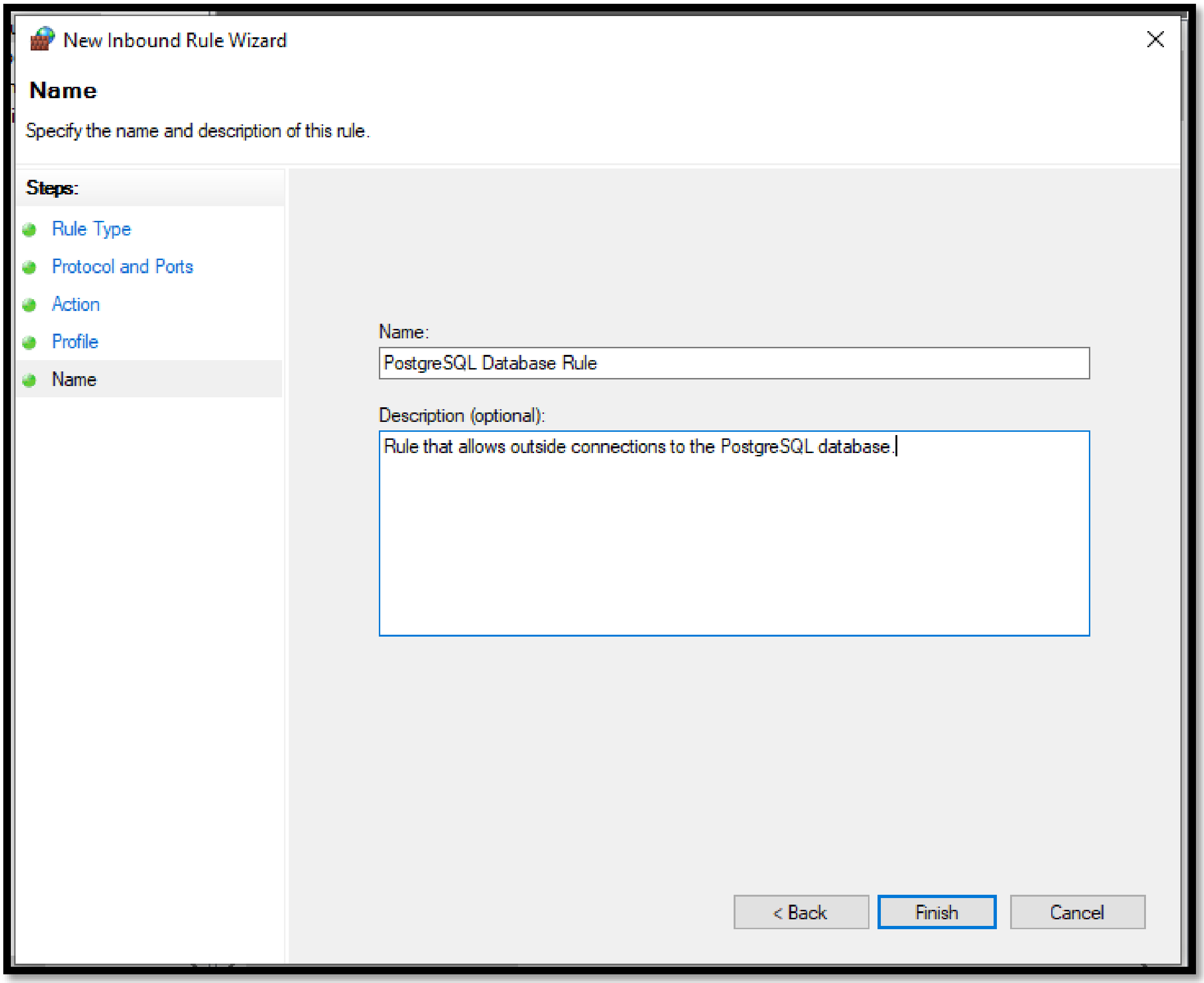

13. Click “Finish” and you’ll arrive at the following screen.

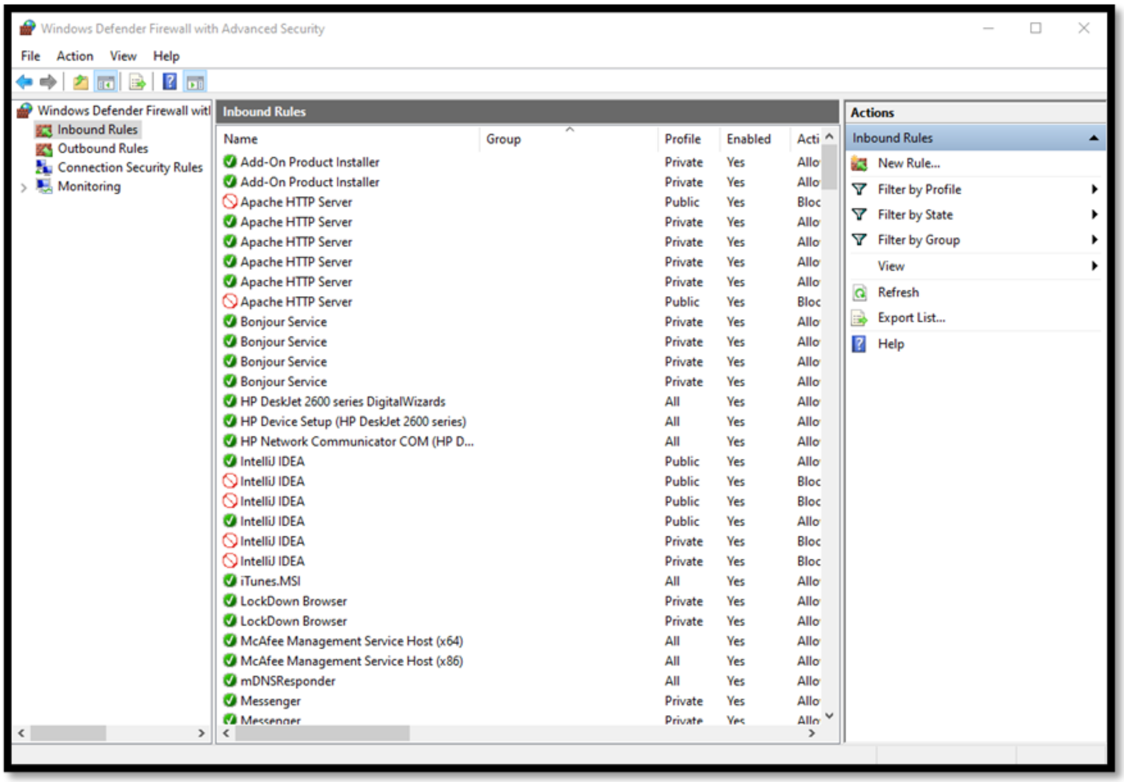

14. Your rule will now be listed under Inbound Rules and can be deleted, rewritten, or recreated using the previous steps.

Configuring PostgreSQL for Remote Access

It is import to configure PostgreSQL for Remote Access because by default it does not allow remote access. Without this configuration nothing will be able to be uploaded to the database, and the project cannot continue.

1. Do not begin this portion before downloading PostgreSQL to your computer, if you have not yet done that please refer to page 2
2. The following steps allow for the server you are creating to be accessed remotely, as by default PostgreSQL doesn't allow remote connections
3. Click on the windows start tab and search for "sql shell" which should have been downloaded to your computer when PostgreSQL was installed.
4. DO NOT left click on the "sql shell" application, instead right click on it and select the "open file location" option

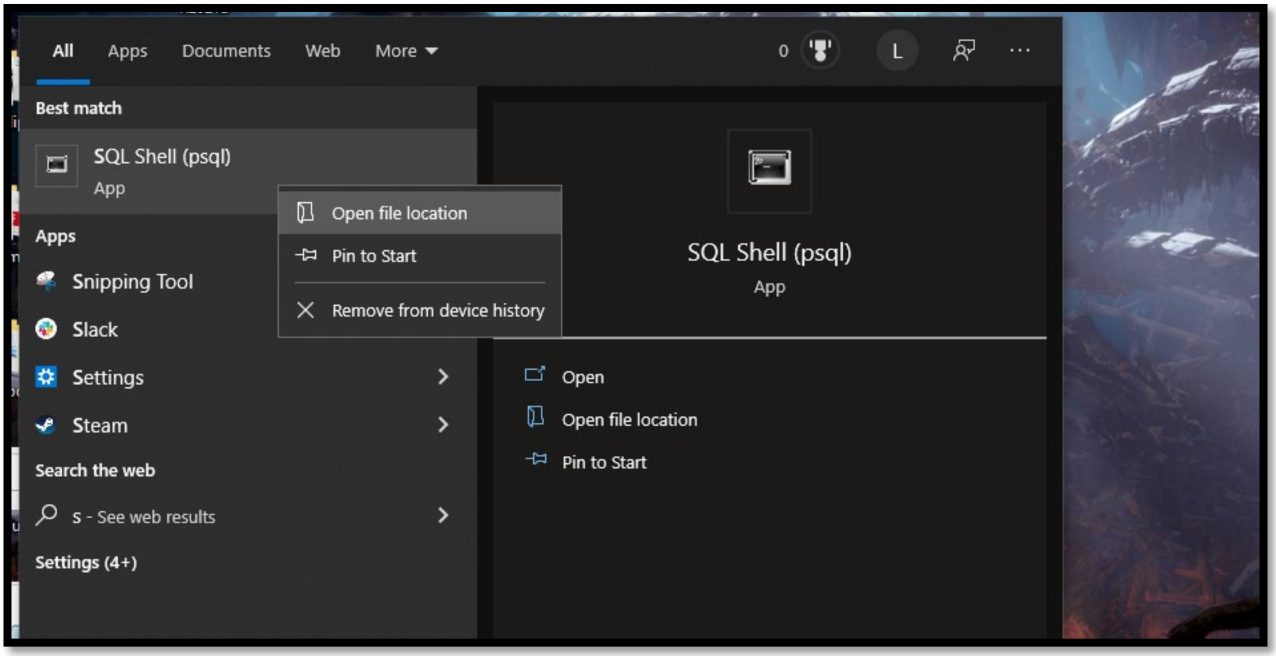

5. You'll arrive at a screen that looks like the following

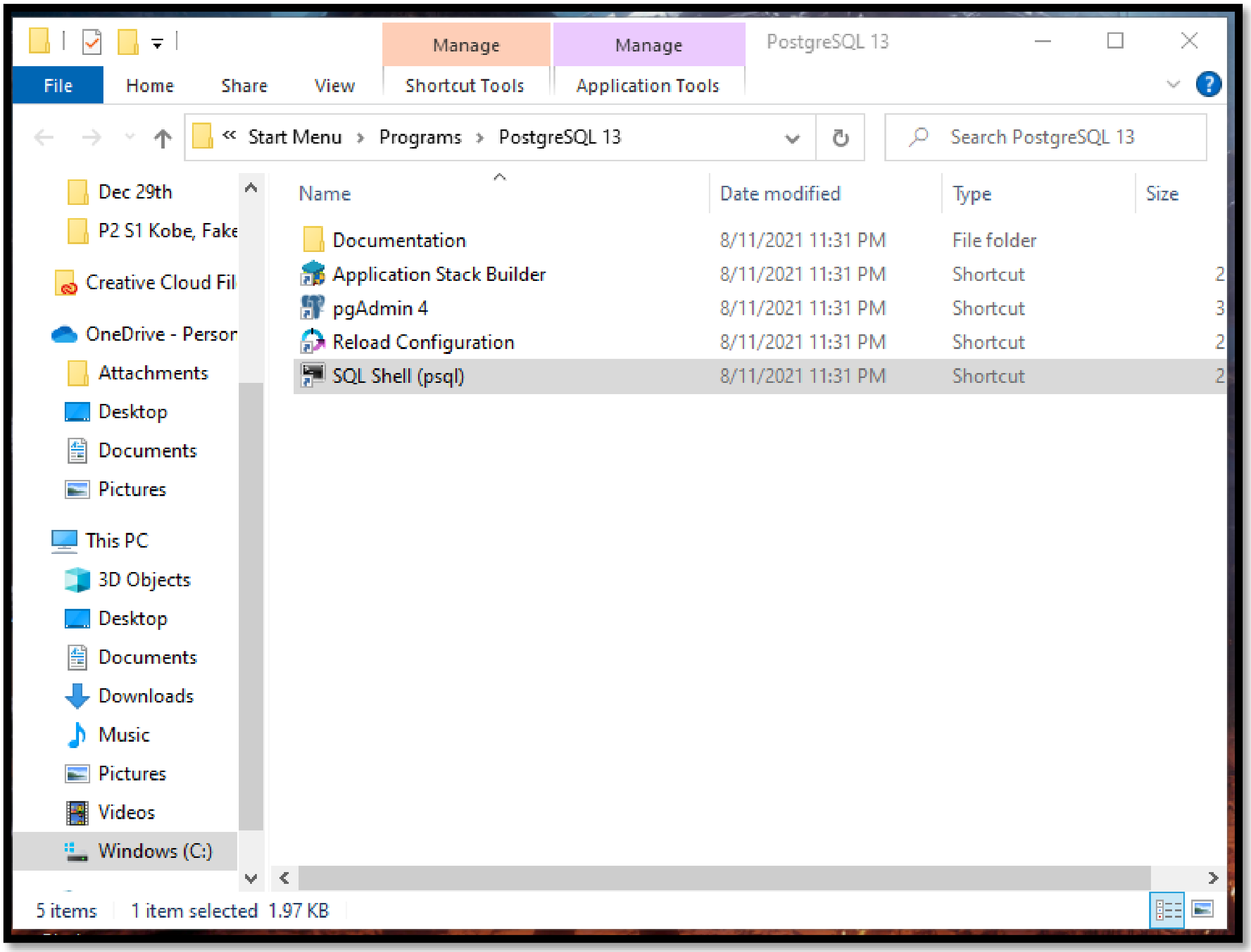

6. Once again right click on SQL shell and arrive at the next screen

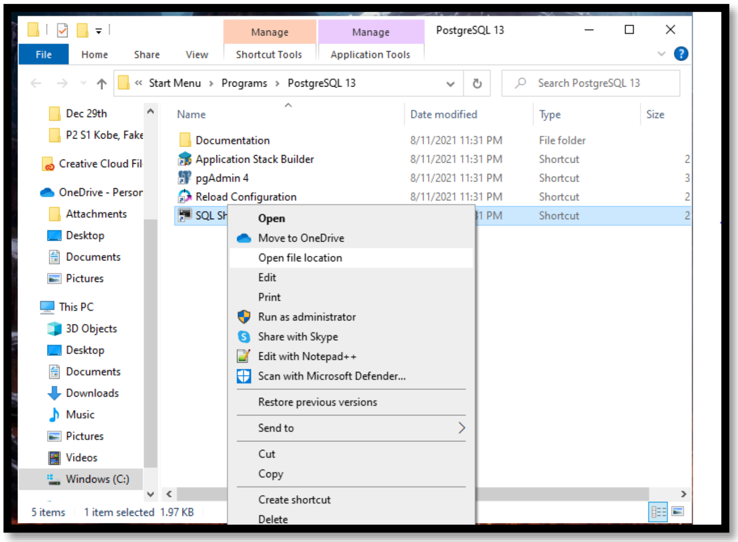

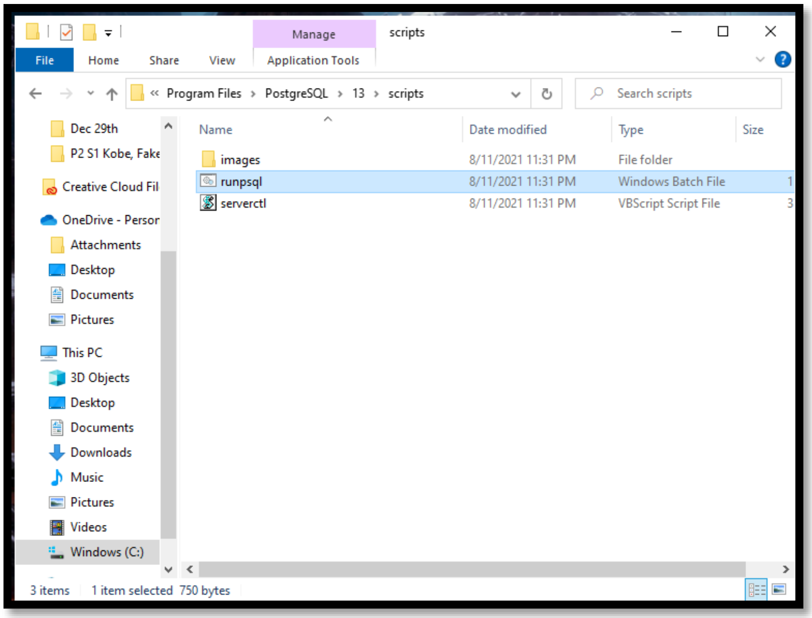

7. Click on “13” and arrive at a screen that should look like the following but may not match exactly. The important part is being able to find the “data” folder. Click on it and look for the pg_hba.conf file.

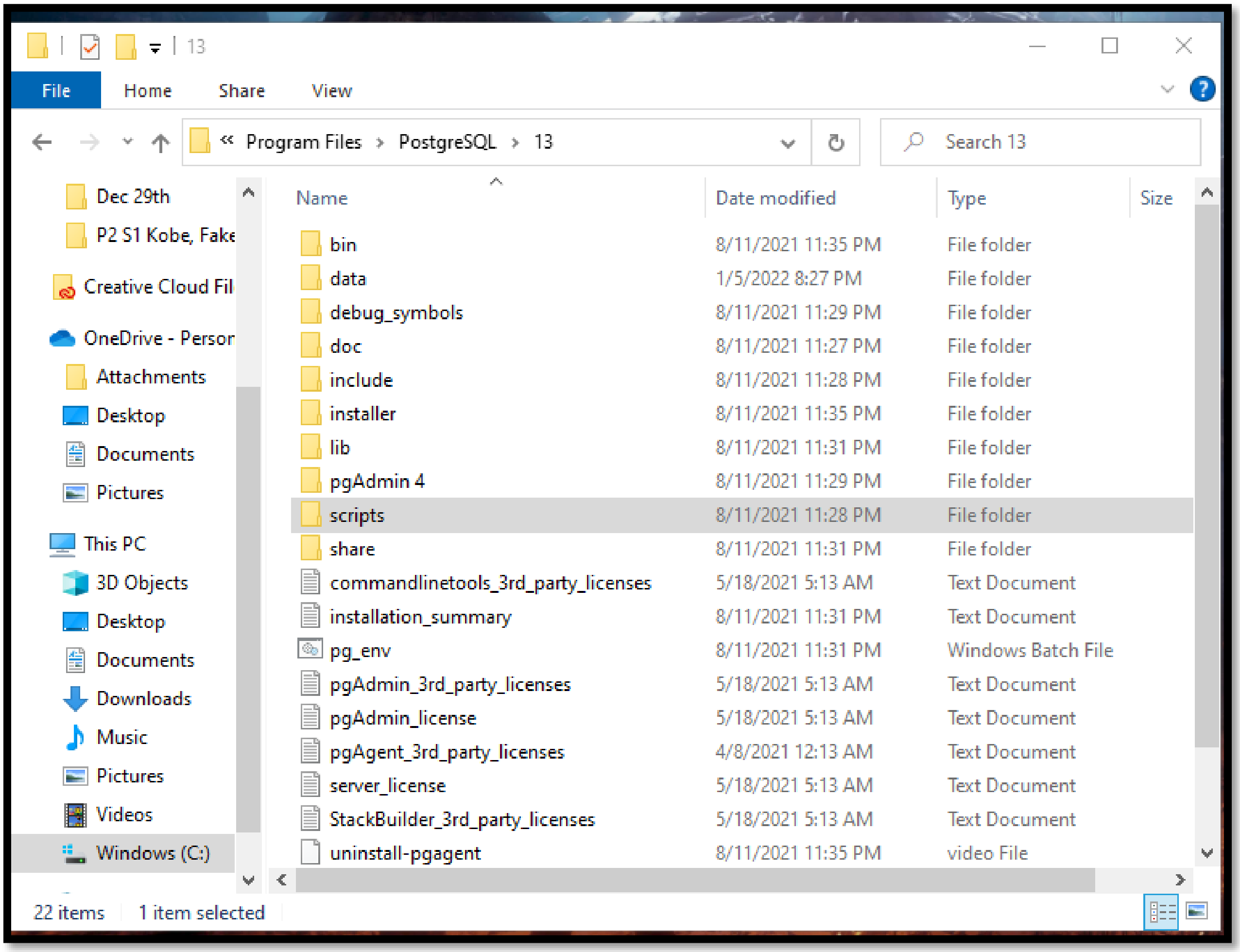

8. Open up the file and scroll all the way to the bottom, copy and past the following lines of code.

```
host     all               all               0.0.0.0/0
md5
host     all               all               ::/0
md5
```

9. It should look like the following

```
# TYPE  DATABASE        USER            ADDRESS                 METHOD

# "local" is for Unix domain socket connections only
local   all             all                                     scram-sha-256
# IPv4 local connections:
host    all             all             127.0.0.1/32            scram-sha-256
# IPv6 local connections:
host    all             all             ::1/128                 scram-sha-256
# Allow replication connections from localhost, by a user with the
# replication privilege.
local   replication     all                                     scram-sha-256
host    replication     all             127.0.0.1/32            scram-sha-256
host    replication     all             ::1/128                 scram-sha-256
host    all             all             0.0.0.0/0               md5
host    all             all             ::0/0                   md5
host    all             all              0.0.0.0/0                       md5
host    all             all              ::/0                            md5
```

Creating A Server and Database

Creating the Server and Database allows for the data our project is creating to be stored, thus allowing us to access it whenever necessary.

1. The easiest way to do this is through the "pgAdmin" GUI already installed when you installed PostgreSQL
2. Simply search in the windows start for "pgAdmin" and you should see the following

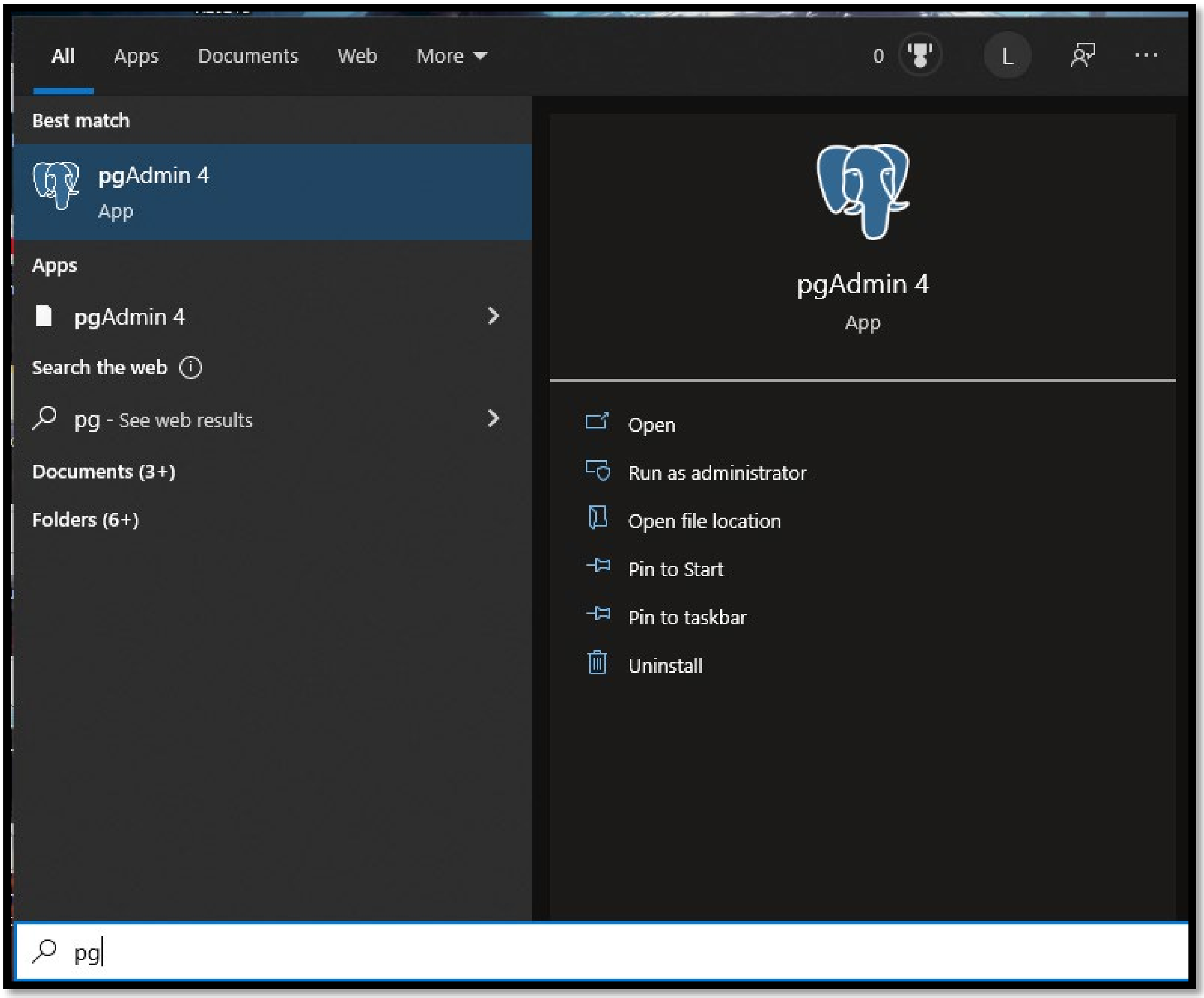

What’s the recommended password

3. Open up the app, you will be prompted to set a master password, make it something simple, recommended “1234”
4. With everything set up you should see a screen like the following

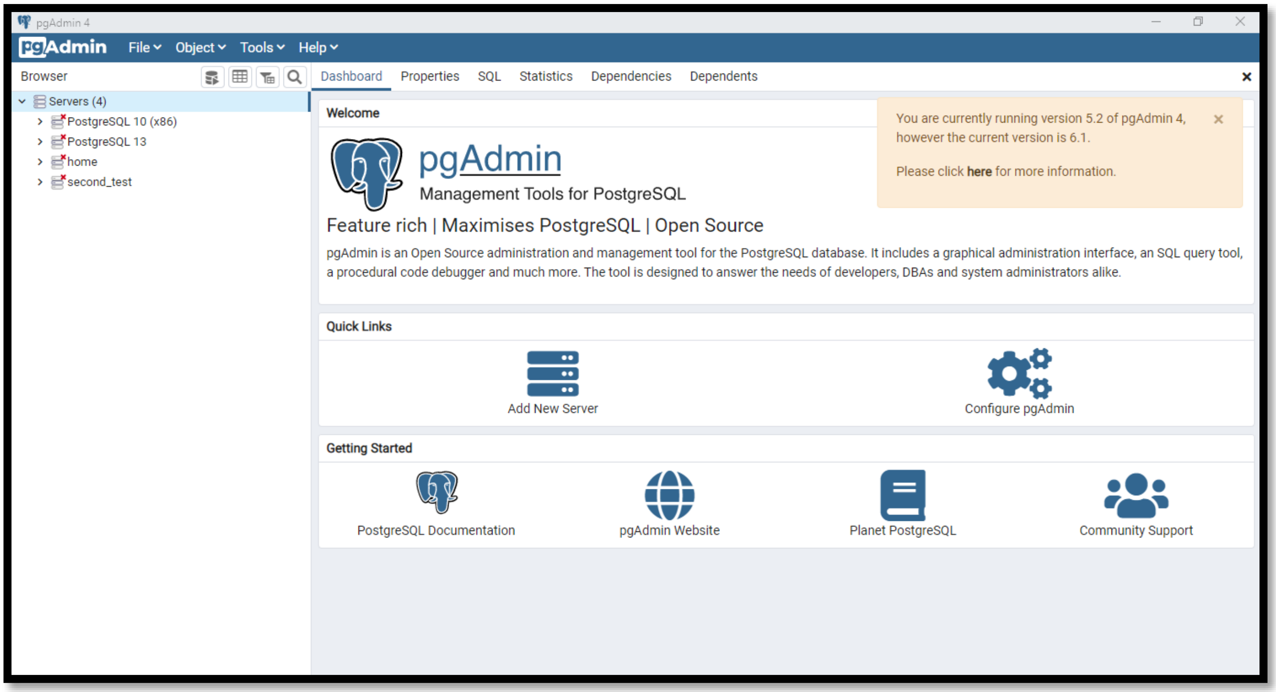

5. You will not have as many servers as you see in the photo, you will likely start off with just 1 named “PostgreSQL 13”

6. Right click on the "servers" label, go to create-> server

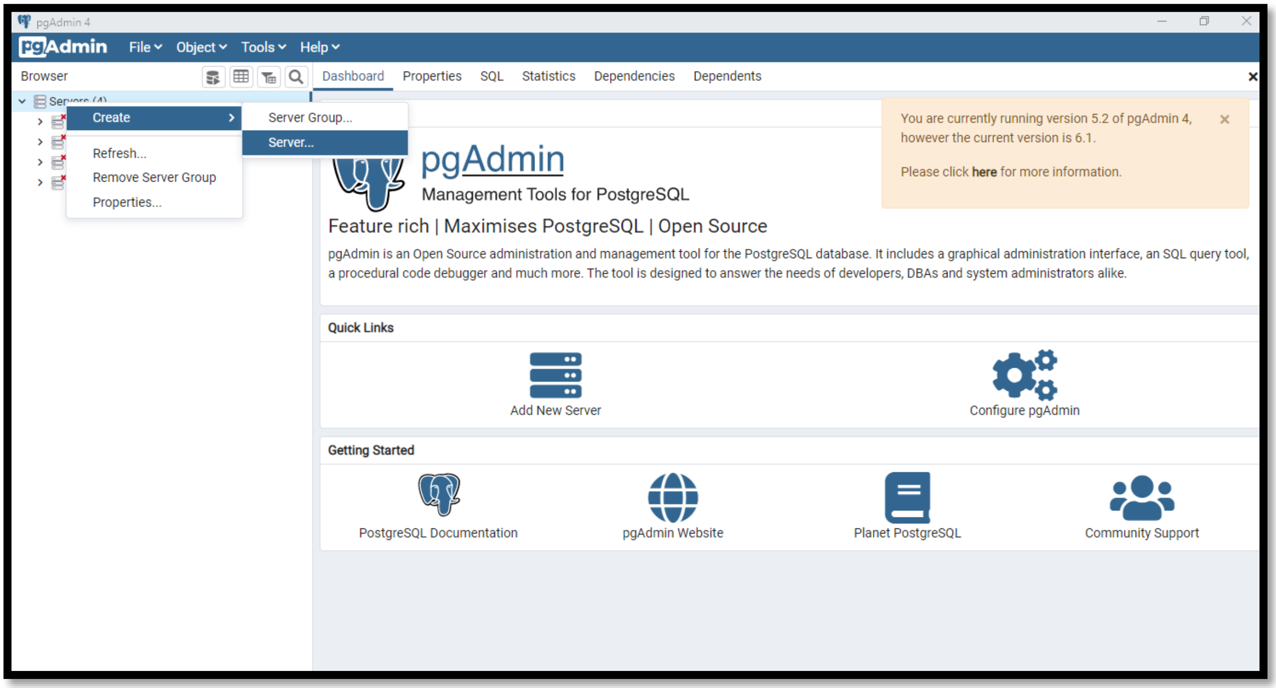

7. You'll arrive at the following screen

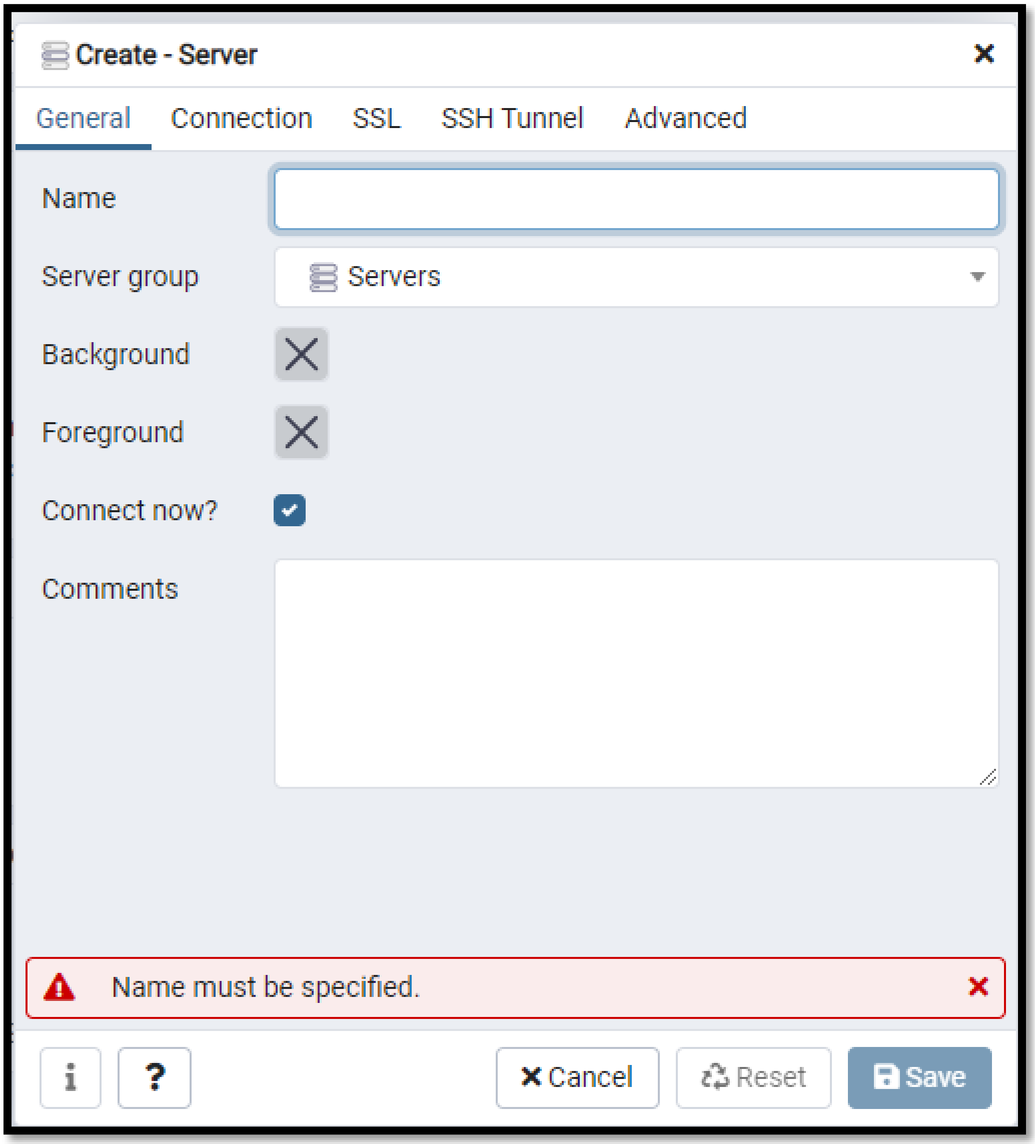

recommended server name

8. Fill "lab_server" into the "Name" field

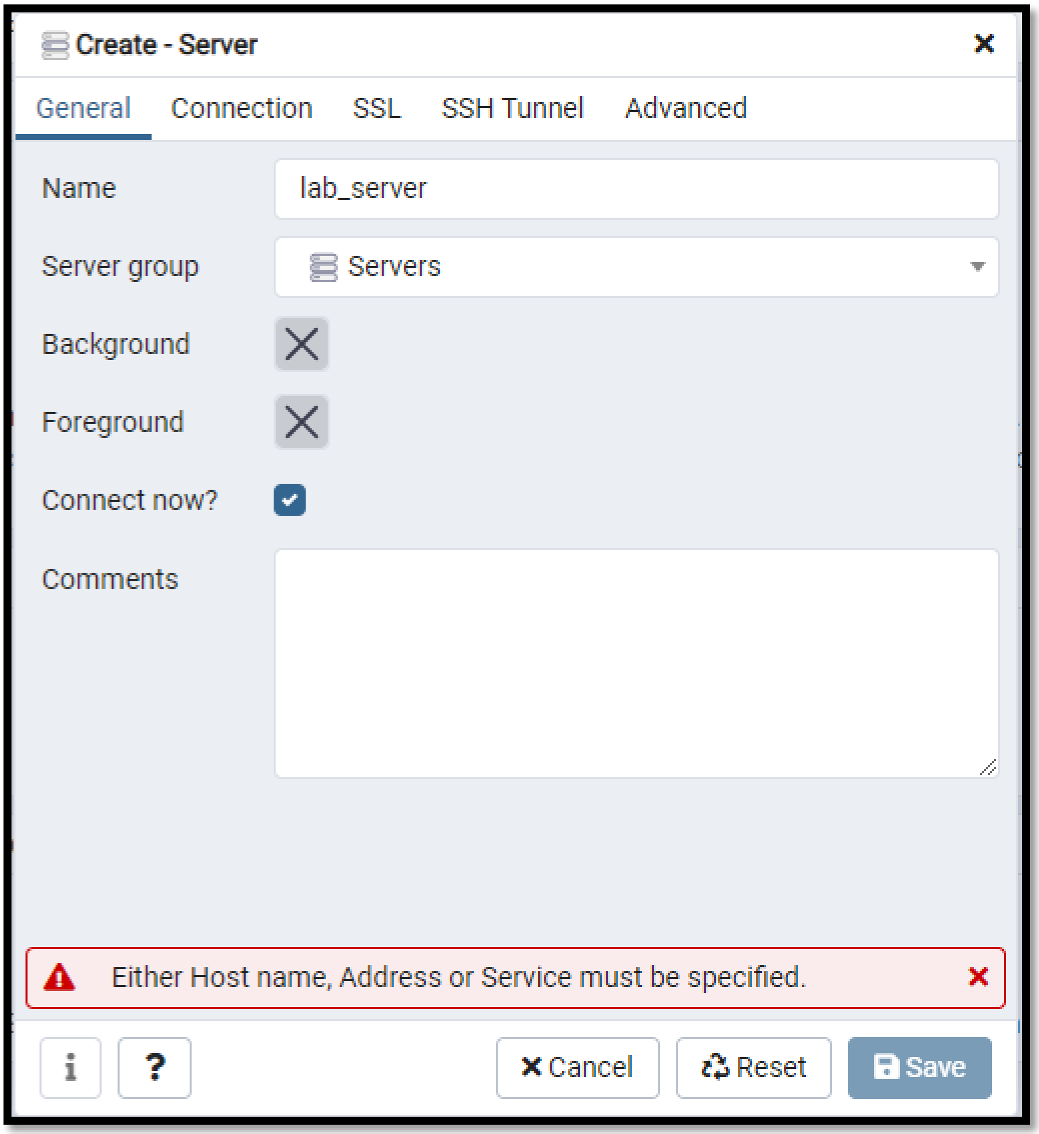

9. Click on the "Connection" tab and arrive at the following screen

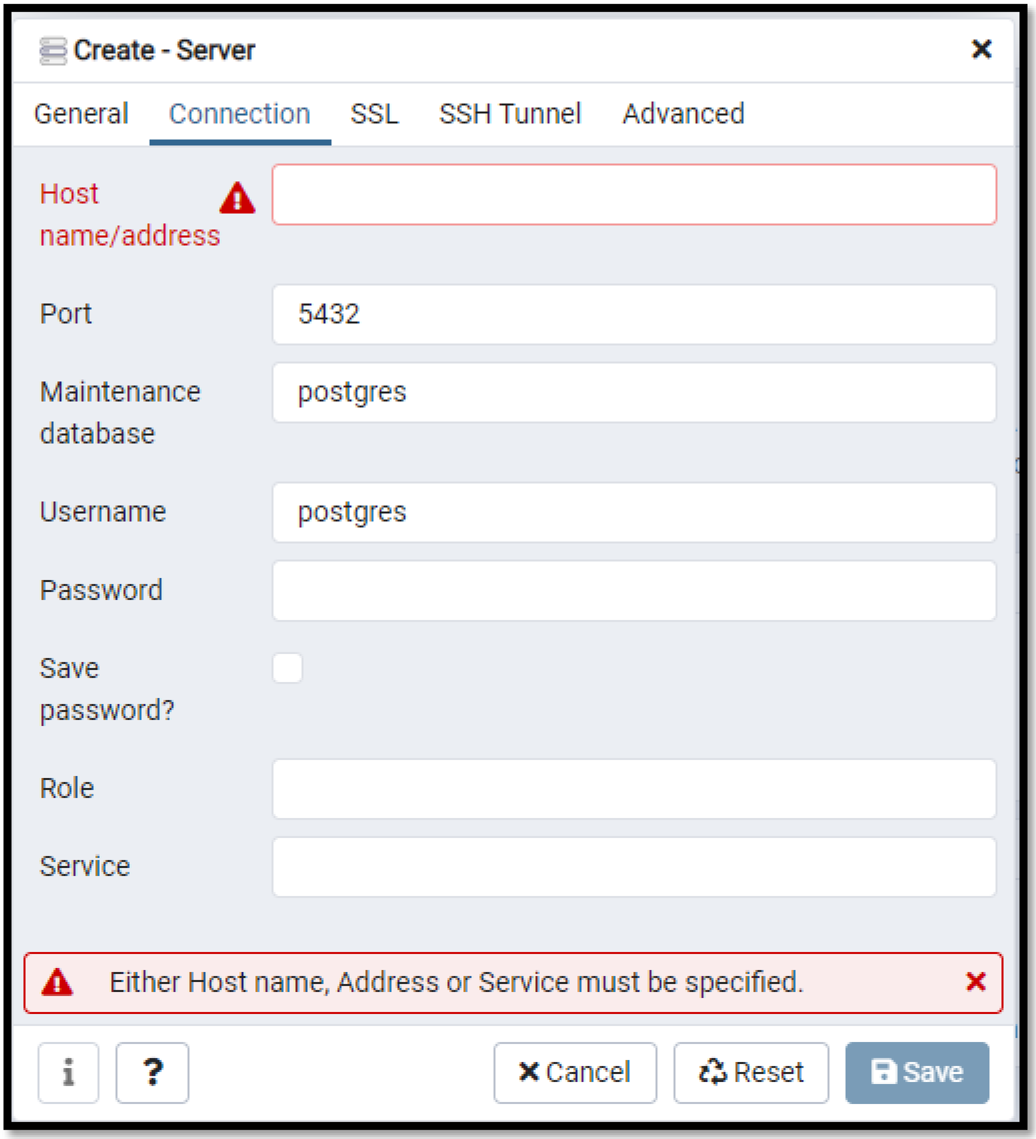

10. Fill “localhost” in the “Host name/address” field

11. Fill “5432” in the “Port” field

12. Fil “postgres” in the “Maintenance database” field

13. Fill “postgres” in the “Username” field

14. Fill the previously created master password into the “Password” field

15. Click on Save

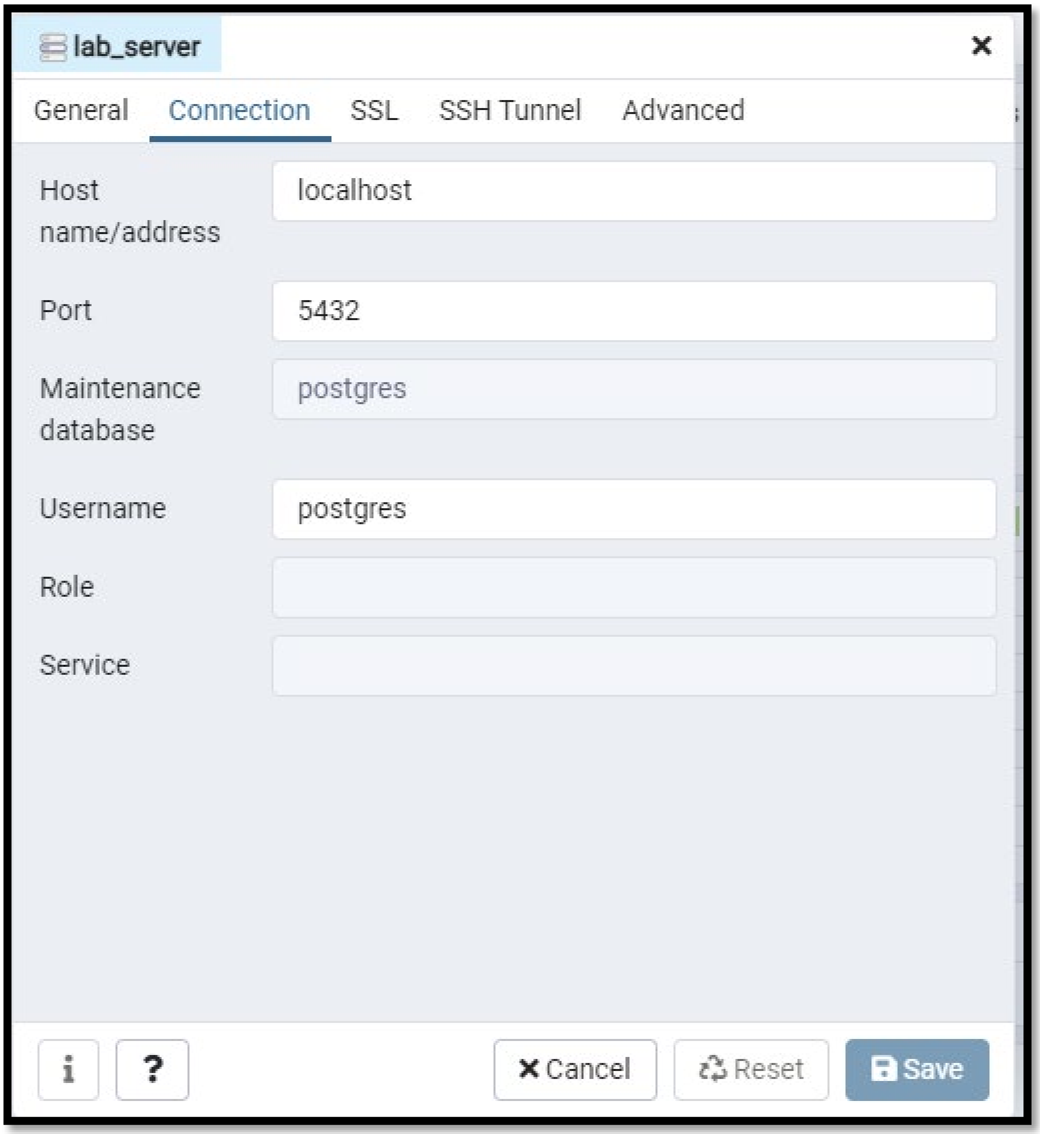

16. You'll now see a new server in your servers group named "lab_server"

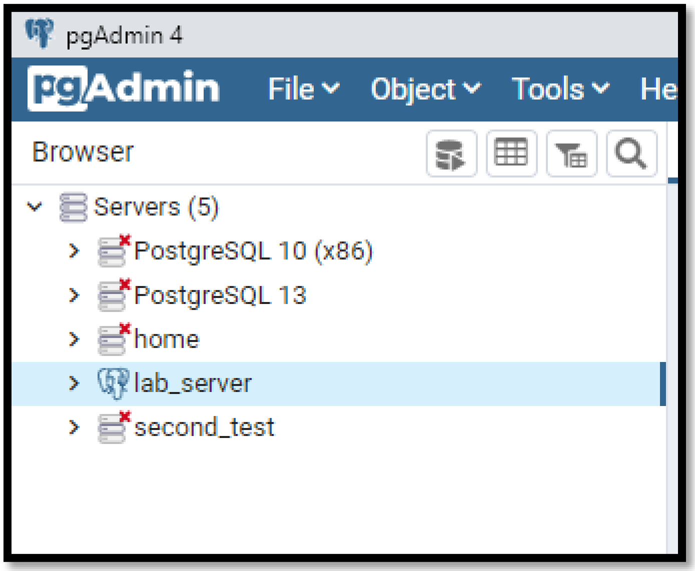

17. With this done expand the “lab_server” tab and right click on “Databases”

18. Go to “Create” -> “Database”

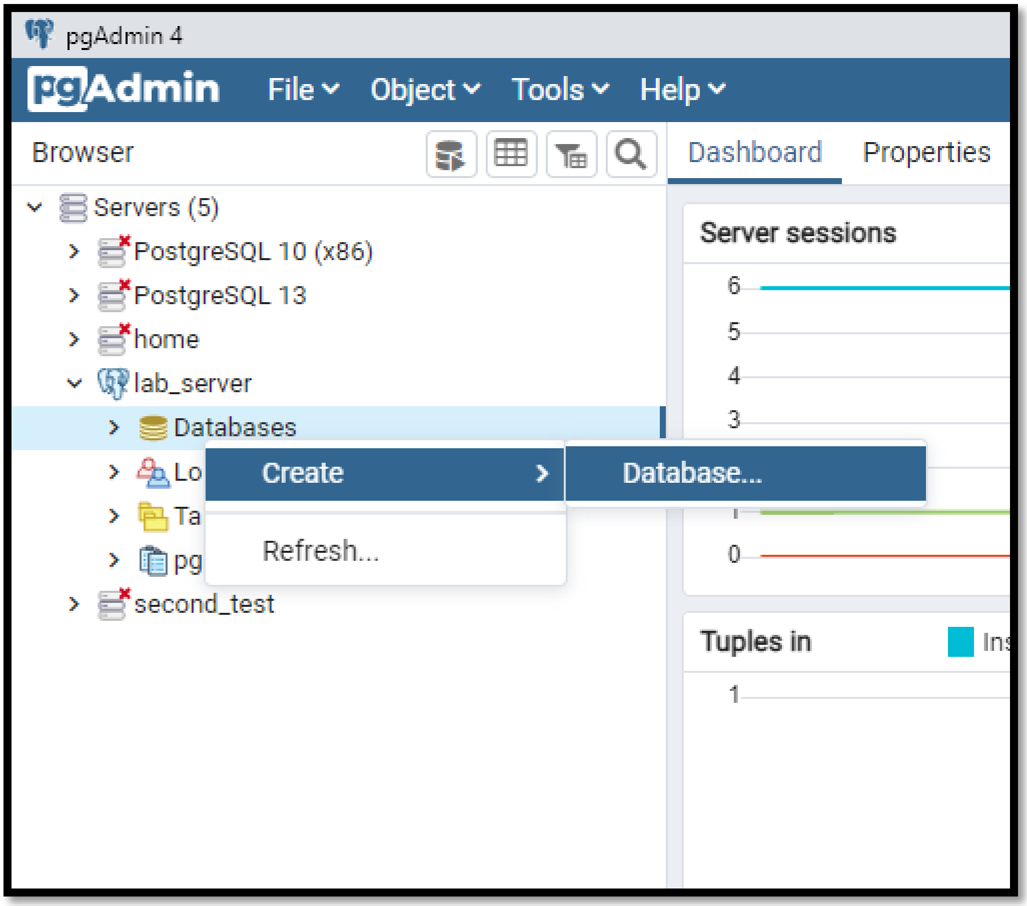

Recommended Database Name

19. Arrive at the following screen and fill in “Live_Database” into the “Database” field and click save

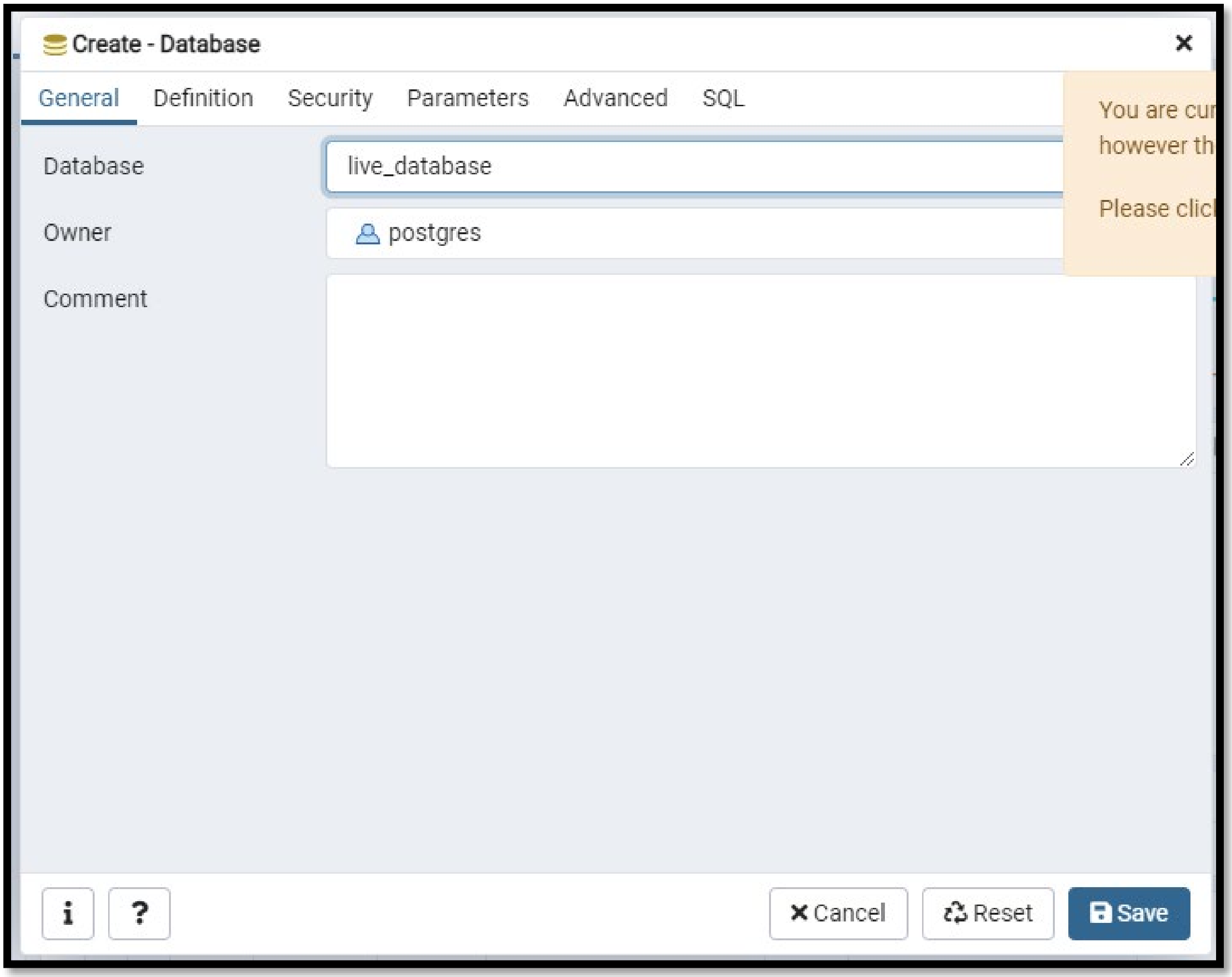

20. If everything was done correctly then by expanding the “database” menu you should see your newly created database

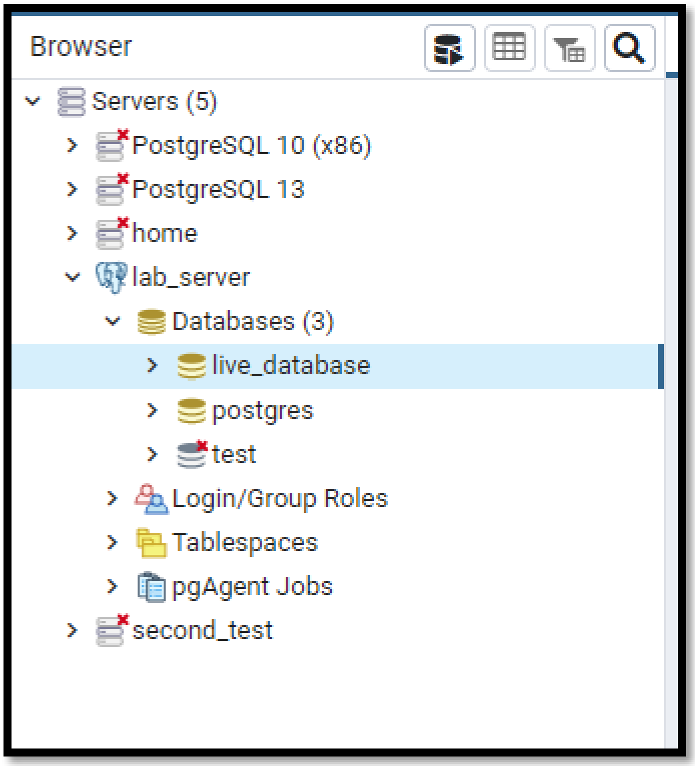

21. With all this done your database is now set up

Creating a Database Connection

Creating the database connection enables the Serendipity app, and other basic connection algorithms to be run. This allows us to actually access the data we have created, enabling the project to move forward.

1. To create a database you must have the MATLAB database toolbox installed, otherwise it is impossible
2. If you do not have it installed, please download it at the following link

https://www.mathworks.com/products/database.html

3. With the database tool box installed you can now proceed with making a database connection
4. Begin by opening MATLAB
5. Navigate to the "Apps" tab in the taskbar
6. Select the "Database Explorer"
    a. You may need to select the down arrow if the "Database Explorer" app isn't immediately seen among the other apps
    b. The "Database Explorer" icon can be seen below

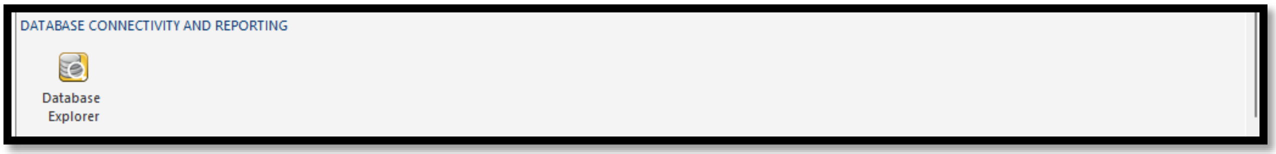

7. With the database explorer app now open go to the “Connections” grouping and select the “Connect” drown down arrow which will open a new menu

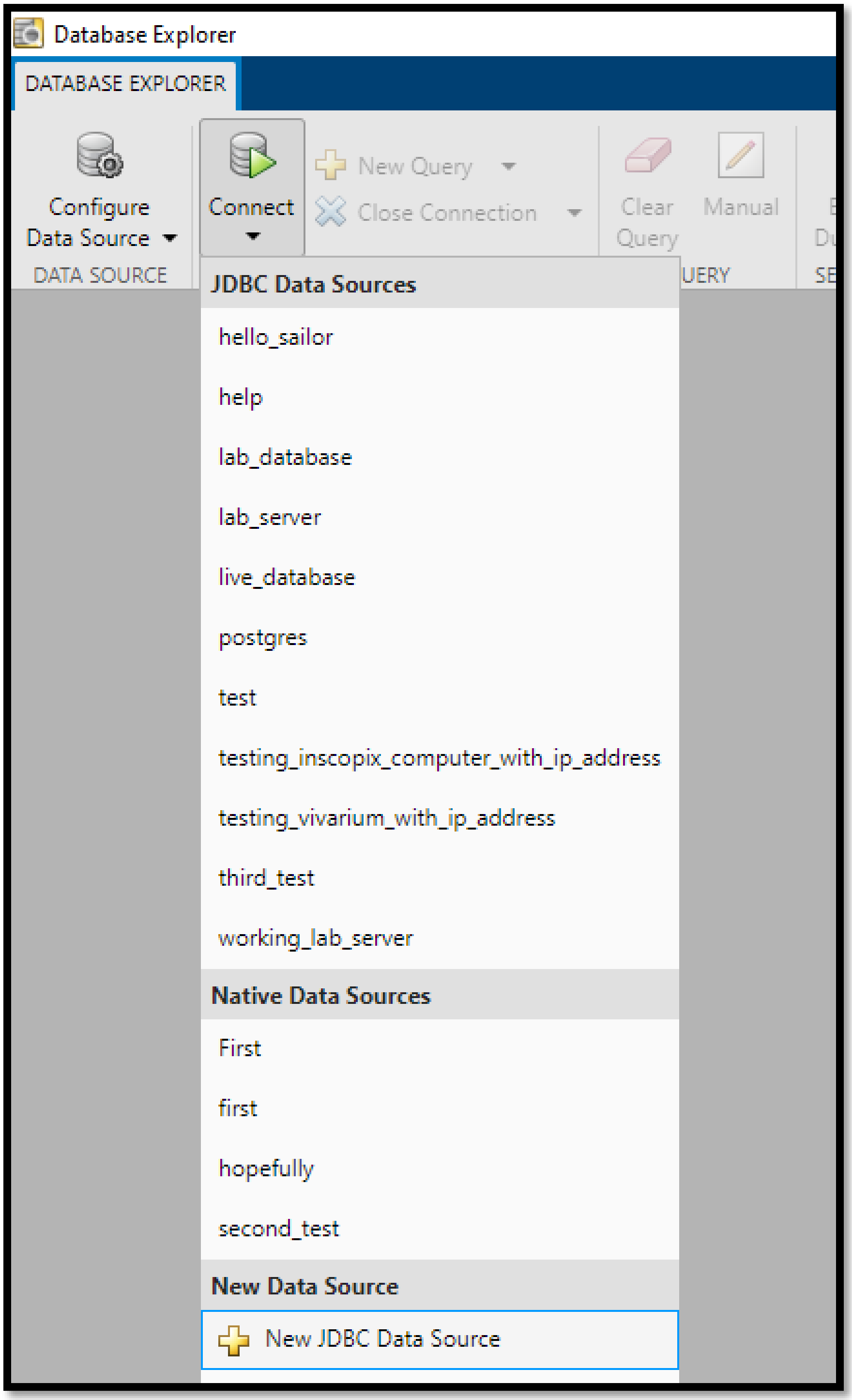

8. Navigate down to "New Data Source" and Select the "New JDBC Data Source" option which will automatically open a creation wizard

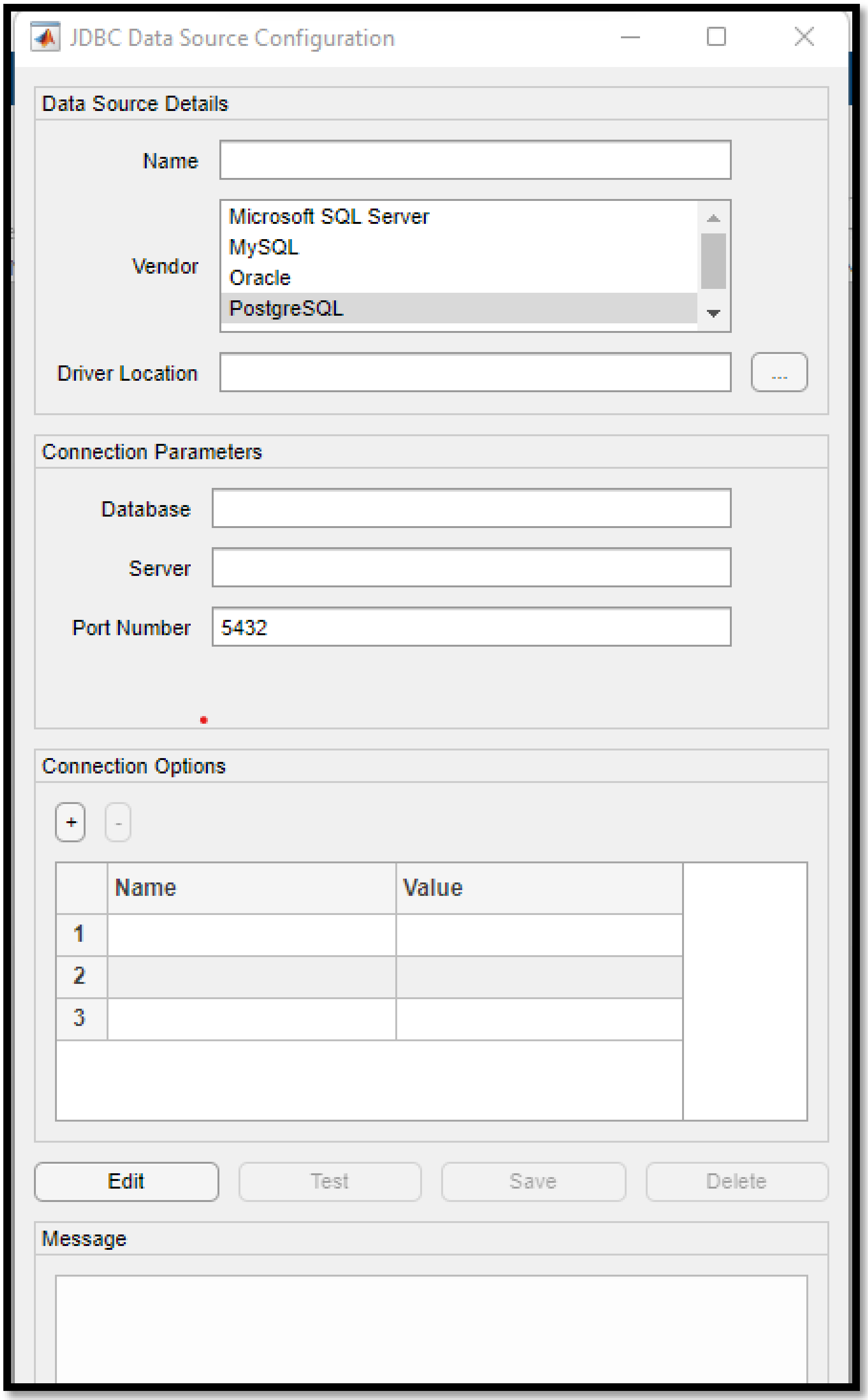

What should I put in my fields?

Name

If you used the recommended database name found in "Recommended Database Name" then you should enter "live_database" into this field. If another name was entered when creating the database that same name must be used here.

Common mistake

If the name of the database and the name of the database connection do not match, none of the code will work

Vendor

Select PostgreSQL as the vendor.

Driver Location

In the "Things to Download Before Beginning" section of this paper, you were instructed to download the JDBC driver file. Here you must select that file before proceeding.

Database

If you used the recommended database name then you should enter "live_database", if you named it something else you must use the other name.

Common Mistake

If the value entered here does not match the name of the database entered when creating the database then none of the code will work.

Server

Enter "localhost" here.

Port Number

By default it should populate 5432, and this should be perfect as long as the ports were never manually changed by someone for security purposes.

9. With all these fields filled in you can now click on the "test" button.

10. A prompt for the password and username will appear

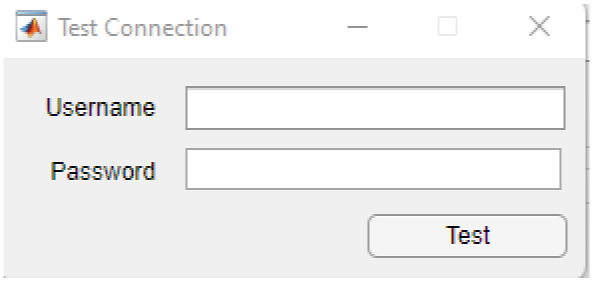

11. Enter “postgres” into the username and “1234” for the password

Creating the Live Table In your database (createLiveTable.m)

It is important to create the live_table because this is where everything will be uploaded and sorted. This table allows our project to quickly and efficiently access our data.

1. To create a table in the “lab_server” database where the information will actually be stored locate the “createLiveTable.m” file
2. Fill in the password, and click run
3. The live_table is now created and is ready to be written to
4. By default the live_table has the following columns

1. subjectID
2. gender
3. birthdate
4. genotype
5. cagenumber
6. health
7. cagemates
8. experimenter
9. tasktypedone
10. notes
11. intensityofcost1
12. intensityofcost2
13. intensityofcost3
14. intensityofcost4
15. costprobability1
16. costprobability2
17. costprobability3
18. costporbability4
19. rewardconcentration1
20. rewardconcentration2
21. rewardconcentration3
22. rewardconcentration4
23. rewardvolume1
24. rewardvolume2
25. rewardvolume3
26. rewardvolume4
27. rewardprobability1
28. rewardprobability2
29. rewardprobability4
30. rewardprobability4
31. mazenumber
32. approachavoidtimestamp
33. approachavoid
34. playstarttrialtone
35. presentcost
36. lightlevel
37. referencetime
38. videostarttime
39. feeder
40. stoptrack
41. trialname
42. detectionsettings
43. trialcontrolsettings
44. referenceduration
45. animalid
46. mazeofferdelivery
47. nazenoofferdelivery
48. starttime
49. recordingafter
50. recordingduration
51. trialduration
52. mazecostoff
53. coordinatetimes
54. xcoordinates
55. ycoordinates
56. presentcostend
57. costpresentationfinish

58. stopincopixrecording
59. decisionmakingtime
60. startincopixrecording
61. date
62. activezonetimestamp
63. activezonevalue
64. presentcosttimestamp
65. costacknowledgementtime stamp
66. deliveryacknowledgedtime stamp

A

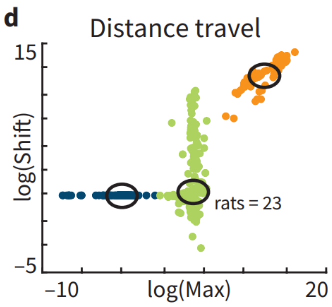

Figure 1: Travel Pixel Max Vs Shift MPC: 0.8925

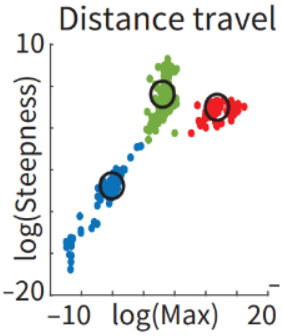

Figure 2: Travel Pixel Max Vs Steepness, MPC: 0.8925

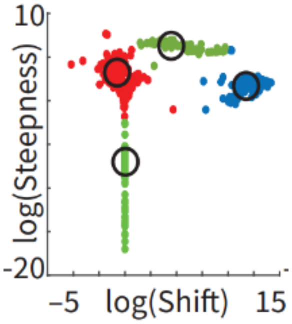

Figure 3: Travel Pixel Shift Vs Steepness, MPC: 0.8628

1) Navigate into Data Analysis Directory
2) Navigate into Old Base Data Directory
3) Navigate into Travel Pixel Directory
4) Open TravelPixelSigmoidClustering.m and make the following edits
    a) Modify the "myDir" variable so it points to "All Sigmoids" which is in the Travel Pixel Directory specified in step 3

```
myDir = "C:\Users\ldd77\OneDrive\Desktop\UTEP-Brain-Computation-Lab\Data Analysis\Travel Pixel\All Sigmoids"; %gets directory
```

Figure 4: example of the variable that has to be modified in travelPixelSigmoidClustering.m

    b) Modify the fullFileName variable with the same path used in myDir variable.

```
fullFileName = fullfile("C:\Users\ldd77\OneDrive\Desktop\UTEP-Brain-Computation-Lab\Data Analysis\Travel Pixel\All Sigmoids", baseFileName);
```

Figure 5: example of the fullFileName variable which needs to be changed in travelPixelSigmoidClustering.m to work

    c) travelPixelSigmoidClustering.m will create a variable called newTable which contains all the data obtained from fitting the raw data with a sigmoid.
5) Run travelPixelMaxVsShift.m, travelPixelMaxVsSteepness.m, and travelPixelShiftVsSteepness.m .

To create an updated figure from our raw data run the following functions. Keep in mind that these figures will likely include the data seen in the figures above, as well as new data. If there is a desire to recreate figure 5, figure 6, and figure 7 directly from raw data you must filter data down to the date ranges found in the file where you downloaded all the data.

Beginning Date Range	End Date Range
11-11-2021	05-23-2022

Table 1: Table of date ranges for base data sigmoid figures

automizeTravelPixelPsychomaticalFunction.m ×

This file will read from featuretable, located in the database backup found in Data Base Backup As 04-11-2023.zip. For each trial in a session that a rat performs it will take the average measured travel pixel, for each of the 4 feeder values. It will write these values to the table found in the database “travelPixelPsychomaticalFunctions”.

1. createMap ()
    a. This function returns a Map T
        i. Keys are dates.
        ii. The values are a list of all rats to run a session on this date.
    b. This function must be modified.
        i. The variable “query” located in line 3 of the file must be modified to exclude any experiment data.

```
query = "SELECT referencetime, subjectid, mazenumber from live_table WHERE LOWER(health)='n/a';"';
```

Figure 6: Example query that filters out all experiment data.

        ii. This must be modified because at the date of this figure creation no experiments were being run and as a result there was no need to filter, but the database now includes experiment data.
2. createPsychomaticalFunction(searchResults)
    a. This function returns xcoordinates, ycoordinates.
    b. xcoordinates is the following array of values.
        i. [0.005, 0.01, 0.02, 0.09]
        ii. Each of these values represents the percentage of glucose located in a feeder which is present during the trials.

c. searchResults is a MATLAB in the following form.

id	travelpixel
1	10000

Table 2: Example of how the searchResults table must be formatted.

d. You can get a table like this by using the following query
   i. date is a date that is formatted in MM-shortened Month-YYYY format
   ii. animalsubjectid is any name of an animal in the table

```
query = strcat("SELECT id, travelpixel FROM featuretable WHERE referencetime LIKE '",date,"%'","AND subjectid ='",animalsubjectid,"';");
searchResults = fetch(conn,query);
```

Figure 7: Query that will return searchResults in a format that will work for createPsychomaticalFunction().

e. ycoordinates is the averages of travel pixel recorded during each of these feeder values.
   i. The average is calculated by summing the travel pixel based on which feeder was active during the trial, and dividing by the number of trials that used that feeder.

3. Once the functions above are run they will automatically write the results to a table in the database named “travelPixelPsychomaticalFunctions” in the following format

subjectid	date	x1	x2	x3	x4	y1	y2	y3	y4
example id	example date	0.005	0.01	0.02	0.09	average	average	average	average

Table 3: Example of travelPixelPsychomaticalFunctions table

travelPixelSigmoidAnalysis.m

1. This function will read from the “travelPixelPsychomaticalFunctions” table created by automizeTravelPixelPsychomaticalFunction.m and fit the data there with a parabola, a sigmoid, and a line.
2. Coefficient of determination (R-Squared) is used to measure goodness of fit
3. Each row of data in the “travelPixelPsychomaticalFunctions” table will be sorted into folders based on which model fits it better, determined by which produces a higher R-Squared value.

After these functions are run, you can now run travelPixelMaxVsShift.m, travelPixelMaxVsSteepness.m, and travelPixelShiftVsSteepness.m. This should create updated figures.

Fig 4d, SF4a Baseline Stopping Point Figures

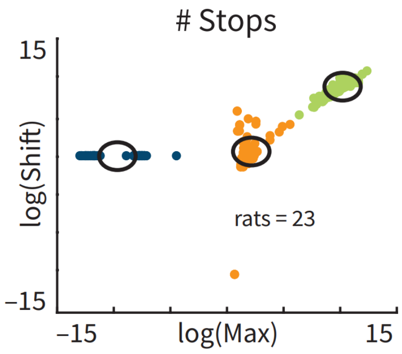

Figure 8: Stopping Points Max Vs Shift MPC: 0.9279

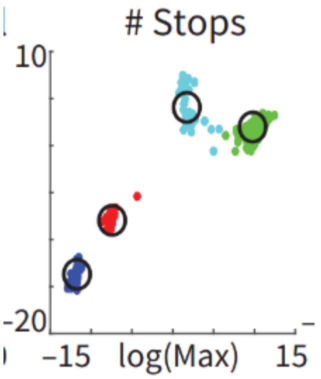

Figure 9: Stopping Points Max Vs Steepness 0.9279

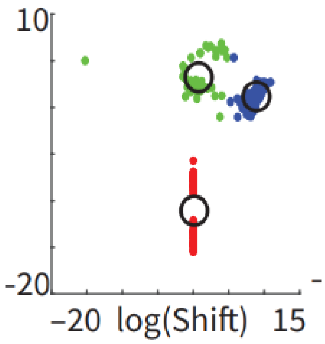

Figure 10: Stopping Points Shift Vs Steepness MPC: 0.9279

1) Navigate into Data Analysis Directory
2) Navigate into Old Base Data Directory
3) Navigate into Stopping Points Directory
4) Open stoppingPointsSigmoidClustering.m and make the following edits.
   a) Modify the "myDir" variable so it points to "All Sigmoids" which is in the Stopping Points Directory specified in step 3.

```
myDir = "C:\Users\ldd77\OneDrive\Desktop\UTEP-Brain-Computation-Lab\Data Analysis\Travel Pixel\All Sigmoids"; %gets directory
```

Figure 11: example of the variable that has to be modified in stoppingPointsSigmoidClustering.m

   b) Modify the fullFileName variable with the same path used in myDir variable.

```
fullFileName = fullfile("C:\Users\ldd77\OneDrive\Desktop\UTEP-Brain-Computation-Lab\Data Analysis\Travel Pixel\All Sigmoids", baseFileName);
```

Figure 12: example of the fullFileName variable which needs to be changed in stoppingPointsSigmoidClustering.m to work

   c) stoppingPointsSigmoidClustering.m will create a variable called newTable which contains all the data obtained from fitting the raw data with a sigmoid.
5) Now run the following files: stoppingPointsMaxVsShift.m, stoppingPointsMaxVsSteepness.m, stoppingPointsShiftVsSteepness.m.

To create an updated figure from our raw data run the following functions. Keep in mind that these figures will likely include the data seen in the figures above, as well as new data. If there is a desire to recreate figure 12, figure 13, and figure 14 directly from raw data you must filter data down to the date ranges found in the file where you downloaded all the data.

automizestoppingptsPsychomaticalFunction.m

This file will read from featuretable, located in the database backup found in Data Base Backup As 04-11-2023.zip. For each trial in a session that a rat performs it will take the average measured stopping points, for each of the 4 feeder values. It will write these values to the table found in the database "stoppingptsPsychomaticalFunctions".

1) createMap ()
   a) This function returns a Map T
      i) Keys are dates.
      ii) The values are a list of all rats to run a session on this date.
   b) This function must be modified.
      i) The variable “query” located in line 3 of the file must be modified to exclude any experiment data.

```
query = "SELECT referencetime, subjectid, mazenumber from live_table WHERE LOWER(health)='n/a';"';
```

Figure 13: Example query that filters out all experiment data.

2) createPsychomaticalFunction(searchResults)
   a) This function returns xcoordinates, ycoordinates.
   b) xcoordinates is the following array of values. [0.005, 0.01, 0.02, 0.09]
      i) Each of these values represents the percentage of glucose located in a feeder which is present during the trials.
   c) searchResults is a MATLAB in the following form.

id	stoppingpts
1	10000

Table 4 Example of how the searchResults table must be formatted.

   d) You can get a table like this by using the following query.
      i) date is a date that is formatted in MM-Shortened Month-YYYY format.
      ii) animalsubjectid is any name of an animal in the table.

```
query=strcat("SELECT id, stoppingpts FROM featuretable WHERE referencetime LIKE '", date,"%'","AND subjectid = '",animalsubjectid,"';'");
searchResults = fetch(conn,query);
```

Table 5: Query that will return searchResults in a format that will be accepted by createPsychomaticalFunction().

3) Once the functions above are run they will automatically write the results to a table in the database named “stoppingptsPsychomaticalFunctions” in the format seen in table 8.

stoppingptsSigmoidAnalysis.m

1) This function will read from the “stoppingptsPsychomaticalFunctions” table created by automizestoppingptsPsychomaticalFunction.m and fit the data there with a parabola, a sigmoid, and a line.
2) Coefficient of determination (R-Squared) is used to measure goodness of fit
3) Each row of data in the “travelPixelPsychomaticalFunctions” table will be sorted into folders based on which model fits it better, determined by which produces a higher R-Squared value.

After these functions are run, you can now run stoppingPtsMaxVsShift.m, stoppingPtsMaxVsSteepness.m, and stoppingPtsShiftVsSteepness.m. This should create updated figures.

Fig 4d, SF4a Baseline Rotation Points Figures

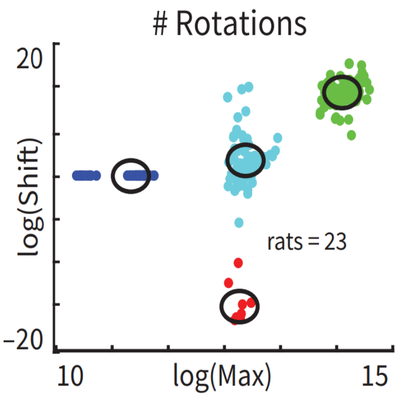

Figure 14: Rotation Points Max Vs Shift MPC 0.9300

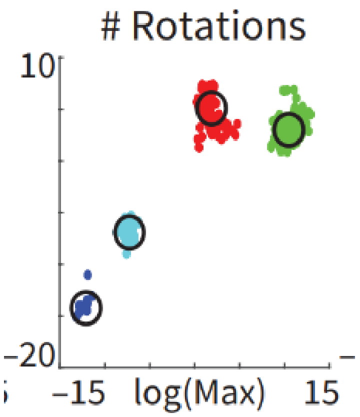

Figure 15: Rotation Points Max Vs Steepness: 0.9422

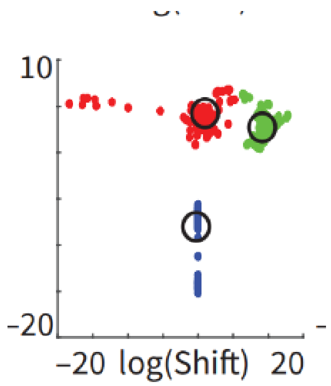

Figure 16: Rotation Points Shift Vs Steepness MPC: 0.8946

1) Navigate into Data Analysis Directory
2) Navigate into Old Base Data Directory
3) Navigate into Rotation Points Directory
4) Open rotationPointsSigmoidClustering.m
   a) Modify the "myDir" variable so it points to the "All Sigmoids", which is in the Rotation Points directory specified in step 3, as shown in figure 8.
   b) Modify the fullFileName variable with the same path used in myDir variable, see figure 9 for an example.
   c) RotationPtsSigmoidClustering.m will create a variable called newTable which contains all the data obtained from fitting the raw data with a sigmoid
5) Now run the following files: rotationPtsMaxVsShift.m, rotationPtsMaxVsSteepness.m, rotationPtsShiftVsSteepness.m

To create an updated figure from our raw data run the following functions. Keep in mind that these figures will likely include the data seen in the figures above, as well as new data. If there is a desire to recreate figure 18, figure 19, and figure 20 directly from raw data you must filter data down to the date ranges found in the file where you downloaded all the data.

automizeRotationPtsPsychomaticalFunction.m

This file will read from featuretable, located in the database backup found in Data Base Backup As 04-11-2023.zip. For each trial in a session that a rat performs it will take the average measured rotation points, for each of the 4 feeder values. It will write these values to the table found in the database "rotationPtsPsychomaticalFunctions".

1. This function works almost identically to automizeTravelPixelPsychomaticalFunction.m, but instead reference the following table instead of table 7 and figure 11.

id	rotationPts

1	10000

Table 6: Example of how the searchResults table must be formatted.

```
query=strcat("SELECT id, rotationpts FROM featuretable WHERE referencetime LIKE '", date,"%'","AND subjectid = '",animalsubjectid,"';'");
searchResults = fetch(conn,query);
```

Table 7: Query that will return searchResults in a format that will work for createPsychomaticalFunction.

2. Once the function runs it will write results to a table in the database named "rotationPtsPsychomaticalFunctions". See table 8 for the format.

RotationPtsSigmoidAnalysis.m

1. This function will read from the "rotationPtsPsychomaticalFunctions" table created by automizeRotationPtsPsychomaticalFunction.m and fit the data there with a parabola, a sigmoid, and a line.
2. Coefficient of determination (R-Squared) is used to measure goodness of fit
3. Each row of data in the "rotationPtsPsychomaticalFunctions" table will be sorted into folders based on which model fits it better, determined by which produces a higher R-Squared value.

After these functions are run, you can now run rotationPtsMaxVsShift.m, rotationPtsMaxVsSteepness.m, and rotationPtsShiftVsSteepness.m. This should create updated figures.

Fig 4d, SF4a Baseline Reaction Time Figures

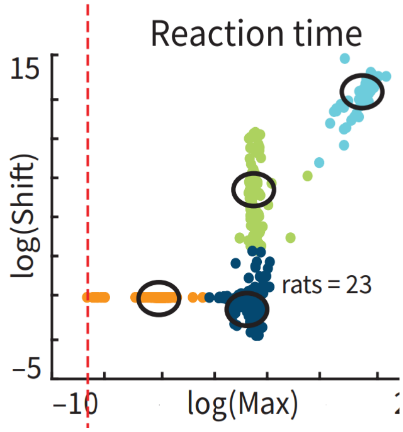

Figure 17: Reaction Time Max Vs Shift MPC: 0.8950

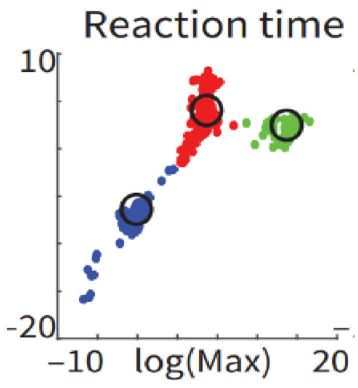

Figure 18: Reaction Time Max VS Steepness MPC: 0.9046

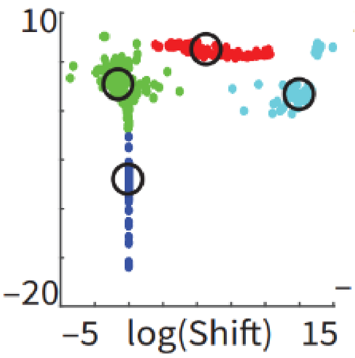

Figure 19: Reaction Time Shift Vs Steepness, MPC: 0.8575

1. Navigate into Data Analysis Directory.
2. Navigate into Old Base Data Directory.
3. Navigate into Reaction Time 1st Directory.
4. Open reactionTime1stSigmoidClustering.m.
   a. Modify the "myDir" variable so it points to "All Sigmoids" in the Reaction Time 1st directory, specified in step 3, for an example see figure 8.
   b. Modify the fullFileName variable with the same path used in myDir variable, see figure 9 for an example.
   c. automizereactiontime1stPsychomaticalFunction.m will create a variable called newTable which contains all the data obtained from fitting the raw data with a sigmoid.
5. Now run the following files: reactionTime1stMaxVsShift.m, reactionTime1stMaxVsSteepness.m, reactiontime1stShiftVsSteepness.m

To create an updated figure from our raw data run the following functions. These figures will include the data seen in the figures above, as well as new data. If you want to recreate figure 21, 22, 23 directly from raw data you must filter the data down to the date ranges found in the file where you downloaded all the data.

Automizereactiontime1stPsychomaticalFunctions.m

This file will read from featuretable, located in the database backup found in Data Base Backup As 04-11-2023.zip. For each trial in a session that a rat performs it will take the average measured rotation points, for each of the 4 feeder values. It will write these values to the table found in the database "basePsychometricFunctions".

1. This function works almost identically to automizeTravelPixelPsychomaticalFunction.m, but instead reference the following table instead of table 7 and figure 11.

id	reactionTime
1	20

Table 8: Example of how the searchResults table must be formatted for reactionTime1st.

```
query = strcat("SELECT id,reactiontime1st FROM featuretable WHERE " + ...
                        "referencetime LIKE '",date,"%'","" + ...
                        " AND subjectid = '",animalsubjectid ,"';");
```

Figure 20: Query that will return searchResults in a format that will work for automizereactiontime1stPsychomaticalFunctions.m

2. Once the function runs it will write results to a table in the database named "basePsychometricFunctions". See table 8 for the format.

ReactionTime1stSigmoidAnalysis.m

3. This function will read from the "basePsychometricFunctions" table created by automizereactiontime1stPsychomaticalFunction.m and fit the data there with a parabola, a sigmoid, and a line.
4. Coefficient of determination (R-Squared) is used to measure goodness of fit.
5. Each row of data in the "basePsychometricFunctions" table will be sorted into folders based on which model fits it better, determined by which produces a higher R-Squared value.

After these functions are run, you can now run reactionTime1stMaxVsShift.m, reactionTime1stMaxVsSteepness.m, and reactionTime1stShiftVsSteepness.m. This should create updated figures.

Fig 4d, SF4a Reward Choice Figures

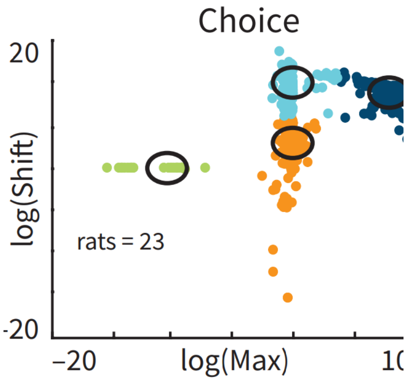

Figure 21: Reward Choice Max Vs Shift, MPC: 0.9057

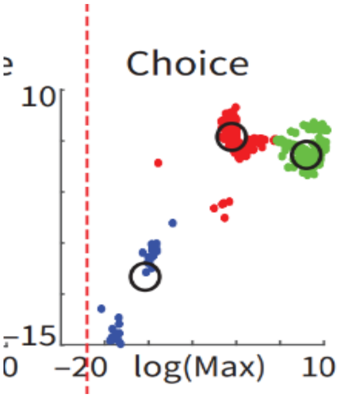

Figure 22: Reward Choice Max Vs Steepness, MPC: 0.9057

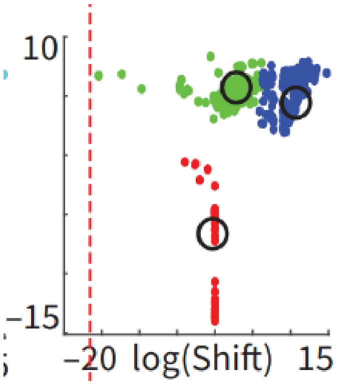

Figure 23: Reward Choice Shift Vs Steepness, MPC: 0.9057

1. Navigate Into Data Analysis Directory
2. Navigate into Old Base Data directory.
3. Navigate into Reward Choice directory.
4. Open [RewardChoiceSigmoidClustering.m]
    a. Modify the "myDir" variable so it contains the file path to "All Sigmoids" directory located in the Reward Choice directory specified in step 3, refer to figure 8 for an example.
    b. Modify the "fullFileName" variable with the same path used in myDir variable, refer to figure 9 for an example.
    c. RewardChoiceSigmoidClustering.m will create a variable called newTable which contains all the data obtained from fitting the raw data with a sigmoid.
5. Now run the following files: rewardChoiceMaxVsShift.m, rewardChoiceMaxVsSteepness.m, rewardChoiceShiftVsSteepness.m.

To create an updated figure from our raw data run the following functions, these updated figures will contain the old data and more. To recreate figures 26, 27, and 28 from raw data you must filter data down to the date ranges found in the file where you downloaded all the data.

automizePsychomaticalFunction.m

This file will read from live_table, located in the database backup found in Data Base Backup As 04-11-2023.zip. For each trial in a session that a rat performs it will take the average measured reward choice for each of the 4 feeder values. It will write these values to the table found in the database "psychomaticalFunctions".
This function works almost identically to automizeTravelPixelPsychomaticalFunction.m, but instead reference the following table instead of table 7 and figure 11.

```
query = strcat("SELECT * FROM live_table WHERE date = '", ...
    date,"' AND subjectid = '", id,"';");
```

Figure 24: Query that will return searchResults in a format that will work for automizePsychomaticalFunction.m

Once the function runs it will write results to a table in the database named “psychomaticalFunctions”. See table 8 for the format.

sigmoidAnalysis.m

1. This function will read from “psychomaticalFunctions” table created by automizePsychomaticalFunction.m and fit the data there with a parabola, a sigmoid, and a line.
2. Coefficient of determination (R-Squared) is used to measure goodness of fit.
3. Each row of data in the “psychomaticalFunctions” table will be sorted into folders based on which model fits it better, determined by which produces a higher R-Squared value.

After these functions are run, you can now run rewardChoiceMaxVsShift.m, rewardChoiceShiftVsSteepness.m, and rewardChoiceMaxVsSteepness.m. This should create updated figures.

Fig SF4dParabola Clustering

Figure 25: Parabola Clustering

1. Navigate into Parabolas Analysis.
2. Run the following file: [getThreeParametersFromParabolas.m]().
3. All the figures should be automatically created.

Fig 4e Average Travel Pixel Sigmoid

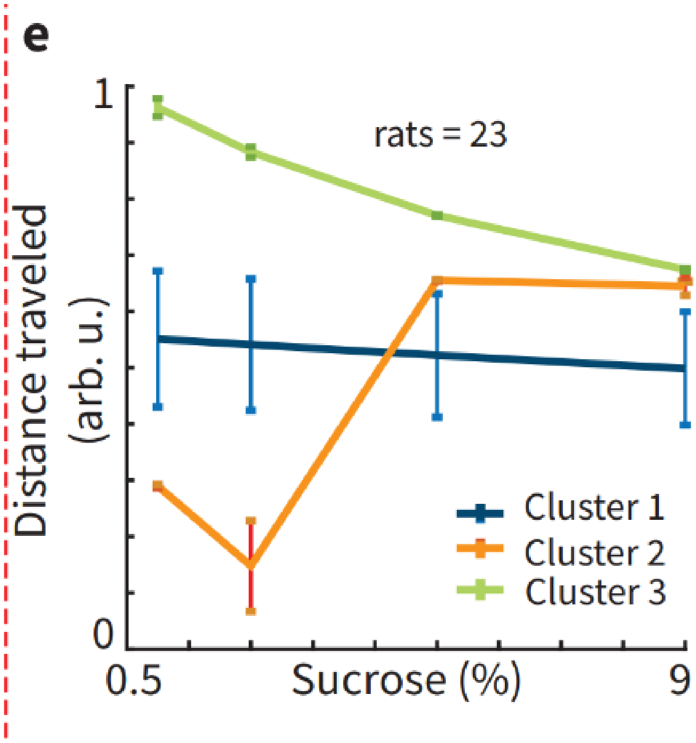

1. Navigate into Create Probability Tables

2. Run createFig4e.m

Fig 4f

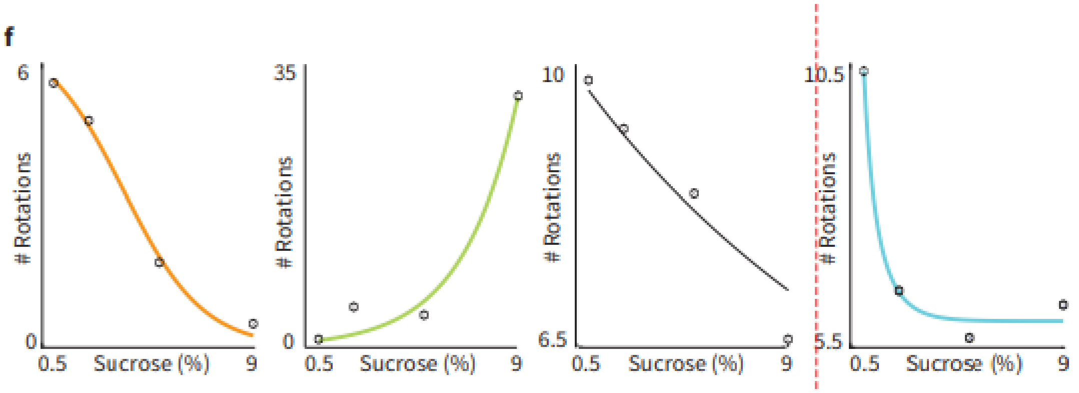

1. Navigate into Data Analysis directory.
2. Navigate into Old Base Data directory.
3. Navigate into Rotation Points directory.
4. These example sigmoid functions above exist within Rotation Pts 2 Parameter Sigmoid, Rotation Pts 3 Parameter Sigmoid, Rotation Pts 4 Parameter Sigmoid.
5. Also included in these folders are all the sigmoid functions created by fitting rotation point data
6. If you wish to generate new sigmoid functions, please follow the steps outlined in "Fig 4d, SF4a Baseline Rotation Points Figures"

Fig 4g Radar Plot

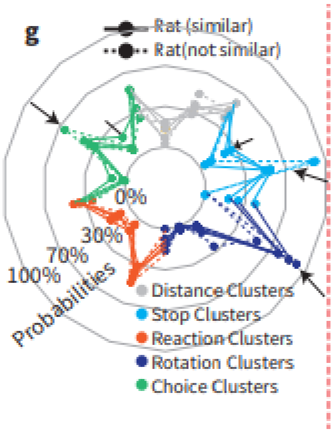

1. Navigate into Raw Figures directory.
2. Navigate into All Clusters directory.
3. Run radarPlotExperiments.m.

Fig 5j

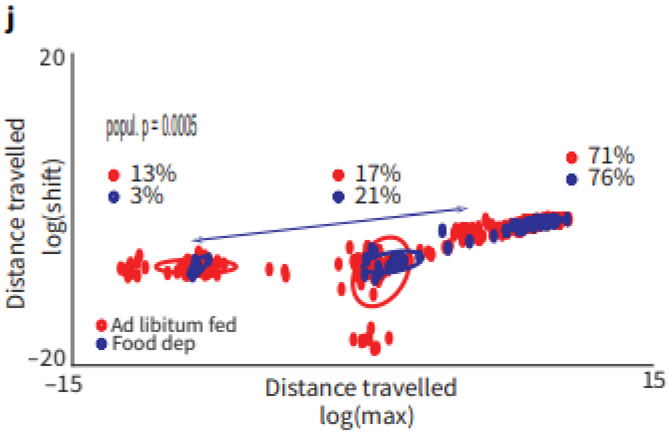

1. Navigate into “Updated Analysis” directory.
2. Navigate into “Utility Functions” directory.
3. Run the following matlab command in the command line
    a) “addpath(pwd)”

2) Navigate back up into the “Updated Analysis Directory”.
3) Run the file named runMe2.m.
4) This will be one of the figures created.

Fig 5k

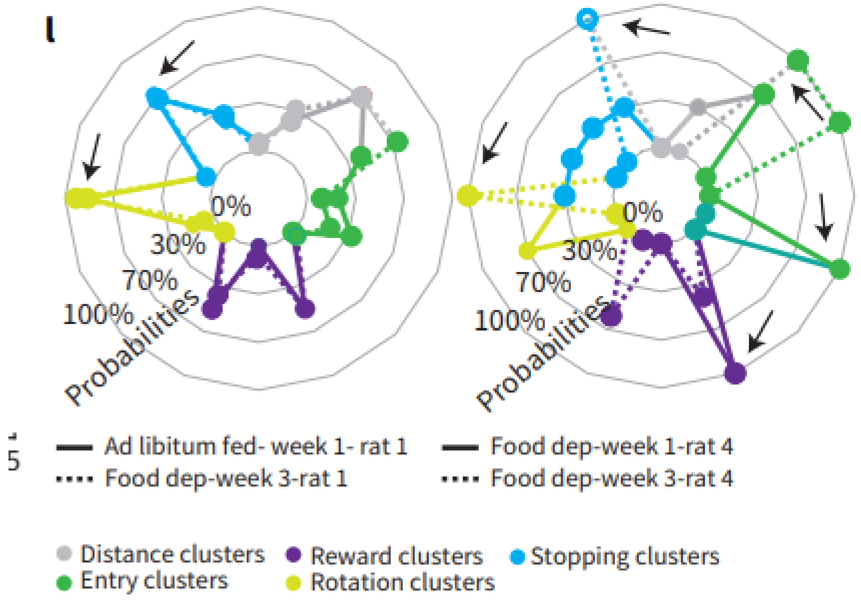

1. Navigate into “Updated Analysis” directory.
2. Navigate into “Utility Functions” directory.
3. Run the following matlab command in the command line
    a. “addpath(pwd)”
4. Navigate back up into the “Updated Analysis Directory”.
5. Run the file named runMe2.m.
6. Upon completion of running a directory labeled “First_and_last_Bin_Spider_plots” will be created.
7. Navigate inside “First_and_last_Bin_Spider_plots”.
8. Inside will be the following directories:
    a. Baseline
    b. Boost_And_Etho
    c. Food_Deprivation
    d. Ghrelin
    e. Oxy
    f. Saline
9. The left panel will be located within the directory named “Baseline” and will be named “1Baseline Early Bin sarah vs Baseline Late Bin sarah Spider Plot.fig”
10. The right panel will be located within the directory named “21FoodDeprivation Early Bin sully vs FoodDeprivation Late Bin sully Spider Plot.fig”.

Fig 5m

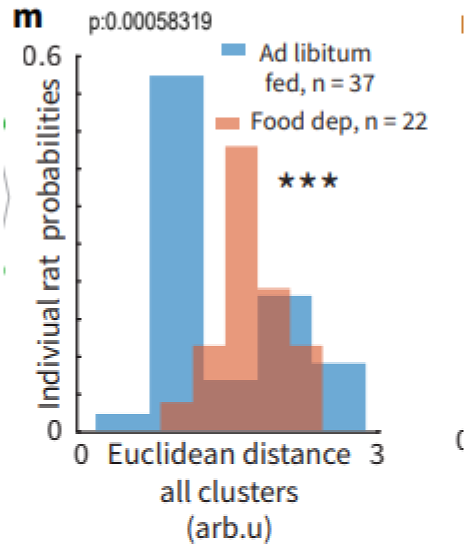

1. Navigate into “Updated Analysis” directory.
2. Navigate into “Utility Functions” directory.
3. Run the following matlab command in the command line
    a. “addpath(pwd)”
4. Navigate back up into the “Updated Analysis Directory”.
5. Run the file named runMe2.m.
6. Upon completion of running a directory named “First_and_last_bin_overlay_plots” will be created.
7. This figure will be inside “First_and_last_bin_overlay_plots”.
8. It will be named “Baseline vs Food_Deprivation.fig”.

Fig 5l

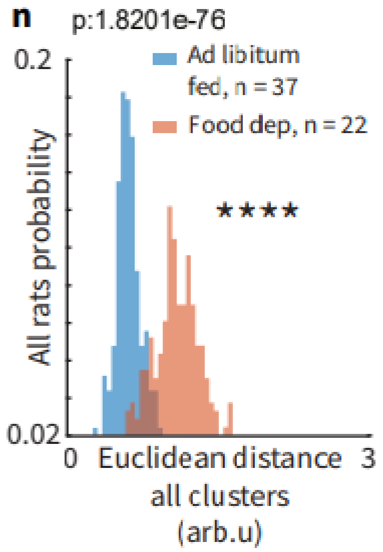

\

1) Navigate into “Updated Analysis” directory.
2) Navigate into “Utility Functions” directory.
3) Run the following matlab command in the command line
    a) “addpath(pwd)”
4) Navigate back up into the “Updated Analysis Directory”.
5) Run the file named runMe2.m.
6) Upon completion of running a directory named “Euc_Dist_All_Features”.
7) Inside “Euc_Dist_All_Features” will be this figure,named “Baseline Vs Food_deprivationAllFeatures.fig”.

Fig 6t

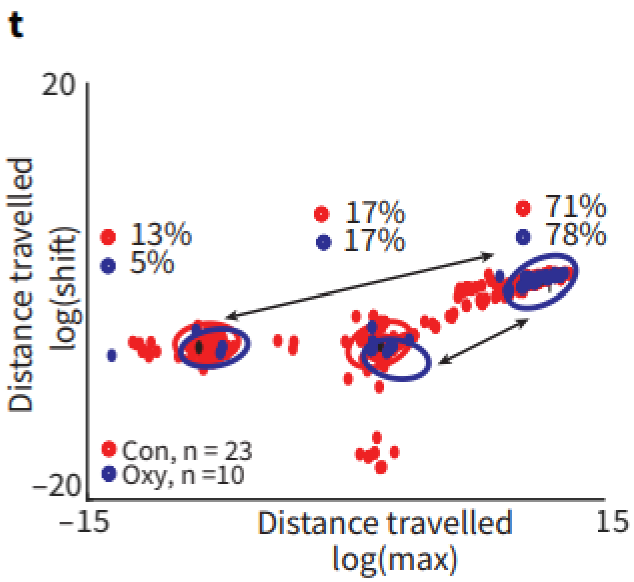

1) Navigate into “Updated Analysis” directory.
2) Navigate into “Utility Functions” directory.
3) Run the following matlab command in the command line
    a) “addpath(pwd)”
4) Navigate back up into the “Updated Analysis Directory”.
5) Run the file named runMe2.m.
6) This will be one of the figures created.

Fig 6u

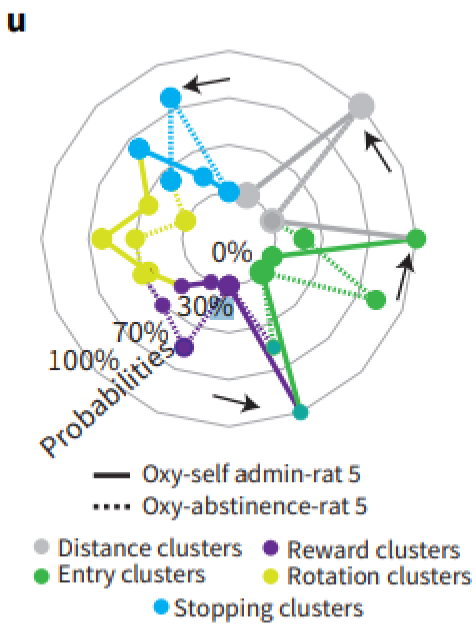

7) Navigate into “Updated Analysis” directory.
8) Navigate into “Utility Functions” directory.
9) Run the following matlab command in the command line
    a) “addpath(pwd)”
10) Navigate back up into the “Updated Analysis Directory”.
11) Run the file named runMe2.m.
12) Upon completion of running a directory named ““First_and_last_Bin_Spider_plots” will be created.
13) Navigate inside “First_and_last_Bin_Spider_plots”.
14) Inside will be the following directories:
    a) Baseline
    b) Boost_And_Etho
    c) Food_Deprivation
    d) Ghrelin

e) Oxy
f) Saline

15) It will be located within the directory named “Oxy” and will be named “12Oxy Early Bin barbie vs Oxy Late Bin barbie Spider Plot.fig”.

Fig 6v

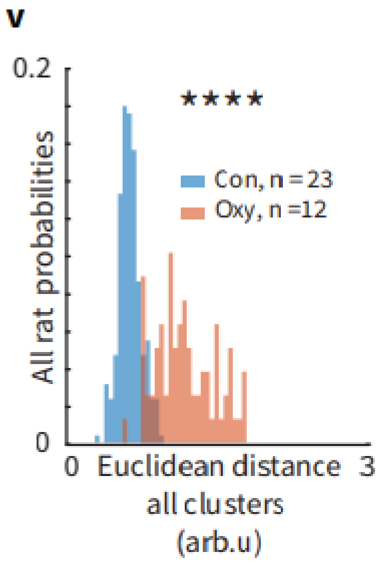

Navigate into “Updated Analysis” directory.

8) Navigate into “Utility Functions” directory.
9) Run the following matlab command in the command line
    a) “addpath(pwd)”
10) Navigate back up into the “Updated Analysis Directory”.
11) Run the file named runMe2.m.
12) Upon completion of running a directory named “Euc_Dist_All_Features”.
13) Inside “Euc_Dist_All_Features” will be this figure,named “Baseline Vs OxyAllFeatures.fig”.

Fig 7k

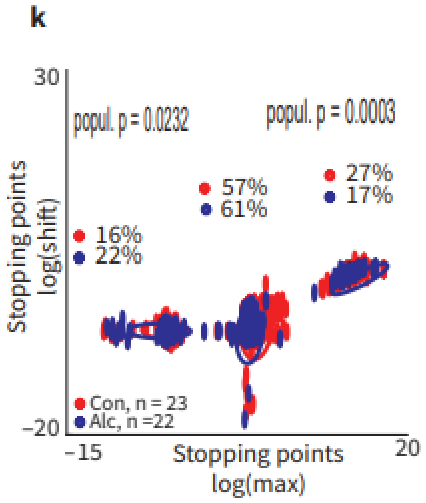

1) Navigate into "Updated Analysis" directory.
2) Navigate into "Utility Functions" directory.
3) Run the following matlab command in the command line
    a) "addpath(pwd)"
4) Navigate back up into the "Updated Analysis Directory".
5) Run the file named runMe2.m.
6) This will be one of the figures created.

Fig 7l

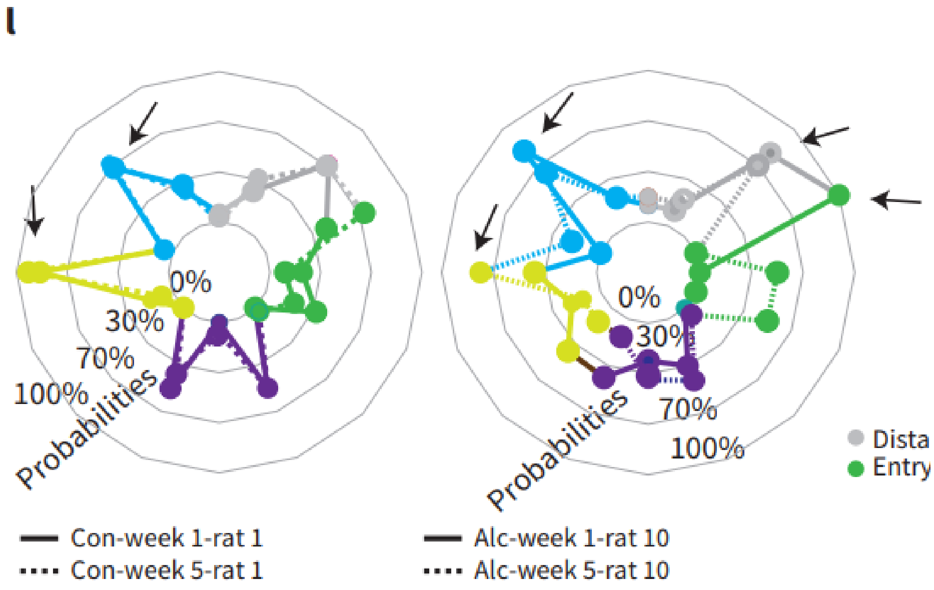

1) Navigate into “Updated Analysis” directory.
2) Navigate into “Utility Functions” directory.
3) Run the following matlab command in the command line
    a) “addpath(pwd)”
4) Navigate back up into the “Updated Analysis Directory”.
5) Run the file named runMe2.m.
6) Upon completion of running a directory named “First_and_last_Bin_Spider_plots” will be created.
7) Navigate inside “First_and_last_Bin_Spider_plots”.
8) Inside will be the following directories:
    a) Baseline
    b) Boost_And_Etho
    c) Food_Deprivation
    d) Ghrelin
    e) Oxy
    f) Saline
9) The left panel will be located in the “Baseline” Directory, and it will be named “1Baseline Early Bin sarah vs Baseline Late Bin sarah Spider Plot.fig”.
10) The right panel will be located in the “Boost_And_Etho” directory, and it will be named “19BoostAndEtho Early Bin sully vs BoostAndEtho Late Bin sully Spider Plot.fig”.

Fig 7m

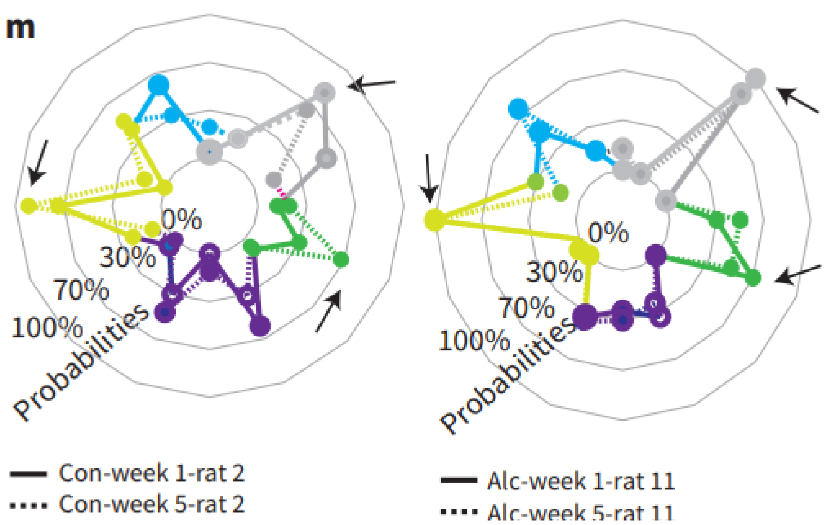

1) Navigate into “Updated Analysis” directory.
2) Navigate into “Utility Functions” directory.
3) Run the following matlab command in the command line
    a) “addpath(pwd)”
4) Navigate back up into the “Updated Analysis Directory”.
5) Run the file named runMe2.m.

6) Upon completion of running a directory named “First_and_last_Bin_Spider_plots” will be created.
7) Navigate inside “First_and_last_Bin_Spider_plots”.
8) Inside will be the following directories:
    a) Baseline
    b) Boost_And_Etho
    c) Food_Deprivation
    d) Ghrelin
    e) Oxy
    f) Saline
9) The left panel will be located in the “Baseline” Directory, and it will be named “13Baseline Early Bin kobe vs Baseline Late Bin kobe Spider Plot.fig”.
10) The right panel will be located in the “Boost_And_Etho” directory, and it will be named “1BoostAndEtho Early Bin kobe vs BoostAndEtho Late Bin kobe Spider Plot.fig”.

Fig 7n

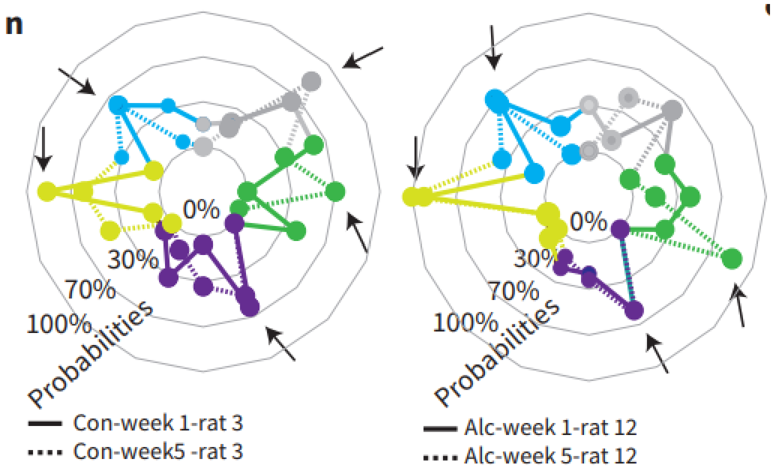

1) Navigate into “Updated Analysis” directory.
2) Navigate into “Utility Functions” directory.
3) Run the following matlab command in the command line
    a) “addpath(pwd)”
4) Navigate back up into the “Updated Analysis Directory”.
5) Run the file named runMe2.m.
6) Upon completion of running a directory named “First_and_last_Bin_Spider_plots” will be created.
7) Navigate inside “First_and_last_Bin_Spider_plots”.
8) Inside will be the following directories:
    a) Baseline
    b) Boost_And_Etho
    c) Food_Deprivation

d) Ghrelin
e) Oxy
f) Saline

9) The left panel will be located in the “Baseline” Directory, and it will be named “23Baseline Early Bin mike vs Baseline Late Bin mike Spider Plot.fig”.
10) The right panel will be located in the “Boost_And_Etho” directory, and it will be named “12BoostAndEtho Early Bin renata vs BoostAndEtho Late Bin renata Spider Plot.fig”.

Fig 7p

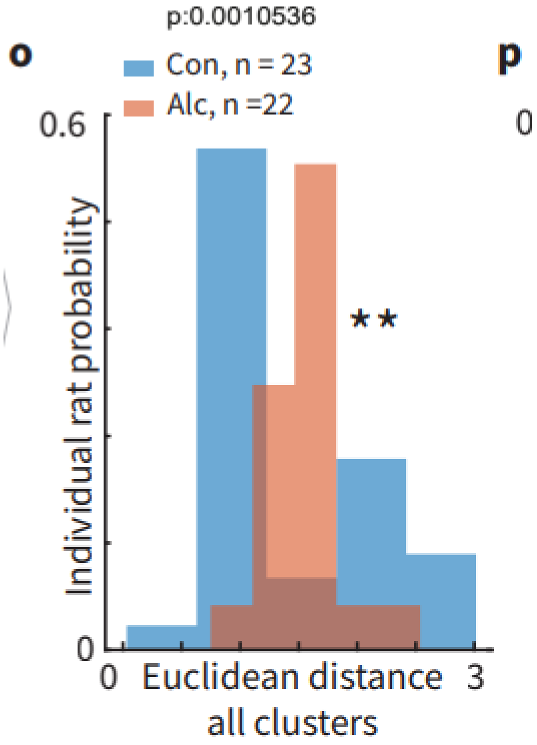

1) Navigate into “Updated Analysis” directory.
2) Navigate into “Utility Functions” directory.
3) Run the following matlab command in the command line
    a) “addpath(pwd)”
4) Navigate back up into the “Updated Analysis Directory”.
5) Run the file named runMe2.m.
6) Upon completion of running, a directory named “First_and_last_bin_overlay_plots” will be created.
7) Inside of it will be this figure, named “Baseline vs Boost_And_Etho.fig”.

Fig 7o

p

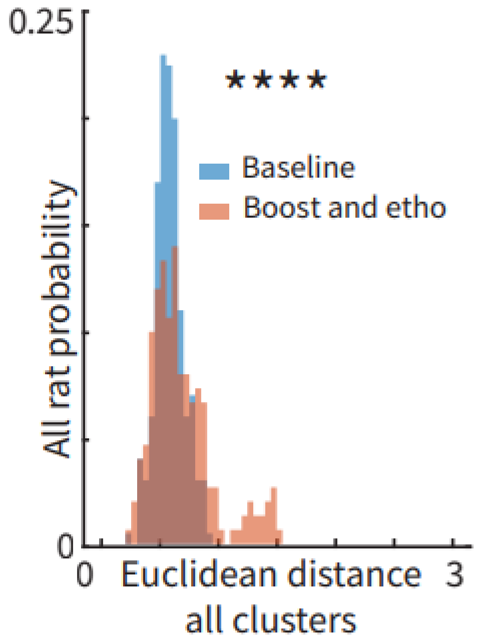

1) Navigate into “Updated Analysis” directory.
2) Navigate into “Utility Functions” directory.
3) Run the following matlab command in the command line
    a) “addpath(pwd)”
4) Navigate back up into the “Updated Analysis Directory”.
5) Run the file named runMe2.m.
6) Upon completion of running a directory named “Euc_Dist_All_Features”.
7) Inside “Euc_Dist_All_Features” will be this figure,named “Baseline Vs Boost_And_EthoAll Features.fig”.

Fig SF4b

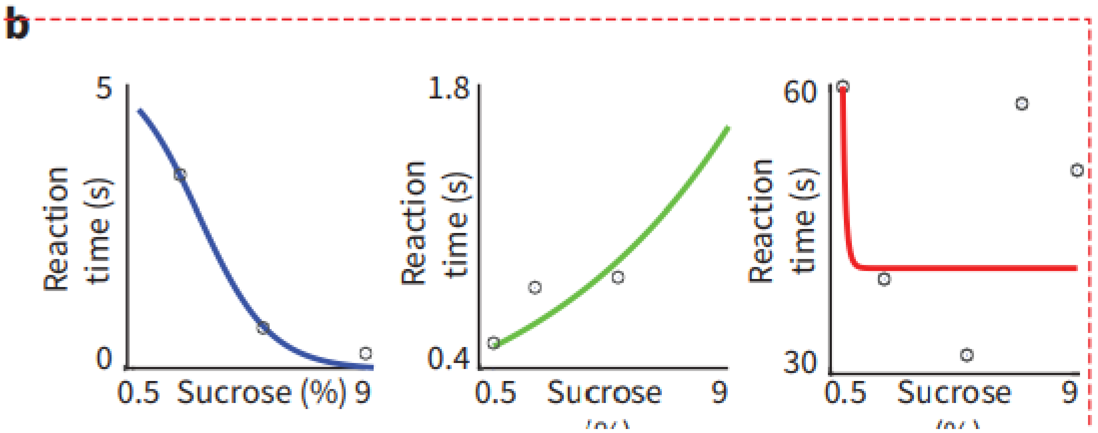

1. Navigate into Data Analysis directory.
2. Navigate into Old Base Data
3. The directories Reward Choice 2 Parameter Sigmoid, Reward Choice 3 Parameter Sigmoid, Reward Choice 4 Parameter Sigmoid contain all sigmoid functions created from raw data including those seen above.

Fig SF4c

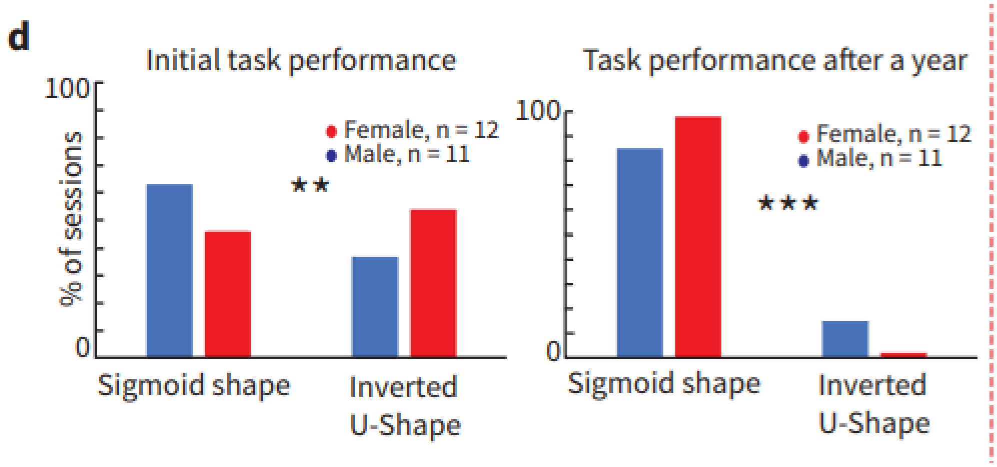

1. This figure was created by counting the total number of sessions, the total number of sessions fitted with a sigmoid, and the total number of sessions fit with a parabola. Then it is simply a matter of dividing the latter two by the former.

Fig SF5o

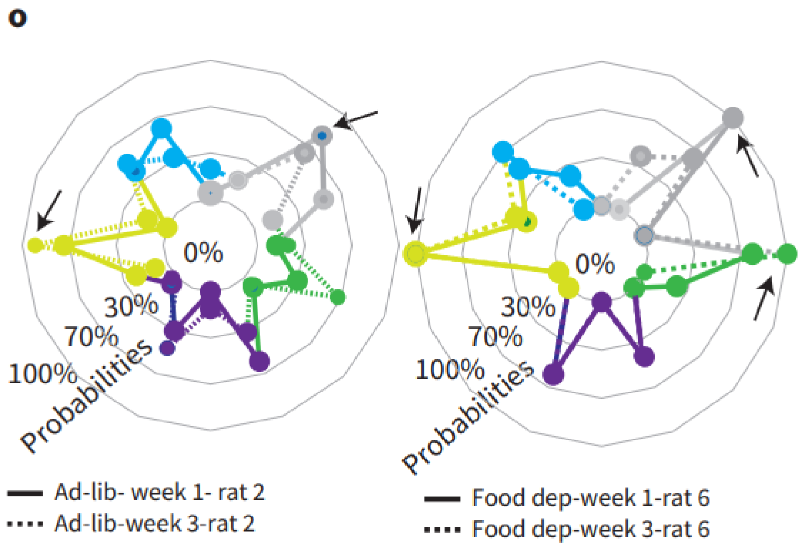

Navigate into “Updated Analysis” directory.

16) Navigate into “Utility Functions” directory.
17) Run the following matlab command in the command line
    a) “addpath(pwd)”
18) Navigate back up into the “Updated Analysis Directory”.
19) Run the file named runMe2.m.
20) Upon completion of running a directory named ““First_and_last_Bin_Spider_plots” will be created.
21) Navigate inside “First_and_last_Bin_Spider_plots”.
22) Inside will be the following directories:
    a) Baseline
    b) Boost_And_Etho
    c) Food_Deprivation
    d) Ghrelin
    e) Oxy
    f) Saline
23) The left panel will be located in the “Baseline” Directory, and it will be named “13Baseline Early Bin kobe vs Baseline Late Bin kobe Spider Plot.fig”.
24) The right panel will be located in “Food Deprivation” directory, and it will be named “2FoodDeprivation Early Bin jimi vs FoodDeprivation Late Bin jimi Spider Plot.fig”

Fig SF5p

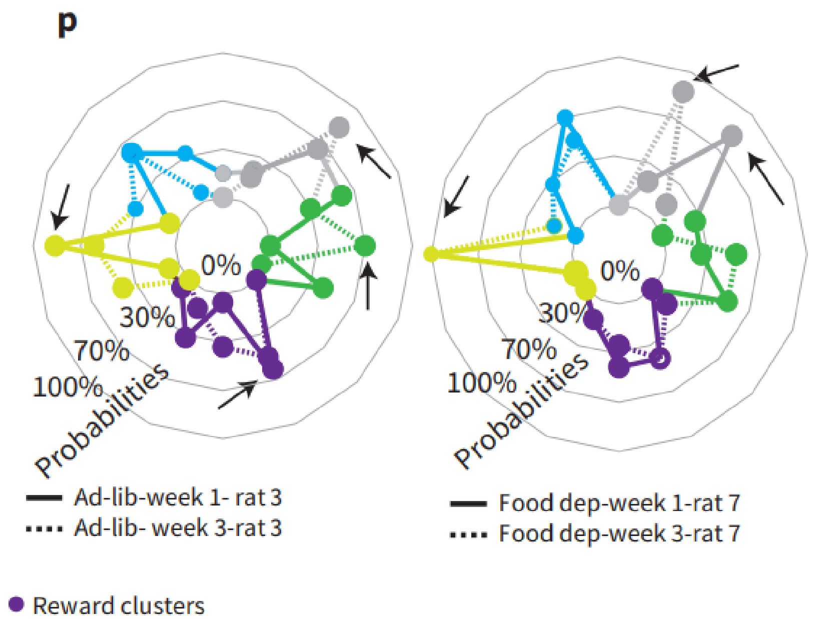

Navigate into “Updated Analysis” directory.

25) Navigate into “Utility Functions” directory.
26) Run the following matlab command in the command line
    a) “addpath(pwd)”
27) Navigate back up into the “Updated Analysis Directory”.
28) Run the file named runMe2.m.
29) Upon completion of running a directory named ““First_and_last_Bin_Spider_plots” will be created.
30) Navigate inside “First_and_last_Bin_Spider_plots”.
31) Inside will be the following directories:
    a) Baseline
    b) Boost_And_Etho
    c) Food_Deprivation
    d) Ghrelin
    e) Oxy
    f) Saline
32) The left panel will be located in the “Baseline” Directory, and it will be named “23Baseline Early Bin mike vs Baseline Late Bin mike Spider Plot.fig”.
33) The right panel will be located in “Food Deprivation” directory, and it will be named “7FoodDeprivation Early Bin harley vs FoodDeprivation Late Bin harley Spider Plot.fig”

Fig SF5q, SF5r

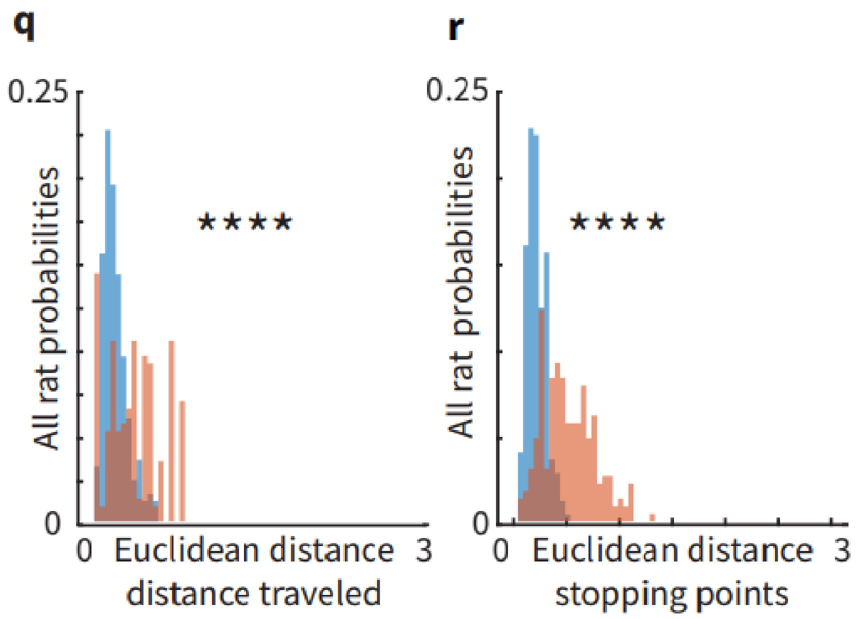

Navigate into “Updated Analysis” directory.

1) Navigate into “Utility Functions” directory.
2) Run the following matlab command in the command line
    a) “addpath(pwd)”
3) Navigate back up into the “Updated Analysis Directory”.
4) Run the file named runMe2.m.
5) Upon completion of running a directory named “Euc_Distance_Plots” will be created.
6) Inside of “Euc_Distance_Plots”, the left panel will be in saved in a figure named “DT_ Baseline Vs Food_Deprivation”.
7) Inside of “Euc_Distance_Plots” the right panel will be saved in a figure named “SP_ Baseline Vs Food_Deprivation.fig”.

Fig SF6i

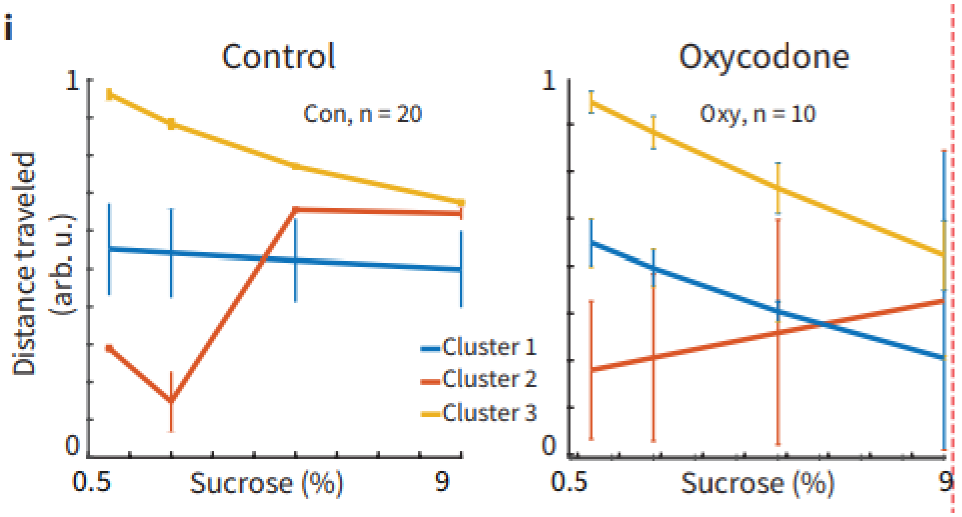

1. Navigate into Create Probability Tables directory.
2. Run createFigSF6i.m.

Fig SF6j

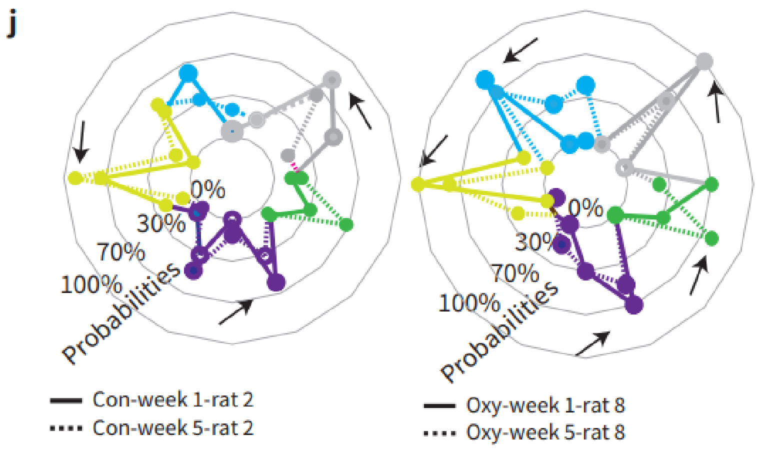

Navigate into “Updated Analysis” directory.

1) Navigate into “Utility Functions” directory.
2) Run the following matlab command in the command line
    a) “addpath(pwd)”
3) Navigate back up into the “Updated Analysis Directory”.
4) Run the file named runMe2.m.
5) Upon completion of running a directory named ““First_and_last_Bin_Spider_plots” will be created.
6) Navigate inside “First_and_last_Bin_Spider_plots”.
7) Inside will be the following directories:
    a) Baseline
    b) Boost_And_Etho
    c) Food_Deprivation
    d) Ghrelin
    e) Oxy
    f) Saline
8) The left panel will be located in the “Baseline” Directory, and it will be named “13Baseline Early Bin kobe vs Baseline Late Bin kobe Spider Plot.fig”,
9) The right panel will be in the “Oxy directory”, and it will be named “2Oxy Early Bin ken vs Oxy Late Bin ken Spider Plot.fig”.

Fig SF6k

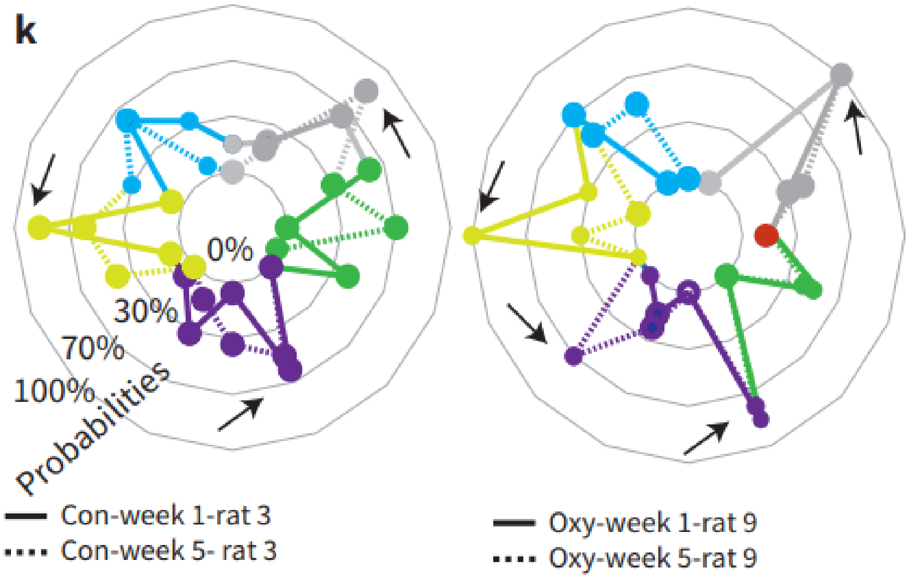

11) Navigate into “Updated Analysis” directory.
12) Navigate into “Utility Functions” directory.
13) Run the following matlab command in the command line
    a) “addpath(pwd)”
14) Navigate back up into the “Updated Analysis Directory”.
15) Run the file named runMe2.m.
16) Upon completion of running a directory named “First_and_last_Bin_Spider_plots” will be created.
17) Navigate inside “First_and_last_Bin_Spider_plots”.
18) Inside will be the following directories:
    a) Baseline
    b) Boost_And_Etho
    c) Food_Deprivation
    d) Ghrelin
    e) Oxy
    f) Saline
19) The left panel will be located in the “Baseline” Directory, and it will be named “23Baseline Early Bin mike vs Baseline Late Bin mike Spider Plot.fig”.
20) The right panel will be located in the “Oxy” directory, and it will be named “6Oxy Early Bin bopeep vs Oxy Late Bin bopeep Spider Plot.fig”.

Fig SF6I

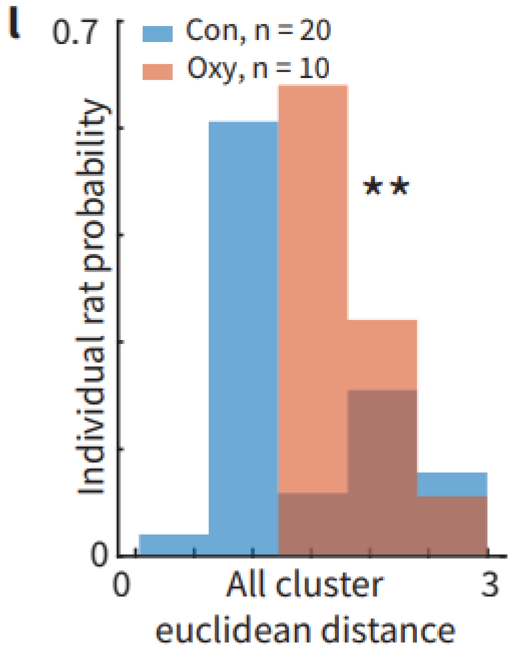

p:0.0011049

1) Navigate into “Updated Analysis” directory.
2) Navigate into “Utility Functions” directory.
3) Run the following matlab command in the command line
    a) “addpath(pwd)”
4) Navigate back up into the “Updated Analysis Directory”.
5) Run the file named runMe2.m.
6) Upon completion of running, a directory named “First_and_last_bin_overlay_plots” will be created.
7) Inside of it will be this figure, named “Baseline vs Oxy.fig”.

Supplemental note 6

Protocol for Running Automatic Trials

Last updated: April 14th

Set-up & Running Experimental Trials

Note:** Don't get animals from the holding room until you're ready to put them in for detection. settings (Steps 10 & 11). First, run the ***system set-up below.

System set-up:

1. **Make solutions** for all arenas and make any if necessary (each syringe should be at least half full after the system check, before any given session).
2. Turn on **powerstrips** (underneath table).
3. Plug in the **noldus box** (back of Noldus box, bottom right, labeled "in").
4. Turn on **MCUs** (usb hub, right button → blue lights will turn on around the MCU usb in use).
5. Turn on **infrared lamps** (one is *by itself* next to the **Noldus Box** and the others are connected to two surge protectors to the *left of arenas 1 and 3*).
6. Make sure *lights and feeders* are working by going to **Documents → Noldus → MCU Commands → File → Open Windows Powershell**
    a. Then type **"./system_check FR2355"** [or 2355_2, 2355_3, 2355_4] The program will then run a valve and light check sequence. Be sure water is coming out from all feeders, and all lights are turning on for each maze.
        i. Hit Ctrl C to terminate the batch job and Y to confirm, then you can rerun the **"./system_check FR[ID#]"**
    b. Hit enter and the program will run, checking valves and feeder lights. Do this for all four mazes.
    c. Recheck syringes to make sure they're full after running system checks for each arena.

Important: If only 2-3 mazes are working that day then put fake rats in mazes that don't work, name as 'none' and proceed with what's working. E.g. when maze 1 isn't working, we put the fake rat in there and run trials with 2, 3, and 4.

Running trials:

7. Load EthoVisionXT and select correct **experiment:**

   a. Right now it is labeled as **"Automatic trials" and "Automatic trials females"**

   b. Close other programs (e.g. MATLab).

   c. Make sure you go to task manager (ctrl→ alt → delete) and set EthoVision's priority to "realtime"

8. Go to **"Trial List"**

a. Create/check the sequence of trials that you're going to run. Enter the TC Settings, Animal Names and User-Defined Feeder (1-4) and Light Level (0-3) variables. Check reward amounts and lux values per maze, by feeder (all variables to the right of the trial list table). Make sure that everything is filled out and matches.
Note: *The versions of the TC settings will change over time as we modify our task. This, for instance, is the trial list we were running last november - and, if you compare it to ours now, you'll see that we're using a different phase with a lot more variables.*

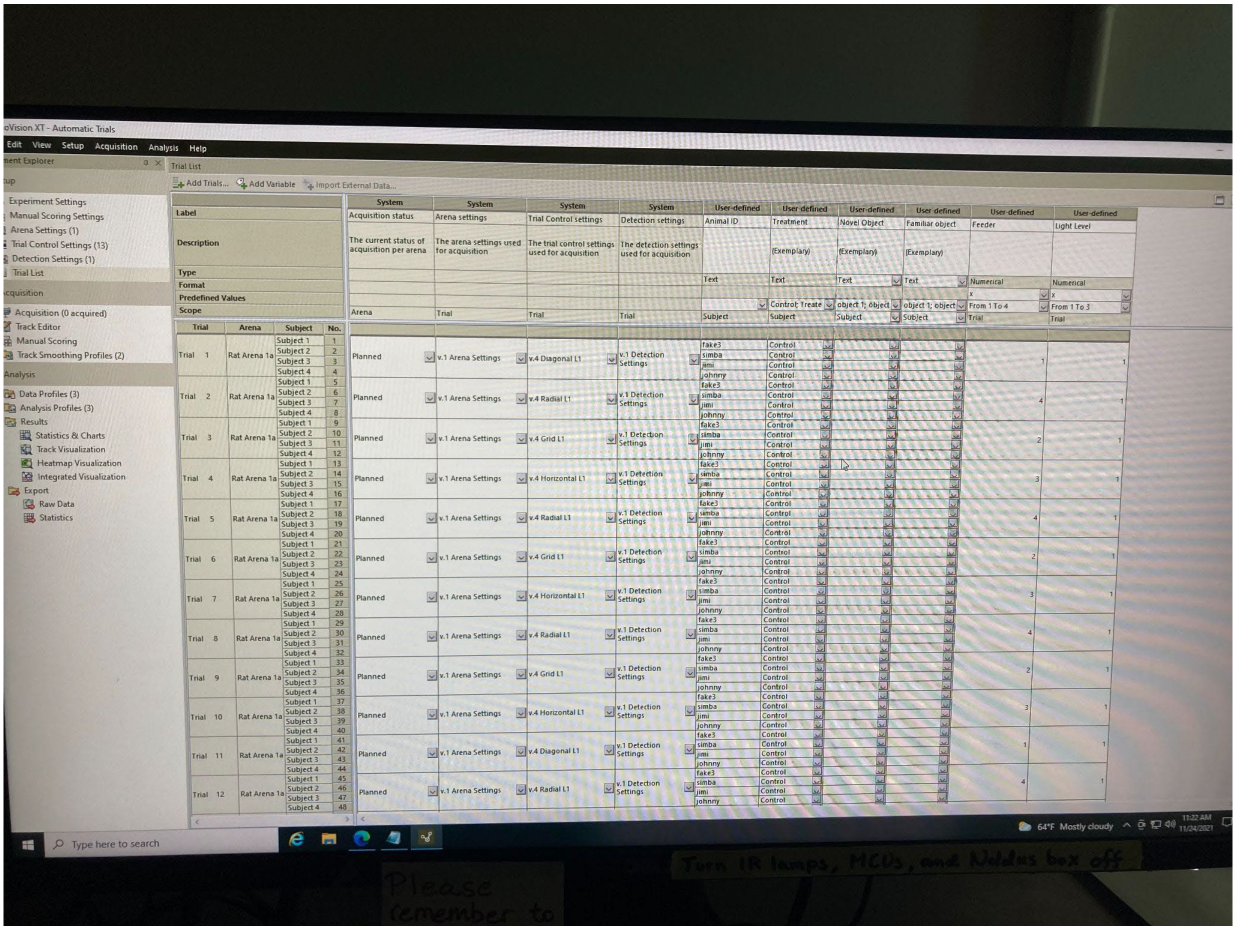

9. Get animals and put them into the arenas (cage 1 into arena 1 and 2, and cage 2 into arena 3 and 4).

10. Select Detection Settings by going to **"Detection Settings"** selecting "**v.1 Detection**

Settings". *This should automatically detect the animals with our pre-saved settings.* If you ever need to re-do them, then follow these steps:

a. Place your animals in one-by one, and click **"Automated Setup".**

b. Then drag boxes around your four animals, finetune your detection results if necessary and click ok.

c. If you need to, mess with advanced settings under “Method, Smoothing, & Subject Contour” and make sure that you have “unmarked subjects” selected.

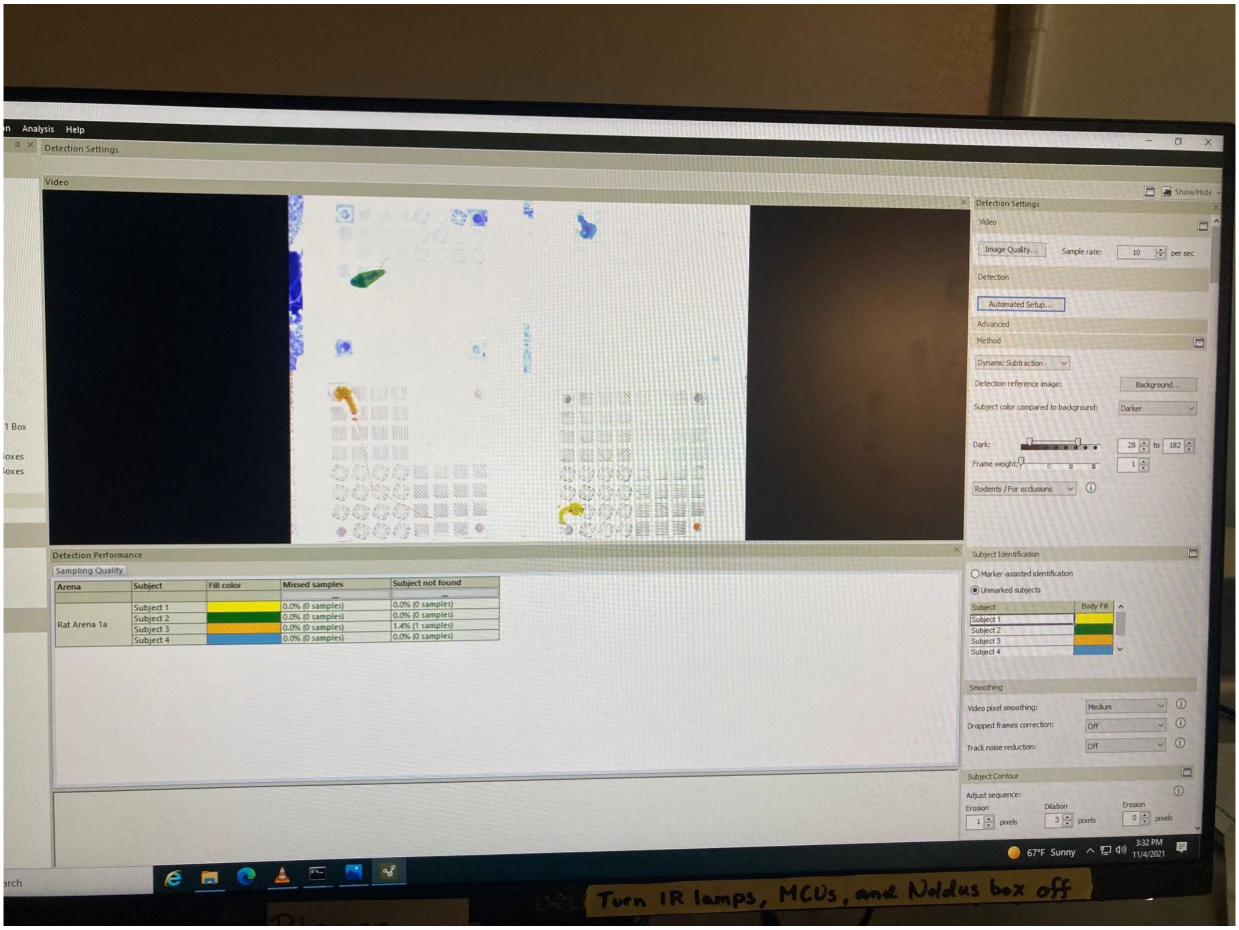

11. Go to **“Acquisition”** and, using the image as a reference, follow these steps:

a. Be sure to select **“track all-planned trials”** in the menu on the right-hand side.

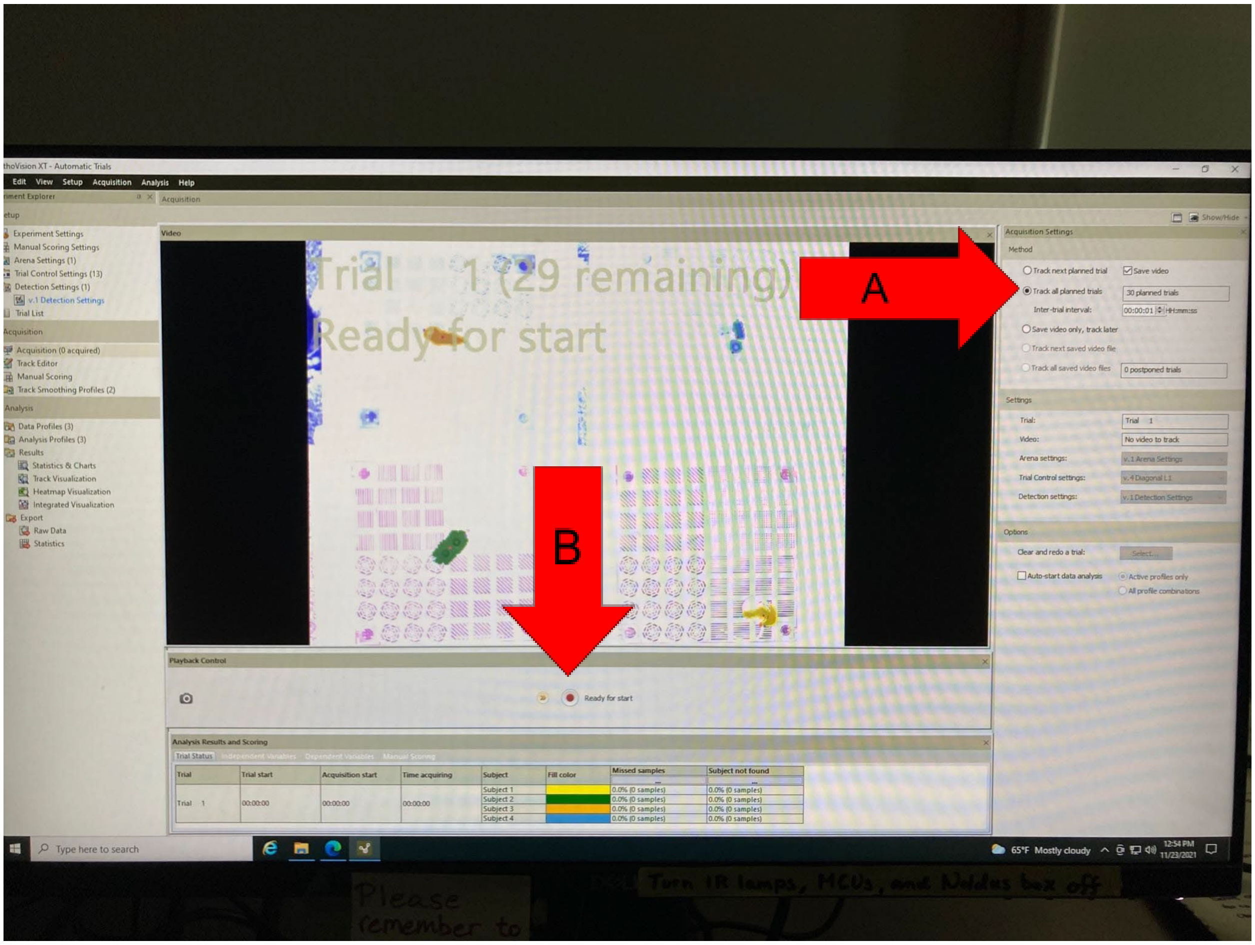

b. Click **"Ready for Start"** button in middle of screen.
c. Let program run, various code (command line) windows will pop up, run, and close themselves.

12. At the end of the trial, a pop-up window will say "trial run finished". Click "ok".

13. Go to **"Analysis"**, click on **"export" → "raw data"**
    a. Make sure you have the following checkboxes selected:

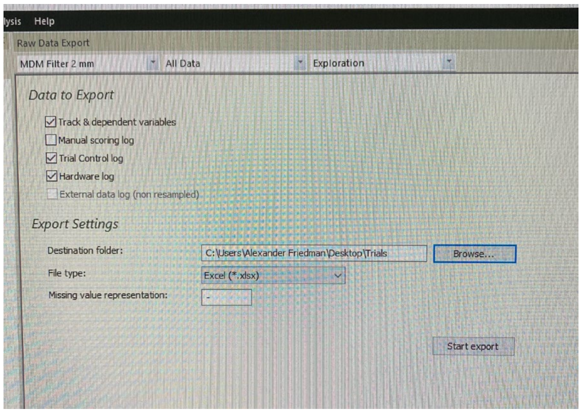
lysis Help
Raw Data Export
MDM Filter 2 mm
All Data
Exploration
Data to Export
Track & dependent variables
Manual scoring log
Trial Control log
Hardware log
External data log (non resampled)
Export Settings
Destination folder:
C:\Users\Alexander Friedman\Desktop\Trials
Browse...
File type:
Excel (*.xlsx)
Missing value representation:
Start export

b. Export as an excel file to the desktop folder: “**Trials**”.
    i. Then go to ‘**Trials**’ folder on the desktop and create/add to a folder by date and sessions (see examples - for how to format - within Trials folder).

14. Running trials will lock experimental settings (arena, trial control and detection). To unlock and make changes after exporting data and before starting a new session (e.g. if you bump a maze and need to redo arena and detection settings after moving it back) go to options on the right side and click the box that says **“select”** next to **“Clear and Redo Trial ”**, select clear tracks and video click ok.

Shut-down

After trials have been run, data exported, and animals put away, do the following:

1. **Clean mazes** (wet a paper towel with alcohol and wipe down mazes).
2. Turn off **cameras** (power strips underneath the table behind the Noldus I/O box)
3. Turn off **MCUs** (usb hub).
4. Unplug **Noldus/IO box**. Black box with black ethernet cord looking-things, take the semi-clear plug out from the back (which will be facing you).
5. Turn off **infrared lamps** (two power strips on the wall by the PCB box stand).
6. Close **ethovision**.
7. Turn off **all lights**.

Shut-Down for the Last Session of the Day

1. **Complete steps 1-7 as you normally would.**
2. Suck the sucrose out of the syringe holders on the valve stands (using big syringes found in the sucrose beaker tray). Dump all left over sucrose into sink and let beakers air dry overnight.
3. At the end of every Friday, run 20mL alcohol through valves using PuTTY. Followed by 20mL of warm water.
4. Biweekly: change pads.

Supplemental Note 7

Animal Behavior Protocol

1. Habituation
    a. Begin handling as early as possible (2 weeks of age preferred)
    b. Habituation takes 1-2 weeks
        i. Depends on animal
    c. Introduce hands into cage
        i. Animal will approach and smell you
        ii. Nibbling at globe and gown is expected
        iii. Stroke animal
            1. Behind ears or behind front legs
    d. Carrying animal
        i. Scoop
            1. Shape dominant hand into scoop
            2. Place hand underneath animals belly
            3. Non dominant hand goes on animals rear to support animal while picking up
        ii. Tail pick up
            1. Place dominant hand on tail as close to the base of the tail as possible
                a. Tail will deglove if not held at base
            2. Pick animal up by tail
            3. Non dominant hand is placed at belly as soon as animal is picked up
                a. This will stabilize the animal
            4. Animal jumping out of cage
                a. Animal will jump out of cage into hands
                b. Will jump back into cage
                c. Animal will stay in hands once it is habituated to you
        iii. Holding animal
            1. After picking up animal place on body
            2. Hold for 5 seconds
            3. Animal will roam around
                a. Allow to happen as long as animal is secure
                b. Animal will roam more as it is more comfortable
    e. Putting down animal
        i. Hands need to be on maze/cage bottom and animal needs to walk off hands
    f. Habituated behavior
        i. Grooming
        ii. Rearing
        iii. Roaming
        iv. Staying in handler’s hands for 10 seconds or more

g. Stressful behavior
    i. Excessive grooming (longer than 2 minutes)
    ii. Grabbing tail
    iii. Hunching over
    iv. Rubbing body on maze walls

2. Trial training
    a. Phase 1: Reward-Reward
        i. Sucrose solution was introduced to maze feeders
            1. Each feeder has a designated solution
        ii. Animal is introduced and allowed to roam around
        iii. After ~60 trials the animal will begin to form a preference
            1. Animal will go to that corner the moment it is introduced into the maze
            2. Will go to that solution percentage over 5/10 times (if it is it's preferred solution)
    b. Phase 2: Reward-Reward Automatic
        i. Sucrose solution was introduced to maze feeders
            1. Each feeder has a designated solution
        ii. Animal is introduced and allowed to roam around iii.
